# Benzoic Acid Derivatives Improve Plasma Stability of Diester Butyrophilin Ligand Prodrugs

**DOI:** 10.1021/acs.jmedchem.5c03034

**Published:** 2026-02-12

**Authors:** Parker A. Kintigh, Umed Singh, Girija Pawge, Sidra Bashir, Chia-Hung Christine Hsiao, Andrew J. Wiemer, David F. Wiemer

**Affiliations:** † Department of Chemistry, 4083 University of Iowa, Iowa City, Iowa 52242-1294, United States; ‡ Department of Pharmaceutical Sciences, 7712 University of Connecticut, Storrs, Connecticut 06269-3092, United States; § Institute for Systems Genomics, University of Connecticut, Storrs, Connecticut 06269-3092, United States

## Abstract

The potent butyrophilin ligand, (*E*)-4-hydroxy-3-methyl-but-2-enyl diphosphate (HMBPP), is a potential cancer immunotherapy agent, but it lacks plasma stability and membrane permeability. Aryl phosphonamidate prodrugs of a key HMBPP analog have improved plasma stability but poor cellular uptake, while aryl phosphonester prodrugs have improved uptake but lack plasma stability. Here, tuning the benzoic acid substructure of a phosphonester prodrug was explored. Twenty-one aryl phosphonester derivatives were prepared in allylic alcohol (**8a–k**) and allylic acetate (**9a–k**) forms. Testing revealed that this strategy can provide compounds with high potency for expansion of γ9δ2 T cells (**8d**, EC_50_ = 0.86 nM) and interferon γ production in response to loaded K562 cells (**8d**, EC_50_ = 2.3 nM). Importantly, these compounds display improved plasma stability (130-fold range; **8d**, *t*_1/2_ > 24 h), showing the importance of the benzoic acid position for plasma versus cellular metabolism. These findings enable next-generation prodrugs with improved stability and potency.

## Graphical abstract

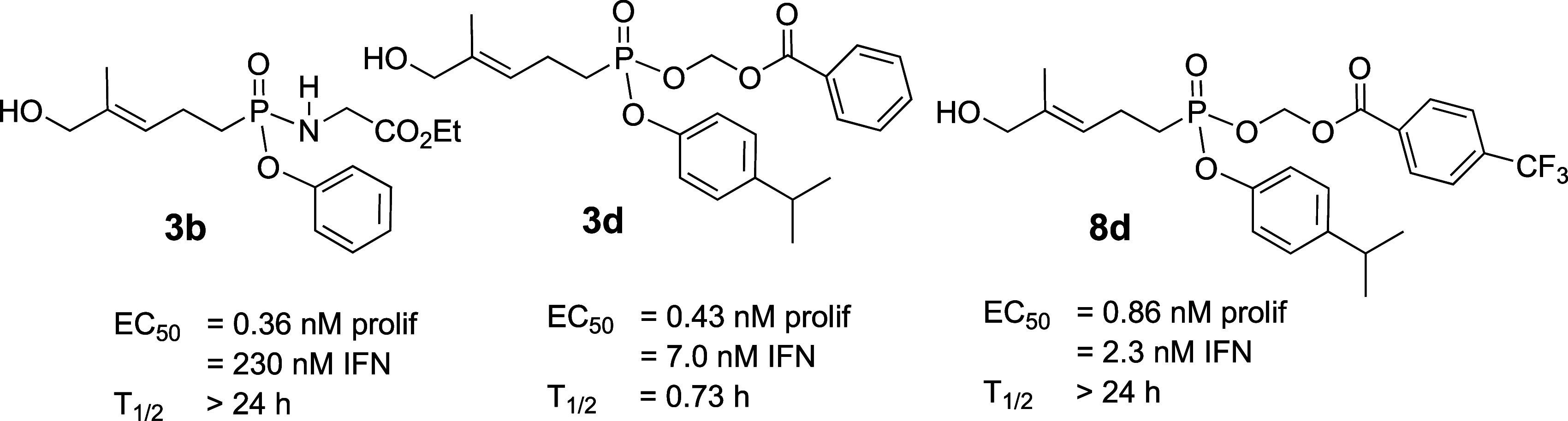

## Introduction

Butyrophilin (BTN) complexes play critical roles in the detection of phosphoantigens, influencing T cell activation.^1,2^ These phosphoantigens act like molecular glues (Figure 1), promoting the interaction between BTN3A1 and BTN2A1 to form active complexes.^3,4^ This association occurs between the intracellular domains of these transmembrane proteins, which promotes changes to the conformation of their extracellular domains. In the active form, the complexes can be detected by the T cell receptor of γ9δ2 T cells, stimulating their activation.^5^ At the same time, the active complexes can interact with CD45 on CD8 T cells to remove an inhibitory checkpoint to their activation.^6^ As such, phosphoantigens have untapped clinical potential to boost T cell responses in indications such as cancer.

**Figure 1:**
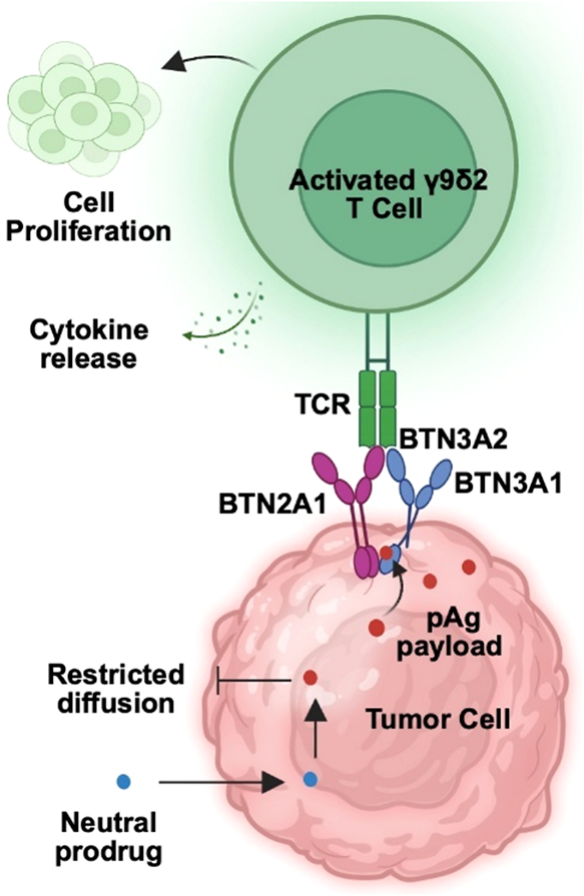
Activation of γ9δ2 T cells by phosphoantigen prodrugs. Prodrugs deliver the charged payloads to the cytoplasm, where they act like molecular glues to the BTN2A1 and BTN3 cytoplasmic domains, forming a tetrameric butyrophilin complex that engages the γ9δ2 T cell receptor. T cell receptor signaling activates the T cells and leads to functions such as proliferation and cytokine production.

The most potent naturally occurring phosphoantigen is (*E*)-4-hydroxy-3-methyl-but-2-enyl diphosphate (HMBPP, **1**, Figure 2).^7,8^ This molecule is an essential intermediate of isoprenoid biosynthesis in bacteria and other microorganisms,^9^ but is not found in human metabolism.^10^ The charged headgroup is critical to butyrophilin binding,^11^ but although there are some exceptions^12^ in most cases diphosphates exhibit poor drug-like properties because they are rapidly degraded and have limited ability to diffuse across biological membranes. To improve the drug-like properties of a butyrophilin ligand, we have replaced the diphosphate with a phosphonate to improve metabolic stability (i.e., **2**) and protected the phosphonate with cell-cleavable prodrug groups to improve its ability to cross membranes (e.g., **3a**,^13^ **3b**,^14^ **3c–e** ^15^). These studies have demonstrated that a ligand with one charged phosphorus is sufficient for potent biological activity, although the possibility that the phosphonate ligand is phosphorylated in living cells cannot be excluded. In either case, the key phosphonate ligand is a very useful probe, but the potency and stability of the prodrugs could be improved further. Specifically, our goal is to push the boundaries of both rapid intracellular activation and high plasma stability.

**Figure 2:**
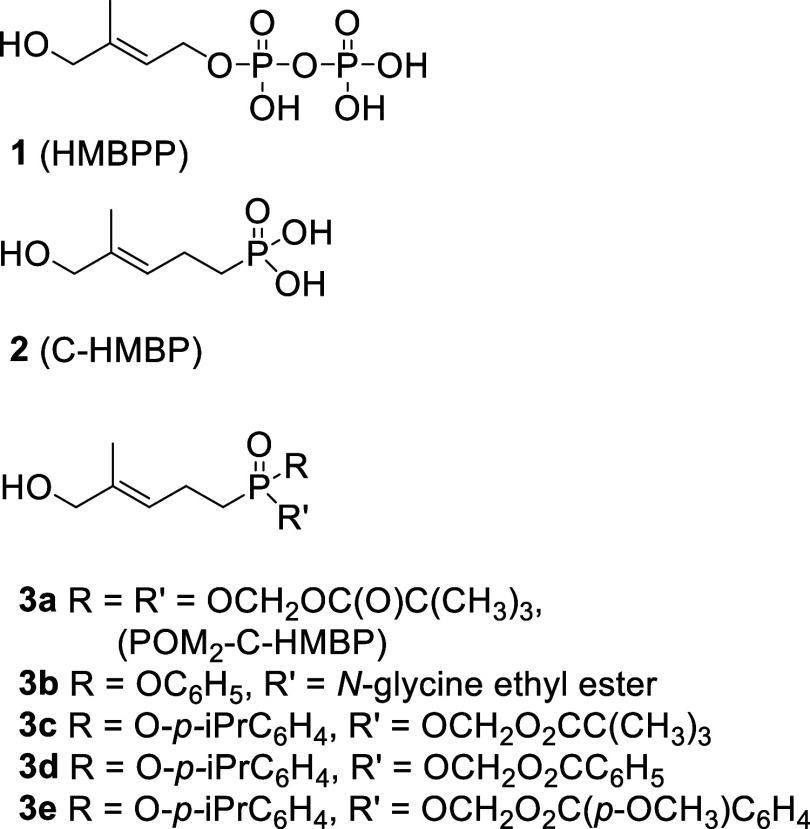
Natural phosphoantigen HMBPP (**1**), a phosphonate analog (**2**), and relevant prior prodrug forms (**3a–e**) with measured potencies for stimulation of γ9δ2 T cells (EC_50_, nM). Published 72 h proliferation EC_50_ values: **1** = 0.5 nM, **2** = 4000 nM, **3a** = 5.4 nM, **3b** = 0.36 nM, **3c** = 1.9 nM, **3d** = 0.43 nM, and **3e** = 1.5 nM. Published 1 h cytokine production EC_50_ values: **1** > 100,000 nM, **2** > 100,000 nM, **3a** = 30 nM, **3b** = 230 nM, **3c** = 3.8 nM, **3d** = 7 nM, and **3e** = 6.4 nM.^13–15,27,28^

Since the approval of fosinopril in 1991, at least six phosphate or phosphonate prodrugs have reached clinical approval.^16^ Earlier versions of phosphate prodrugs such as adefovir dipivoxil^17^ contain the pivaloyloxymethyl (POM)^18^ groups, which improve oral availability but lack plasma stability, releasing the payload into the blood. Later phosphoramidate derivatives of phosphates, such as sofosbuvir^19^ improved the plasma stability to allow distribution to the liver upon oral delivery. Remdesivir,^20^ also a phosphoramidate derivative of a modified nucleotide, provided a new mode of action with increased distribution to the lung following IV delivery.

Perhaps because of the success of nucleoside phosphoramidates^21^ such as sofosbuvir and remdesivir, many in the field view phosphonamidates as superior to ester prodrug forms of phosphonates due to their higher plasma stability.^22–25^ Nevertheless, others have shown that highly lipophilic esters can effectively deliver triphosphate nucleotides.^26^ We previously found that phosphonate diester forms have faster activation kinetics, and suspect that diester forms with improved plasma stability may offer attractive alternatives to phosphonamidate forms.^15,27^ We have described diester prodrugs that contain one *para-*isopropylphenyl group and one acyloxy aryl group containing benzoic acid, which have exceptional potency (e.g., **3d**, 0.43 nM EC_50_ and **3e**, 1.5 nM EC_50_, Figure 2).^27^ Unfortunately, these past compounds exhibited poor plasma stability, with compound **3d** displaying a half-life in plasma of only 44 min (0.73 h). Interestingly, compound **3e** did show an improved half-life of 8.3 h, but its potency was not among the best we have tested. Based on these findings, we hypothesized that the hydrolysis of the benzoic acid is the rate-limiting step for payload release and that by decorating the benzoic acid substructure with various functional groups, we could find combinations that improve plasma stability while retaining cellular potency (Figure 3). In this report, we disclose an impressive increase in plasma stability, which opens new possibilities for the design and application of phosphonester prodrugs.

**Figure 3:**
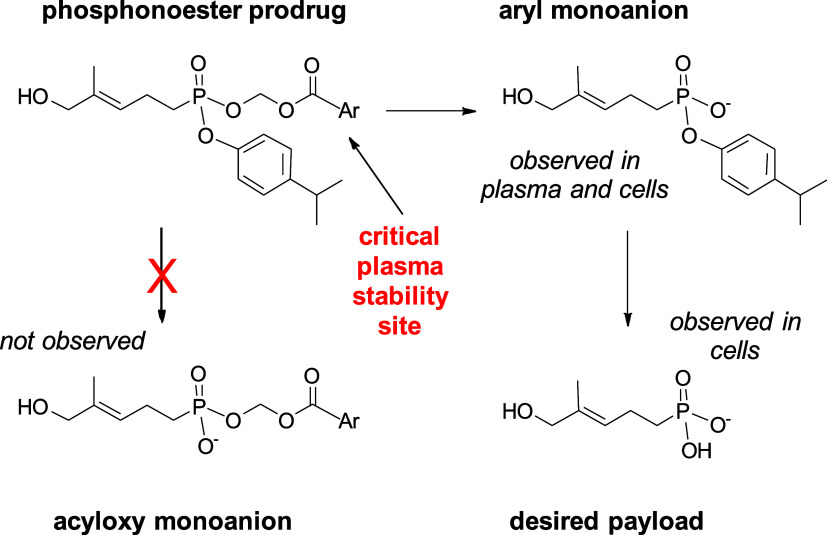
Metabolism of phosphonester prodrugs as determined by LC-MS. Release of the desired payload occurs in two steps via the aryl monoanion intermediate. Both steps can occur via cellular esterases, while only the first step can be performed by plasma esterases. The plasma esterases are unknown but likely differ from the cellular enzymes. The modified benzoic acid (Ar) substituents evaluated in this study were designed based on the hypothesis that this position serves as a critical site for manipulating plasma stability without impacting cellular bioactivation.

## Results

### Design and Synthesis of Prodrugs

The synthetic route to this new collection of diester prodrugs is outlined in Scheme 1. A variety of substituted benzoic acids (**4a–4k**) was converted to the corresponding chloromethyl esters (**5a–5k**) via a standard reaction with chloromethyl chlorosulfate.^29^ Reaction of the benzoate chloromethyl esters with the mixed aryl methyl ester **6** results in the selective replacement of the methyl ester with the acyloxy group to form **7a–7k**. Alternatively, the methyl ester could be hydrolyzed selectively to the corresponding monoacid at or below room temperature and then allowed to react with the chloromethyl ester. This latter method allows transformations at room temperature, which is advantageous for heat-sensitive substrates. In either sequence, the *p*-isopropylphenyl group was held constant throughout this series because a *p*-isopropylphenyl ester had proven to have attractive plasma stability in earlier studies,^15^ and with this group constant any significant differences in biological activity could be attributed to modification of the acyloxy ester. Oxidation with selenium dioxide gave allylic alcohols **8a–8k** with the desired *trans* olefin stereochemistry,^30^ and these compounds were readily converted to the corresponding acetate forms (**9a–9k**). All of the new compounds were evaluated in bioassays on both the alcohol and acetate forms when available.

**Scheme 1:**
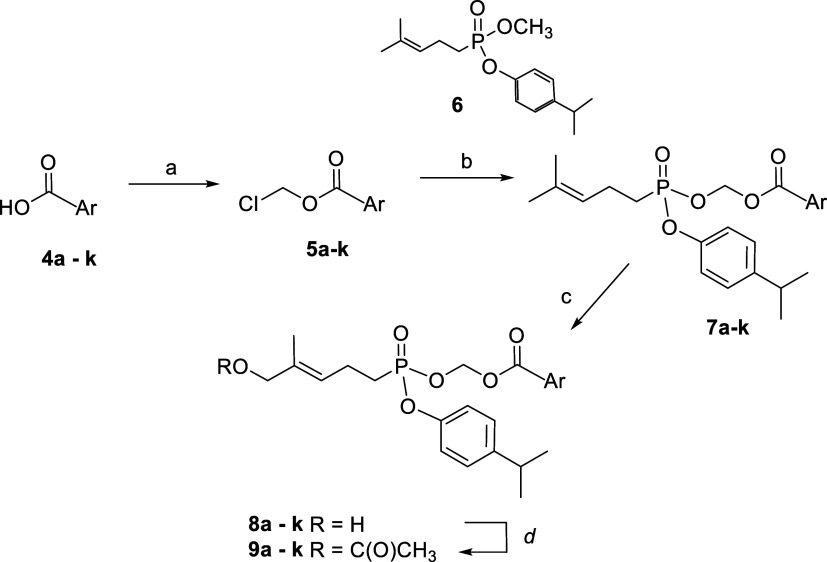
Synthesis of Mixed Aryl Acyloxy Ester Phosphonate Prodrugs^a^

a Reagents and conditions: (a) NaHCO_3_, Bu_4_NHSO_4_, chloromethyl chlorosulfate, CH_2_Cl_2_:H_2_O 1:1, rt, overnight, 89–90%; (b) NaI, CH_3_CN (anhyd), reflux, 2 days; or alternatively TMSBr, CH_2_Cl_2_, 0 °C to rt; then THF/H_2_O; Et_3_N, DMF, rt, 2 days, 8–62%; (c) SeO_2_, *t*-BuOOH, 4-hydroxybenzoic acid, CH_2_Cl_2_, 0 °C, 3 days, 6–24%; (d) Ac_2_O, Et_3_N, CH_2_Cl_2_, rt, overnight, 79–88%. The substituents a–k are defined in Table 1.

The target compounds (Table 1) were synthesized based on the following rationale. The first group of new prodrug forms placed a fluoro or trifluoromethyl group on the benzoic acid ring (**8a–d, 9a–d**) (first column). Initial testing suggested that in the *ortho* position, the trifluoromethyl group was superior to the fluoro modification. This led to additional synthesis of analogs containing the trifluoromethyl group at the *meta* and *para* positions. Then, to explore the impact of other electron-withdrawing groups, at least as reflected by Hammett constants,^31^ in the second group, three different electron-withdrawing substituents were placed in the *para* position (**8e–g, 9e,f**) (second column). Finally, in the third group, several disubstituted compounds were prepared, again including at least one electron-withdrawing group (**8h–k, 9h–k**) (third column). In ten of the eleven cases, the prodrug was prepared with both a free allylic alcohol and with that alcohol masked as an acetate derivative. In the single case of the *p*-nitro substituent, the limited availability of alcohol **8g** precluded preparation of the acetate. Our previous work had shown that acetylation only modestly increases the potency of the prodrug in some cases,^15^ so a larger-scale preparation of the acetate derivative of compound **8g** was postponed. Thus, a total of twenty-one new aryl acyloxy phosphonester prodrug forms was available for bioassays (Table 1). The new compounds ranged in mass from 450.44 to 610.48 Da, while their calculated partition coefficients (cLogP) ranged from 4.73 to 7.43 (Table 2). These cLogP values are slightly lipophilic compared to most drugs, but are in the normal range for prodrug forms, and low enough to allow for aqueous solubility at the concentrations evaluated. These new compounds were examined in proliferation, interferon γ secretion, and plasma stability bioassays as described in the following paragraphs.

**Table 1:**
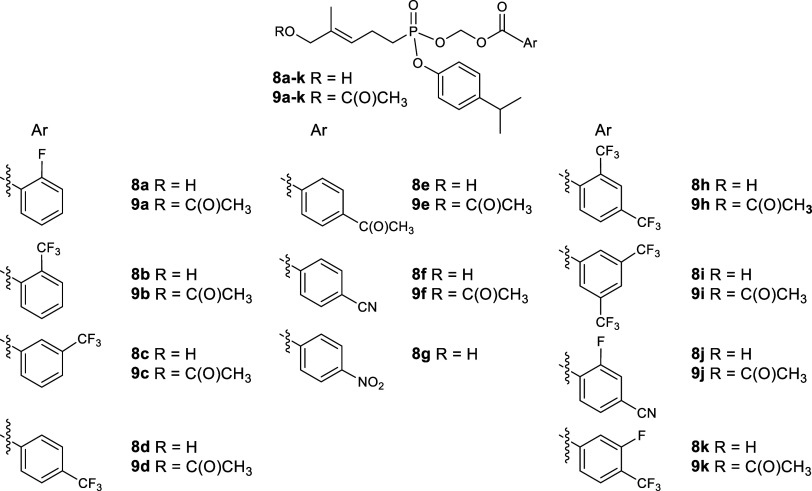
Twenty-one New Diester Prodrug Forms

**Table 2:** Compound Characteristics^a^

	alcohol				acetate		
	structure	M.W.	cLogP		structure	M.W.	cLogP
**3b**	amidate^14^	341.43	2.05				
**3d**	H^15,27^	432.45	5.26		H^15,27^	474.49	5.67
**3e**	4-MeO^27^	462.48	5.06		4-MeO^27^	504.51	5.47
**8a**	2-F	450.44	5.40	**9a**	2-F	492.47	5.82
**8b**	2-CF_3_	500.44	6.13	**9b**	2-CF_3_	542.48	6.55
**8c**	3-CF_3_	500.44	6.13	**9c**	3-CF_3_	542.48	6.55
**8d**	4-CF_3_	500.44	6.13	**9d**	4-CF_3_	542.48	6.55
**8e**	4-acetyl	474.48	4.73	**9e**	4-acetyl	516.52	5.14
**8f**	4-CN	457.46	5.11	**9f**	4-CN	499.49	5.52
**8g**	4-nitro	477.44	5.21				
**8h**	2,4-CF_3_	568.44	7.01	**9h**	2,4-CF_3_	610.48	7.43
**8i**	3,5-CF_3_	568.44	7.01	**9i**	3,5-CF_3_	610.48	7.43
**8j**	2-F, 4-CN	475.45	5.26	**9j**	2-F, 4-CN	517.48	5.67
**8k**	3-F, 4-CF_3_	518.43	6.28	**9k**	3-F, 4-CF_3_	560.47	6.69

a Structure column refers to Ar = −C_6_H_4_X (**3e, 8a–g, 9a–f**), Ar = −C_6_H_3_X_2_ (**8/9h,i**), or Ar = −C_6_H_3_XY (**8/9j**–**k**). Numerical position is derived from the parent benzoic acid **4**. Parameters determined using Marvin Sketch.

### Evaluation of Phosphoantigen Activity

The compounds were assessed using two functional assays to evaluate phosphoantigen activity. T cell activation by phosphoantigens elicits several functional outcomes, including proliferation and cytokine production (Figure 1). Proliferation was monitored via flow cytometry, measuring the proportion of cells expressing CD3 (a pan–T cell marker) and the γδ T cell receptor (a specific marker for γ9δ2 T cells). An increased percentage of these markers indicates phosphoantigen-induced proliferation. Cytokine production was evaluated by ELISA, quantifying secreted interferon γ, a prototypical cytokine generated by phosphoantigen-activated γ9δ2 T cells. The most potent phosphoantigen prodrugs typically demonstrate subnanomolar EC_50_ values in these assays (Figure 2). The ELISA protocol utilizes a brief, one-hour exposure period (within the linear range of prodrug uptake), enabling the detection of potency differences due to faster or slower intracellular payload delivery.^28^

First, proliferation of γ9δ2 T cells stimulated by the test compounds was assessed (Table 3, Supporting Information Figure S1). In this assay,^15^ human peripheral blood mononuclear cells (containing γ9δ2 T cells) were incubated with various doses of the test compounds for 3 days, washed, and then incubated for an additional 11 days. The percentage of γ9δ2 T cells at day 14 was determined by flow cytometry with staining for the γδ T cell receptor. The novel compounds were all active in this assay, with potencies ranging from 0.22 nM (compound **9d**) to 7.7 nM (**8h**), a 35-fold range. This was viewed as promising, in the range of our most potent phosphonamidate (0.28 nM) and phosphonester (0.27 nM) forms, and below our target of 1 nM.^27,32^ Five prodrug scaffolds (**a**, **c**, **d**, **f**, and **j**) were more potent than compound **3e** in both the alcohol (**8**) and acetate (**9**) forms. While the trifluoromethyl-substituted compounds **8/9c** and **8/9d** displayed excellent potency, addition of a second trifluoromethyl group was counterproductive, producing some of the weaker compounds (**8/9h** and **8/9i**).

**Table 3:** 14-Day Proliferation γ9δ2 T Cells^a^

	alcohol			acetate	
	structure	EC_50_ (nM)		structure	EC_50_ (nM)
**3b**	amidate^14^	0.36			
**3d**	H^15,27^	0.43		H^15,27^	0.27
**3e**	4-MeO^27^	1.5		4-MeO^27^	0.84
**8a**	2-F	0.75 (0.21 to 2.7)	**9a**	2-F	0.63 (0.19 to 2.1)
**8b**	2-CF_3_	4.0 (2.9 to 5.6)	**9b**	2-CF_3_	2.8 (1.2 to 6.5)
**8c**	3-CF_3_	1.0 (0.60 to 1.8)	**9c**	3-CF_3_	0.57 (0.17 to 1.9)
**8d**	4-CF_3_	0.86 (0.24 to 3.1)	**9d**	4-CF_3_	0.22 (0.022 to 2.2)
**8e**	4-acetyl	2.6 (0.43 to 15)	**9e**	4-acetyl	1.3 (0.22 to 7.4)
**8f**	4-CN	1.2 (0.42 to 3.3)	**9f**	4-CN	0.74 (0.18 to 3.1)
**8g**	4-nitro	0.58 (0.061 to 5.5)			
**8h**	2,4-CF_3_	7.7 (2.5 to 24)	**9h**	2,4-CF_3_	6.3 (2.6 to 15s)
**8i**	3,5-CF_3_	7.3 (0.11 to 480)	**9i**	3,5-CF_3_	2.1 (0.49 to 8.8)
**8j**	2-F, 4-CN	1.0 (0.035 to 29)	**9j**	2-F, 4-CN	0.32 (0.062 to 1.7)
**8k**	3-F, 4-CF_3_	3.9 (0.80 to 19)	**9k**	3-F, 4-CF_3_	1.1 (0.063 to 20)

a Values represent mean EC_50_ with 95% CI after 3-day loading and 11-day culture (*n* = 3).

Next, interferon γ release from the γ9δ2 T cells stimulated by the test compounds was assessed (Table 4, Supporting Information Figure S2). In this assay, K562 leukemia cells were incubated with various doses of the test compounds for 1 h, washed, and then cocultured with purified effector γ9δ2 T cells for 20 h. The amount of interferon γ released by the γ9δ2 T cells was determined by ELISA.^33^ Again, the novel compounds were all active in this assay, with potencies ranging from 0.80 nM (**8k**) to 31 nM (**8i**), a 39-fold range. Eight prodrug scaffolds were more potent than compound **3e** in both the alcohol and acetate forms. The only two that were weaker were those with a second trifluoromethyl group (**8/9h** and **8/9i)**, consistent with the proliferation testing. As with our prior studies,^15,27^ the average potency in the ELISA is slightly weaker than in the proliferation assay, likely as a result of the much shorter 1-h incubation period. The SAR trends of the two functional assays were reasonably correlated, with a Pearson coefficient of 0.65.

**Table 4:** ELISA for Interferon γ Secretion by Activated γ9δ2 T Cells^a^

	alcohol			acetate	
	structure	EC_50_ (nM)		structure	EC_50_ (nM)
**3b**	amidate^28^	230			
**3d**	H^15,27^	7.0		H^15,27^	2.6
**3e**	4-MeO^27^	6.4		4-MeO^27^	4.1
**8a**	2-F	1.0 (0.44 to 3.3)	**9a**	2-F	1.7 (0.71 to 6.2)
**8b**	2-CF_3_	3.9 (2.6 to 5.7)	**9b**	2-CF_3_	3.7 (2.7 to 4.9)
**8c**	3-CF_3_	0.84 (0.46 to 1.7)	**9c**	3-CF_3_	1.9 (1.4 to 2.6)
**8d**	4-CF_3_	2.3 (1.4 to 4.0)	**9d**	4-CF_3_	1.2 (0.87 to 1.7)
**8e**	4-acetyl	1.4 (0.40 to 13)	**9e**	4-acetyl	0.83 (0.62 to 1.1)
**8f**	4-CN	1.2 (0.53 to 3.4)	**9f**	4-CN	1.5 (0.91 to 2.6)
**8g**	4-nitro	3.2 (1.6 to 7.7)			
**8h**	2,4-CF_3_	9.3 (5.2 to 21)	**9h**	2,4-CF_3_	19 (10 to 51)
**8i**	3,5-CF_3_	31 (20 to 54)	**9i**	3,5-CF_3_	26 (16 to 50)
**8j**	2-F, 4-CN	1.6 (0.75 to 4.0)	**9j**	2-F, 4-CN	1.9 (1.4 to 2.8)
**8k**	3-F, 4-CF_3_	0.80 (0.31 to 3.4)	**9k**	3-F, 4-CF_3_	3.1 (2.0 to 5.6)

a Values represent mean EC_50_ with 95% CI after 1 h loading and 20 h coculture (*n* = 3).

### Prodrug Stability in Human Plasma

The compounds were also evaluated for plasma stability (Table 5, Supporting Information Figures S3 and S4). In this assay, the test compounds were incubated for various time points in 50% pooled human plasma in tris-buffered saline. The amount of compound remaining at the indicated time points was determined by LC-MS. Stabilities ranged from a 0.3 h half-life (compound **8j**) to 39 h (**8b**), a 130-fold range. At the 2 h time point, most of these prodrug scaffolds were more stable in human plasma than compound **3d**, while several of the alcohol forms were more stable than compound **3e**. The acetate forms were generally less stable than the alcohol forms, likely due to enzymatic conversion of the acetate to the alcohol form.

**Table 5:** Human Plasma Stability^a^

	alcohol					acetate			
	structure	2 h (%)	24 h (%)	*t*_1/2_ (h)		structure	2 h (%)	24 h (%)	*t*_1/2_ (h)
**3b**	amidate^14^	100	96	>24					
**3d**	H^15,27^	13		0.73		H^15,27^	34		1.1
**3e**	4-MeO^27^	71		8.3		4-MeO^27^	62		3
**8a**	2-F	0.75 ± 0.44	0	0.41	**9a**	2-F	4.7 ± 1.6	0	0.51
**8b**	2-CF_3_	91 ± 6.4	76 ± 14	39	**9b**	2-CF_3_	89 ± 4.7	67 ± 12	33
**8c**	3-CF_3_	86 ± 11	28 ± 13	8.9	**9c**	3-CF_3_	66 ± 8.7	1.2 ± 1.4	3.3
**8d**	4-CF_3_	80 ± 5.3	56 ± 8.1	26	**9d**	4-CF_3_	81 ± 9.4	24 ± 17	7.2
**8e**	4-acetyl	63 ± 3.9	9.6 ± 3.3	4.7	**9e**	4-acetyl	48 ± 6.0	4.3 ± 2.8	2.0
**8f**	4-CN	9.1 ± 7.1	0	0.74	**9f**	4-CN	17 ± 10	0	0.96
**8g**	4-nitro	37 ± 16	0.46 ± 0.92	1.1					
**8h**	2,4-CF_3_	81 ± 27	49 ± 11	29	**9h**	2,4-CF_3_	89 ± 15	44 ± 6.7	20
**8i**	3,5-CF_3_	81 ± 14	14 ± 16	5.0	**9i**	3,5-CF_3_	93 ± 19	18 ± 22	4.3
**8j**	2-F, 4-CN	4.1 ± 3.3	0.10 ± 0.15	0.30	**9j**	2-F, 4-CN	17 ± 12	0.23 ± 0.32	0.42
**8k**	3-F, 4-CF_3_	79 ± 18	0.65 ± 1.1	4.7	**9k**	3-F, 4-CF_3_	45 ± 2.6	0.19 ± 0.29	1.7

a Values represent mean percentage (±stdev) remaining after 2 or 24 h in plasma (*n* = 4). Half lives were determined in time course experiments (*n* = 2).

### Prodrug Uptake and Cellular Metabolism

We next selected a subset of compounds to evaluate for cellular uptake and payload release (Figure 4). To avoid interference by the allylic acetate forms, we focused on some of the most potent allylic alcohol forms (**8c**–**e**), the least potent (**8h**, **8i**), and an inactive negative control (compound **10**, (*E*)-(((2,6-diisopropylphenoxy)­(5-hydroxy-4-methylpent-3-en-1-yl)­phosphoryl)­oxy)­methyl pivalate).^15^ In this assay, the test compounds were incubated with K562 cells for 60 min, and the intracellular levels of aryl monoanion and desired payload were determined by LC-MS. Because all of the compound **8** variants release the same aryl monoanion (Figure 3), their relative amounts can be determined by comparing the integrated peak intensities following treatment with each compound (Figure 4A). Similarly, the compound **8** variants and compound **10** negative control would all release the same dianionic payload (Figure 4B). Treatment with compound **8d** led to the highest cellular levels of both the aryl monoanion and the desired payload. The less active compounds **8h** and **8i** clearly delivered lower levels. Negative control compound **10** released only negligible levels of the desired payload (Figure 4B).

**Figure 4:**
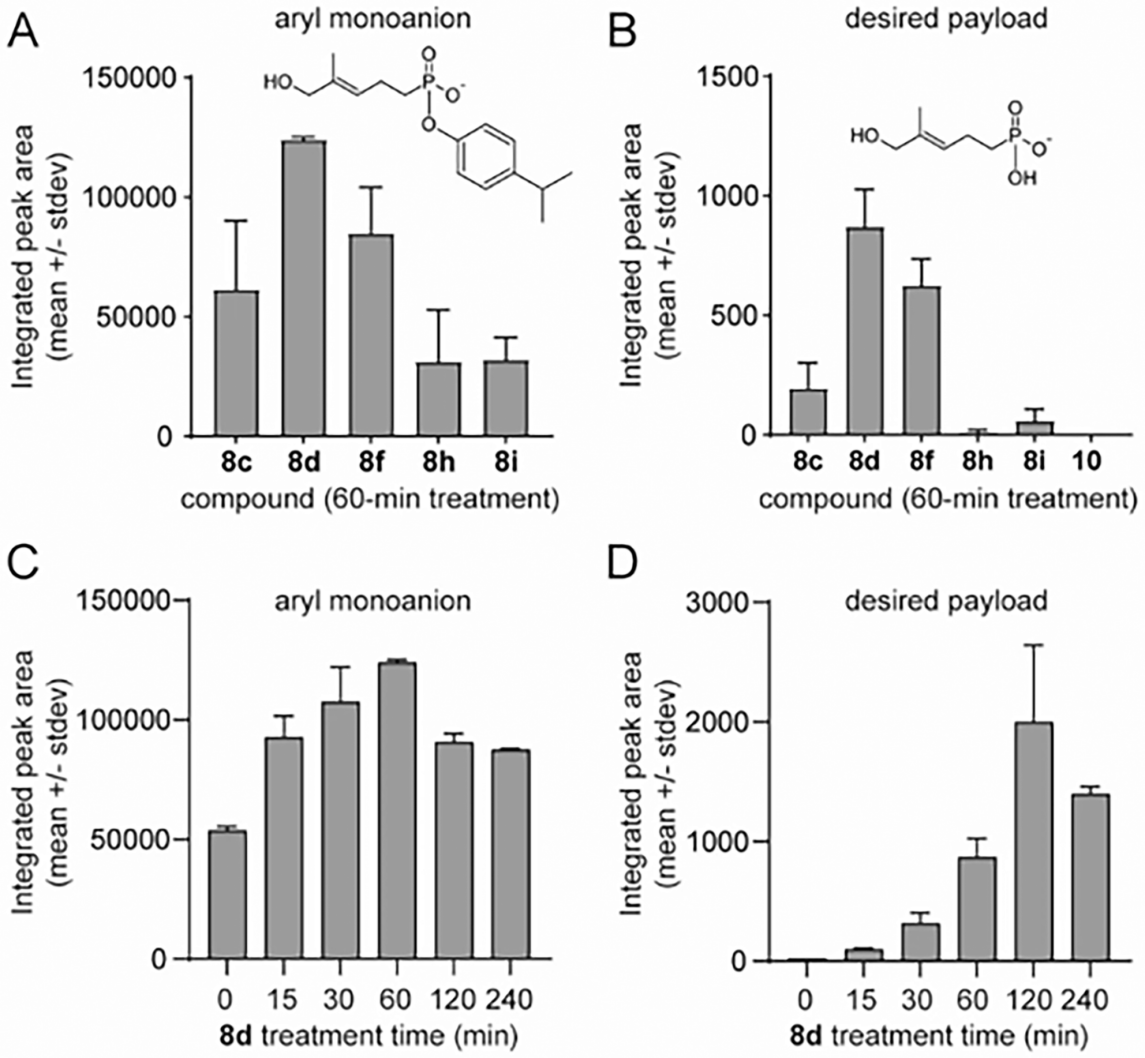
Cellular uptake and release of selected prodrug forms. Relative intracellular amounts of (A) the aryl monoanion and (B) the desired payload after treatment of K562 cells with 100 μM of the indicated compounds for 60 min. (C, D) Aryl monoanion and desired payload after treatment of K562 cells with 100 μM **8d** for indicated times. Values represent mean (±stdev) integrated peak intensities (*n* = 3).

Compound **8d** was then evaluated for release in a cellular time course experiment. The cellular levels of aryl monoanion peaked at 60 min of treatment time (Figure 4C). A significant amount of the aryl monoanion was observed even at 0 min, suggesting that some internalization and metabolism of the compound to the aryl monoanion occurs faster than the samples can be processed. However, levels of the desired payload (Figure 4D) were negligible at 0 min but peaked after 120 min of treatment. A decline was observed after 240 min, suggesting that the cells are either exporting or metabolizing the desired payload. Taken together, these prodrug forms undergo rapid internalization and conversion to the aryl monoanion, followed by slower conversion of the aryl monoanion to the desired payload.

### Prodrug Cytotoxicity

The compounds were also evaluated for cytotoxicity (Supporting Information Figure S5). After 72-h treatment with a concentration of 10 μM, no significant impact on cell viability was observed in any of the new compounds. This is notable because the compounds are active as phosphoantigens at significantly lower doses and exposure times.

### Emergence of **8d** as a Lead Analog

The compounds were evaluated based on their activity in the proliferation and ELISA assays and their stability in the plasma stability assay. From this ranking, it was determined that compounds **8/9d** exhibited the best blend of characteristics. Compound **8d** dose dependently increased the percentages of γ9δ2 T cells (Figure 5A–C) with an EC_50_ of 0.86, among the most potent of the group. The compound increased the proliferation of the T cells, causing a 7.5-fold increase in cell numbers versus nonstimulated cells (Figure 5B). The ELISA activity was also above average for the group, with an EC_50_ of 2.3 nM (Figure 5D). Compound **8d** was also among the most stable of the group, with a half-life of 26 h (Figure 5E). Due to this blend of potency and plasma stability, compound **8d** was determined to be the most promising allylic alcohol in the group. The allylic acetate **9d** displayed a similar pattern of features to **8d**, confirming the SAR trend. However, the stability of **8d** was superior to **9d** so it was judged as the better lead.

**Figure 5:**
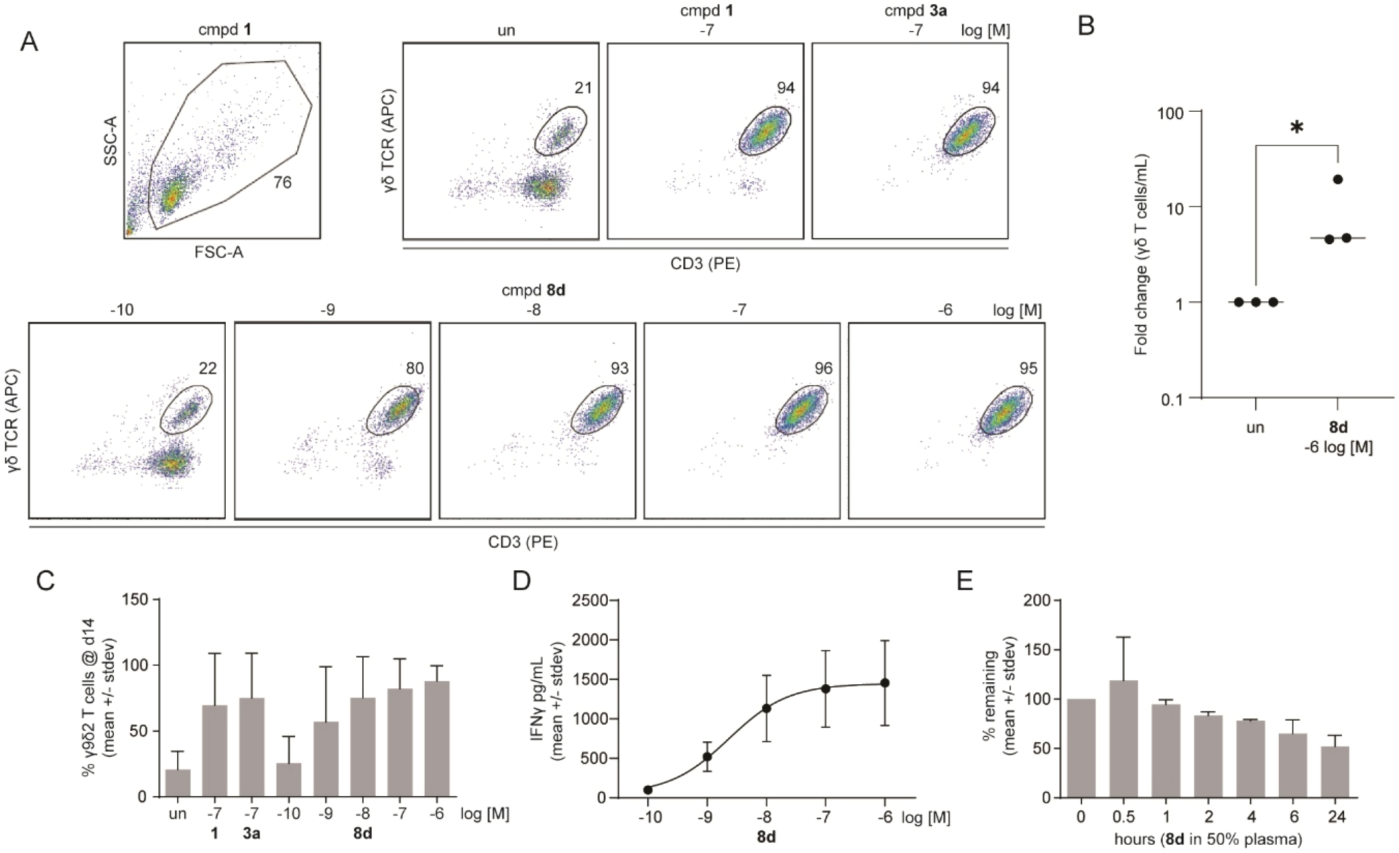
Compound **8d** demonstrates potent phosphoantigen activity with high plasma stability. (A) Proliferation assessment by flow cytometry. Human peripheral blood mononuclear cells were treated with test compounds, including **8d** for 3 days, then grown for an additional 11 days. The lymphocytes were gated on forward and side scatter, then the γδ T cells quantified by anti-CD3 and anti-TCRγ/δ. The phosphoantigen-responsive population was gated. Data is representative of *n* = 3 independent experiments. (B) Fold increase in abundance of γδ T cells following stimulation with 1 μM **8d**. (C) Quantification of proliferation data for compound **8d**. (D) Quantification of interferon γ secreted in response to K562 cells loaded with **8d** as determined by ELISA. Cells were loaded for 1 h and cocultured with γδ T cells for 20 h. (E) Plasma stability of **8d** at various time points determined by LC-MS.

## Discussion

Historically, achieving satisfactory plasma stability with ester-based prodrugs has been considered challenging due to the activity of plasma esterases. In this study, we hypothesized that incorporating modified benzoic acid substructures may enhance plasma stability while maintaining high cellular potency. To test that hypothesis, we synthesized 21 novel prodrug analogs of HMBPP, evaluated them in two complementary functional assays, and determined their plasma stability. The synthesis proved to be efficient, enabling the reaction of various chloromethyl esters with phosphonomethyl esters or acids to yield the targeted phosphonate prodrug via two distinct methods. Notably, one approach facilitates the reaction at room temperature, which would be required for heat-labile substituents. The potency of the compounds was excellent, showing a 35-fold range in the proliferation assay and a 39-fold range in the ELISA with reasonable correlation, with most compounds active in the sub- or low- nanomolar range. Notably, plasma stability exhibited considerable variability, spanning a 130-fold range, with certain ester prodrugs demonstrating plasma stabilities exceeding 24 h. This substantial variability in plasma stability aligns with and supports our initial hypothesis.

To prioritize the compounds, we determined which ones contained an improved blend of both potency and plasma stability. Clearly, the compounds containing a single trifluoromethyl group were superior in this regard, with the *para-*trifluoromethyl-substituted compound **8d** being the best (Figure 5). Its excellent activity profile can be best understood by comparing it to compounds **3b** (an aryl amidate) and **3d** (an acyloxy aryl phosphonester prodrug derived from unsubstituted benzoic acid). Compound **8d** is appealing because, relative to compound **3b**, its potency in the 1-h ELISA experiment increased by 100-fold to 2.3 nM. Relative to compound **3d**, its plasma stability increased by 36-fold to 26 h.

On the other end of the spectrum were the less effective compounds. Compounds containing two trifluoromethyl groups (**8h/9h** and to a lesser extent **8i/9i**) did significantly improve plasma stability, but at the same time their potency was decreased. It was interesting that the 2-CF_3_ (**8b**), 4-CF_3_ (**8d**), and 2,4-bisCF_3_ (**8h**) analogs all had half-lives over 26 h, while the 3-CF_3_ (**8c**) and the 3,5-bisCF_3_ (**8i**) half-lives were in the 5–9 h range. Compounds containing 2-fluoro (**8a**) or 2-fluoro,4-cyano (**8j**) substituents had a similar activity and stability profile to the undecorated compound (**3d**), indicating there was no additive benefit to these two modifications.

The trifluoromethyl group contained in compound **8d** may influence its characteristics in a variety of ways, including electronic, steric, and hydrophobic effects. We suspect that steric and hydrophobic effects play only minor roles in this system, as larger and more hydrophobic analogs failed to achieve the plasma stability of compound **8d**. This group is strongly electron-withdrawing, yet other strongly electron-withdrawing modifications we tested (the cyano group in **8f**, and nitro substituent in **8g**) did not improve stability. We suspect the plasma stability of **8d**, and also **8b**, is due to inductive effects that are most pronounced when the substituent is placed at the *para* and *ortho* positions, while resonance effects of the other groups were counterproductive. Interestingly, the trifluoromethyl group increases stability in the plasma but does not eliminate cellular potency, suggesting that such analogs are poor substrates for plasma esterases but nevertheless viable substrates for intracellular esterases. While trifluoromethyl groups are often used in medicinal chemistry to improve stability, our study is unique in applying the trifluoromethyl group to an ester prodrug moiety to impart selective stability in plasma relative to cells.

Although it is conceivable that a neutral prodrug could bind to the BTN3A1 protein or that hydrolysis of the phosphonate ester to a charged species could occur extracellularly in response to secreted esterases (or ones naturally found in the culture media), both possibilities are unlikely. In our initial study in this area, we reported that the dimethyl ester of this phosphonate ligand is inactive^13^ which was expected given the resistance of phosphonate methyl esters to enzymatic hydrolysis.^34^ This indicated the importance of the charged ligand for BTN binding. In a more recent study, we established that the phenyl monoanion and the naphthyl monoanion of this phosphonate each have significantly reduced potency in the proliferation assay relative to their aryl acyloxy counterparts.^35^

The optimal use of phosphoantigens and their prodrugs to trigger and expand T cells in clinical settings is still uncertain. It is likely that the development of phosphoantigens and γδ T cell therapies will follow patterns similar to those seen with traditional T cell treatments. Options may include administering ex vivo phosphoantigen-expanded γδ T cells as adoptive cell therapy or using phosphoantigen prodrugs directly as therapeutic agents, either in a role like immune checkpoint modulators or as preventative treatments resembling therapeutic vaccines. In any scenario, potent and stable phosphoantigen prodrugs like the ones identified in this study could prove beneficial.

Our findings also have broader implications for those working on the development of phosphonate prodrugs. Ester-based nucleotide prodrugs such as adefovir dipivoxil and tenofovir disoproxil have achieved clinical success, but these are used primarily to increase oral bioavailability, delivering the free acid payload into the bloodstream. These prodrug forms lack sustained stability in the bloodstream, decreasing their ability to deliver the payload to peripheral tissue. It is possible that the trifluoromethyl benzoate modified ester-based prodrugs described herein, either through faster cellular uptake kinetics or through extended plasma stability, may achieve better distribution to peripheral tissue. This could benefit the intravenous or potentially even the oral route of administration.

This study has significant implications for the future development of phosphonate prodrugs as therapeutic agents. By demonstrating that benzoic acid derivatives, particularly those with single trifluoromethyl substitutions, can dramatically enhance plasma stability while maintaining robust cellular potency, this work provides a new strategy for optimizing drug-like properties in phosphonate prodrugs. The selective stabilization achieved through benzoic acid modification ultimately could be more broadly applicable to other ester-based prodrugs, opening new avenues for the design of next-generation therapeutics with improved plasma stability.

## Conclusions

In this study, we synthesized and characterized 21 novel prodrug derivatives of C-HMBP. We found that phosphonester prodrugs with single trifluoromethyl substitutions in the benzoic acid substructure, especially the *para-*trifluoromethyl analog **8d**, demonstrate a superior balance of cell potency and plasma stability. These insights could inform future development of phosphonester prodrugs by combining sustained plasma stability with robust cellular activity.

## Experimental

### Chemical Synthesis

#### General Experimental Procedures

Acetonitrile and dichloromethane were distilled from calcium hydride prior to use. Sodium iodide was dried in an oven, while triethylamine was dried over molecular sieve. All other reagents were purchased from commercial sources and used without further purification. All reactions in nonaqueous environments were conducted in flame-dried glassware under a positive pressure of nitrogen and with magnetic stirring. All NMR spectra were obtained at 400 or 500 MHz for ^1^H, 101 or 126 MHz for ^13^C, and 162 or 203 MHz for ^31^P with internal standards of (CH_3_)_4_Si (^1^H, 0.00) or CDCl_3_ (^1^H, 7.26; ^13^C, 77.2 ppm) for nonaqueous samples and external H_3_PO_4_.. The purity of each assayed compound was evaluated with an Agilent 1220 series HPLC (100% methanol, Column: Restek ultrasilica, 5 μm, C18, dimensions 250 × 4.6 mm (analytical), flow rate: 0.5 mL/min), and all assayed compounds had a purity >95%.

##### Chloromethyl-2-fluorobenzoate (**5a**)

To a stirring solution of 2-fluorobenzoic acid (1.52 g, 10.8 mmol) in dichloromethane (40 mL) was added water (40 mL), sodium bicarbonate (3.65 g, 43.4 mmol), and tetrabutylammonium hydrogen sulfate (0.37 g, 1.09 mmol). The reaction mixture was stirred at rt for one hour, chloromethyl chlorosulfate (1.43 mL, 14.1 mmol) was added dropwise at 0 °C, and the mixture was stirred overnight at rt. The reaction mixture was extracted with dichloromethane (3 × 30 mL) and the combined organic fractions were dried (Na_2_SO_4_) and concentrated *in vacuo* to obtain product **5a** as a colorless oil (1.97 g, 96%): ^1^H NMR (CDCl_3_, 500 MHz) δ 8.00–7.96 (td, *J* = 7.3, 1.4 Hz, 1H), 7.61–7.57 (m, 1H), 7.26–7.15 (m, 2H), 5.95 (s, 2H); ^13^C NMR (CDCl_3_, 126 MHz) δ 162.2 (d, *J*_CF_ = 263 Hz), 162.0 (d, *J*_CF_ = 3.9 Hz), 135.5 (d, *J*_CF_ = 9.2 Hz), 132.2 (2C), 124.0 (d, *J*_CF_ = 4.0 Hz), 117.1 (d, *J*_CF_ = 22.0 Hz), 69.0 ppm.

##### (((4-Isopropylphenoxy)­(4-methylpent-3-en-1-yl)­phosphoryl)­oxy)­methyl-2-fluorobenzoate (**7a**)

The chloromethyl ester **5a** (1.39 g, 7.35 mmol) was dissolved in acetonitrile (10 mL) and transferred to a round-bottom flask containing the phosphonate **6** (870 mg, 2.94 mmol) and sodium iodide (660 mg, 4.40 mmol). The mixture was stirred and heated at reflux for 2 days. The mixture was extracted with diethyl ether (3 × 30 mL), and the combined organic fractions were dried (Na_2_SO_4_) and concentrated *in vacuo*. Final purification via column chromatography (10–40% ethyl acetate in hexane) yielded the desired olefin **7a** as a yellow oil (572 mg, 45%): ^1^H NMR (CDCl_3_, 400 MHz) δ 7.92–7.88 (td, *J* = 7.5, 1.5 Hz, 1H), 7.58–7.53 (m, 1H), 7.20–7.14 (m, 2H), 7.11 (s, 4H), 5.97–5.93 (dd, *J*_PH_ = 13.9 Hz, *J*_HH_ = 5.2 Hz, 1H), 5.90–5.86 (dd, *J*_PH_ = 12.9 Hz, *J*_HH_ = 5.2 Hz, 1H), 5.12–5.09 (td, *J* = 7.1, 1.4 Hz, 1H), 2.85–2.80 (m, 1H), 2.42–2.35 (m, 2H), 2.05–1.98 (m, 2H), 1.66 (s, 3H), 1.57 (s, 3H), 1.19 (d, *J* = 2.2 Hz, 3H), 1.18 (d, *J* = 2.2 Hz, 3H); ^13^C NMR (CDCl_3_, 126 MHz) δ 162.5 (d, *J*_CF_ = 3.9 Hz), 162.4 (d, *J*_CF_ = 263 Hz), 147.8 (d, *J*_PC_ = 9.4 Hz), 145.7 (d, *J*_PC_ = 1.4 Hz), 135.4 (d, *J*_CF_ = 9.2 Hz), 133.3 (d, *J*_PC_ = 1.8 Hz), 132.4 (2C), 127.6 (2C), 124.1 (d, *J*_CF_ = 4.0 Hz), 122.5 (d, *J*_PC_ = 17.5 Hz), 120.3 (d, *J*_PC_ = 4.2 Hz, 2C), 117.1 (d, *J*_CF_ = 22.1 Hz), 82.2 (d, *J*_PC_ = 6.3 Hz), 33.4, 26.4 (d, *J*_PC_ = 137.9 Hz), 25.6, 24.0 (2C), 20.8 (d, *J*_PC_ = 4.8 Hz), 17.6; ^31^P NMR (CDCl_3_, 203 MHz) δ 29.8 ppm.

##### (*E*)-(((5-Hydroxy-4-methylpent-3-en-1-yl)­(4-isopropylphenoxy)­phosphoryl)­oxy)­methyl-2-fluorobenzoate (**8a**)

The olefin **7a** (530 mg, 1.22 mmol) was added to a suspension of selenium dioxide (108 mg, 0.97 mmol) and 4-hydroxybenzoic acid (24 mg, 0.17 mmol) in dichloromethane (10 mL). At 0 °C, *tert*-butyl hydroperoxide (70 wt % in H_2_O, 674 μL, 4.89 mmol) was slowly added, and the reaction mixture was stirred at 0 °C for 3 days. The reaction was quenched by the addition of sodium bicarbonate and extracted with dichloromethane (3 × 10 mL). The combined organic fractions were dried (Na_2_SO_4_) and concentrated *in vacuo*. Final purification via column chromatography (100% ether to 3% methanol in ether) afforded the desired alcohol **8a** as an oil (75.8 mg, 14%): ^1^H NMR (CDCl_3_, 500 MHz) δ 7.91–7.88 (td, *J* = 7.3, 1.3 Hz, 1H), 7.58–7.54 (m, 1H), 7.21–7.14 (m, 2H), 7.11 (s, 4H), 5.96–5.92 (dd, *J*_PH_ = 13.7 Hz, *J*_HH_ = 5.2 Hz, 1H), 5.88–5.85 (dd, *J*_PH_ = 12.9 Hz, *J*_HH_ = 5.2 Hz, 1H), 5.43–5.40 (td, *J* = 7.2, 1.4 Hz, 1H), 3.95 (s, 2H), 2.85–2.80 (m, 1H) 2.48–2.41 (m, 2H), 2.09–2.02 (m, 2H), 1.61 (s, 3H), 1.19 (d, *J* = 2.3 Hz, 3H), 1.18 (d, *J* = 2.3 Hz, 3H); ^13^C NMR (CDCl_3_, 126 MHz) δ 162.6 (d, *J*_CF_ = 3.8 Hz), 162.3 (d, *J*_CF_ = 263 Hz), 147.7 (d, *J*_PC_ = 9.4 Hz), 145.8 (d, *J*_PC_ = 1.4 Hz), 136.7 (d, *J*_PC_ = 1.3 Hz), 135.5 (d, *J*_CF_ = 9.2 Hz), 132.4 (2C), 127.6 (2C), 124.1 (d, *J*_CF_ = 4.0 Hz), 123.0 (d, *J*_PC_ = 15.8 Hz), 120.3 (d, *J*_PC_ = 4.2 Hz, 2C), 117.1 (d, *J*_CF_ = 22.1 Hz), 82.1 (d, *J*_PC_ = 6.3 Hz), 68.1, 33.4, 26.1 (d, *J*_PC_ = 139.1 Hz), 24.0 (2C), 20.5 (d, *J*_PC_ = 5.0 Hz), 13.6; ^31^P NMR (CDCl_3_, 203 MHz) δ 29.8 ppm. HRMS (ESI^+^) *m*/*z*: calcd for C_23_H_29_FO_6_P (M + H)^+^, 451.1686; found 451.1680. HPLC purity 100% (*t*_R_ = 6.69).

##### (*E*)-(((5-Acetoxy-4-methylpent-3-en-1-yl)­(4-isopropylphenoxy)­phosphoryl)­oxy)­methyl-2-fluorobenzoate (**9a**)

Alcohol **8a** (33.7 mg, 0.07 mmol), acetic anhydride (11 μL, 0.11 mmol), and triethylamine (21 μL, 0.15 mmol) were dissolved in dichloromethane (2 mL), and the resulting reaction mixture was stirred overnight at rt. The reaction was quenched by the addition of sodium bicarbonate and extracted with dichloromethane (3 × 10 mL). The combined extracts were dried (Na_2_SO_4_) and concentrated *in vacuo*. Purification via column chromatography (100% ether to 1% methanol in ether) gave the desired product **9a** as an oil (30.7 mg, 83%): ^1^H NMR (CDCl_3_, 500 MHz) δ 7.91–7.88 (td, *J* = 7.3, 1.1 Hz, 1H), 7.58–7.54 (m, 1H), 7.21–7.13 (m, 2H), 7.11 (s, 4H), 5.97–5.93 (dd, *J*_PH_ = 13.8 Hz, *J*_HH_ = 5.2 Hz, 1H), 5.89–5.85 (dd, *J*_PH_ = 12.9 Hz, *J*_HH_ = 5.2 Hz, 1H), 5.47–5.44 (td, *J* = 7.1, 1.4 Hz, 1H), 4.42 (s, 2H), 2.86–2.80 (m, 1H), 2.50–2.42 (m, 2H), 2.09–2.02 (m, 2H), 2.05 (s, 3H), 1.63 (s, 3H), 1.20–1.19 (d, *J* = 2.1 Hz, 3H), 1.18 (d, *J* = 2.1 Hz, 3H); ^13^C NMR (CDCl_3_, 126 MHz) δ 170.8, 162.5 (d, *J*_CF_ = 3.8 Hz), 162.3 (d, *J*_CF_ = 263 Hz), 147.7 (d, *J*_PC_ = 9.4 Hz), 145.8 (d, *J*_PC_ = 1.4 Hz), 135.4 (d, *J*_CF_ = 9.2 Hz), 132.4 (2C), 131.8 (d, *J*_PC_ = 1.8 Hz), 127.6 (2C), 126.9 (d, *J*_PC_ = 17.4 Hz), 124.1 (d, *J*_CF_ = 4.0 Hz), 120.3 (d, *J*_PC_ = 4.2 Hz, 2C), 117.1 (d, *J*_CF_ = 22.1 Hz), 82.2 (d, *J*_PC_ = 6.2 Hz), 69.5, 33.4, 25.9 (d, *J*_PC_ = 139.6 Hz), 24.0 (2C), 20.9, 20.6 (d, *J*_PC_ = 4.8 Hz), 13.9; ^31^P NMR (CDCl_3_, 203 MHz) δ 29.4 ppm. HRMS (ESI^+^) *m*/*z*: calcd for C_25_H_31_FO_7_P (M + H)^+^, 493.1791; found 493.1779. HPLC purity 100% (*t*_R_ = 6.69).

##### Chloromethyl-2-(trifluoromethyl)­benzoate (**5b**)

To a stirring solution of 2-trifluoromethylbenzoic acid (2.0 g, 10.5 mmol) in dichloromethane (40 mL) was added water (40 mL), sodium bicarbonate (3.53 g, 42.0 mmol), and tetrabutylammonium hydrogen sulfate (350 mg, 1.0 mmol). After the reaction mixture was stirred at rt for one hour, chloromethyl chlorosulfate (2.2 g, 13.6 mmol) was added dropwise at 0 °C, and the mixture was stirred overnight at rt. The reaction mixture was extracted with dichloromethane (3 × 30 mL) and the combined organic fractions were dried (Na_2_SO_4_) and concentrated *in vacuo* to obtain product **5b** as a colorless oil (2.3 g, 92%): ^1^H NMR (400 MHz, CDCl_3_) δ 7.87–7.76 (m, 2H), 7.66–7.63 (m, 2H), 5.91 (s, 2H); ^13^C NMR (101 MHz, CDCl_3_) δ 164.4, 132.3, 131.9, 130.8 (2C), 130.6 (q, *J*_CF_ = 32.9 Hz), 127.1 (q, *J*_CF_ = 3.7 Hz), 123.4 (q, *J*_CF_ = 274.4 Hz), 69.5.

##### (((4-Isopropylphenoxy)­(4-methylpent-3-en-1-yl)­phosphoryl)­oxy)­methyl-2-(trifluoromethyl)­benzoate­(**7b**)

The mixed aryl methyl ester **6** (1.1 g, 3.7 mmol) was dissolved in freshly distilled dichloromethane (15 mL) and cooled to 0 °C in an ice bath. Trimethylsilyl bromide (1.42 g, 9.3 mmol) was added dropwise, and the resulting reaction mixture was stirred overnight at room rt. All the solvents were removed *in vacuo*, the resulting oil was dissolved in tetrahydrofuran and water (1:10 ratio) and stirred for 1 h at rt. After all volatiles were removed *in vacuo*, the residue was coevaporated three times with toluene to remove all traces of water. The resulting material was dried overnight at high vacuum. The phosphonic acid (1.0 g, 3.5 mmol) was dissolved in dimethylformamide (10 mL) followed by the addition of triethylamine (1.0 g, 9.9 mmol) and chloromethyl ester **5b** (2.1 g, 8.8 mmol). The resulting reaction mixture was stirred at rt for 2 days. The mixture was extracted with diethyl ether, and the combined extracts were washed with brine. The organic portions were combined, dried (Na_2_SO_4_), and filtered through celite, and the filtrate was concentrated *in vacuo*. The resulting oil was purified *via* flash chromatography (silica, 100% hexanes to 30% EtOAc in hexanes) to give the desired product **7b** as an oil (500 mg, 28%): ^1^H NMR (400 MHz, CDCl_3_) δ 7.78–7.75 (m, 2H), 7.64–7.55 (m, 2H), 7.10 (s, 4H), 5.93 (dd, *J*_PH_ = 13.6 Hz, *J*_HH_ = 5.2 Hz, 1H), 5.85 (dd, *J*_PH_ = 12.4 Hz, *J*_HH_ = 5.2 Hz, 1H), 5.10 (td, *J* = 7.2 Hz, 1.4 Hz, 1H), 2.87–2.77 (m, 1H), 2.43–2.33 (m, 2H), 2.03–1.92 (m, 2H), 1.65 (s, 3H), 1.56 (s, 3H), 1.17 (d, *J* = 6.9 Hz, 6H); ^13^C NMR (101 MHz, CDCl_3_) δ 164.9, 148.2 (d, *J*_PC_ = 9.3 Hz), 146.0, 133.7, 132.4, 132.2, 131.2 (2C), 129.5 (q, *J*_CF_ = 32.8 Hz), 128.0 (2C), 127.3 (q, *J*_CF_ = 5.5 Hz), 123.7 (q, *J*_CF_ = 274.4 Hz), 122.8 (d, *J*_PC_ = 17.7 Hz), 120.7 (d, *J*_PC_ = 4.2 Hz, 2C), 82.8 (d, *J*_PC_ = 6.2 Hz), 33.8, 26.6 (d, *J*_PC_ = 138.1 Hz), 25.9, 24.4 (2C), 21.2 (d, *J*_PC_ = 4.8 Hz), 18.0; ^31^P NMR (162 MHz, CDCl_3_) 29.7 ppm.

##### (*E*)-(((5-Hydroxy-4-methylpent-3-en-1-yl)­(4-isopropylphenoxy)­phosphoryl)­oxy)­methyl-2-(trifluoromethyl)­benzoate (**8b**)

The olefin **7b** (500 mg, 1.0 mmol) was added to a solution of selenium dioxide (92 mg, 0.829 mmol) and 4-hydroxybenzoic acid (20.0 mg, 0.14 mmol) in dichloromethane (6 mL). At 0 °C, *tert*-butyl hydroperoxide (70 wt % in H_2_O, 0.570 mL, 4.12 mmol) was added slowly to the stirred reaction mixture. The resulting reaction was stirred at 0 °C and allowed to react for 3 days. The reaction mixture was diluted by the addition of water (4 mL), quenched with Na_2_SO_3_ (3 mL), and extracted with dichloromethane (3 × 5 mL). The combined extracts were dried (Na_2_SO_4_), filtered through celite, and the filtrate was concentrated *in vacuo*. The resulting oil was purified by column chromatography (silica, 100% ether–3% MeOH in ether) to give the desired product **8b** as an oil in 12% yield (60 mg): ^1^H NMR (400 MHz, CDCl_3_) δ 7.79–7.76 (m, 2H), 7.66–7.57 (m, 2H), 7.11 (s, 4H), 5.93 (dd, *J*_PH_ = 13.6 Hz, *J*_HH_ = 5.2 Hz, 1H), 5.84 (dd, *J*_PH_ = 12.4 Hz, *J*_HH_ = 5.2 Hz, 1H), 5.42 (td, *J* = 7.2 Hz, 1.4 Hz, 1H), 3.96 (s, 2H), 2.88–2.80 (m, 1H), 2.49–2.40 (m, 2H), 2.08–2.00 (m, 2H), 1.62 (s, 3H), 1.18 (d, *J* = 6.9 Hz, 6H); ^13^C NMR (101 MHz, CDCl_3_) δ 165.0, 148.1 (d, *J*_PC_ = 9.5 Hz), 146.2, 137.0, 132.5, 132.2, 131.2 (2C), 129.7 (q, *J*_CF_ = 32.8 Hz), 128.1 (2C), 127.3 (q, *J*_CF_ = 5.5 Hz), 123.5 (q, *J*_CF_ = 274.4 Hz), 123.5 (d, *J*_PC_ = 16.5 Hz), 120.6 (d, *J*_PC_ = 4.2 Hz, 2C), 82.8 (d, *J*_PC_ = 6.3 Hz), 68.6, 33.9, 26.5 (d, *J*_PC_ = 139.3 Hz), 24.4 (2C), 20.8 (d, *J*_PC_ = 4.9 Hz), 14.0; ^31^P NMR (162 MHz, CDCl_3_) 29.2 ppm. HRMS (ESI^+^) *m*/*z* calcd for C_24_H_29_F_3_O_6_P (M + H)^+^ 501.1642, found 501.1654. HPLC purity >99% (*t*_R_ = 6.6).

##### (*E*)-(((5-Acetoxy-4-methylpent-3-en-1-yl)­(4-isopropylphenoxy)­phosphoryl)­oxy)­methyl-2-(trifluoromethyl)­benzoate (**9b**)

Alcohol **8b** (20 mg, 0.04 mmol), acetic anhydride (6.0 mg, 0.06 mmol), and triethylamine (8.0 mg, 0.08 mmol) were dissolved in freshly distilled methylene chloride (3 mL), and the resultant reaction mixture was allowed to react overnight at rt. The reaction mixture was diluted by the addition of water (3 mL), quenched with sodium bicarbonate (2 mL), and extracted with methylene chloride (3 × 4 mL). The combined extracts were dried (Na_2_SO_4_) and filtered through celite, and the filtrate was concentrated *in vacuo*. The residue was purified by column chromatography (silica gel, 100% hexane–50% EtOAc in hexane) and the resulting acetate **9b** was isolated as an oil in 88% yield (19 mg): ^1^H NMR (400 MHz, CDCl_3_) δ 7.79–7.76 (m, 2H), 7.66–7.56 (m, 2H), 7.10 (s, 4H), 5.94 (dd, *J*_PH_ = 13.6 Hz, *J*_HH_ = 5.2 Hz, 1H), 5.84 (dd, *J*_PH_ = 12.4 Hz, *J*_HH_ = 5.2 Hz, 1H), 5.44 (td, *J* = 7.2 Hz, 1.4 Hz, 1H), 4.42 (s, 2H), 2.88–2.79 (m, 1H), 2.49–2.40 (m, 2H), 2.07–1.99 (m, 2H), 2.05 (s, 3H), 1.62 (s, 3H), 1.18 (d, *J* = 6.9 Hz, 6H); ^13^C NMR (101 MHz, CDCl_3_) δ 170.7, 164.4, 147.5 (d, *J*_PC_ = 9.3 Hz), 145.6, 131.8, 131.7, 131.6, 130.6 (2C), 129.1 (q, *J*_CF_ = 32.8 Hz), 127.4 (2C), 126.6 (d, *J*_PC_ = 17.6 Hz), 126.3 (q, *J*_CF_ = 5.5 Hz), 123.8 (q, *J*_CF_ = 274.5 Hz), 120.1 (d, *J*_PC_ = 4.2 Hz, 2C), 82.2 (d, *J*_PC_ = 6.3 Hz), 69.3, 33.2, 25.6 (d, *J*_PC_ = 139.7 Hz), 23.7 (2C), 20.7, 20.3 (d, *J*_PC_ = 4.8 Hz), 13.7; ^31^P NMR (162 MHz, CDCl_3_) 28.9 ppm. HRMS (ESI^+^) *m*/*z* calcd for C_26_H_31_F_3_O_7_P (M + H)^+^ 543.1759, found 543.1755. HPLC purity >99% (*t*_R_ = 6.6).

##### Chloromethyl-3-(trifluoromethyl)­benzoate (**5c**)

To a stirring solution of 3-(trifluoromethyl)­benzoic acid (2.00 g, 10.5 mmol) in dichloromethane (45 mL) was added water (45 mL), sodium bicarbonate (3.53 g, 42.0 mmol), and tetrabutylammonium hydrogen sulfate (0.36 g, 1.06 mmol). The reaction mixture was stirred at rt for one hour, chloromethyl chlorosulfate (1.38 mL, 13.7 mmol) was added dropwise at 0 °C, and the mixture was stirred overnight at rt. The reaction mixture was extracted with dichloromethane (3 × 35 mL) and the combined organic fractions were dried (Na_2_SO_4_) and concentrated *in vacuo* to obtain product **5c** ^36^ as a colorless oil (2.33 g, 93%): ^1^H NMR (CDCl_3_, 500 MHz) δ 8.34 (s, 1H), 8.28–8.27 (d, *J* = 7.9 Hz, 1H), 7.89–7.87 (d, *J* = 7.8 Hz, 1H), 7.65–7.62 (t, *J* = 7.8 Hz, 1H), 5.98 (s, 2H); ^13^C NMR (CDCl_3_, 126 MHz) δ 163.4, 133.2, 131.4 (q, *J*_CF_ = 33.3 Hz), 130.4 (q, *J*_CF_ = 3.6 Hz), 129.5, 129.4, 126.9 (q, *J*_CF_ = 3.9 Hz), 123.5 (q, *J*_CF_ = 273 Hz), 69.4 ppm.

##### (((4-Isopropylphenoxy)­(4-methylpent-3-en-1-yl)­phosphoryl)­oxy)­methyl-3-(trifluoromethyl)­benzoate (**7c**)

The chloromethyl ester **5c** (1.61 g, 6.75 mmol) was dissolved in acetonitrile (10 mL) and transferred to a round-bottom flask containing the phosphonate **6** (800 mg, 2.70 mmol) and sodium iodide (610 mg, 4.07 mmol). The mixture was stirred and heated at reflux for 2 days. The mixture was extracted with diethyl ether (3 × 30 mL), and the combined organic fractions were dried (Na_2_SO_4_) and concentrated *in vacuo*. Purification via column chromatography (10–40% ethyl acetate in hexane) yielded the desired olefin **7c** as a yellow oil (671 mg, 51%): ^1^H NMR (CDCl_3_, 400 MHz) δ 8.29 (s, 1H), 8.19–8.17 (d, *J* = 7.8 Hz, 1H), 7.86–7.84 (d, *J* = 7.8 Hz, 1H), 7.60–7.56 (t, *J* = 7.8 Hz, 1H), 7.10 (s, 4H), 6.00–5.95 (dd, *J*_PH_ = 14.6 Hz, *J*_HH_ = 5.2 Hz, 1H), 5.92–5.88 (dd, *J*_PH_ = 12.4 Hz, *J*_HH_ = 5.2 Hz, 1H), 5.11–5.08 (td, *J* = 7.1, 1.4 Hz, 1H), 2.83–2.77 (m, 1H), 2.43–2.34 (m, 2H), 2.06–1.97 (m, 2H), 1.65 (s, 3H), 1.56 (s, 3H), 1.17–1.16 (d, *J* = 3.9 Hz, 3H), 1.16–1.15 (d, *J* = 3.9 Hz, 3H); ^13^C NMR (CDCl_3_, 101 MHz) δ 163.8, 147.7 (d, *J*_PC_ = 9.5 Hz), 145.7 (d, *J*_PC_ = 1.3 Hz), 133.5 (d, *J*_PC_ = 1.7 Hz), 133.2, 131.3 (q, *J*_CF_ = 33.3 Hz), 130.2 (q, *J*_CF_ = 3.6 Hz), 129.7, 129.2, 127.6 (2C), 126.9 (q, *J*_CF_ = 3.9 Hz), 123.5 (q, *J*_CF_ = 273 Hz), 122.3 (d, *J*_PC_ = 17.5 Hz), 120.3 (d, *J*_PC_ = 4.3 Hz, 2C), 82.3 (d, *J*_PC_ = 6.4 Hz), 33.4, 26.4 (d, *J*_PC_ = 138.4 Hz), 25.6, 23.9 (2C), 20.8 (d, *J*_PC_ = 4.8 Hz), 17.6; ^31^P NMR (CDCl_3_, 162 MHz) δ 29.8 ppm.

##### (*E*)-(((5-Hydroxy-4-methylpent-3-en-1-yl)­(4-isopropylphenoxy)­phosphoryl)­oxy)­methyl-3-(trifluoromethyl)­benzoate (**8c**)

The olefin **7c** (641 mg, 1.32 mmol) was added to a suspension of selenium dioxide (117 mg, 1.05 mmol) and 4-hydroxybenzoic acid (26 mg, 0.19 mmol) in dichloromethane (10 mL). At 0 °C, *tert*-butyl hydroperoxide (70 wt % in H_2_O, 730 μL, 5.29 mmol) was slowly added, and the reaction mixture was stirred at 0 °C for 4 days. The reaction was quenched by the addition of sodium bicarbonate and extracted with dichloromethane (3 × 15 mL). The combined organic fractions were dried (Na_2_SO_4_) and concentrated *in vacuo*. Final purification via column chromatography (100% ether to 2% methanol in ether) afforded the desired alcohol **8c** as an oil (75.2 mg, 11%): ^1^H NMR (CDCl_3_, 500 MHz) δ 8.28 (s, 1H), 8.18–8.17 (d, *J* = 7.8 Hz, 1H), 7.86–7.84 (d, *J* = 7.8 Hz, 1H), 7.60–7.57 (t, *J* = 7.8 Hz, 1H), 7.09 (s, 4H), 5.99–5.95 (dd, *J*_PH_ = 14.5 Hz, *J*_HH_ = 5.2 Hz, 1H), 5.90–5.87 (dd, *J*_PH_ = 12.3 Hz, *J*_HH_ = 5.2 Hz, 1H), 5.43–5.40 (td, *J* = 7.1, 1.4 Hz, 1H), 3.96 (s, 2H), 2.85–2.76 (m, 1H), 2.49–2.41 (m, 2H), 2.08–2.03 (m, 2H), 1.62 (s, 3H), 1.18–1.17 (d, *J* = 5.0 Hz, 3H), 1.16–1.15 (d, *J* = 5.0 Hz, 3H); ^13^C NMR (CDCl_3_, 126 MHz) δ 163.7, 147.5 (d, *J*_PC_ = 9.4 Hz), 145.7 (d, *J*_PC_ = 1.3 Hz), 136.5 (d, *J*_PC_ = 1.4 Hz), 133.0, 131.2 (q, *J*_CF_ = 33.2 Hz), 130.1 (q, *J*_CF_ = 3.6 Hz), 129.5, 129.1, 127.5 (2C), 126.7 (q, *J*_CF_ = 3.9 Hz), 123.3 (q, *J*_CF_ = 273 Hz), 123.0 (d, *J*_PC_ = 16.1 Hz), 120.1 (d, *J*_PC_ = 4.2 Hz, 2C), 82.1 (d, *J*_PC_ = 6.4 Hz), 68.1, 33.2, 26.0 (d, *J*_PC_ = 139.6 Hz), 23.8 (2C), 20.3 (d, *J*_PC_ = 4.9 Hz), 13.4; ^31^P NMR (CDCl_3_, 203 MHz) δ 29.7 ppm. HRMS (ESI^+^) *m*/*z*: calcd for C_24_H_29_F_3_O_6_P (M + H)^+^, 501.1654; found 501.1642. HPLC purity 100% (*t*_R_ = 6.61).

##### (*E*)-(((5-Acetoxy-4-methylpent-3-en-1-yl)­(4-isopropylphenoxy)­phosphoryl)­oxy)­methyl-3-(trifluoromethyl)­benzoate (**9c**)

Alcohol **8c** (30.0 mg, 0.06 mmol), acetic anhydride (9 μL, 0.09 mmol), and triethylamine (17 μL, 0.12 mmol) were dissolved in dichloromethane (3 mL), and the resulting reaction mixture was stirred overnight at rt. The reaction then was quenched by the addition of sodium bicarbonate and extracted with dichloromethane (3 × 8 mL). The combined extracts were dried (Na_2_SO_4_) and concentrated *in vacuo*. Final purification of the residue via column chromatography (100% ether) gave the desired product **9c** as an oil (28.7 mg, 88%): ^1^H NMR (CDCl_3_, 400 MHz) δ 8.28 (s, 1H), 8.18–8.16 (d, *J* = 7.8 Hz, 1H), 7.86–7.84 (d, *J* = 7.8 Hz, 1H), 7.61–7.57 (t, *J* = 7.8 Hz, 1H), 7.09 (s, 4H), 6.00–5.95 (dd, *J*_PH_ = 14.7 Hz, *J*_HH_ = 5.2 Hz, 1H), 5.91–5.87 (dd, *J*_PH_ = 12.2 Hz, *J*_HH_ = 5.2 Hz, 1H), 5.46–5.43 (td, *J* = 7.1, 1.4 Hz, 1H), 4.42 (s, 2H), 2.85–2.75 (m, 1H), 2.50–2.41 (m, 2H), 2.09–2.01 (m, 2H), 2.05 (s, 3H), 1.62 (s, 3H), 1.17–1.16 (d, *J* = 4.1 Hz, 3H), 1.16–1.15 (d, *J* = 4.1 Hz, 3H); ^13^C NMR (CDCl_3_, 101 MHz) δ 170.8, 163.8, 147.7 (d, *J*_PC_ = 9.4 Hz), 145.8 (d, *J*_PC_ = 1.4 Hz), 133.2, 132.0 (d, *J*_PC_ = 1.7 Hz), 131.3 (q, *J*_CF_ = 33.3 Hz), 130.3 (q, *J*_CF_ = 3.6 Hz), 129.7, 129.3, 127.6 (2C), 126.9 (q, *J*_CF_ = 3.9 Hz), 126.7 (d, *J*_PC_ = 17.5 Hz), 123.5 (q, *J*_CF_ = 274 Hz), 120.2 (d, *J*_PC_ = 4.3 Hz, 2C), 82.3 (d, *J*_PC_ = 6.5 Hz), 69.5, 33.4, 25.9 (d, *J*_PC_ = 140.2 Hz), 23.9 (2C), 20.9, 20.6 (d, *J*_PC_ = 4.7 Hz), 13.9; ^31^P NMR (CDCl_3_, 162 MHz) δ 29.0 ppm. HRMS (ESI^+^) *m*/*z*: calcd for C_26_H_31_F_3_O_7_P (M + H)^+^, 543.1759; found 543.1748. HPLC purity 100% (*t*_R_ = 6.57).

##### Chloromethyl-4-(trifluoromethyl)­benzoate (**5d**)

To a stirring solution of 4-trifluoromethylbenzoic acid (2.5 g, 13.1 mmol) in dichloromethane (40 mL) was added water (40 mL), sodium bicarbonate (4.42 g, 52.6 mmol), and tetrabutylammonium hydrogen sulfate (446 mg, 1.31 mmol). After the reaction mixture was stirred at rt for one hour, chloromethyl chlorosulfate (2.8 g, 16.9 mmol) was added dropwise at 0 °C, and the mixture was stirred overnight at rt. The reaction mixture was extracted with dichloromethane (3 × 40 mL) and the combined organic fractions were dried (Na_2_SO_4_) and concentrated *in vacuo* to obtain product **5d** ^36^ as a colorless oil (2.8 g, 90%): ^1^H NMR (400 MHz, CDCl_3_) δ 8.15 (d, *J*_HH_ = 8.4 Hz, 2H), 7.69 (d, *J*_HH_ = 8.4 Hz, 2H), 5.94 (s, 2H); ^13^C NMR (101 MHz, CDCl_3_) δ 163.8, 135.6 (q, *J*_CF_ = 32.9 Hz), 132.2, 130.6 (2C), 125.9 (q, *J*_CF_ = 3.7 Hz, 2C), 123.8 (q, *J*_CF_ = 273.7 Hz), 69.8.

##### (((4-Isopropylphenoxy)­(4-methylpent-3-en-1-yl)­phosphoryl)­oxy)­methyl-4-(trifluoromethyl)­benzoate (**7d**)

The mixed aryl methyl ester **6** (1.1 g, 3.71 mmol) was dissolved in freshly distilled acetonitrile and concentrated *in vacuo* three times and then added to a solution of the chloromethyl ester **5d** (2.2 g, 9.2 mmol, 2.5 equiv) and sodium iodide (835 mg, 5.6 mmol, 1.5 equiv) in acetonitrile. The solution was heated at reflux for 2 days while monitored by TLC analysis. The reaction then was allowed to cool to rt, extracted with diethyl ether, and the combined extracts were washed with brine. The combined organic portions were dried (Na_2_SO_4_) and filtered through celite, and the filtrate was concentrated *in vacuo*. The resulting oil was purified *via* flash chromatography (silica, 100% hexanes to 30% EtOAc in hexanes) to give the desired product **7d** as an oil (0.5 g, 28%): ^1^H NMR (400 MHz, CDCl_3_) δ 8.07 (d, *J*_HH_ = 8.2 Hz, 2H), 7.65 (d, *J*_HH_ = 8.3 Hz, 2H), 7.06 (s, 4H), 5.95 (dd, *J*_PH_ = 15.0 Hz, *J*_HH_ = 5.2 Hz, 1H), 5.87 (dd, *J*_PH_ = 12.3 Hz, *J*_HH_ = 5.2 Hz, 1H), 5.07 (td, *J* = 7.1 Hz, 1.4 Hz, 1H), 2.81–2.72 (m, 1H), 2.40–2.31 (m, 2H), 2.04–1.96 (m, 2H), 1.63 (s, 3H), 1.54 (s, 3H), 1.12 (d, *J* = 6.9 Hz, 6H); ^13^C NMR (101 MHz, CDCl_3_) δ 164.3, 148.1 (d, *J*_PC_ = 9.6 Hz), 146.2, 135.4 (q, *J*_CF_ = 32.9 Hz), 133.9, 130.8 (2C), 130.6, 127.9 (2C), 125.8 (q, *J*_CF_ = 3.7 Hz, 2C), 123.8 (q, *J*_CF_ = 273.7 Hz), 122.6 (d, *J*_PC_ = 17.3 Hz), 120.6 (d, *J*_PC_ = 4.2 Hz, 2C), 82.6 (d, *J*_PC_ = 6.6 Hz), 33.7, 26.7 (d, *J*_PC_ = 138.6 Hz), 25.9, 24.2 (2C), 21.1 (d, *J*_PC_ = 4.8 Hz), 17.9; ^31^P NMR (162 MHz, CDCl_3_) 30.0 ppm.

##### (*E*)-(((5-Hydroxy-4-methylpent-3-en-1-yl)­(4-isopropylphenoxy)­phosphoryl)­oxy)­methyl-4-(trifluoromethyl)­benzoate (**8d**)

The olefin **7d** (200 mg, 0.413 mmol) was added to a solution of selenium dioxide (37 mg, 0.324 mmol) and 4-hydroxybenzoic acid (8.0 mg, 0.057 mmol) in dichloromethane (5 mL). At 0 °C *tert*-butyl hydroperoxide (70 wt % in H_2_O, 0.228 mL, 1.64 mmol) was added slowly to the stirred reaction mixture. The resulting reaction mixture was stirred at 0 °C and allowed to react for 4 days. The reaction mixture was then diluted by the addition of water (3 mL), quenched with Na_2_SO_3_ (3 mL), and extracted with dichloromethane (3 × 5 mL). The combined extracts were dried (Na_2_SO_4_), filtered through celite, and the filtrate was concentrated *in vacuo*. The resulting oil was purified by column chromatography (silica, 100% ether–3% MeOH in ether) to give the desired product **8d** as an oil in 24% yield (50 mg): ^1^H NMR (400 MHz, CDCl_3_) δ 8.02 (d, *J*_HH_ = 8.2 Hz, 2H), 7.61 (d, *J*_HH_ = 8.3 Hz, 2H), 7.01 (s, 4H), 5.89 (dd, *J*_PH_ = 15.0 Hz, *J*_HH_ = 5.2 Hz, 1H), 5.80 (dd, *J*_PH_ = 12.3 Hz, *J*_HH_ = 5.2 Hz, 1H), 5.33 (td, *J* = 7.2 Hz, 1.4 Hz, 1H), 3.89 (s, 2H), 2.77–2.67 (m, 1H), 2.42–2.32 (m, 2H), 2.01–1.85 (m, 2H), 1.55 (s, 3H), 1.09 (d, *J* = 6.9 Hz, 6H); ^13^C NMR (101 MHz, CDCl_3_) δ 164.4, 148.1 (d, *J*_PC_ = 9.5 Hz), 146.3, 137.1, 135.4 (q, *J*_CF_ = 32.9 Hz), 132.4, 130.8 (2C), 128.1 (2C), 126.0 (q, *J*_CF_ = 3.7 Hz, 2C), 123.8 (q, *J*_CF_ = 251.8 Hz), 123.5 (d, *J*_PC_ = 16.0 Hz), 120.7 (d, *J*_PC_ = 4.2 Hz, 2C), 82.6 (d, *J*_PC_ = 6.6 Hz), 68.7, 33.8, 26.6 (d, *J*_PC_ = 140.0 Hz), 25.9 (2C), 20.8 (d, *J*_PC_ = 5.0 Hz), 14.1; ^31^P NMR (162 MHz, CDCl_3_) 29.4 ppm. HRMS (ESI^+^) *m*/*z* calcd for C_24_H_29_F_3_O_6_P (M + H)^+^ 501.1654, found 501.1643. HPLC purity >99% (*t*_R_ = 6.5).

##### (*E*)-(((5-Acetoxy-4-methylpent-3-en-1-yl)­(4-isopropylphenoxy)­phosphoryl)­oxy)­methyl-4-(trifluoromethyl)­benzoate (**9d**)

Alcohol **8d** (20 mg, 0.04 mmol), acetic anhydride (6.0 mg, 0.06 mmol), and triethylamine (8.0 mg, 0.08 mmol) were dissolved in freshly distilled methylene chloride (2 mL), and the resultant reaction mixture was allowed to react overnight at rt. The reaction mixture was diluted by the addition of water (2 mL), quenched with sodium bicarbonate (2 mL), and extracted with methylene chloride (3 × 3 mL). The combined extracts were dried (Na_2_SO_4_) and filtered through celite, and the filtrate was concentrated *in vacuo*. The residue was purified by column chromatography (silica gel, 100% hexane–50% EtOAc in hexane) and the resulting acetate **9d** was isolated as an oil in 88% yield (19 mg): ^1^H NMR (400 MHz, CDCl_3_) δ 8.01 (d, *J*_HH_ = 8.1 Hz, 2H), 7.62 (d, *J*_HH_ = 8.2 Hz, 2H), 7.01 (s, 4H), 5.89 (dd, *J*_PH_ = 15.0 Hz, *J*_HH_ = 5.2 Hz, 1H), 5.80 (dd, *J*_PH_ = 12.3 Hz, *J*_HH_ = 5.2 Hz, 1H), 5.37 (td, *J* = 7.3 Hz, 1.4 Hz, 1H), 4.35 (s, 2H), 2.77–2.68 (m, 1H), 2.42–2.33 (m, 2H), 2.03–1.93 (m, 2H), 1.97 (s, 3H), 1.55 (s, 3H), 1.09 (d, *J* = 6.9 Hz, 6H); ^13^C NMR (101 MHz, CDCl_3_) δ 171.3, 164.3, 148.1 (d, *J*_PC_ = 9.5 Hz), 146.3, 135.6 (q, *J*_CF_ = 32.9 Hz), 132.4 (2C), 130.9 (2C), 128.1 (2C), 127.1 (d, *J*_PC_ = 17.2 Hz, 2C), 126.0 (q, *J*_CF_ = 3.6 Hz), 123.8 (q, *J*_CF_ = 273.7 Hz), 120.6 (d, *J*_PC_ = 4.2 Hz, 2C), 82.6 (d, *J*_PC_ = 6.6 Hz), 69.9, 33.8, 26.4 (d, *J*_PC_ = 140.4 Hz), 24.3 (2C), 21.4, 21.0 (d, *J*_PC_ = 5.0 Hz), 14.4; ^31^P NMR (162 MHz, CDCl_3_) 29.1 ppm. HRMS (ESI^+^) *m*/*z* calcd for C_26_H_31_F_3_O_7_P (M + H)^+^ 543.1759, found 543.1756. HPLC purity >99% (*t*_R_ = 6.5).

##### Chloromethyl-4-acetylbenzoate (**5e**)

To a stirring solution of 4-acetylbenzoic acid (2.0 g, 12.2 mmol) in dichloromethane (40 mL) was added water (40 mL), sodium bicarbonate (4.09 g, 47.6 mmol) and tetrabutylammonium hydrogen sulfate (413 mg, 1.21 mmol). The reaction mixture was stirred at rt for one hour, chloromethyl chlorosulfate (2.6 g, 15.7 mmol) was added dropwise at 0 °C, and the mixture was stirred overnight at rt. Then the reaction mixture was extracted with dichloromethane (3 × 30 mL) and the combined organic fractions were dried (Na_2_SO_4_) and concentrated *in vacuo* to obtain product **5e** as a colorless oil (2.5 g, 97%): ^1^H NMR (400 MHz, CDCl_3_) δ 8.14 (d, *J*_HH_ = 8.3 Hz, 2H), 7.99 (d, *J*_HH_ = 8.3 Hz, 2H), 5.95 (s, 2H), 2.63 (s, 3H); ^13^C NMR (101 MHz, CDCl_3_) δ 197.7, 164.2, 141.4, 132.7, 130.7 (2C), 128.7 (2C), 69.8, 27.3.

##### (((4-Isopropylphenoxy)­(4-methylpent-3-en-1-yl)­phosphoryl)­oxy)­methyl-4-acetylbenzoate (**7e**)

The mixed aryl methyl ester **6** (1.0 g, 3.4 mmol) was dissolved in freshly distilled acetonitrile and concentrated *in vacuo* 3 times and then added to a solution of the chloromethyl ester **5e** (1.79 g, 8.4 mmol, 2.5 equiv) and sodium iodide (759 mg, 5.0 mmol, 1.5 equiv) in acetonitrile. The solution was heated at reflux for 3 days while monitored by TLC analysis. The reaction then was allowed to cool to rt, extracted with diethyl ether, and the combined extracts were washed with brine. The combined organic portions were dried (Na_2_SO_4_) and filtered through celite, and the filtrate was concentrated *in vacuo*. The resulting oil was purified *via* flash chromatography (silica, 100% hexanes to 30% EtOAc in hexanes) to give the desired product **7e** as an oil (400 mg, 26%): ^1^H NMR (400 MHz, CDCl_3_) δ 8.01 (d, *J*_HH_ = 8.3 Hz, 2H), 7.93 (d, *J*_HH_ = 8.3 Hz, 2H), 7.04 (s, 4H), 5.90 (dd, *J*_PH_ = 14.5 Hz, *J*_HH_ = 5.2 Hz, 1H), 5.83 (dd, *J*_PH_ = 12.5 Hz, *J*_HH_ = 5.2 Hz, 1H), 5.05 (td, *J* = 7.2 Hz, 1.4 Hz, 1H), 2.80–2.71 (m, 1H), 2.58 (s, 3H), 2.36–2.27 (m, 2H), 2.01–1.89 (m, 2H), 1.59 (s, 3H), 1.50 (s, 3H), 1.11 (d, *J* = 6.9 Hz, 6H); ^13^C NMR (101 MHz, CDCl_3_) δ 198.0, 164.6, 148.0 (d, *J*_PC_ = 9.5 Hz), 146.2, 141.1, 133.8, 132.8, 130.6 (3C), 128.6 (2C), 127.9, 122.5 (d, *J*_PC_ = 17.3 Hz), 120.6 (d, *J*_PC_ = 4.2 Hz, 2C), 82.6 (d, *J*_PC_ = 6.5 Hz), 33.7, 27.2, 26.6 (d, *J*_PC_ = 138.4 Hz), 25.8, 24.4 (2C), 21.0 (d, *J*_PC_ = 4.9 Hz), 17.9; ^31^P NMR (162 MHz, CDCl_3_) 29.9 ppm.

##### (*E*)-(((5-Hydroxy-4-methylpent-3-en-1-yl)­(4-isopropylphenoxy)­phosphoryl)­oxy)­methyl-4-acetylbenzoate (**8e**)

The olefin **7e** (300 mg, 0.655 mmol) was added to a solution of selenium dioxide (58 mg, 0.52 mmol) and 4-hydroxybenzoic acid (13.0 mg, 0.09 mmol) in dichloromethane (5 mL). At 0 °C *tert*-butyl hydroperoxide (70 wt % in H_2_O, 0.361 mL, 2.6 mmol) was added slowly to the stirred reaction mixture. The resulting reaction mixture was stirred at 0 °C and was allowed to react for 3 days. The reaction mixture was then diluted by the addition of water (4 mL), quenched with Na_2_SO_3_ (3 mL), and extracted with dichloromethane (3 × 5 mL). The combined extracts were dried (Na_2_SO_4_), filtered through celite, and the filtrate was concentrated *in vacuo*. The resulting oil was purified by column chromatography (silica, 100% ether–3% MeOH in ether) to give the desired product **8e** as an oil in 20% yield (60 mg): ^1^H NMR (400 MHz, CDCl_3_) δ 8.07 (d, *J*_HH_ = 8.3 Hz, 2H), 7.98 (d, *J*_HH_ = 8.3 Hz, 2H), 7.09 (s, 4H), 5.96 (dd, *J*_PH_ = 14.5 Hz, *J*_HH_ = 5.2 Hz, 1H), 5.88 (dd, *J*_PH_ = 12.5 Hz, *J*_HH_ = 5.2 Hz, 1H), 5.40 (td, *J* = 7.2 Hz, 1.4 Hz, 1H), 3.96 (s, 2H), 2.85–2.77 (m, 1H), 2.63 (s, 3H), 2.48–2.39 (m, 2H), 2.08–2.00 (m, 2H), 1.61 (s, 3H), 1.16 (d, *J* = 6.9 Hz, 6H); ^13^C NMR (101 MHz, CDCl_3_) δ 197.7, 164.7, 148.1 (d, *J*_PC_ = 9.5 Hz), 146.2, 141.3, 137.1, 132.9, 130.7 (3C), 128.7 (2C), 128.1, 123.6 (d, *J*_PC_ = 16.1 Hz), 120.6 (d, *J*_PC_ = 4.2 Hz, 2C), 82.6 (d, *J*_PC_ = 6.4 Hz), 68.7, 33.8, 27.3, 26.5 (d, *J*_PC_ = 138.5 Hz), 24.4 (2C), 20.8 (d, *J*_PC_ = 4.9 Hz), 14.1; ^31^P NMR (162 MHz, CDCl_3_) 29.3 ppm. HRMS (ESI^+^) *m*/*z* calcd for C_25_H_31_NaO_7_P (M + Na)^+^ 497.1705, found 497.1683. HPLC purity >99% (*t*_R_ = 6.6).

##### (*E*)-(((5-Acetoxy-4-methylpent-3-en-1-yl)­(4-isopropylphenoxy)­phosphoryl)­oxy)­methyl-4-acetylbenzoate (**9e**)

Alcohol **8e** (28 mg, 0.04 mmol), acetic anhydride (9.0 mg, 0.09 mmol), and triethylamine (12.0 mg, 0.12 mmol) were dissolved in freshly distilled methylene chloride (4 mL), and the resultant reaction mixture was allowed to react overnight at rt. Then the reaction mixture was diluted by the addition of water (2 mL), quenched with sodium bicarbonate (2 mL), and extracted with methylene chloride (3 × 3 mL). The combined extracts were dried (Na_2_SO_4_) and filtered through celite, and the filtrate was concentrated *in vacuo*. The residue was purified by column chromatography (silica gel, 100% hexane–50% EtOAc in hexane) and the resulting acetate **9e** was isolated as an oil in 82% yield (25 mg): ^1^H NMR (400 MHz, CDCl_3_) δ 8.07 (d, *J*_HH_ = 8.3 Hz, 2H), 7.98 (d, *J*_HH_ = 8.3 Hz, 2H), 7.09 (s, 4H), 5.96 (dd, *J*_PH_ = 14.5 Hz, *J*_HH_ = 5.2 Hz, 1H), 5.87 (dd, *J*_PH_ = 12.5 Hz, *J*_HH_ = 5.2 Hz, 1H), 5.43 (td, *J* = 7.2 Hz, 1.4 Hz, 1H), 4.40 (s, 2H), 2.85–2.75 (m, 1H), 2.63 (s, 3H), 2.49–2.40 (m, 2H), 2.07–1.99 (m, 2H), 2.05 (s, 3H), 1.61 (s, 3H), 1.16 (d, *J* = 6.9 Hz, 6H); ^13^C NMR (101 MHz, CDCl_3_) δ 197.4, 170.9, 164.3, 147.8 (d, *J*_PC_ = 9.5 Hz), 145.9, 140.9, 132.6, 132.0, 130.4 (3C), 128.4 (2C), 127.8, 126.9 (d, *J*_PC_ = 16.1 Hz), 120.4 (d, *J*_PC_ = 4.2 Hz, 2C), 82.3 (d, *J*_PC_ = 6.4 Hz), 69.6, 33.5, 27.0, 26.1 (d, *J*_PC_ = 140.3 Hz), 24.1 (2C), 21.1, 20.7 (d, *J*_PC_ = 4.9 Hz), 14.0; ^31^P NMR (162 MHz, CDCl_3_) 29.0 ppm. HRMS (ESI^+^) *m*/*z* calcd for C_27_H_34_O_8_P (M + H)^+^ 517.1991, found 517.1973. HPLC purity >99% (*t*_R_ = 6.6).

##### Chloromethyl-4-cyanobenzoate (**5f**)

To a stirring solution of 4-cyanobenzoic acid (1.52 g, 10.3 mmol) in dichloromethane (35 mL) was added water (35 mL), sodium bicarbonate (3.47 g, 41.3 mmol), and tetrabutylammonium hydrogen sulfate (0.35 g, 1.03 mmol). The reaction mixture was stirred at rt for one hour, chloromethyl chlorosulfate (1.36 mL, 13.4 mmol) was added dropwise at 0 °C, and the mixture was stirred overnight at rt. The reaction mixture was extracted with dichloromethane (3 × 30 mL) and the combined organic fractions were dried (Na_2_SO_4_) and concentrated *in vacuo* to obtain product **5f** as a white solid (1.87 g, 93%): ^1^H NMR (CDCl_3_, 500 MHz) δ 8.20–8.18 (d, *J* = 8.2 Hz, 2H), 7.80–7.78 (d, *J* = 8.1 Hz, 2H), 5.97 (s, 2H); ^13^C NMR (CDCl_3_, 126 MHz) δ 163.1, 132.4 (3C), 130.5 (2C), 117.7, 117.4, 69.5 ppm.

##### (((4-Isopropylphenoxy)­(4-methylpent-3-en-1-yl)­phosphoryl)­oxy)­methyl-4-cyanobenzoate (**7f**)

The chloromethyl ester **5f** (1.31 g, 6.70 mmol) was dissolved in acetonitrile (10 mL) and transferred to a round-bottom flask containing the phosphonate **6** (792 mg, 2.68 mmol) and sodium iodide (600 mg, 4.00 mmol). The mixture was stirred and heated at reflux for 2 days. The mixture was extracted with diethyl ether (3 × 35 mL) and the combined organic fractions were dried (Na_2_SO_4_) and concentrated *in vacuo*. Final purification via column chromatography (15–50% ethyl acetate in hexane) yielded the desired olefin **7f** as a yellow oil (736 mg, 62%): ^1^H NMR (CDCl_3_, 500 MHz) δ 8.09–8.07 (d, *J* = 8.2 Hz, 2H), 7.73–7.71 (d, *J* = 8.2 Hz, 2H), 7.09 (s, 4H), 5.98–5.94 (dd, *J*_PH_ = 15.1 Hz, *J*_HH_ = 5.2 Hz, 1H), 5.90–5.87 (dd, *J*_PH_ = 12.3 Hz, *J*_HH_ = 5.2 Hz, 1H), 5.11–5.08 (td, *J* = 7.1, 1.4 Hz, 1H), 2.84–2.78 (m, 1H), 2.41–2.32 (m, 2H), 2.04–1.97 (m, 2H), 1.66 (s, 3H), 1.57 (s, 3H), 1.19–1.18 (d, *J* = 4.6 Hz, 3H), 1.18–1.16 (d, *J* = 4.6 Hz, 3H); ^13^C NMR (CDCl_3_, 126 MHz) δ 163.5, 147.8 (d, *J*_PC_ = 9.4 Hz), 145.8 (d, *J*_PC_ = 1.3 Hz), 133.5 (d, *J*_PC_ = 1.7 Hz), 132.6, 132.3 (2C), 130.5 (2C), 127.6 (2C), 122.3 (d, *J*_PC_ = 17.3 Hz), 120.3 (d, *J*_PC_ = 4.3 Hz, 2C), 117.7, 117.1, 82.4 (d, *J*_PC_ = 6.5 Hz), 33.4, 26.4 (d, *J*_PC_ = 138.3 Hz), 25.6, 24.0, 23.9, 20.8 (d, *J*_PC_ = 4.8 Hz), 17.6 ppm; ^31^P NMR (CDCl_3_, 203 MHz) δ 30.3 ppm.

##### (*E*)-(((5-Hydroxy-4-methylpent-3-en-1-yl)­(4-isopropylphenoxy)­phosphoryl)­oxy)­methyl-4-cyanobenzoate (**8f**)

The olefin **7f** (662 mg, 1.50 mmol) was added to a suspension of selenium dioxide (133 mg, 1.20 mmol) and 4-hydroxybenzoic acid (29 mg, 0.21 mmol) in dichloromethane (10 mL). At 0 °C, *tert*-butyl hydroperoxide (70 wt % in H_2_O, 830 μL, 5.98 mmol) was slowly added, and the reaction mixture was stirred at 0 °C for 3 days. The reaction was quenched by the addition of sodium bicarbonate and extracted with dichloromethane (3 × 10 mL). The combined organic fractions were dried (Na_2_SO_4_) and concentrated *in vacuo*. Final purification *via* column chromatography (100% ether to 3% methanol in ether) afforded the desired alcohol **8f** as an oil (59.7 mg, 9%): ^1^H NMR (CDCl_3_, 400 MHz) δ 8.09–8.07 (d, *J* = 8.4 Hz, 2H), 7.74–7.72 (d, *J* = 8.4 Hz, 2H), 7.09 (s, 4H), 5.99–5.94 (dd, *J*_PH_ = 15.1 Hz, *J*_HH_ = 5.2 Hz, 1H), 5.90–5.85 (dd, *J*_PH_ = 12.1 Hz, *J*_HH_ = 5.2 Hz, 1H), 5.43–5.40 (td, *J* = 7.1, 1.4 Hz, 1H), 3.97 (s, 2H), 2.87–2.76 (m, 1H), 2.49–2.40 (m, 2H), 2.09–2.01 (m, 2H), 1.63 (s, 3H), 1.19–1.18 (d, *J* = 4.4 Hz, 3H), 1.18–1.16 (d, *J* = 4.4 Hz, 3H); ^13^C NMR (CDCl_3_, 101 MHz) δ 163.6, 147.7 (d, *J*_PC_ = 9.5 Hz), 145.9 (d, *J*_PC_ = 1.3 Hz), 136.7, 132.5, 132.3 (2C), 130.5 (2C), 127.7 (2C), 123.0 (d, *J*_PC_ = 15.9 Hz), 120.2 (d, *J*_PC_ = 4.2 Hz, 2C), 117.7, 117.2, 82.3 (d, *J*_PC_ = 6.6 Hz), 68.2, 33.4, 26.1 (d, *J*_PC_ = 139.9 Hz), 24.0, 23.9, 20.4 (d, *J*_PC_ = 5.0 Hz), 13.6; ^31^P NMR (CDCl_3_, 162 MHz) δ 29.4 ppm. HRMS (ESI^+^) *m*/*z*: calcd for C_24_H_29_NO_6_P (M + H)^+^, 458.1732; found 458.1726. HPLC purity 98.5% (*t*_R_ = 6.66).

##### (*E*)-(((5-Acetoxy-4-methylpent-3-en-1-yl)­(4-isopropylphenoxy)­phosphoryl)­oxy)­methyl-4-cyanobenzoate (**9f**)

Alcohol **8f** (28.1 mg, 0.06 mmol), acetic anhydride (9.0 μL, 0.09 mmol), and triethylamine (17 μL, 0.12 mmol) were dissolved in dichloromethane (3 mL), and the resulting reaction mixture was stirred overnight at rt. The reaction was quenched by the addition of sodium bicarbonate and extracted with dichloromethane (3 × 8 mL), and the combined extracts were dried (Na_2_SO_4_) and concentrated *in vacuo*. Final purification via column chromatography (100% ether to 1% methanol in ether) yielded product **9f** as an oil (24.2 mg, 79%): ^1^H NMR (CDCl_3_, 400 MHz) δ 8.08–8.06 (d, *J* = 8.6 Hz, 2H), 7.74–7.72 (d, *J* = 8.6 Hz, 2H), 7.09 (s, 4H), 5.99–5.94 (dd, *J*_PH_ = 15.3 Hz, *J*_HH_ = 5.2 Hz, 1H), 5.89–5.85 (dd, *J*_PH_ = 12.0 Hz, *J*_HH_ = 5.2 Hz, 1H), 5.47–5.43 (td, *J* = 7.1, 1.1 Hz, 1H), 4.43 (s, 2H), 2.86–2.76 (m, 1H), 2.50–2.41 (m, 2H), 2.09–2.00 (m, 2H), 2.06 (s, 3H), 1.63 (s, 3H), 1.19–1.18 (d, *J* = 4.6 Hz, 3H), 1.17–1.16 (d, *J* = 4.6 Hz, 3H); ^13^C NMR (CDCl_3_, 101 MHz) δ 170.8, 163.5, 147.7 (d, *J*_PC_ = 9.5 Hz), 145.9 (d, *J*_PC_ = 1.3 Hz), 132.6, 132.3 (2C), 132.0 (d, *J*_PC_ = 1.7 Hz), 130.5 (2C), 127.7 (2C), 126.7 (d, *J*_PC_ = 17.2 Hz), 120.2 (d, *J*_PC_ = 4.3 Hz, 2C), 117.7, 117.2, 82.3 (d, *J*_PC_ = 6.7 Hz), 69.5, 33.4, 25.9 (d, *J*_PC_ = 140.3 Hz), 24.0, 23.9, 20.9, 20.6 (d, *J*_PC_ = 4.7 Hz), 13.9; ^31^P NMR (CDCl_3_, 162 MHz) δ 29.1 ppm. HRMS (ESI^+^) *m*/*z*: calcd for C_26_H_31_NO_7_P (M + H)^+^, 500.1838; found 500.1832. HPLC purity 100% (*t*_R_ = 6.64).

##### Chloromethyl-4-nitrobenzoate (**5g**)

To a stirring solution of 4-nitrobenzoic acid (1.55 g, 9.27 mmol) in dichloromethane (40 mL) was added water (40 mL), sodium bicarbonate (3.12 g, 37.1 mmol), and tetrabutylammonium hydrogen sulfate (0.31 g, 0.91 mmol). The reaction mixture was stirred at rt for one hour, chloromethyl chlorosulfate (1.22 mL, 12.1 mmol) was added dropwise at 0 °C, and the mixture was stirred overnight at rt. The reaction mixture was extracted with dichloromethane (3 × 25 mL) and the combined organic fractions were dried (Na_2_SO_4_) and concentrated *in vacuo* to obtain product **5g** ^37^ as a white solid (1.97 g, 99%): ^1^H NMR (CDCl_3_, 400 MHz) δ 8.34–8.32 (d, *J* = 9.0 Hz, 2H), 8.28–8.26 (d, *J* = 9.0 Hz, 2H), 5.99 (s, 2H); ^13^C NMR (CDCl_3_, 101 MHz) δ 162.9, 151.01, 133.9, 131.2 (2C), 123.8 (2C), 69.5 ppm.

##### (((4-Isopropylphenoxy)­(4-methylpent-3-en-1-yl)­phosphoryl)­oxy)­methyl-4-nitrobenzoate (**7g**)

The mixed aryl methyl ester 6 (800 mg, 2.70 mmol) was dissolved in freshly distilled dichloromethane (15 mL) and cooled to 0 °C in an ice bath. Trimethylsilyl bromide (1.03 g, 6.5 mmol) was added dropwise, and the resulting reaction mixture was stirred overnight at rt. All the solvents were removed *in vacuo*, and the crude oil was dissolved in tetrahydrofuran and water (1:10 ratio), and stirred for 1 h at rt. After which all volatiles were removed *in vacuo* and residue was coevaporated three times with toluene to remove all traces of water. The resulting crude material was dried overnight at high vacuum for the next step. The phosphonic acid derivative of compound **6** (800 mg, 2.84 mmol) was dried under high vacuum for 24 h before being dissolved in dimethylformamide (8 mL), followed by the addition of triethylamine (1.20 mL, 8.50 mmol) and the chloromethyl ester **5g** (1.53 g, 7.10 mmol). The reaction mixture was stirred at rt for 2 days. The mixture was extracted with diethyl ether (3 × 25 mL) and the combined organic fractions were washed with ice-cold water and dried over Na_2_SO_4_. After concentration *in vacuo*, the residue was purified via column chromatography (20–40% ethyl acetate in hexane) to afford the desired product **7g** as a colorless semisolid (109 mg, 8%): ^1^H NMR (CDCl_3_, 400 MHz) δ 8.27–8.25 (d, *J* = 8.8 Hz, 2H), 8.15–8.13 (d, *J* = 8.9 Hz, 2H), 7.09 (s, 4H), 6.01–5.96 (dd, *J*_PH_ = 15.3 Hz, *J*_HH_ = 5.2 Hz, 1H), 5.93–5.88 (dd, *J*_PH_ = 12.2 Hz, *J*_HH_ = 5.2 Hz, 1H), 5.12–5.09 (td, *J* = 7.1, 1.4 Hz, 1H), 2.84–2.77 (m, 1H), 2.43–2.34 (m, 2H), 2.06–1.98 (m, 2H), 1.66 (s, 3H), 1.58 (s, 3H), 1.18–1.17 (d, *J* = 4.9 Hz, 3H), 1.16–1.15 (d, *J* = 4.9 Hz, 3H); ^13^C NMR (CDCl_3_, 101 MHz) δ 163.3, 150.9, 147.7 (d, *J*_PC_ = 9.5 Hz), 145.8 (d, *J*_PC_ = 1.3 Hz), 134.2, 133.6, 131.3, 131.1 (2C), 127.6, 123.8, 123.6 (2C), 122.3 (d, *J*_PC_ = 17.3 Hz), 120.3 (d, *J*_PC_ = 4.2 Hz), 82.4 (d, *J*_PC_ = 6.7 Hz), 33.4, 26.4 (d, *J*_PC_ = 138.6 Hz), 25.6, 24.0, 23.9, 20.8 (d, *J*_PC_ = 4.8 Hz), 17.7; ^31^P NMR (CDCl_3_, 162 MHz) δ 30.0 ppm.

##### (*E*)-(((5-Hydroxy-4-methylpent-3-en-1-yl)­(4-isopropylphenoxy)­phosphoryl)­oxy)­methyl-4-nitrobenzoate (**8g**)

The olefin **7g** (100 mg, 0.22 mmol) was added to a suspension of selenium dioxide (19 mg, 0.17 mmol) and 4-hydroxybenzoic acid (4 mg, 0.03 mmol) in dichloromethane (3 mL). At 0 °C, *tert*-butyl hydroperoxide (70 wt % in H_2_O, 120 μL, 0.85 mmol) was slowly added, and the reaction mixture was stirred at 0 °C for 3 days. The reaction was quenched by the addition of sodium bicarbonate and extracted with dichloromethane (3 × 10 mL). The combined organic fractions were dried (Na_2_SO_4_) and concentrated *in vacuo*. Final purification via column chromatography (100% ether to 3% methanol in ether) afforded the desired alcohol **8g** as an oil (9.4 mg, 9%): ^1^H NMR (CDCl_3_, 400 MHz) δ 8.28–8.25 (d, *J* = 8.7 Hz, 2H), 8.15–8.13 (d, *J* = 8.7 Hz, 2H), 7.10 (s, 4H), 6.01–5.95 (dd, *J*_PH_ = 15.2 Hz, *J*_HH_ = 5.2 Hz, 1H), 5.91–5.87 (dd, *J*_PH_ = 12.1 Hz, *J*_HH_ = 5.2 Hz, 1H), 5.44–5.40 (td, *J* = 7.1, 1.4 Hz, 1H), 3.98 (s, 2H), 2.86–2.76 (m, 1H), 2.50–2.41 (m, 2H), 2.10–2.01 (m, 2H), 1.63 (s, 3H), 1.19–1.17 (d, *J* = 5.2 Hz, 3H), 1.17–1.16 (d, *J* = 5.2 Hz, 3H); ^13^C NMR (CDCl_3_, 101 MHz) δ 163.3, 151.0, 147.7 (d, *J*_PC_ = 9.6 Hz), 145.9 (d, *J*_PC_ = 1.3 Hz), 136.7, 134.1, 131.2 (2C), 130.7, 127.7, 123.6 (2C), 123.1 (d, *J*_PC_ = 16.0 Hz), 120.2 (d, *J*_PC_ = 4.2 Hz, 2C), 82.4 (d, *J*_PC_ = 6.6 Hz), 68.2, 33.4, 26.1 (d, *J*_PC_ = 139.8 Hz), 24.0, 23.9, 20.4 (d, *J*_PC_ = 5.0 Hz), 13.7; ^31^P NMR (CDCl_3_, 162 MHz) δ 29.4 ppm. HRMS (ESI^+^) *m*/*z*: calcd for C_23_H_29_NO_8_P (M + H)^+^, 478.1631; found 478.1621. HPLC purity 100% (*t*_R_ = 6.61).

##### Chloromethyl-2,4-bis­(trifluoromethyl)­benzoate (**5h**)

To a stirring solution of 2,4-bis­(trifluoromethyl)­benzoic acid (2.01 g, 7.79 mmol) in dichloromethane (45 mL) was added water (45 mL), sodium bicarbonate (2.62 g, 31.2 mmol), and tetrabutylammonium hydrogen sulfate (0.26 g, 0.78 mmol). The reaction mixture was stirred at rt for one hour, chloromethyl chlorosulfate (1.02 mL, 10.1 mmol) was added dropwise at 0 °C, and the mixture was stirred overnight at rt. The reaction mixture was extracted with dichloromethane (3 × 35 mL) and the combined organic fractions were dried (Na_2_SO_4_) and concentrated *in vacuo* to obtain product **5h** as a colorless oil (2.13 g, 89%): ^1^H NMR (CDCl_3_, 500 MHz) δ 8.04 (s, 1H), 8.00–7.98 (d, *J* = 8.1 Hz, 1H), 7.94–7.93 (d, *J* = 8.2 Hz, 1H), 5.94 (s, 2H); ^13^C NMR (CDCl_3_, 126 MHz) δ 163.3, 134.2 (q, *J*_CF_ = 34.1 Hz), 132.7, 131.4, 130.3 (q, *J*_CF_ = 33.9 Hz), 128.9 (q, *J*_CF_ = 3.6 Hz), 124.2 (qq, *J*_CF_ = 3.7 Hz), 122.7 (q, *J*_CF_ = 273 Hz), 122.3 (q, *J*_CF_ = 275 Hz), 69.6 ppm.

##### (((4-Isopropylphenoxy)­(4-methylpent-3-en-1-yl)­phosphoryl)­oxy)­methyl-2,4-bis­(trifluoromethyl)­benzoate (**7h**)

The chloromethyl ester **5h** (1.84 g, 6.00 mmol) was dissolved in acetonitrile (10 mL) and transferred to a round-bottom flask containing the phosphonate **6** (742 mg, 2.51 mmol) and sodium iodide (560 mg, 3.74 mmol). The mixture was stirred and heated at reflux for 2 days. The mixture was extracted with diethyl ether (3 × 30 mL), and the combined organic fractions were dried (Na_2_SO_4_) and concentrated *in vacuo*. Purification via column chromatography (10–30% ethyl acetate in hexane) yielded the desired olefin **7h** as a yellow oil (684 mg, 49%): ^1^H NMR (CDCl_3_, 500 MHz) δ 8.01 (s, 1H), 7.88–7.87 (d, *J* = 8.1 Hz, 1H), 7.84–7.83 (d, *J* = 8.3 Hz, 1H), 7.11 (s, 4H), 5.98–5.94 (dd, *J*_PH_ = 14.5 Hz, *J*_HH_ = 5.2 Hz, 1H), 5.89–5.85 (dd, *J*_PH_ = 12.3 Hz, *J*_HH_ = 5.2 Hz, 1H), 5.13–5.10 (td, *J* = 7.1, 1.2 Hz, 1H), 2.87–2.79 (m, 1H), 2.42–2.35 (m, 2H), 2.04–1.97 (m, 2H), 1.67 (s, 3H), 1.59 (s, 3H), 1.19–1.18 (d, *J* = 3.7 Hz, 3H), 1.18–1.17 (d, *J* = 3.7 Hz, 3H); ^13^C NMR (CDCl_3_, 126 MHz) δ 163.5, 147.8 (d, *J*_PC_ = 9.3 Hz), 145.8 (d, *J*_PC_ = 1.4 Hz), 134.0 (q, *J*_CF_ = 34.0 Hz), 133.5 (d, *J*_PC_ = 1.8 Hz), 132.9, 131.5, 130.3 (q, *J*_CF_ = 33.9 Hz), 128.8 (q, *J*_CF_ = 3.7 Hz), 127.6 (2C), 124.1 (qq, *J*_CF_ = 3.9 Hz), 122.7 (q, *J*_CF_ = 274 Hz), 122.4 (q, *J*_CF_ = 274 Hz), 122.3 (d, *J*_PC_ = 17.5 Hz), 120.3 (d, *J*_PC_ = 4.2 Hz, 2C), 82.7 (d, *J*_PC_ = 6.5 Hz), 33.4, 26.3 (d, *J*_PC_ = 137.9 Hz), 25.6, 23.9 (2C), 20.8 (d, *J*_PC_ = 4.9 Hz), 17.6; ^31^P NMR (CDCl_3_, 203 MHz) δ 30.2 ppm.

##### (*E*)-(((5-Hydroxy-4-methylpent-3-en-1-yl)­(4-isopropylphenoxy)­phosphoryl)­oxy)­methyl-2,4-bis­(trifluoromethyl)­benzoate (**8h**)

The olefin **7h** (600 mg, 1.09 mmol) was added to a suspension of selenium dioxide (96 mg, 0.87 mmol) and 4-hydroxybenzoic acid (21 mg, 0.15 mmol) in dichloromethane (10 mL). At 0 °C, *tert*-butyl hydroperoxide (70 wt % in H_2_O, 600 μL, 4.3 mmol) was slowly added, and the reaction mixture was stirred at 0 °C for 3 days. The reaction was quenched by the addition of sodium bicarbonate and extracted with dichloromethane (3 × 10 mL). The combined organic fractions were dried (Na_2_SO_4_) and concentrated *in vacuo*. The crude material was dissolved in methanol (2 mL) and cooled to 0 °C, and NaBH_4_ (25 mg, 0.07 mmol) was added in several aliquots. The reaction mixture was stirred for 30 min before quenching with ammonium chloride. The solution was extracted with dichloromethane (3 × 10 mL) and the combined extracts were dried (Na_2_SO_4_) and concentrated *in vacuo*. Final purification via column chromatography (100% ether to 2% methanol in ether) afforded the desired alcohol **8h** as an oil (30.4 mg, 6%): ^1^H NMR (CDCl_3_, 500 MHz) δ 8.01 (s, 1H), 7.89–7.87 (d, *J* = 8.1 Hz, 1H), 7.85–7.84 (d, *J* = 8.3 Hz, 1H), 7.11 (s, 4H), 5.98–5.94 (dd, *J*_PH_ = 14.3 Hz, *J*_HH_ = 5.2 Hz, 1H), 5.88–5.84 (dd, *J*_PH_ = 12.3 Hz, *J*_HH_ = 5.2 Hz, 1H), 5.44–5.42 (td, *J* = 7.0, 1.2 Hz 1H), 3.98 (s, 2H), 2.86–2.80 (m, 1H), 2.49–2.42 (m, 2H), 2.09–2.02 (m, 2H), 1.64 (s, 3H), 1.19 (d, *J* = 3.6 Hz, 3H), 1.18–1.17 (d, *J* = 3.6 Hz, 3H); ^13^C NMR (CDCl_3_, 126 MHz) δ 163.6, 147.7 (d, *J*_PC_ = 9.3 Hz), 146.0 (d, *J*_PC_ = 1.2 Hz), 136.7 (d, *J*_PC_ = 1.3 Hz), 134.1 (q, *J*_CF_ = 33.7 Hz), 132.9, 131.5, 130.3 (q, *J*_CF_ = 34.1 Hz), 128.8 (q, *J*_CF_ = 3.6 Hz), 127.7 (2C), 124.2 (qq, *J*_CF_ = 3.7 Hz), 123.2 (d, *J*_PC_ = 16.3 Hz), 121.5 (q, *J*_CF_ = 275 Hz), 121.1 (q, *J*_CF_ = 275 Hz), 120.2 (d, *J*_PC_ = 4.2 Hz, 2C), 82.7 (d, *J*_PC_ = 6.4 Hz), 68.3, 33.4, 26.0 (d, *J*_PC_ = 139.2 Hz), 23.9 (2C), 20.4 (d, *J*_PC_ = 5.1 Hz), 13.6; ^31^P NMR (CDCl_3_, 203 MHz) δ 29.8 ppm. HRMS (ESI^+^) *m*/*z*: calcd for C_25_H_28_F_6_O_6_P (M + H)^+^, 569.1528; found 569.1520. HPLC purity 100% (*t*_R_ = 6.52).

##### (*E*)-(((5-Acetoxy-4-methylpent-3-en-1-yl)­(4-isopropylphenoxy)­phosphoryl)­oxy)­methyl-2,4-bis­(trifluoromethyl)­benzoate (**9h**)

Alcohol **8h** (21.1 mg, 0.04 mmol), acetic anhydride (6 μL, 0.06 mmol), and triethylamine (12 μL, 0.09 mmol) were dissolved in dichloromethane (3 mL), and the resulting reaction mixture was stirred overnight at rt. The reaction then was quenched by the addition of sodium bicarbonate and extracted with dichloromethane (3 × 8 mL). The combined extracts were dried (Na_2_SO_4_) and concentrated *in vacuo*. Final purification of the residue *via* column chromatography (100% ether to 1% methanol in ether) gave the desired product **9h** as an oil (19.3 mg, 85%): ^1^H NMR (CDCl_3_, 400 MHz) δ 8.01 (s, 1H), 7.88–7.86 (d, *J* = 8.3 Hz, 1H), 7.86–7.83 (d, *J* = 8.5 Hz, 1H), 7.11 (s, 4H), 5.99–5.94 (dd, *J*_PH_ = 14.5 Hz, *J*_HH_ = 5.2 Hz, 1H), 5.88–5.84 (dd, *J*_PH_ = 12.2 Hz, *J*_HH_ = 5.2 Hz, 1H), 5.48–5.44 (td, *J* = 7.2, 1.1 Hz, 1H), 4.44 (s, 2H), 2.88–2.78 (m, 1H), 2.51–2.42 (m, 2H), 2.09–2.01 (m, 2H), 2.06 (s, 3H), 1.64 (s, 3H), 1.19 (d, *J* = 3.0 Hz, 3H), 1.18–1.17 (d, *J* = 3.0 Hz, 3H); ^13^C NMR (CDCl_3_, 101 MHz) δ 170.9, 163.5, 147.7 (d, *J*_PC_ = 9.3 Hz), 146.0 (d, *J*_PC_ = 1.3 Hz), 134.0 (q, *J*_CF_ = 34.0 Hz), 132.8, 132.0 (d, *J*_PC_ = 1.7 Hz), 131.4, 130.3 (q, *J*_CF_ = 34.0 Hz), 128.8 (q, *J*_CF_ = 3.6 Hz), 127.7 (2C), 126.7 (d, *J*_PC_ = 17.3 Hz), 124.1 (qq, *J*_CF_ = 3.7 Hz), 122.7 (q, *J*_CF_ = 275 Hz), 122.4 (q, *J*_CF_ = 275 Hz), 120.3 (d, *J*_PC_ = 4.3 Hz, 2C), 82.7 (d, *J*_PC_ = 6.5 Hz), 69.5, 33.4, 25.8 (d, *J*_PC_ = 139.9 Hz), 23.9 (2C), 20.9, 20.6 (d, *J*_PC_ = 4.8 Hz), 13.9; ^31^P NMR (CDCl_3_, 162 MHz) δ 29.1 ppm. HRMS (ESI^+^) *m*/*z*: calcd for C_27_H_30_F_6_O_7_P (M + H)^+^, 611.1633; found 611.1627. HPLC purity 100% (*t*_R_ = 6.51).

##### Chloromethyl-3,5-bis­(trifluoromethyl)­benzoate (**5i**)

To a stirring solution of 3,5-bistrifluoromethylbenzoic acid (2.3 g, 8.9 mmol) in dichloromethane (40 mL) was added water (40 mL), sodium bicarbonate (3.0 g, 35.7 mmol), and tetrabutylammonium hydrogen sulfate (300 mg, 0.9 mmol). After the reaction mixture was stirred at rt for one hour, chloromethyl chlorosulfate (1.9 g, 11.5 mmol) was added dropwise at 0 °C, and the mixture was stirred overnight at rt. The reaction mixture then was extracted with dichloromethane (3 × 30 mL) and the combined organic fractions were dried (Na_2_SO_4_) and concentrated *in vacuo* to obtain product **5i** as a colorless oil (2.5 g, 95%): ^1^H NMR (400 MHz, CDCl_3_) δ 8.52 (s, 2H), 8.11 (s, 1H), 5.99 (s, 2H); ^13^C NMR (101 MHz, CDCl_3_) δ 162.6, 132.9 (q, *J*_CF_ = 32.9 Hz, 2C), 131.3, 130.5 (q, *J*_CF_ = 3.7 Hz, 2C), 127.7 (q, *J*_CF_ = 3.7 Hz), 123.1 (q, *J*_CF_ = 273.9 Hz, 2C), 70.0.

##### (((4-Isopropylphenoxy)­(4-methylpent-3-en-1-yl)­phosphoryl)­oxy)­methyl-3,5- bis­(trifluoromethyl)­benzoate (**7i**)

The mixed aryl methyl ester **6** (1.0 g, 3.37 mmol) was dissolved in freshly distilled acetonitrile and concentrated *in vacuo* 3 times and then added to a solution of the chloromethyl ester **5i** (2.6 g, 8.5 mmol, 2.5 equiv) and sodium iodide (760 mg, 5.0 mmol, 1.5 equiv) in acetonitrile. The solution was heated at reflux for two days while monitored by TLC analysis. The reaction then was allowed to cool to rt, extracted with diethyl ether, and the combined extracts were washed with brine. The combined organic portions were dried (Na_2_SO_4_) and filtered through celite, and the filtrate was concentrated *in vacuo*. The resulting oil was purified *via* flash chromatography (silica, 100% hexanes to 30% EtOAc in hexanes) to give the desired product **7i** as an oil (600 g, 32%): ^1^H NMR (400 MHz, CDCl_3_) δ 8.44 (s, 2H), 8.10 (s, 1H), 7.10 (s, 4H), 6.01 (dd, *J*_PH_ = 15.1 Hz, *J*_HH_ = 5.2 Hz, 1H), 5.92 (dd, *J*_PH_ = 11.8 Hz, *J*_HH_ = 5.2 Hz, 1H), 5.09 (td, *J* = 7.2 Hz, 1.4 Hz, 1H), 2.82–2.72 (m, 1H), 2.42–2.33 (m, 2H), 2.08–1.98 (m, 2H), 1.65 (s, 3H), 1.57 (s, 3H), 1.13 (d, *J* = 6.9 Hz, 6H); ^13^C NMR (101 MHz, CDCl_3_) δ 163.0, 148.1 (d, *J*_PC_ = 9.5 Hz), 146.3, 134.1, 132.6 (q, *J*_CF_ = 34.3 Hz, 2C), 131.5, 130.5 (q, *J*_CF_ = 3.5 Hz, 2C), 128.0 (2C), 127.5 (q, *J*_CF_ = 3.6 Hz), 123.1 (q, *J*_CF_ = 273.8 Hz, 2C), 122.6 (d, *J*_PC_ = 17.4 Hz), 120.5 (d, *J*_PC_ = 4.2 Hz, 2C), 82.9 (d, *J*_PC_ = 6.5 Hz), 33.8, 26.7 (d, *J*_PC_ = 138.3 Hz), 26.0, 24.2 (2C), 21.2 (d, *J*_PC_ = 4.8 Hz), 18.0; ^31^P NMR (162 MHz, CDCl_3_) 29.9 ppm.

##### (*E*)-(((5-Hydroxy-4-methylpent-3-en-1-yl)­(4-isopropylphenoxy)­phosphoryl)­oxy)­methyl-3,5-bis­(trifluoromethyl)­benzoate (**8i**)

The olefin **7i** (597 mg, 1.08 mmol) was added to a solution of selenium dioxide (96 mg, 0.865 mmol) and 4-hydroxybenzoic acid (21.0 mg, 0.15 mmol) in dichloromethane (6 mL). At 0 °C *tert*-butyl hydroperoxide (70 wt % in H_2_O, 0.597 mL, 4.32 mmol) was added slowly to the stirred reaction mixture. The resulting reaction was stirred at 0 °C and allowed to react for three days. The reaction mixture was diluted by the addition of water (4 mL), quenched with Na_2_SO_3_ (3 mL), and extracted with dichloromethane (3 × 5 mL). The combined extracts were dried (Na_2_SO_4_), filtered through celite, and the filtrate was concentrated *in vacuo*. The resulting oil was purified by column chromatography (silica, 100% ether–3% MeOH in ether) to give the desired product **8i** as an oil in 10% yield (60 mg): ^1^H NMR (400 MHz, CDCl_3_) δ 8.45 (s, 2H), 8.10 (s, 1H), 7.10 (s, 4H), 6.01 (dd, *J*_PH_ = 15.1 Hz, *J*_HH_ = 5.2 Hz, 1H), 5.91 (dd, *J*_PH_ = 11.8 Hz, *J*_HH_ = 5.2 Hz, 1H), 5.42 (td, *J* = 7.2 Hz, 1.4 Hz, 1H), 3.97 (s, 2H), 2.81–2.73 (m, 1H), 2.50–2.41 (m, 2H), 2.10–2.02 (m, 2H), 1.63 (s, 3H), 1.13 (d, *J* = 6.9 Hz, 6H); ^13^C NMR (101 MHz, CDCl_3_) δ 163.0, 148.1 (d, *J*_PC_ = 9.5 Hz), 146.4, 137.2, 132.8 (q, *J*_CF_ = 32.8 Hz, 2C), 131.4, 130.5 (q, *J*_CF_ = 3.5 Hz, 2C), 128.1 (2C), 127.5 (q, *J*_CF_ = 3.6 Hz), 123.1 (q, *J*_CF_ = 274.0 Hz, 2C), 123.4 (d, *J*_PC_ = 16.4 Hz), 120.5 (d, *J*_PC_ = 4.2 Hz, 2C), 82.9 (d, *J*_PC_ = 6.6 Hz), 68.7, 33.8, 26.5 (d, *J*_PC_ = 139.6 Hz), 24.3 (2C), 20.8 (d, *J*_PC_ = 4.8 Hz), 14.1; ^31^P NMR (162 MHz, CDCl_3_) 29.4 ppm. HRMS (ESI^+^) *m*/*z* calcd for C_25_H_28_F_6_O_6_P (M + H)^+^ 569.1528, found 569.1515. HPLC purity >99% (*t*_R_ = 6.4).

##### (*E*)-(((5-Acetoxy-4-methylpent-3-en-1-yl)­(4-isopropylphenoxy)­phosphoryl)­oxy)­methyl-3,5-bis­(trifluoromethyl)­benzoate (**9i**)

Alcohol **8i** (22 mg, 0.038 mmol), acetic anhydride (6.0 mg, 0.06 mmol), and triethylamine (8.0 mg, 0.08 mmol) were dissolved in freshly distilled methylene chloride (3 mL), and the resultant reaction mixture was allowed to react overnight at rt. The reaction mixture was diluted by the addition of water (3 mL), quenched with sodium bicarbonate (2 mL), and extracted with methylene chloride (3 × 4 mL). The combined extracts were dried (Na_2_SO_4_) and filtered through celite, and the filtrate was concentrated *in vacuo*. The residue was purified by column chromatography (silica gel, 100% hexane–50% EtOAc in hexane) and the resulting acetate **9i** was isolated as an oil in 85% yield (20 mg): ^1^H NMR (400 MHz, CDCl_3_) δ 8.44 (s, 2H), 8.08 (s, 1H), 7.04 (s, 4H), 5.99 (dd, *J*_PH_ = 15.2 Hz, *J*_HH_ = 5.2 Hz, 1H), 5.90 (dd, *J*_PH_ = 11.7 Hz, *J*_HH_ = 5.2 Hz, 1H), 5.45 (td, *J* = 7.2 Hz, 1.4 Hz, 1H), 4.42 (s, 2H), 2.81–2.74 (m, 1H), 2.50–2.41 (m, 2H), 2.09–2.01 (m, 2H), 2.05 (s, 3H), 1.63 (s, 3H), 1.13 (d, *J* = 6.9 Hz, 6H); ^13^C NMR (101 MHz, CDCl_3_) δ 170.9, 162.7, 147.7 (d, *J*_PC_ = 9.3 Hz), 146.1, 132.5 (q, *J*_CF_ = 34.4 Hz, 2C), 132.2, 131.1, 130.2 (q, *J*_CF_ = 3.5 Hz, 2C), 127.8 (2C), 127.2 (q, *J*_CF_ = 3.6 Hz), 126.6 (d, *J*_PC_ = 17.4 Hz), 122.8 (q, *J*_CF_ = 274.0 Hz, 2C), 120.2 (d, *J*_PC_ = 4.2 Hz, 2C), 82.6 (d, *J*_PC_ = 6.6 Hz), 69.6, 33.5, 26.0 (d, *J*_PC_ = 140.1 Hz), 23.9 (2C), 21.0, 20.6 (d, *J*_PC_ = 4.8 Hz), 14.0; ^31^P NMR (162 MHz, CDCl_3_) 29.1 ppm. HRMS (ESI^+^) *m*/*z* calcd for C_27_H_30_F_6_O_7_P (M + H)^+^ 611.1633, found 611.1624. HPLC purity >99% (*t*_R_ = 6.4).

##### Chloromethyl-4-cyano-2-fluorobenzoate (**5j**)

To a stirring solution of 4-cyano-2-fluorobenzoic acid (1.52 g, 9.21 mmol) in dichloromethane (40 mL) was added water (40 mL), sodium bicarbonate (3.09 g, 36.8 mmol), and tetrabutylammonium hydrogen sulfate (0.31 g, 0.91 mmol). After the reaction mixture was stirred at rt for one hour, chloromethyl chlorosulfate (1.21 mL, 11.9 mmol) was added dropwise at 0 °C, and the mixture was stirred overnight at rt. The reaction mixture was extracted with dichloromethane (3 × 25 mL) and the combined organic fractions were dried (Na_2_SO_4_) and concentrated *in vacuo* to obtain product **5j** as a white solid (1.93 g, 98%): ^1^H NMR (CDCl_3_, 400 MHz) δ 8.13–8.09 (t, *J* = 7.2 Hz, 1H), 7.58–7.55 (dd, *J* = 8.1, 0.8 Hz, 1H), 7.52–7.49 (dd, *J* = 9.8, 1.3 Hz, 1H), 5.96 (s, 2H); ^13^C NMR (CDCl_3_, 101 MHz) δ 161.5 (d, *J*_CF_ = 267 Hz), 160.9 (d, *J*_CF_ = 4.1 Hz), 133.4, 127.9 (d, *J*_CF_ = 4.6 Hz), 121.6 (d, *J*_CF_ = 9.8 Hz), 121.1 (d, *J*_CF_ = 25.8 Hz), 118.7 (d, *J*_CF_ = 9.8 Hz), 116.4 (d, *J*_CF_ = 2.7 Hz), 69.4 ppm.

##### (((4-Isopropylphenoxy)­(4-methylpent-3-en-1-yl)­phosphoryl)­oxy)­methyl-4-cyano-2-fluorobenzoate (**7j**)

The chloromethyl ester **5j** (1.84 g, 8.61 mmol) was dissolved in acetonitrile (12 mL) and transferred to a round-bottom flask containing the phosphonate **6** (1.00 g, 3.38 mmol) and sodium iodide (760 mg, 5.07 mmol). The mixture was stirred and heated at reflux for 2 days. The mixture was extracted with diethyl ether (3 × 30 mL), and the combined organic fractions were dried (Na_2_SO_4_) and concentrated *in vacuo*. Final purification via column chromatography (10–50% ethyl acetate in hexane) yielded the desired olefin **7j** as a yellow oil (757 mg, 52%): ^1^H NMR (CDCl_3_, 400 MHz) δ 8.01–7.97 (t, *J* = 7.5 Hz, 1H), 7.50–7.44 (m, 2H), 7.11 (s, 4H), 6.00–5.95 (dd, *J*_PH_ = 14.6 Hz, *J*_HH_ = 5.2 Hz, 1H), 5.92–5.87 (dd, *J*_PH_ = 12.6 Hz, *J*_HH_ = 5.2 Hz, 1H), 5.13–5.09 (td, *J* = 7.1, 1.4 Hz, 1H), 2.86–2.79 (m, 1H), 2.43–2.34 (m, 2H), 2.08–1.99 (m, 2H), 1.67 (s, 3H), 1.58 (s, 3H), 1.19 (d, *J* = 2.5 Hz, 3H), 1.18–1.17 (d, *J* = 2.5 Hz, 3H); ^13^C NMR (CDCl_3_, 101 MHz) δ 161.5 (d, *J*_CF_ = 267 Hz), 161.1 (d, *J*_CF_ = 4.0 Hz), 147.7 (d, *J*_PC_ = 9.5 Hz), 145.9 (d, *J*_PC_ = 1.3 Hz), 133.6 (d, *J*_PC_ = 1.7 Hz), 133.4, 127.8 (d, *J*_CF_ = 4.6 Hz), 127.6 (2C), 122.2 (d, *J*_PC_ = 17.4 Hz), 121.8 (d, *J*_CF_ = 9.5 Hz), 121.0 (d, *J*_CF_ = 25.7 Hz), 120.3 (d, *J*_PC_ = 4.2 Hz, 2C), 118.4 (d, *J*_CF_ = 9.8 Hz), 116.4 (d, *J*_CF_ = 2.4 Hz), 82.6 (d, *J*_PC_ = 6.5 Hz), 33.4, 26.3 (d, *J*_PC_ = 137.9 Hz), 25.6, 24.0 (2C), 20.8 (d, *J*_PC_ = 5.0 Hz), 17.6; ^31^P NMR (CDCl_3_, 162 MHz) δ 30.0 ppm.

##### (*E*)-(((5-Hydroxy-4-methylpent-3-en-1-yl)­(4-isopropylphenoxy)­phosphoryl)­oxy)­methyl-4-cyano-2-fluorobenzoate (**8j**)

The olefin **7j** (700 mg, 1.63 mmol) was added to a suspension of selenium dioxide (145 mg, 1.31 mmol) and 4-hydroxybenzoic acid (32 mg, 0.23 mmol) in dichloromethane (10 mL). At 0 °C, *tert*-butyl hydroperoxide (70 wt % in H_2_O, 900 μL, 6.52 mmol) was slowly added, and the reaction mixture was stirred at 0 °C for 4 days. The reaction was quenched by the addition of sodium bicarbonate and extracted with dichloromethane (3 × 10 mL). The combined organic fractions were dried (Na_2_SO_4_) and concentrated *in vacuo*. Purification of the residue via column chromatography (100% ether to 3% methanol in ether) afforded the desired alcohol **8j** as an oil (51.3 mg, 7%): ^1^H NMR (CDCl_3_, 500 MHz) δ 8.00–7.97 (t, *J* = 7.5 Hz, 1H), 7.50–7.48 (d, *J* = 8.2 Hz, 1H), 7.47–7.45 (d, *J* = 9.9 Hz, 1H), 7.11–7.10 (d, *J* = 4.7 Hz, 4H), 5.97–5.94 (dd, *J*_PH_ = 14.5 Hz, *J*_HH_ = 5.2 Hz, 1H), 5.89–5.85 (dd, *J*_PH_ = 12.5 Hz, *J*_HH_ = 5.2 Hz, 1H), 5.44–5.41 (td, *J* = 7.1, 1.4 Hz, 1H), 3.97 (s, 2H), 2.86–2.80 (m, 1H), 2.49–2.42 (m, 2H), 2.09–2.02 (m, 2H), 1.64 (s, 3H), 1.20–1.19 (d, *J* = 3.1 Hz, 3H), 1.18 (d, *J* = 3.1 Hz, 3H); ^13^C NMR (CDCl_3_, 126 MHz) δ 161.4 (d, *J*_CF_ = 267 Hz), 161.0 (d, *J*_CF_ = 3.9 Hz), 147.5 (d, *J*_PC_ = 9.3 Hz), 145.8 (d, *J*_PC_ = 1.1 Hz), 136.6 (d, *J*_PC_ = 1.4 Hz), 133.2, 127.6 (d, *J*_CF_ = 4.6 Hz), 127.5 (2C), 122.9 (d, *J*_PC_ = 16.0 Hz), 121.6 (d, *J*_CF_ = 9.5 Hz), 120.8 (d, *J*_CF_ = 25.7 Hz), 120.1 (d, *J*_PC_ = 4.2 Hz, 2C), 118.3 (d, *J*_CF_ = 9.9 Hz), 116.2 (d, *J*_CF_ = 2.5 Hz), 82.4 (d, *J*_PC_ = 6.4 Hz), 68.0, 33.3, 25.9 (d, *J*_PC_ = 139.1 Hz), 23.8 (2C), 20.3 (d, *J*_PC_ = 5.0 Hz), 13.5; ^31^P NMR (CDCl_3_, 203 MHz) δ 29.8 ppm. HRMS (ESI^+^) *m*/*z*: calcd for C_24_H_28_FNO_6_P (M + H)^+^, 476.1638; found 476.1623. HPLC purity 100% (*t*_R_ = 6.62).

##### (*E*)-(((5-Acetoxy-4-methylpent-3-en-1-yl)­(4-isopropylphenoxy)­phosphoryl)­oxy)­methyl-4-cyano-2-fluorobenzoate (**9j**)

Alcohol **8j** (26.9 mg, 0.06 mmol), acetic anhydride (8.6 μL, 0.09 mmol), and triethylamine (17 μL, 0.12 mmol) were dissolved in dichloromethane (2 mL), and the resulting reaction mixture was stirred overnight at rt. The reaction was quenched by the addition of sodium bicarbonate and extracted with dichloromethane (3 × 10 mL). The combined extracts were dried (Na_2_SO_4_) and concentrated *in vacuo* to yield product **9j** as an oil without further purification (25.2 mg, 88%): ^1^H NMR (CDCl_3_, 500 MHz) δ 8.00–7.97 (t, *J* = 7.5 Hz, 1H), 7.50–7.49 (d, *J* = 8.4 Hz, 1H), 7.48–7.46 (d, *J* = 9.8 Hz, 1H), 7.11–7.10 (d, *J* = 3.8 Hz, 4H), 5.98–5.94 (dd, *J*_PH_ = 14.6 Hz, *J*_HH_ = 5.2 Hz, 1H), 5.89–5.86 (dd, *J*_PH_ = 12.4 Hz, *J*_HH_ = 5.2 Hz, 1H), 5.48–5.45 (td, *J* = 7.1, 1.4 Hz, 1H), 4.43 (s, 2H), 2.86–2.80 (m, 1H), 2.50–2.42 (m, 2H), 2.09–2.00 (m, 2H), 2.06 (s, 3H), 1.65 (s, 3H), 1.20–1.19 (d, *J* = 3.1 Hz, 3H), 1.18 (d, *J* = 3.1 Hz, 3H); ^13^C NMR (CDCl_3_, 126 MHz) δ 170.8, 161.4 (d, *J*_CF_ = 266 Hz), 161.1 (d, *J*_CF_ = 4.0 Hz), 147.7 (d, *J*_PC_ = 9.4 Hz), 145.9 (d, *J*_PC_ = 1.1 Hz), 133.3, 132.0 (d, *J*_PC_ = 1.7 Hz), 127.8 (d, *J*_CF_ = 4.5 Hz), 127.6 (2C), 126.7 (d, *J*_PC_ = 17.1 Hz), 121.7 (d, *J*_CF_ = 9.6 Hz), 121.0 (d, *J*_CF_ = 25.7 Hz), 120.2 (d, *J*_PC_ = 4.2 Hz, 2C), 118.4 (d, *J*_CF_ = 9.8 Hz), 116.4 (d, *J*_CF_ = 2.5 Hz), 82.5 (d, *J*_PC_ = 6.4 Hz), 69.5, 33.4, 25.8 (d, *J*_PC_ = 139.6 Hz), 24.0, 23.9, 20.9, 20.6 (d, *J*_PC_ = 4.8 Hz), 13.9; ^31^P NMR (CDCl_3_, 203 MHz) δ 29.5 ppm. HRMS (ESI^+^) *m*/*z*: calcd for C_26_H_30_FNO_7_P (M + H)^+^, 518.1744; found 518.1728. HPLC purity 100% (*t*_R_ = 6.61).

##### Chloromethyl-3-fluoro-4-(trifluoromethyl)­benzoate (**5k**)

To a stirring solution of 3-fluoro-4-trifluoromethylbenzoic acid (1.0 g, 4.8 mmol) in dichloromethane (20 mL) was added water (20 mL), sodium bicarbonate (1.61 g, 19.1 mmol), and tetrabutylammonium hydrogen sulfate (161 mg, 0.5 mmol). The reaction mixture was stirred at rt for one hour then chloromethyl chlorosulfate (1.0 g, 6.0 mmol) was added dropwise at 0 °C, and the mixture was stirred overnight at rt. The reaction mixture was extracted with dichloromethane (3 × 30 mL) and the combined organic fractions were dried (Na_2_SO_4_) and concentrated *in vacuo* to obtain product **5k** as a colorless oil (1.1 g, 89%): ^1^H NMR (400 MHz, CDCl_3_) δ 7.95 (d, *J* = 8.1 Hz, 1H), 7.86 (d, *J* = 10.4 Hz, 1H), 7.72 (t, *J* = 7.3 Hz, 1H), 5.95 (s, 2H); ^13^C NMR (101 MHz, CDCl_3_) δ 162.9, 159.6 (d, *J*_CF_ = 261.3 Hz), 134.6 (d, *J*_CF_ = 7.7 Hz), 128.1 (q, *J*_CF_ = 3.7 Hz), 126.0 (d, *J*_CF_ = 4.1 Hz, 2C), 123.1 (q, *J*_CF_ = 273.9 Hz), 118.8 (d, *J*_CF_ = 22.9 Hz), 69.9.

##### (((4-Isopropylphenoxy)­(4-methylpent-3-en-1-yl)­phosphoryl)­oxy)­methyl-3-fluoro-4-(trifluoromethyl)­benzoate (**7k**)

The mixed aryl methyl ester **6** (0.8 g, 2.7 mmol) was dissolved in freshly distilled acetonitrile and concentrated *in vacuo* 3 times and then added to a solution of the chloromethyl ester **5k** (1.7 g, 6.6 mmol, 2.5 equiv) and sodium iodide (607 mg, 4.0 mmol, 1.5 equiv) in acetonitrile. The solution was heated at reflux for 2 days while monitored by TLC analysis. The reaction then was allowed to cool to rt, extracted with diethyl ether, and the combined extracts were washed with brine. The combined organic portions were dried (Na_2_SO_4_) and filtered through celite, and the filtrate was concentrated *in vacuo*. The resulting oil was purified *via* flash chromatography (silica, 100% hexanes to 30% EtOAc in hexanes) to give the desired product **7k** as an oil (505 g, 37%): ^1^H NMR (400 MHz, CDCl_3_) δ 7.84–7.64 (m, 3H), 7.08 (s, 4H), 5.98 (dd, *J*_PH_ = 15.4 Hz, *J*_HH_ = 5.2 Hz, 1H), 5.89 (dd, *J*_PH_ = 12.0 Hz, *J*_HH_ = 4.2 Hz, 1H), 5.10 (td, *J* = 7.2 Hz, 1.4 Hz, 1H), 2.83–2.75 (m, 1H), 2.43–2.34 (m, 2H), 2.09–1.99 (m, 2H), 1.66 (s, 3H), 1.57 (s, 3H), 1.15 (d, *J* = 6.5 Hz, 6H); ^13^C NMR (101 MHz, CDCl_3_) δ 162.6, 159.5 (d, *J*_PC_ = 261.1 Hz), 147.3 (d, *J*_PC_ = 9.7 Hz), 145.7, 134.2 (d, *J*_CF_ = 7.7 Hz), 133.4, 127.5 (3C), 127.3 (q, *J*_CF_ = 3.7 Hz), 125.3 (d, *J*_CF_ = 4.1 Hz), 123.1 (q, *J*_CF_ = 274.0 Hz), 121.9 (d, *J*_PC_ = 17.3 Hz), 120.0 (d, *J*_PC_ = 4.2 Hz, 2C), 118.1 (d, *J*_CF_ = 22.7 Hz), 82.7 (d, *J*_PC_ = 6.8 Hz), 33.1, 26.1 (d, *J*_PC_ = 138.6 Hz), 25.4, 23.6 (2C), 20.5 (d, *J*_PC_ = 4.9 Hz), 17.4; ^31^P NMR (202 MHz, CDCl_3_) 30.2 ppm.

##### *(E*)-(((5-Hydroxy-4-methylpent-3-en-1-yl)­(4-isopropylphenoxy)­phosphoryl)­oxy)­methyl-3-fluoro-4-(trifluoromethyl)­benzoate (**8k**)

The olefin **7k** (500 mg, 0.99 mmol) was added to a solution of selenium dioxide (88 mg, 0.79 mmol) and 4-hydroxybenzoic acid (19.0 mg, 0.13 mmol) in dichloromethane (6 mL). At 0 °C *tert*-butyl hydroperoxide (70 wt % in H_2_O, 0.550 mL, 3.98 mmol) was added slowly to the stirred reaction mixture. The resulting reaction was stirred at 0 °C and was allowed to react for three days. The reaction mixture was diluted by the addition of water (4 mL), quenched with Na_2_SO_3_ (3 mL), and extracted with dichloromethane (3 × 5 mL). The combined extracts were dried (Na_2_SO_4_), filtered through celite, and the filtrate was concentrated *in vacuo*. The resulting oil was purified by column chromatography (silica, 100% ether–3% MeOH in ether) to give the desired product **8k** as an oil in 11% yield (56 mg): ^1^H NMR (500 MHz, CDCl_3_) δ 7.84 (d, *J*_HH_ = 8.1 Hz, 1H), 7.75 (d, *J*_HH_ = 10.4 Hz, 1H), 7.67 (t, *J*_HH_ = 7.3 Hz, 1H), 7.08 (s, 4H), 5.95 (dd, *J*_PH_ = 15.4 Hz, *J*_HH_ = 5.2 Hz, 1H), 5.86 (dd, *J*_PH_ = 12.0 Hz, *J*_HH_ = 4.2 Hz, 1H), 5.42 (td, *J* = 7.2 Hz, 1.4 Hz, 1H), 3.97 (s, 2H), 2.83–2.75 (m, 1H), 2.48–2.41 (m, 2H), 2.08–2.01 (m, 2H), 1.63 (s, 3H), 1.16 (d, *J* = 6.5 Hz, 6H); ^13^C NMR (126 MHz, CDCl_3_) δ 163.2, 159.5 (d, *J*_PC_ = 261.1 Hz), 148.0 (d, *J*_PC_ = 9.5 Hz), 146.3, 137.1, 134.7 (d, *J*_CF_ = 7.5 Hz), 128.1 (2C), 128.0 (d, *J*_CF_ = 1.2 Hz), 127.9, (d, *J*_CF_ = 1.3 Hz), 125.9 (d, *J*_CF_ = 4.0 Hz), 123.5 (q, *J*_CF_ = 274.0 Hz), 123.3 (d, *J*_PC_ = 15.8 Hz), 120.6 (d, *J*_PC_ = 4.2 Hz, 2C), 118.7 (d, *J*_CF_ = 22.7 Hz), 82.7 (d, *J*_PC_ = 6.8 Hz), 68.5, 33.7, 26.5 (d, *J*_PC_ = 139.9 Hz), 24.2 (2C), 20.8 (d, *J*_PC_ = 4.9 Hz), 14.0; ^31^P NMR (202 MHz, CDCl_3_) 29.8 ppm. HRMS (ESI^+^) *m*/*z* calcd for C_24_H_28_F_4_O_6_P (M + H)^+^ 519.1560, found 519.1552. HPLC purity >99% (*t*_R_ = 6.4).

##### (*E*)-(((5-Acetoxy-4-methylpent-3-en-1-yl)­(4-isopropylphenoxy)­phosphoryl)­oxy)­methyl-3-fluoro-4-(trifluoromethyl)­benzoate (**9k**)

Alcohol **8k** (22 mg, 0.042 mmol), acetic anhydride (6.0 mg, 0.06 mmol), and triethylamine (8.0 mg, 0.08 mmol) were dissolved in freshly distilled methylene chloride (3 mL), and the resultant reaction mixture was allowed to react overnight at rt. The reaction mixture was diluted by the addition of water (3 mL), quenched with sodium bicarbonate (2 mL), and extracted with methylene chloride (3 × 4 mL). The combined extracts were dried (Na_2_SO_4_) and filtered through celite, and the filtrate was concentrated *in vacuo*. The residue was purified by column chromatography (silica gel, 100% hexane–50% EtOAc in hexane) and the resulting acetate **9k** was isolated as an oil in 88% yield (21 mg): ^1^H NMR (500 MHz, CDCl_3_) δ 7.84 (d, *J*_HH_ = 8.1 Hz, 1H), 7.75 (d, *J*_HH_ = 10.4 Hz, 1H), 7.67 (t, *J*_HH_ = 7.3 Hz, 1H), 7.08 (s, 4H), 5.95 (dd, *J*_PH_ = 15.7 Hz, *J*_HH_ = 5.2 Hz, 1H), 5.85 (dd, *J*_PH_ = 11.9 Hz, *J*_HH_ = 4.2 Hz, 1H), 5.45 (td, *J* = 7.2 Hz, 1.4 Hz, 1H), 4.42 (s, 2H), 2.83–2.75 (m, 1H), 2.49–2.41 (m, 2H), 2.08–2.01 (m, 2H), 2.05 (s, 3H), 1.64 (s, 3H), 1.15 (d, *J* = 6.6 Hz, 6H); ^13^C NMR (126 MHz, CDCl_3_) δ 170.7, 162.7, 159.7 (d, *J*_PC_ = 255.3 Hz), 147.4 (d, *J*_PC_ = 9.5 Hz), 145.7, 134.7 (d, *J*_CF_ = 7.5 Hz), 131.9, 127.5 (2C), 127.4 (m, 2C), 126.4 (d, *J*_PC_ = 17.0 Hz), 125.3 (d, *J*_CF_ = 4.0 Hz), 123.5 (q, *J*_CF_ = 274.0 Hz), 120.0 (d, *J*_PC_ = 4.3 Hz, 2C), 118.1 (d, *J*_CF_ = 22.7 Hz), 82.3 (d, *J*_PC_ = 6.7 Hz), 69.3, 33.2, 26.5 (d, *J*_PC_ = 140.3 Hz), 23.7 (2C), 20.8, 20.4 (d, *J*_PC_ = 4.6 Hz), 13.8; ^31^P NMR (202 MHz, CDCl_3_) 29.5 ppm. HRMS (ESI^+^) *m*/*z* calcd for C_26_H_30_F_4_O_7_P (M + H)^+^ 561.1665, found 561.1658. HPLC purity >99% (*t*_R_ = 6.5).

### Biological Assays

#### Materials

The following materials were sourced from commercial suppliers for this study. The APC-conjugated anti-γδ-TCR (clone B1) antibody, the PE-conjugated anti-CD3 (UCHT1) antibody, and the interferon γ enzyme-linked immunosorbent assay kit were sourced from Biolegend (San Diego, CA). HMBPP was purchased from Cayman Chemical (Ann Arbor, MI). The TCR γ/δ+ T cell isolation kit and interleukin 2 (human IL-2-IS, research grade) were procured from Miltenyi (Bergisch Gladbach, Germany). Pooled human plasma was purchased from Innovative Research (Novi, MI). Lymphoprep was obtained from Cosmo Bio USA (Carlsbad, CA).

#### Cells

Buffy coat, used for the isolation of peripheral blood mononuclear cells via density gradient centrifugation, was obtained from Research Blood Components (Boston, MA). K562 cells were previously obtained from Sigma Aldrich (St. Louis, MO). Both T cells and K562 cells were maintained at 37 °C and 5% CO_2_ in T cell media (RPMI-1640, 1.5 g/L sodium bicarbonate, 10% heat inactivated FBS, 10 mM HEPES pH 7.6, 10 mM nonessential amino acids, 10 mM sodium pyruvate, 1× penicillin-streptomycin, and 50 μM β-mercaptoethanol). T cell cultures additionally contained 5 ng/mL IL-2 supplied every 3 days.

#### γ9δ2 T-Cell Proliferation

Test compounds were assessed for their capacity to promote the expansion of human peripheral blood γ9δ2 T cells.^13^ Peripheral blood mononuclear cells (3 × 10^6^ cells in 1.5 mL per well of a 12-well plate) were stimulated for 3 days with the test compounds and IL-2, followed by two washes and an additional 11-day culture period with IL-2. The proportion of γ9δ2 T cells was quantified by flow cytometry using CD3 and γδ-TCR staining. HMBPP and POM_2_-C-HMBP (100 nM) served as positive controls. EC_50_ values were defined as the concentration required to achieve 50% of the maximal proliferative response. Each experiment was conducted at least three times with cells from a minimum of two independent donors (*n* = 3). Data analysis was performed using GraphPad Prism 10, with EC_50_ values and corresponding 95% confidence intervals reported. Where indicated, γδ T-cell abundance was estimated by normalizing the number of γδ TCR^+^ events to the recorded acquisition time at a constant flow rate; fold-change was then calculated relative to unstimulated controls.

#### ELISA for Interferon-γ

Test compounds were evaluated for their ability to enable K562 cells to stimulate cytokine production by activated effector γ9δ2 T cells.^33^ K562 cells were cultured in T cell media at densities below 0.5 × 10^6^ and split one day before the experiment. Purified γ9δ2 T cells, previously expanded with HMBPP as above and stored in liquid nitrogen, were thawed and maintained overnight with IL-2 supplementation. For the assay, K562 cells were exposed to the test compounds in T cell media for 1 h. Following incubation, cells were washed three times using centrifugation (20 s, benchtop centrifuge, strip tubes, 250 μL). Media was removed from pellets, and cells were resuspended in T cell media. In a 96-well plate, 20 μL of compound-treated K562 cells were added to effector T cells at a 3:1 ratio (T cells:K562 cells), with each well containing 4000 K562 cells and 12,000 T cells. Plates were incubated for 20 h before assessing interferon-γ release by ELISA. The results were analyzed by generating interferon-γ release versus concentration plots and determining EC_50_ values using GraphPad Prism software. Each ELISA was conducted at least three times with cells from a minimum of two independent donors (*n* = 3).

#### Plasma Stability

Stability of the compounds was quantified by LC-MS after incubation with human plasma for various times. Pooled human plasma was diluted to 50% with Tris-buffered saline at pH 7.5. Test compounds were added to a final concentration of 100 μM in a volume of 200 μL. Compounds were incubated at 37 °C. At each time point, 25 μL of sample was extracted using 75 μL of LC-MS grade acetonitrile and vortexed. Precipitated debris was separated by centrifugation at 10,000 rcf for 2 min. Then, 10 μL of the extract was analyzed with a Waters Synapt G2-Si mass spectrometer in positive polarity mode. The chromatographic gradient progressed from 50 to 90% acetonitrile over 9 min. The precursor ion [M + H]^+^ and sodium adduct [M + Na]^+^ were monitored based on calculated *m*/*z* values. The integrated peak areas for both ions were summed and divided by the initial values to determine the fraction remaining.

#### Cellular Metabolism Studies

The cellular metabolism of the prodrugs was determined using negative-mode LC-MS after incubation with K562 cells as reported previously.^27^

## Supplementary Material





## Notes

NMR spectra and Supporting Information figures with additional bioassay data summarized in Tables 3–5 (PDF)
Molecular formula strings (CSV)

## Abbreviations Used

BTN: butyrophilin
HMBPP: (*E*)-4-hydroxy-3-methyl-but-2-enyl diphosphate
POM: pivaloyloxymethyl
TCR: T cell receptor
