# Supplementary material for: Benzoic Acid Derivatives Improve Plasma Stability of Diester Butyrophilin Ligand Prodrugs
Source: J Med Chem. 2026 Feb 12;69(4):4329–45. doi: 10.1021/acs.jmedchem.5c03034 (PMC12951462; doi:10.1021/acs.jmedchem.5c03034)
Supplement: Supplementary file 1 [file jm5c03034_si_001.pdf]

Benzoic acid derivatives improve plasma stability of diester butyrophilin ligand prodrugs

Parker A. Kintigh,[1] Umed Singh,[1] Girija Pawge,[2] Sidra Bashir,[2] Chia-Hung Christine Hsiao,[2] Andrew J. Wiemer,[2,3*] and David F. Wiemer[1*]

[1]*Department of Chemistry, University of Iowa, Iowa City, Iowa 52242-1294, United States*

[2]*Department of Pharmaceutical Sciences, University of Connecticut, Storrs, CT 06269-3092, United States*

[3]*Institute for Systems Genomics, University of Connecticut, Storrs, CT 06269-3092, United States*

Andrew J. Wiemer: andrew.wiemer@uconn.edu

David F. Wiemer: david-wiemer@uiowa.edu

Table of Contents

^{1}H NMR Spectrum of Compound **5a** ($CDCl_3$, 500 MHz) ... 7

^{13}C NMR Spectrum of Compound **5a** ($CDCl_3$, 126 MHz) ... 8

^{1}H NMR Spectrum of Compound **7a** ($CDCl_3$, 500 MHz) ... 9

^{13}C NMR Spectrum of Compound **7a** ($CDCl_3$, 126 MHz) ... 10

^{31}P NMR Spectrum of Compound **7a** ($CDCl_3$, 203 MHz) ... 11

^{1}H NMR Spectrum of Compound **8a** ($CDCl_3$, 500 MHz) ... 12

^{13}C NMR Spectrum of Compound **8a** ($CDCl_3$, 126 MHz) ... 13

^{31}P NMR Spectrum of Compound **8a** ($CDCl_3$, 203 MHz) ... 14

^{1}H NMR Spectrum of Compound **9a** ($CDCl_3$, 500 MHz) ... 15

^{13}C NMR Spectrum of Compound **9a** ($CDCl_3$, 126 MHz) ... 16

^{31}P NMR Spectrum of Compound **9a** ($CDCl_3$, 203 MHz) ... 17

^{1}H NMR Spectrum of Compound **5b** ($CDCl_3$, 400 MHz) ... 18

^{13}C NMR Spectrum of Compound **5b** ($CDCl_3$, 101 MHz) ... 19

^{1}H NMR Spectrum of Compound **7b** ($CDCl_3$, 400 MHz) ... 20

^{13}C NMR Spectrum of Compound **7b** ($CDCl_3$, 101 MHz) ... 21

^{1}P NMR Spectrum of Compound **7b** ($CDCl_3$, 203 MHz) ... 22

^{1}H NMR Spectrum of Compound **8b** ($CDCl_3$, 400 MHz) ... 23

^{13}C NMR Spectrum of Compound **8b** ($CDCl_3$, 101 MHz) ... 24

^{1}P NMR Spectrum of Compound **8b** ($CDCl_3$, 203 MHz) ... 25

^{1}H NMR Spectrum of Compound **9b** ($CDCl_3$, 400 MHz) ... 26

^{13}C NMR Spectrum of Compound **9b** ($CDCl_3$, 101 MHz) ... 27

^{31}P NMR Spectrum of Compound **9b** ($CDCl_3$, 162 MHz) ... 28

^{1}H NMR Spectrum of Compound **5c** ($CDCl_3$, 500 MHz) ... 29

^{13}C NMR Spectrum of Compound **5c** ($CDCl_3$, 126 MHz) ... 30

^{1}H NMR Spectrum of Compound **7c** ($CDCl_3$, 400 MHz) ... 31

^{13}C NMR Spectrum of Compound **7c** ($CDCl_3$, 101 MHz) ... 32

^{31}P NMR Spectrum of Compound **7c** ($CDCl_3$, 162 MHz) ... 33

^{1}H NMR Spectrum of Compound **8c** ($CDCl_3$, 500 MHz) ... 34

^{13}C NMR Spectrum of Compound **8c** ($CDCl_3$, 126 MHz) ... 35

^{31}P NMR Spectrum of Compound **8c** ($CDCl_3$, 203 MHz) ... 36

^{1}H NMR Spectrum of Compound **9c** ($CDCl_3$, 400 MHz) ... 37

^{13}C NMR Spectrum of Compound **9c** ($CDCl_3$, 101 MHz) 38
^{31}P NMR Spectrum of Compound **9c** ($CDCl_3$, 162 MHz) 39
^{1}H NMR Spectrum of Compound **5d** ($CDCl_3$, 400 MHz) 40
^{13}C NMR Spectrum of Compound **5d** ($CDCl_3$, 101 MHz) 41
^{1}H NMR Spectrum of Compound **7d** ($CDCl_3$, 400 MHz) 42
^{13}C NMR Spectrum of Compound **7d** ($CDCl_3$, 101 MHz) 43
^{31}P NMR Spectrum of Compound **7d** ($CDCl_3$, 162 MHz) 44
^{1}H NMR Spectrum of Compound **8d** ($CDCl_3$, 400 MHz) 45
^{13}C NMR Spectrum of Compound **8d** ($CDCl_3$, 101 MHz) 46
^{31}P NMR Spectrum of Compound **8d** ($CDCl_3$, 162 MHz) 47
^{1}H NMR Spectrum of Compound **9d** ($CDCl_3$, 400 MHz) 48
^{13}C NMR Spectrum of Compound **9d** ($CDCl_3$, 101 MHz) 49
^{31}P NMR Spectrum of Compound **9d** ($CDCl_3$, 162 MHz) 50
^{1}H NMR Spectrum of Compound **5e** ($CDCl_3$, 400 MHz) 51
^{13}C NMR Spectrum of Compound **5e** ($CDCl_3$, 101 MHz) 52
^{1}H NMR Spectrum of Compound **7e** ($CDCl_3$, 400 MHz) 53
^{13}C NMR Spectrum of Compound **7e** ($CDCl_3$, 101 MHz) 54
^{31}P NMR Spectrum of Compound **7e** ($CDCl_3$, 162 MHz) 55
^{1}H NMR Spectrum of Compound **8e** ($CDCl_3$, 400 MHz) 56
^{13}C NMR Spectrum of Compound **8e** ($CDCl_3$, 101 MHz) 57
^{31}P NMR Spectrum of Compound **8e** ($CDCl_3$, 162 MHz) 58
^{1}H NMR Spectrum of Compound **9e** ($CDCl_3$, 400 MHz) 59
^{13}C NMR Spectrum of Compound **9e** ($CDCl_3$, 101 MHz) 60
^{31}P NMR Spectrum of Compound **9e** ($CDCl_3$, 162 MHz) 61
^{1}H NMR Spectrum of Compound **5f** ($CDCl_3$, 500 MHz) 62
^{13}C NMR Spectrum of Compound **5f** ($CDCl_3$, 126 MHz) 63
^{1}H NMR Spectrum of Compound **7f** ($CDCl_3$, 500 MHz) 64
^{13}C NMR Spectrum of Compound **7f** ($CDCl_3$, 126 MHz) 65
^{31}P NMR Spectrum of Compound **7f** ($CDCl_3$, 203 MHz) 66
^{1}H NMR Spectrum of Compound **8f** ($CDCl_3$, 400 MHz) 67
^{13}C NMR Spectrum of Compound **8f** ($CDCl_3$, 101 MHz) 68
^{31}P NMR Spectrum of Compound **8f** ($CDCl_3$, 162 MHz) 69
^{1}H NMR Spectrum of Compound **9f** ($CDCl_3$, 400 MHz) 70

^{13}C NMR Spectrum of Compound **9f** ($CDCl_3$, 101 MHz) ... 71
^{31}P NMR Spectrum of Compound **9f** ($CDCl_3$, 162 MHz) ... 72
^{1}H NMR Spectrum of Compound **5g** ($CDCl_3$, 400 MHz) ... 73
^{13}C NMR Spectrum of Compound **5g** ($CDCl_3$, 101 MHz) ... 74
^{1}H NMR Spectrum of Compound **7g** ($CDCl_3$, 400 MHz) ... 75
^{13}C NMR Spectrum of Compound **7g** ($CDCl_3$, 101 MHz) ... 76
^{31}P NMR Spectrum of Compound **7g** ($CDCl_3$, 162 MHz) ... 77
^{1}H NMR Spectrum of Compound **8g** ($CDCl_3$, 400 MHz) ... 78
^{13}C NMR Spectrum of Compound **8g** ($CDCl_3$, 101 MHz) ... 79
^{31}P NMR Spectrum of Compound **8g** ($CDCl_3$, 162 MHz) ... 80
^{1}H NMR Spectrum of Compound **5h** ($CDCl_3$, 500 MHz) ... 81
^{13}C NMR Spectrum of Compound **5h** ($CDCl_3$, 126 MHz) ... 82
^{1}H NMR Spectrum of Compound **7h** ($CDCl_3$, 500 MHz) ... 83
^{13}C NMR Spectrum of Compound **7h** ($CDCl_3$, 126 MHz) ... 84
^{31}P NMR Spectrum of Compound **7h** ($CDCl_3$, 203 MHz) ... 85
^{1}H NMR Spectrum of Compound **8h** ($CDCl_3$, 500 MHz) ... 86
^{13}C NMR Spectrum of Compound **8h** ($CDCl_3$, 126 MHz) ... 87
^{31}P NMR Spectrum of Compound **8h** ($CDCl_3$, 203 MHz) ... 88
^{1}H NMR Spectrum of Compound **9h** ($CDCl_3$, 400 MHz) ... 89
^{13}C NMR Spectrum of Compound **9h** ($CDCl_3$, 101 MHz) ... 90
^{31}P NMR Spectrum of Compound **9h** ($CDCl_3$, 162 MHz) ... 91
^{1}H NMR Spectrum of Compound **5i** ($CDCl_3$, 400 MHz) ... 92
^{13}C NMR Spectrum of Compound **5i** ($CDCl_3$, 101 MHz) ... 93
^{1}H NMR Spectrum of Compound **7i** ($CDCl_3$, 400 MHz) ... 94
^{13}C NMR Spectrum of Compound **7i** ($CDCl_3$, 101 MHz) ... 95
^{31}P NMR Spectrum of Compound **7i** ($CDCl_3$, 162 MHz) ... 96
^{1}H NMR Spectrum of Compound **8i** ($CDCl_3$, 400 MHz) ... 97
^{13}C NMR Spectrum of Compound **8i** ($CDCl_3$, 101 MHz) ... 98
^{31}P NMR Spectrum of Compound **8i** ($CDCl_3$, 162 MHz) ... 99
^{1}H NMR Spectrum of Compound **9i** ($CDCl_3$, 400 MHz) ... 100
^{13}C NMR Spectrum of Compound **9i** ($CDCl_3$, 101 MHz) ... 101
^{31}P NMR Spectrum of Compound 9**i** ($CDCl_3$, 162 MHz) ... 102
^{1}H NMR Spectrum of Compound **5j** ($CDCl_3$, 400 MHz) ... 103

^{31}C NMR Spectrum of Compound **5j** ($CDCl_3$, 101 MHz) ... 104
^{1}H NMR Spectrum of Compound **7j** ($CDCl_3$, 400 MHz) ... 105
^{13}C NMR Spectrum of Compound **7j** ($CDCl_3$, 101 MHz) ... 106
^{31}P NMR Spectrum of Compound **7j** ($CDCl_3$, 162 MHz) ... 107
^{1}H NMR Spectrum of Compound **8j** ($CDCl_3$, 500 MHz) ... 108
^{13}C NMR Spectrum of Compound **8j** ($CDCl_3$, 126 MHz) ... 109
^{31}P NMR Spectrum of Compound **8j** ($CDCl_3$, 203 MHz) ... 110
^{1}H NMR Spectrum of Compound **9j** ($CDCl_3$, 500 MHz) ... 111
^{13}C NMR Spectrum of Compound **9j** ($CDCl_3$, 126 MHz) ... 112
^{31}P NMR Spectrum of Compound **9j** ($CDCl_3$, 203 MHz) ... 113
^{1}H NMR Spectrum of Compound **5k** ($CDCl_3$, 400 MHz) ... 114
^{13}C NMR Spectrum of Compound **5k** ($CDCl_3$, 126 MHz) ... 115
^{1}H NMR Spectrum of Compound **7k** ($CDCl_3$, 400 MHz) ... 116
^{13}C NMR Spectrum of Compound **7k** ($CDCl_3$, 101 MHz) ... 117
^{31}P NMR Spectrum of Compound **7k** ($CDCl_3$, 203 MHz) ... 118
^{1}H NMR Spectrum of Compound **8k** ($CDCl_3$, 500 MHz) ... 119
^{13}C NMR Spectrum of Compound **8k** ($CDCl_3$, 126 MHz) ... 120
^{31}P NMR Spectrum of Compound **8k** ($CDCl_3$, 203 MHz) ... 121
^{1}H NMR Spectrum of Compound **9k** ($CDCl_3$, 500 MHz) ... 122
^{13}C NMR Spectrum of Compound **9k** ($CDCl_3$, 126 MHz) ... 123
^{31}P NMR Spectrum of Compound **9k** ($CDCl_3$, 203 MHz) ... 124
HPLC Chromatogram of Compound **8a** ... 125
HPLC Chromatogram of Compound **9a** ... 126
HPLC Chromatogram of Compound **8b** ... 127
HPLC Chromatogram of Compound **9b** ... 128
HPLC Chromatogram of Compound **8c** ... 129
HPLC Chromatogram of Compound **9c** ... 130
HPLC Chromatogram of Compound **8d** ... 131
HPLC Chromatogram of Compound **9d** ... 132
HPLC Chromatogram of Compound **8e** ... 133
HPLC Chromatogram of Compound **9e** ... 134
HPLC Chromatogram of Compound **8f** ... 135
HPLC Chromatogram of Compound **9f** ... 136

HPLC Chromatogram of Compound **8g** 137
HPLC Chromatogram of Compound **8h** 138
HPLC Chromatogram of Compound **9h** 139
HPLC Chromatogram of Compound **8i** 140
HPLC Chromatogram of Compound **9i** 141
HPLC Chromatogram of Compound **8j** 142
HPLC Chromatogram of Compound **9j** 143
HPLC Chromatogram of Compound **8k** 144
HPLC Chromatogram of Compound **9k** 145
Supplemental Figure S1. 146
Supplemental Figure S2.. 147
Supplemental Figure S3.. 148
Supplemental Figure S4.. 149
Supplemental Figure S5.. 150

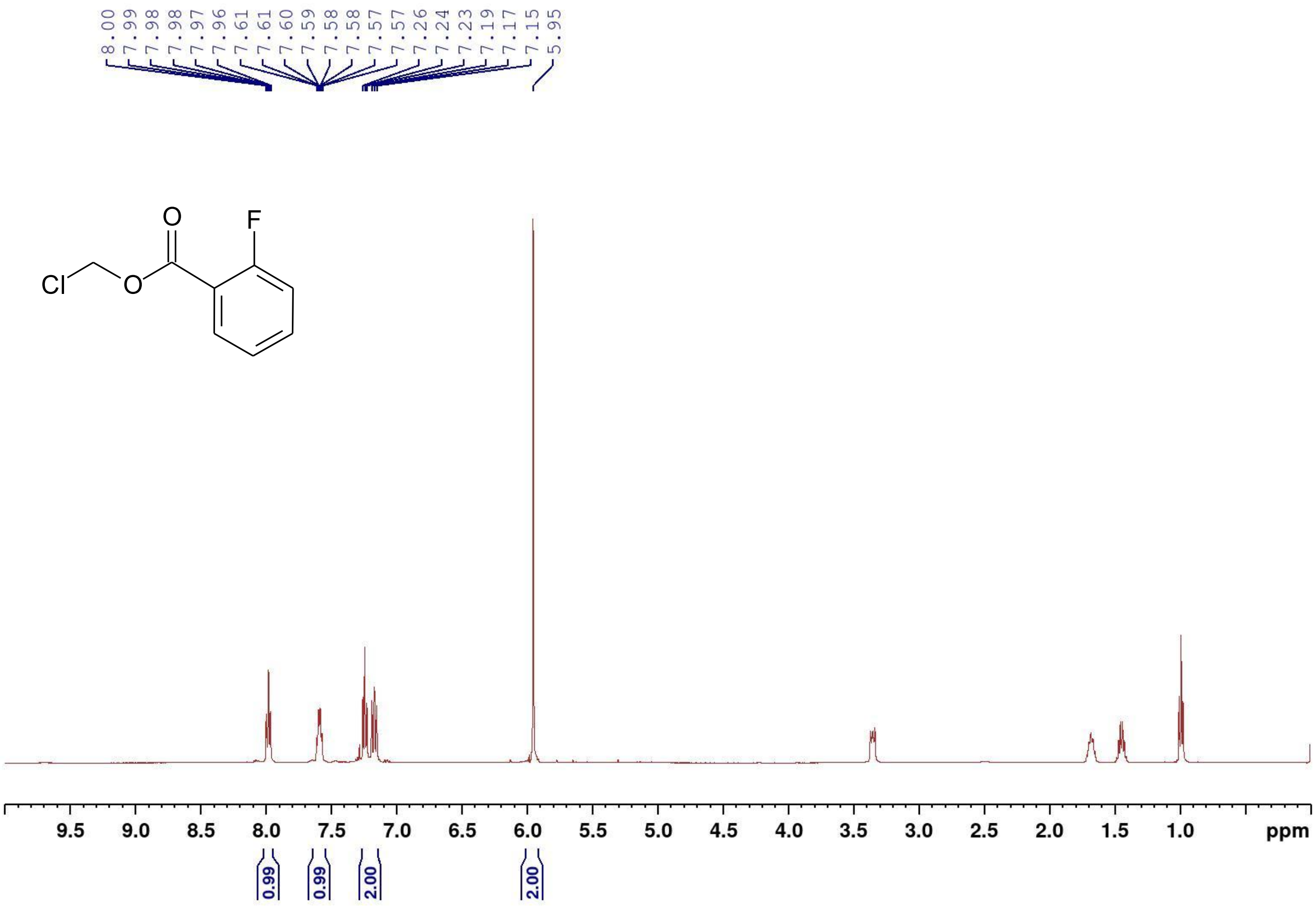

^{1}H NMR Spectrum of Compound **5a** ($CDCl_3$, 500 MHz)

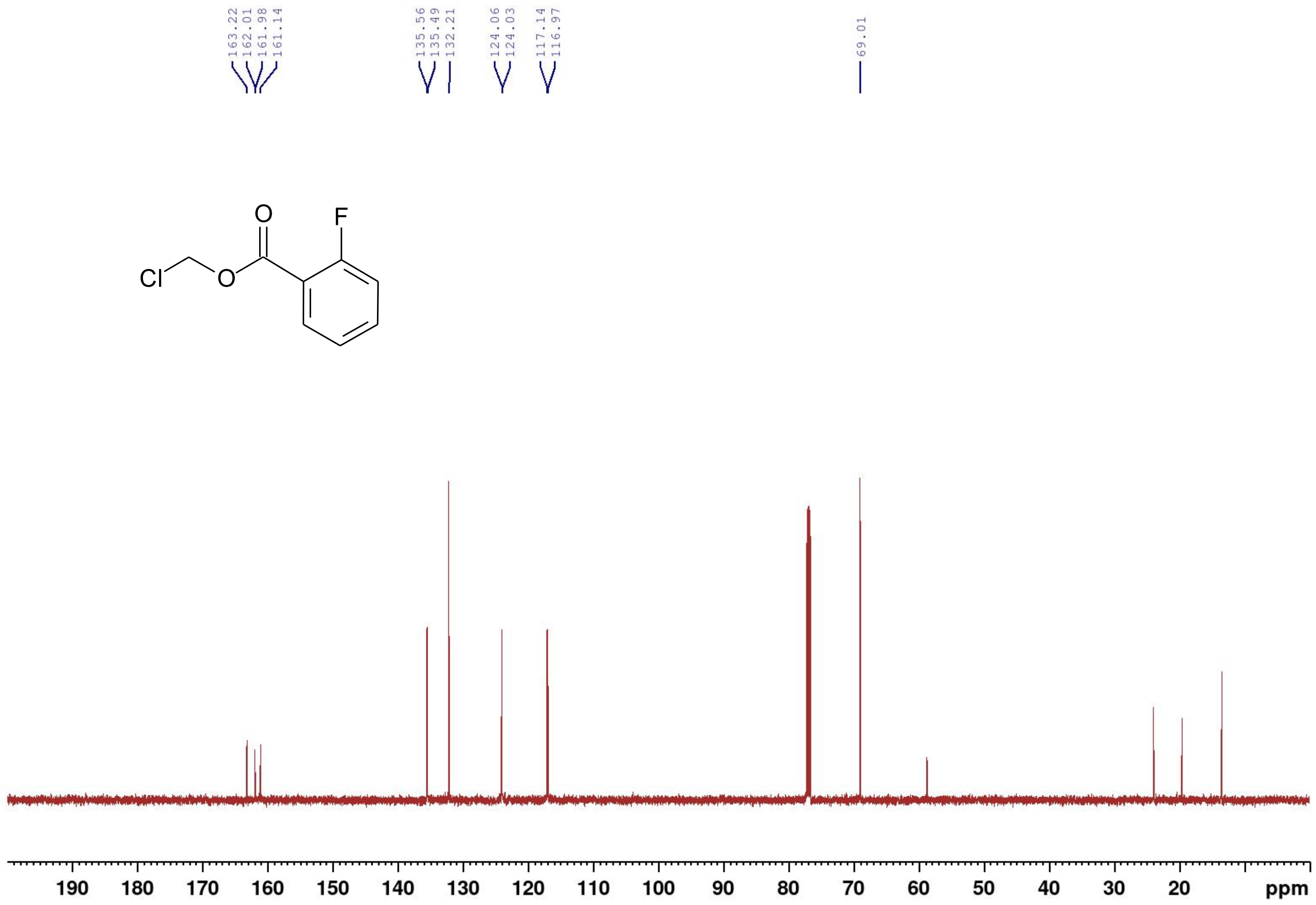

^{13}C NMR Spectrum of Compound **5a** ($CDCl_3$, 126 MHz)

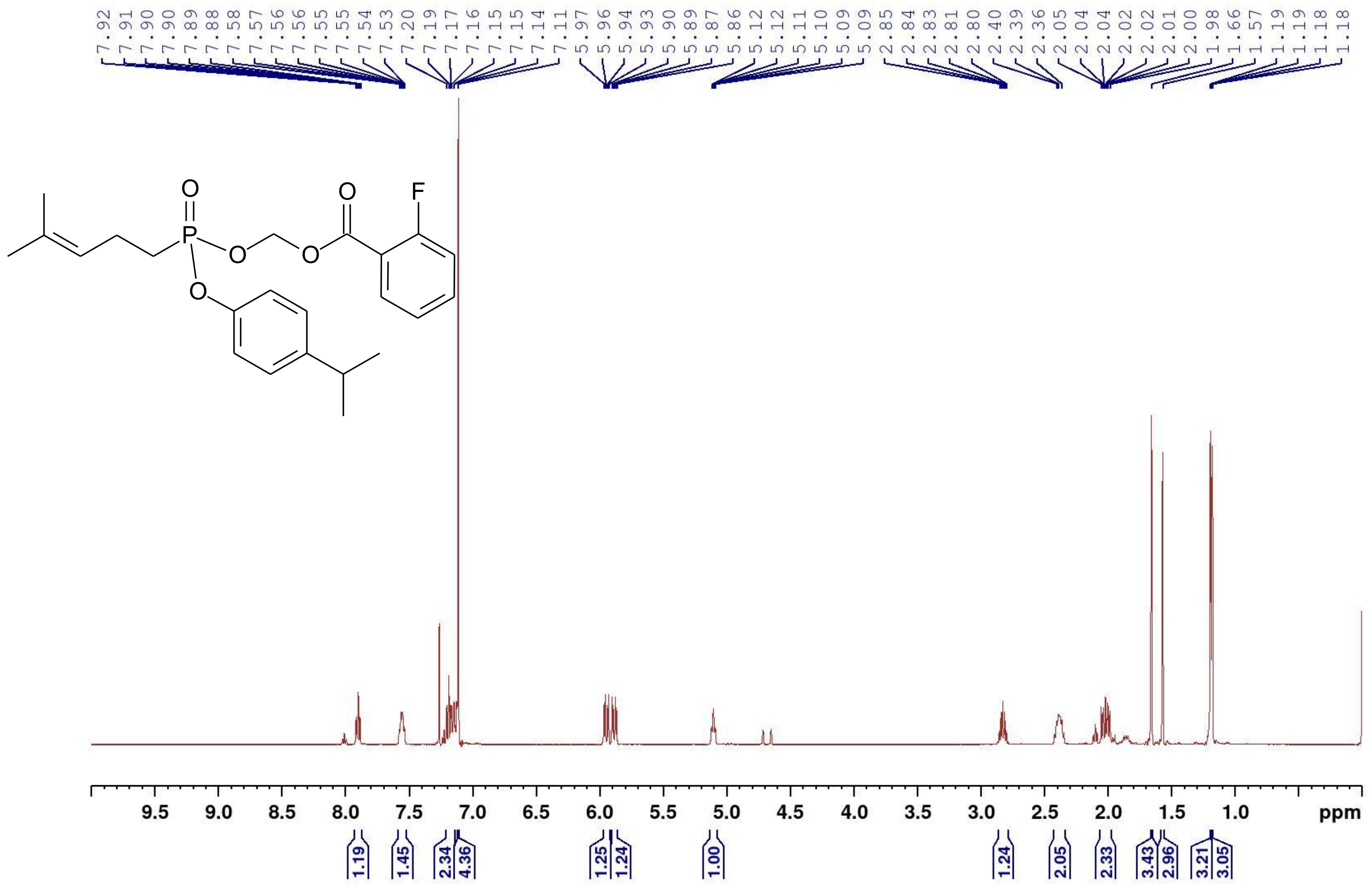

^{1}H NMR Spectrum of Compound **7a** ($CDCl_3$, 500 MHz)

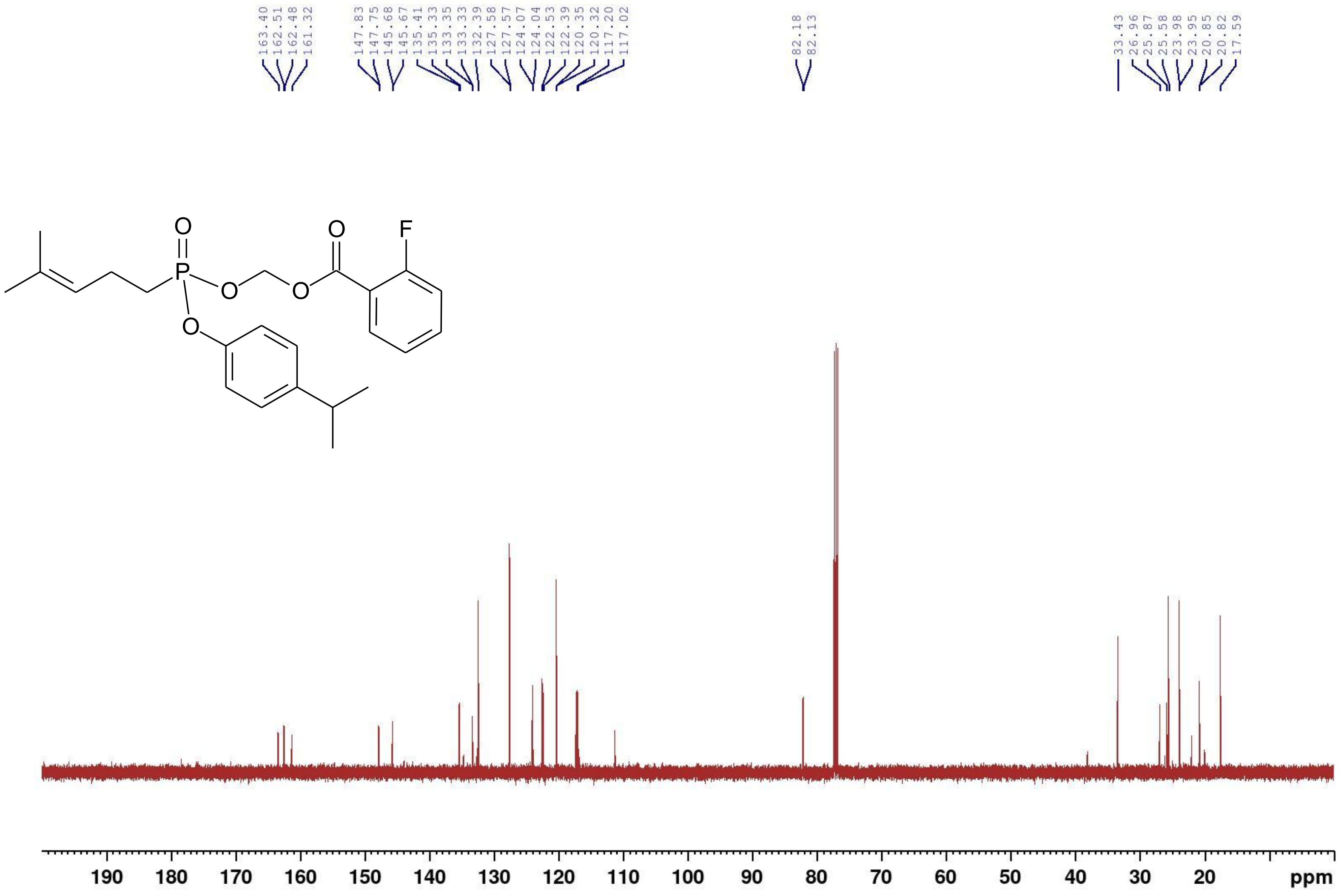

^{13}C NMR Spectrum of Compound **7a** ($CDCl_3$, 126 MHz)

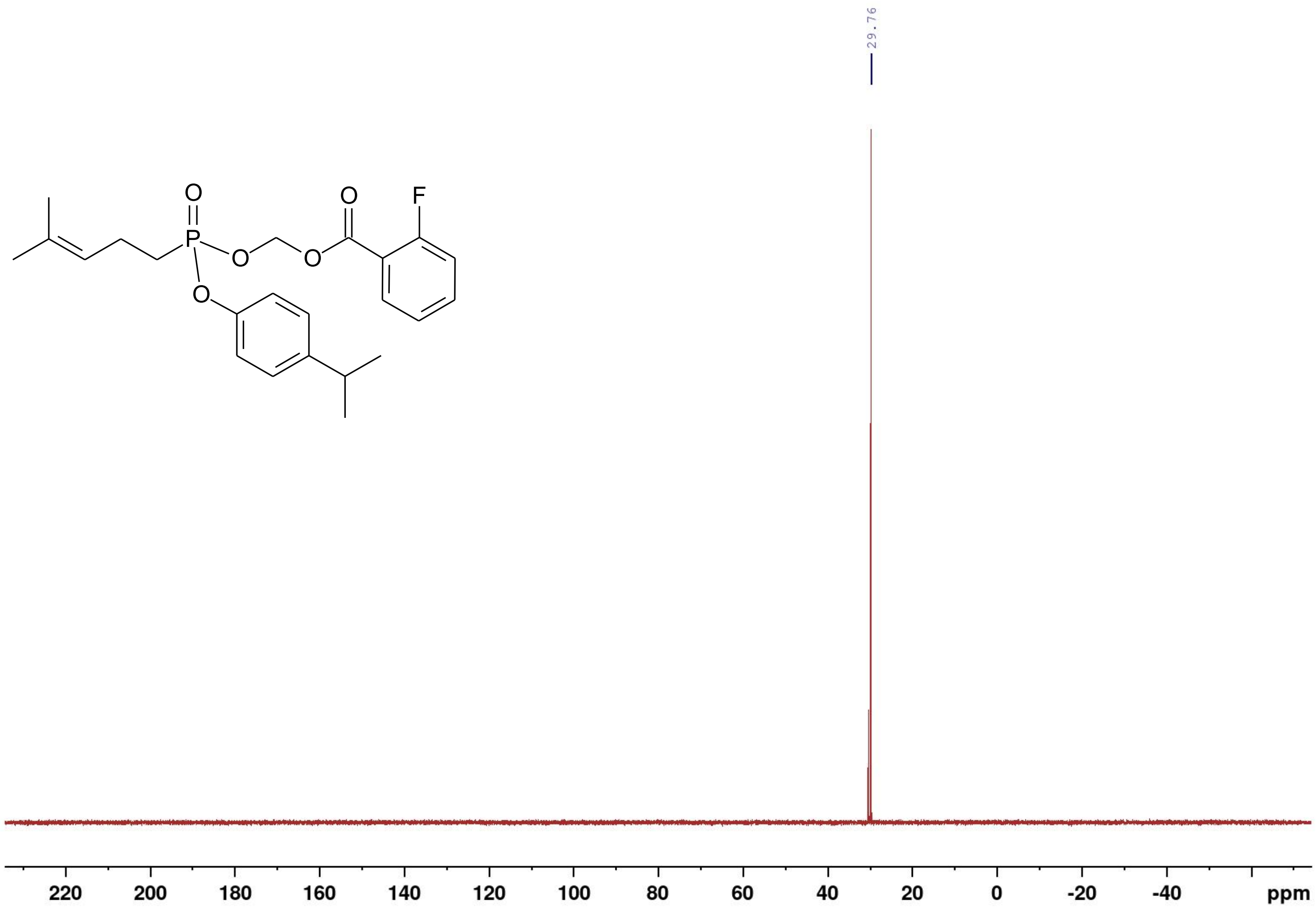

^{31}P NMR Spectrum of Compound **7a** ($CDCl_3$, 203 MHz)

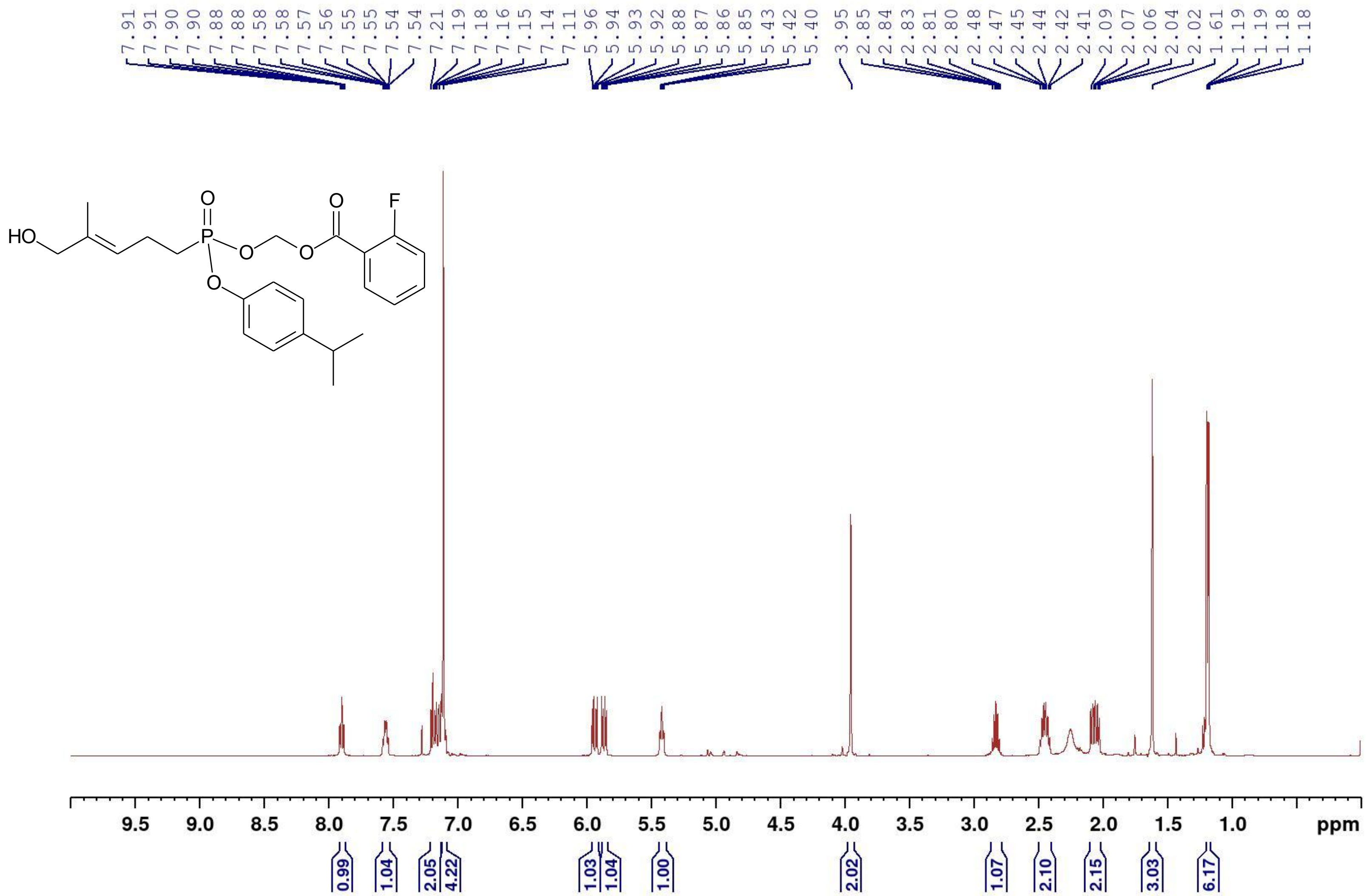

^{1}H NMR Spectrum of Compound **8a** ($CDCl_3$, 500 MHz)

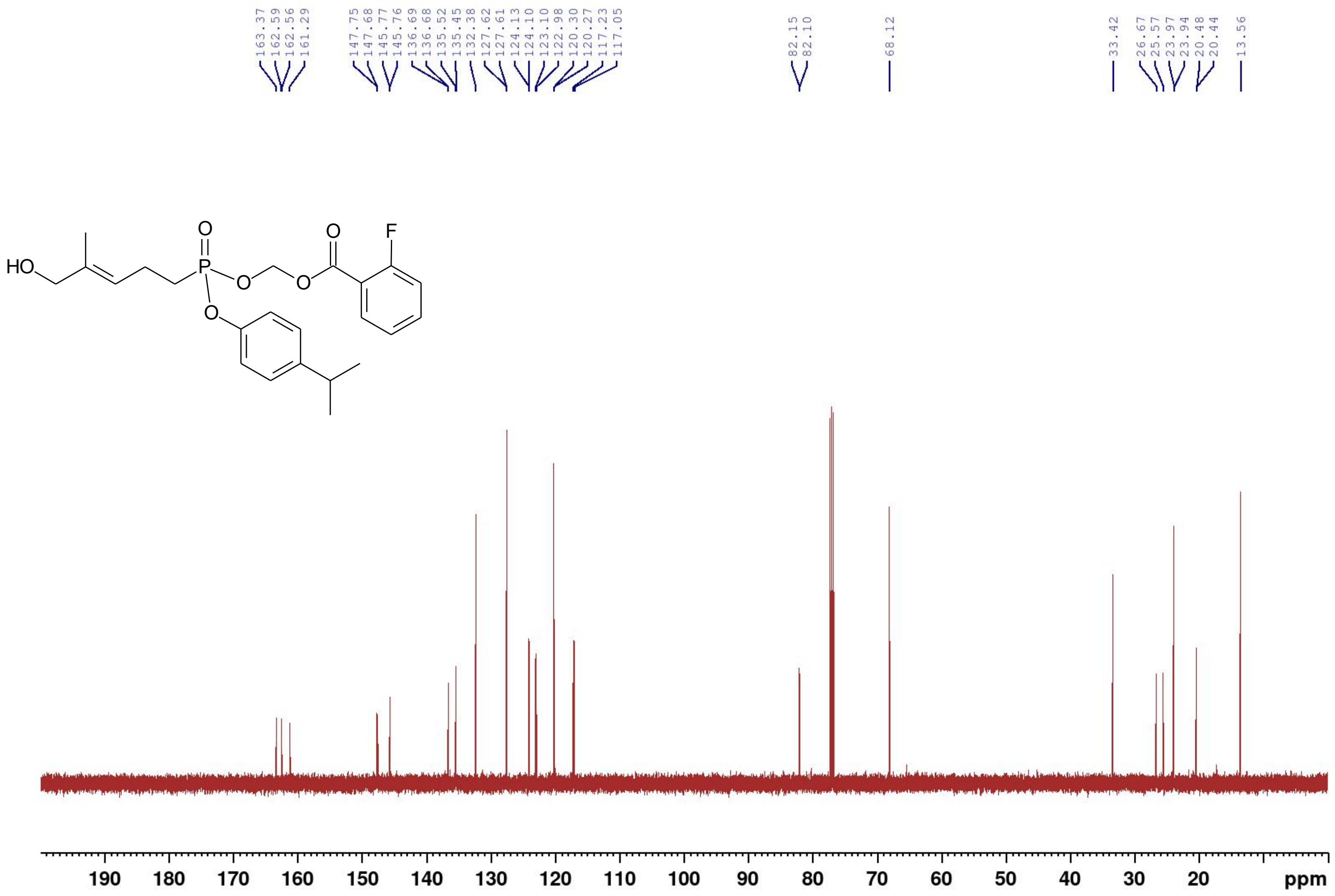

^{13}C NMR Spectrum of Compound **8a** ($CDCl_3$, 126 MHz)

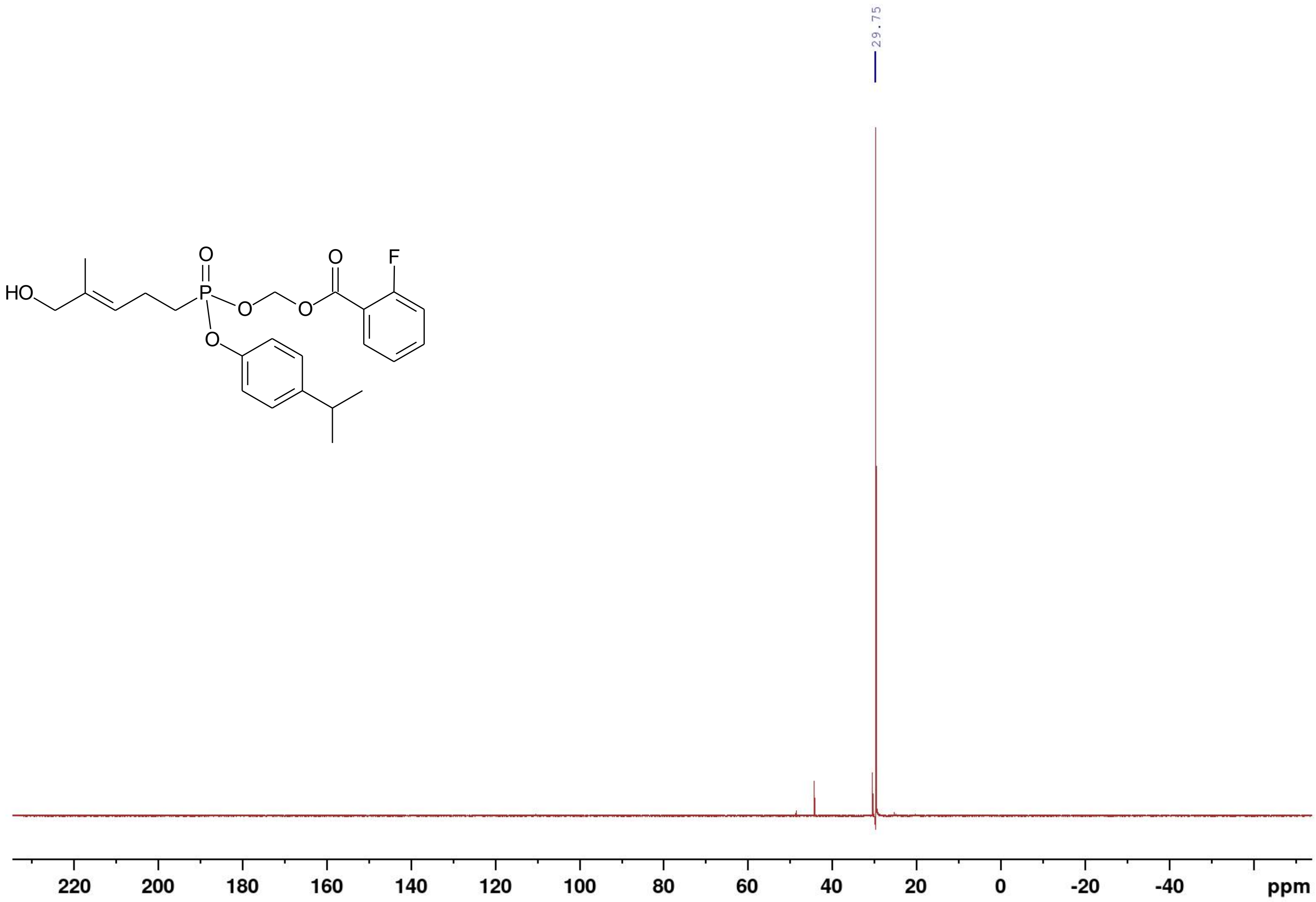

^{31}P NMR Spectrum of Compound **8a** ($CDCl_3$, 203 MHz)

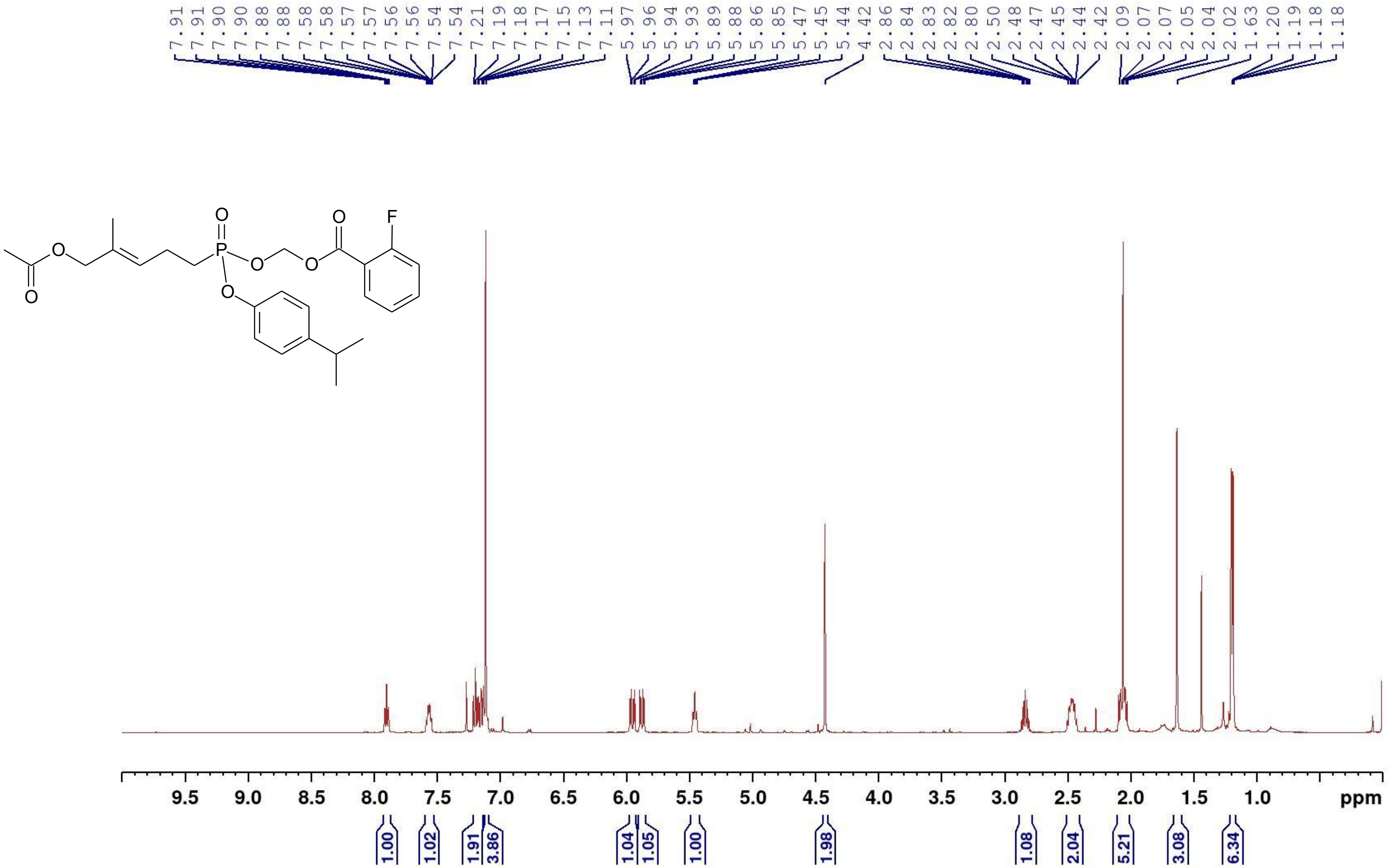

^{1}H NMR Spectrum of Compound **9a** ($CDCl_3$, 500 MHz)

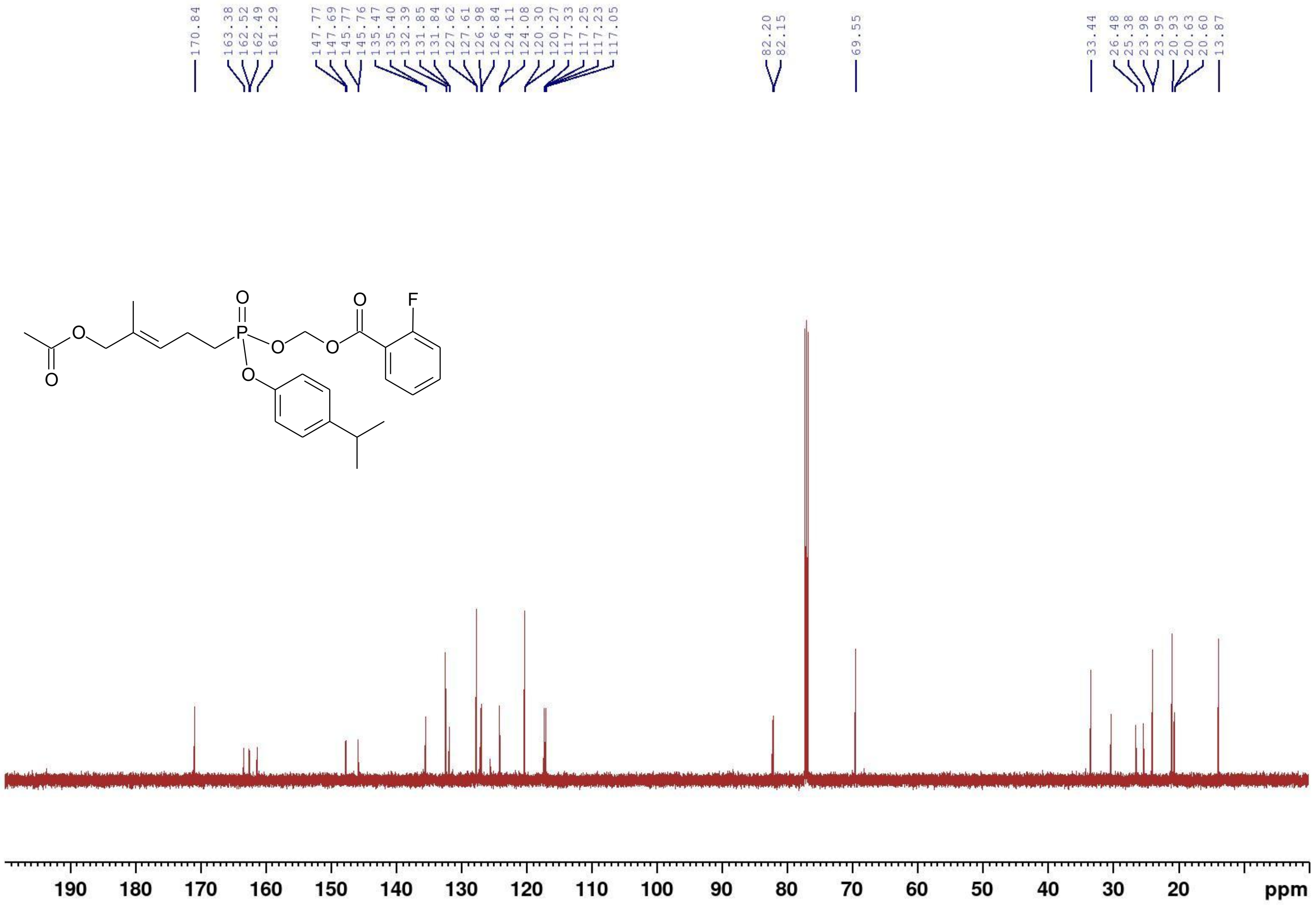

^{13}C NMR Spectrum of Compound **9a** ($CDCl_3$, 126 MHz)

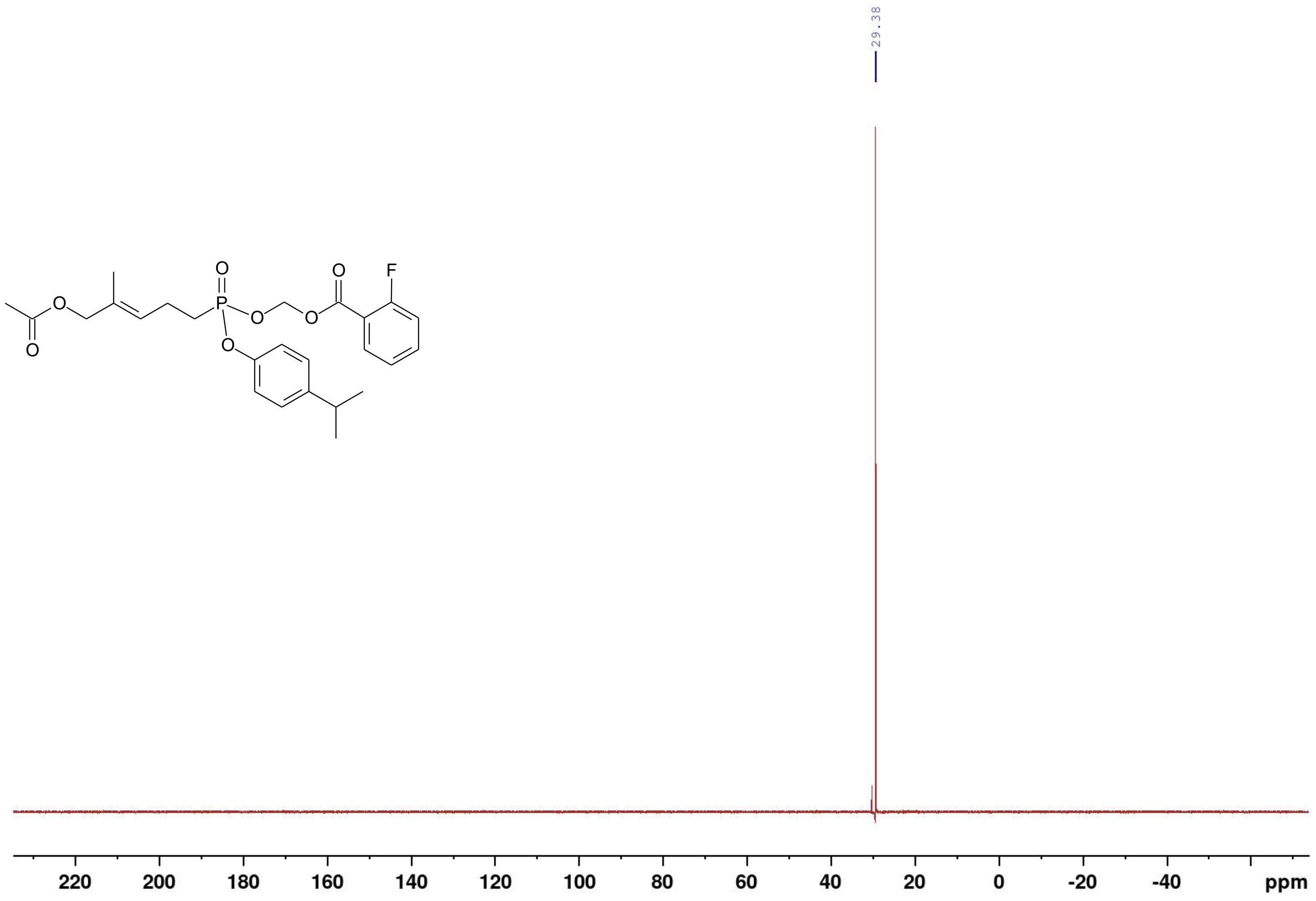

^{31}P NMR Spectrum of Compound **9a** ($CDCl_3$, 203 MHz)

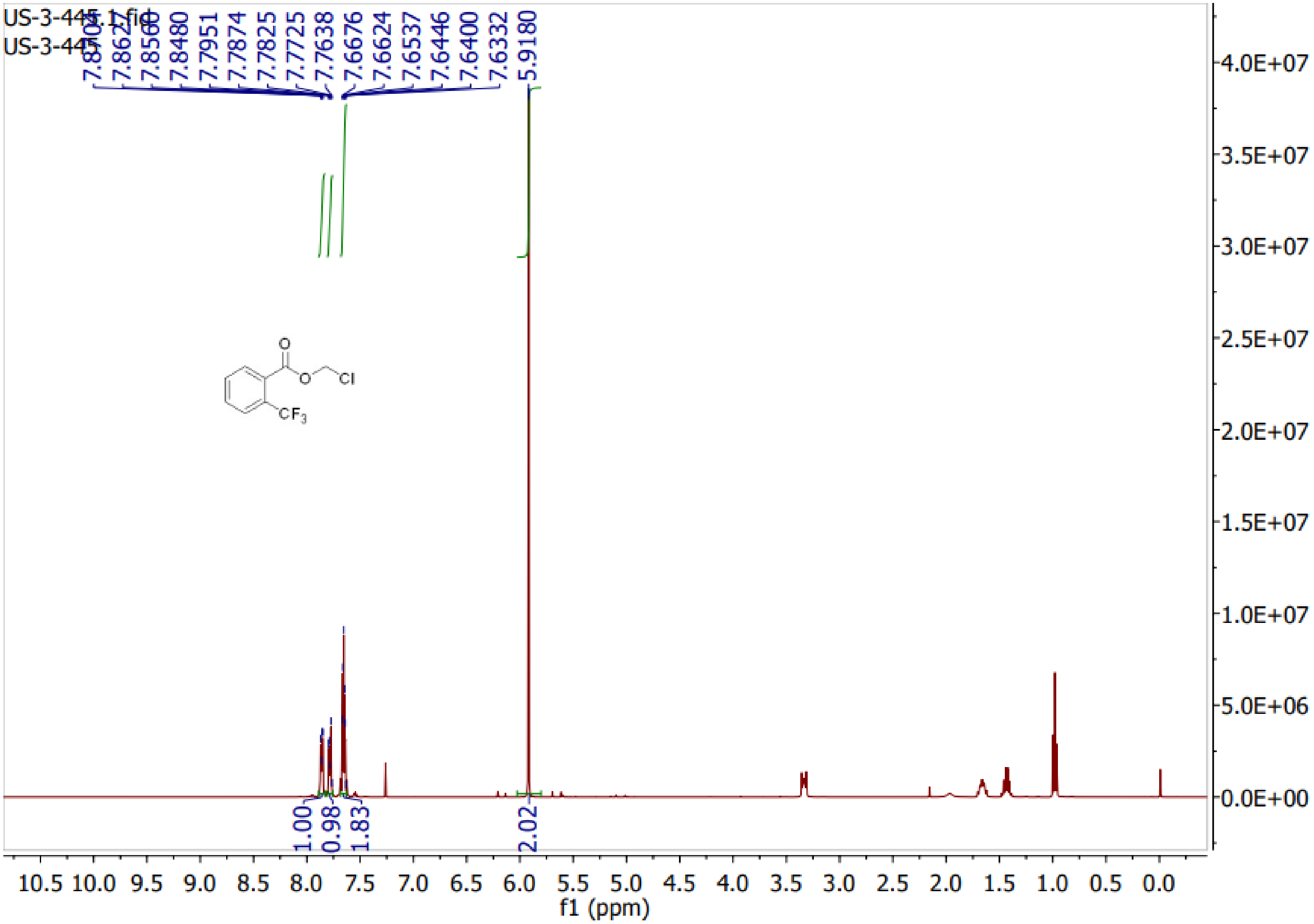

^{1}H NMR Spectrum of Compound **5b** ($CDCl_3$, 400 MHz)

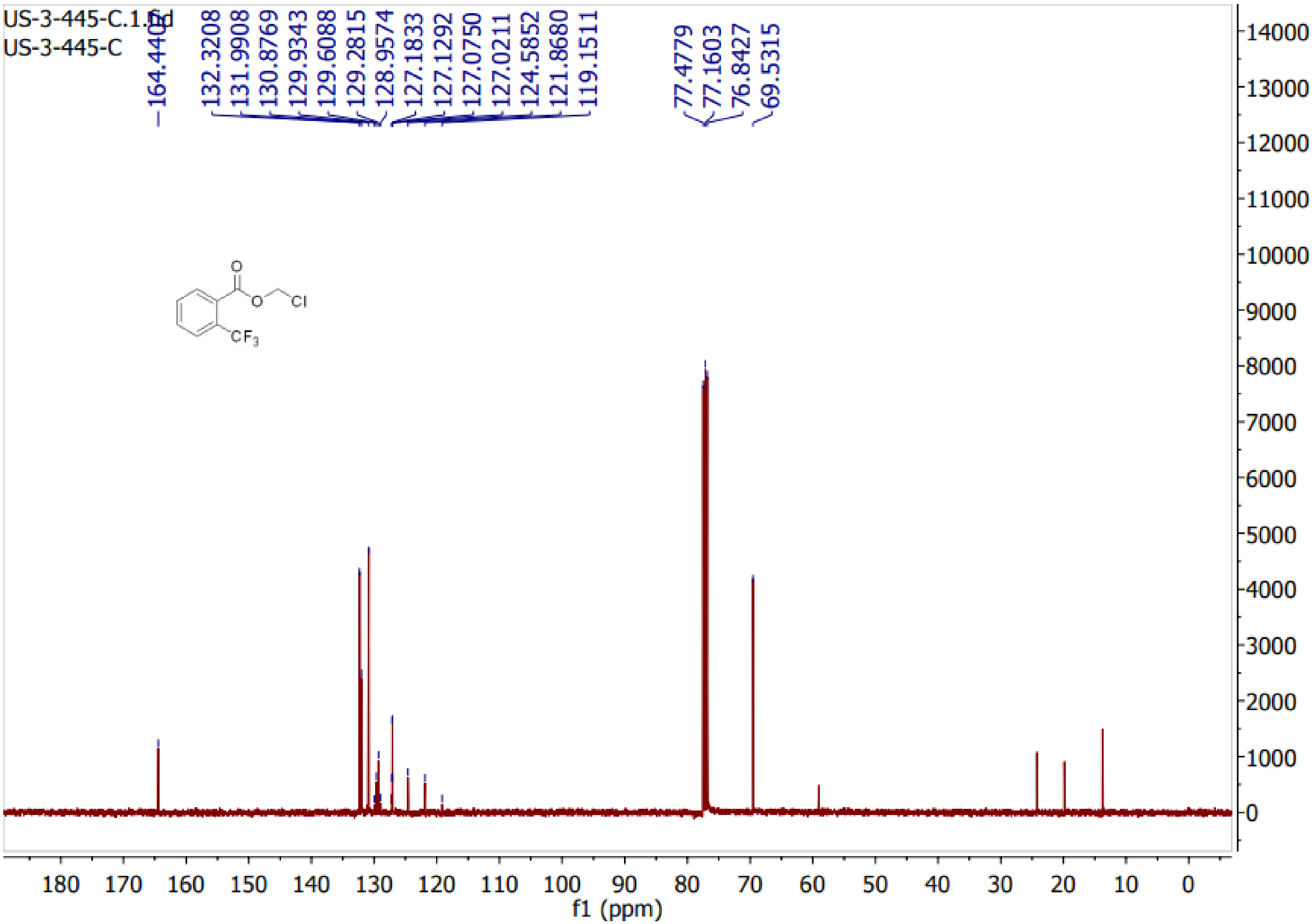

^{13}C NMR Spectrum of Compound **5b** ($CDCl_3$, 101 MHz)

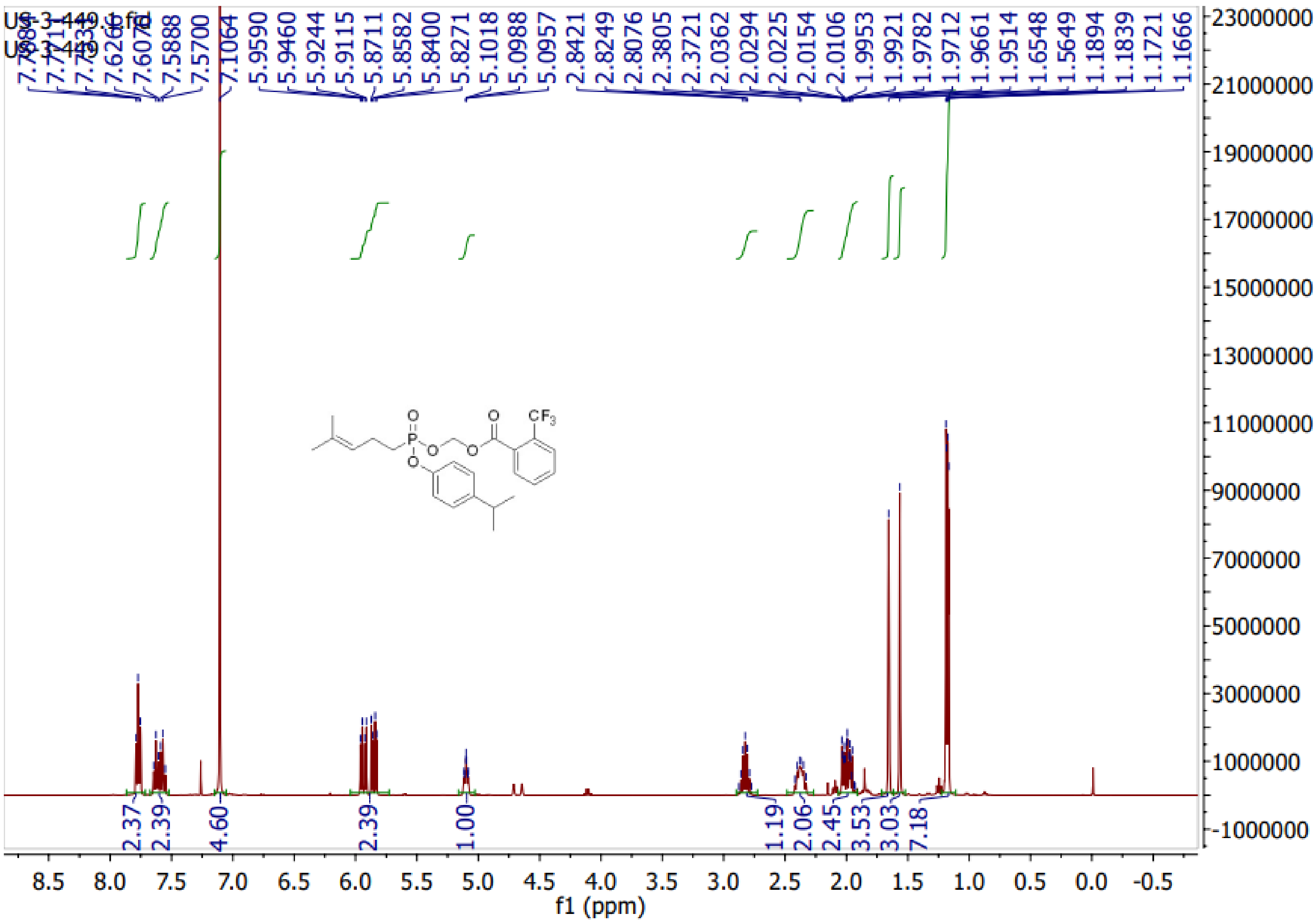

^{1}H NMR Spectrum of Compound **7b** ($CDCl_3$, 400 MHz)

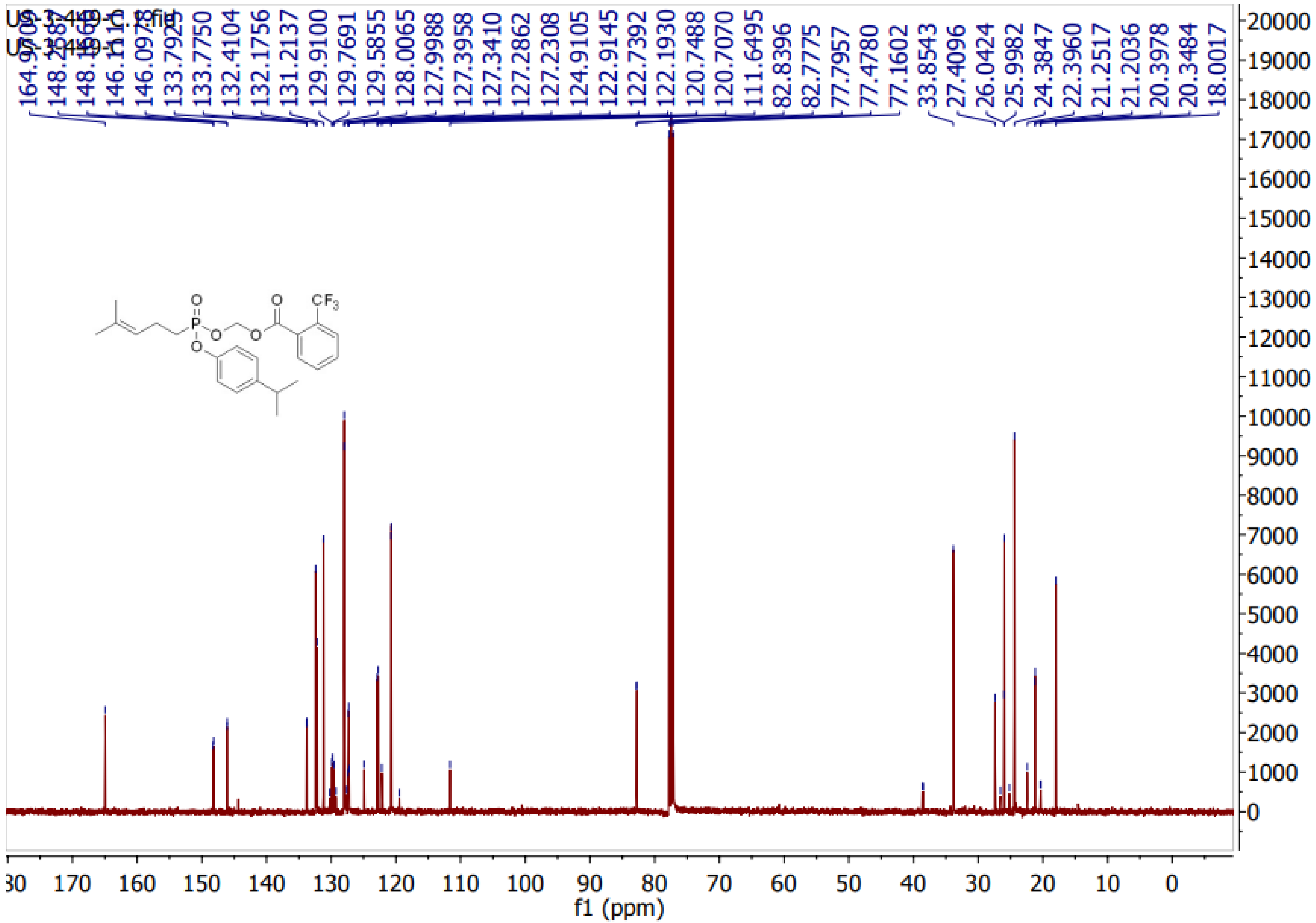

^{13}C NMR Spectrum of Compound **7b** ($CDCl_3$, 101 MHz)

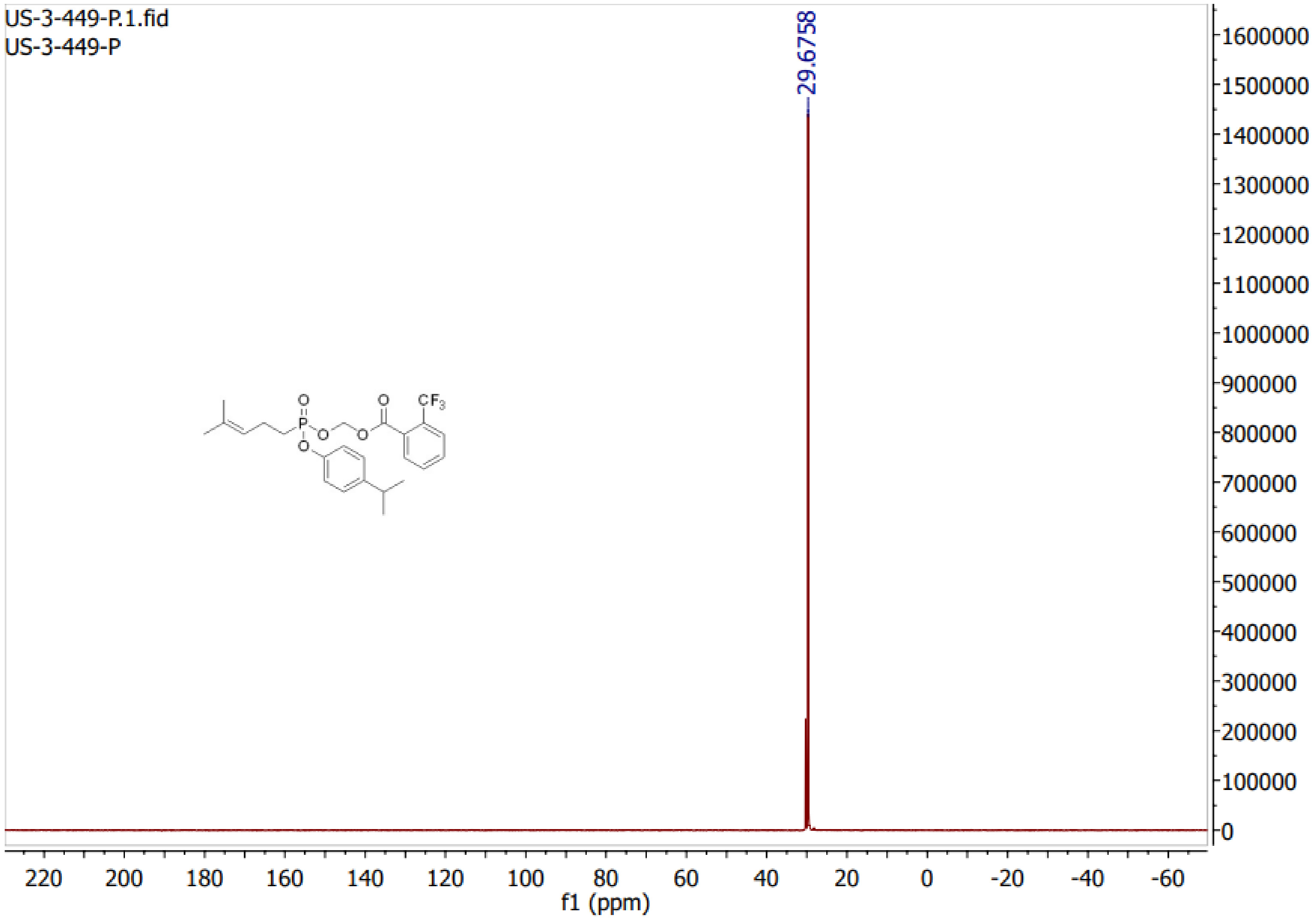

[1]P NMR Spectrum of Compound **7b** ($CDCl_3$, 203 MHz)

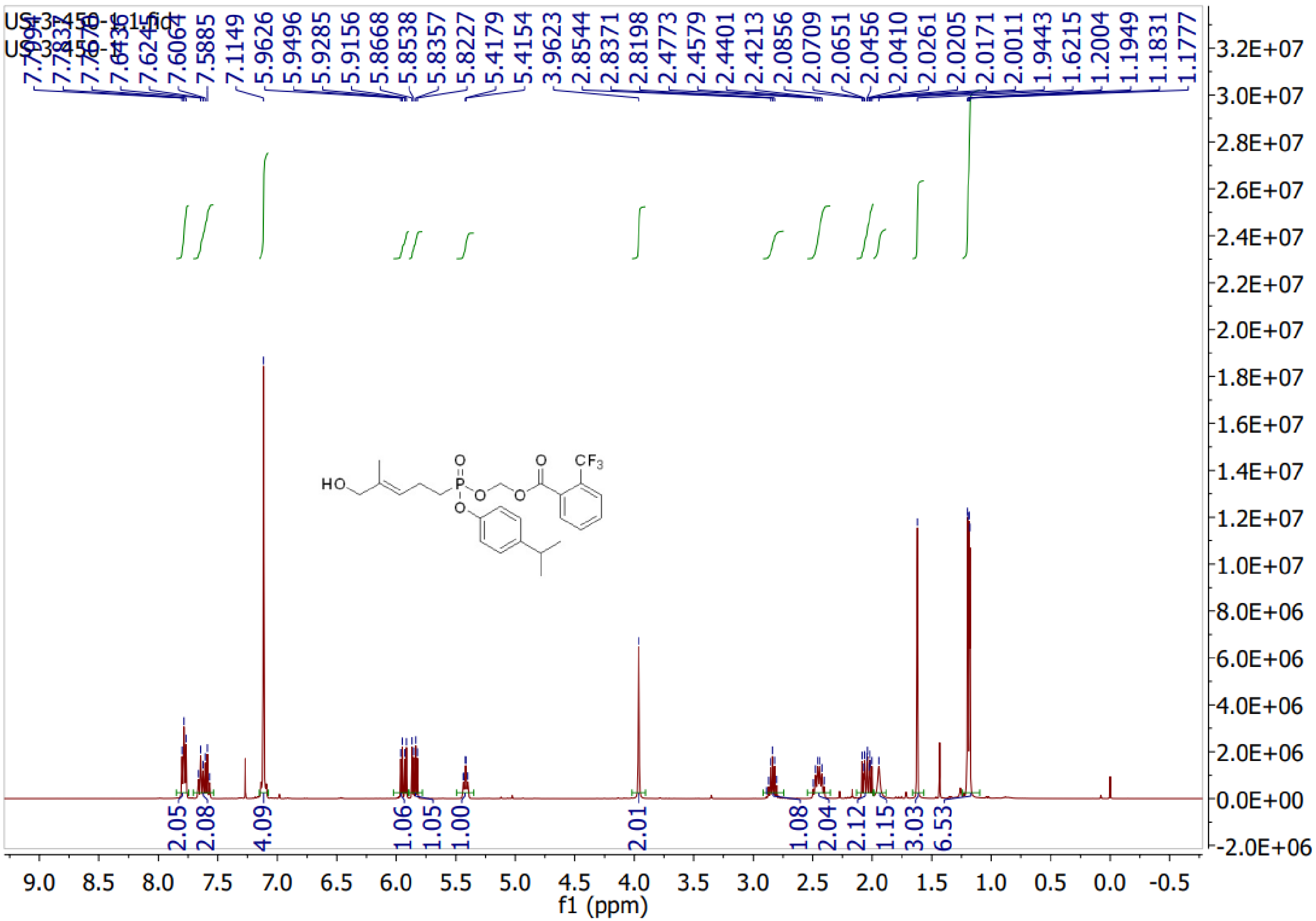

^{1}H NMR Spectrum of Compound **8b** ($CDCl_3$, 400 MHz)

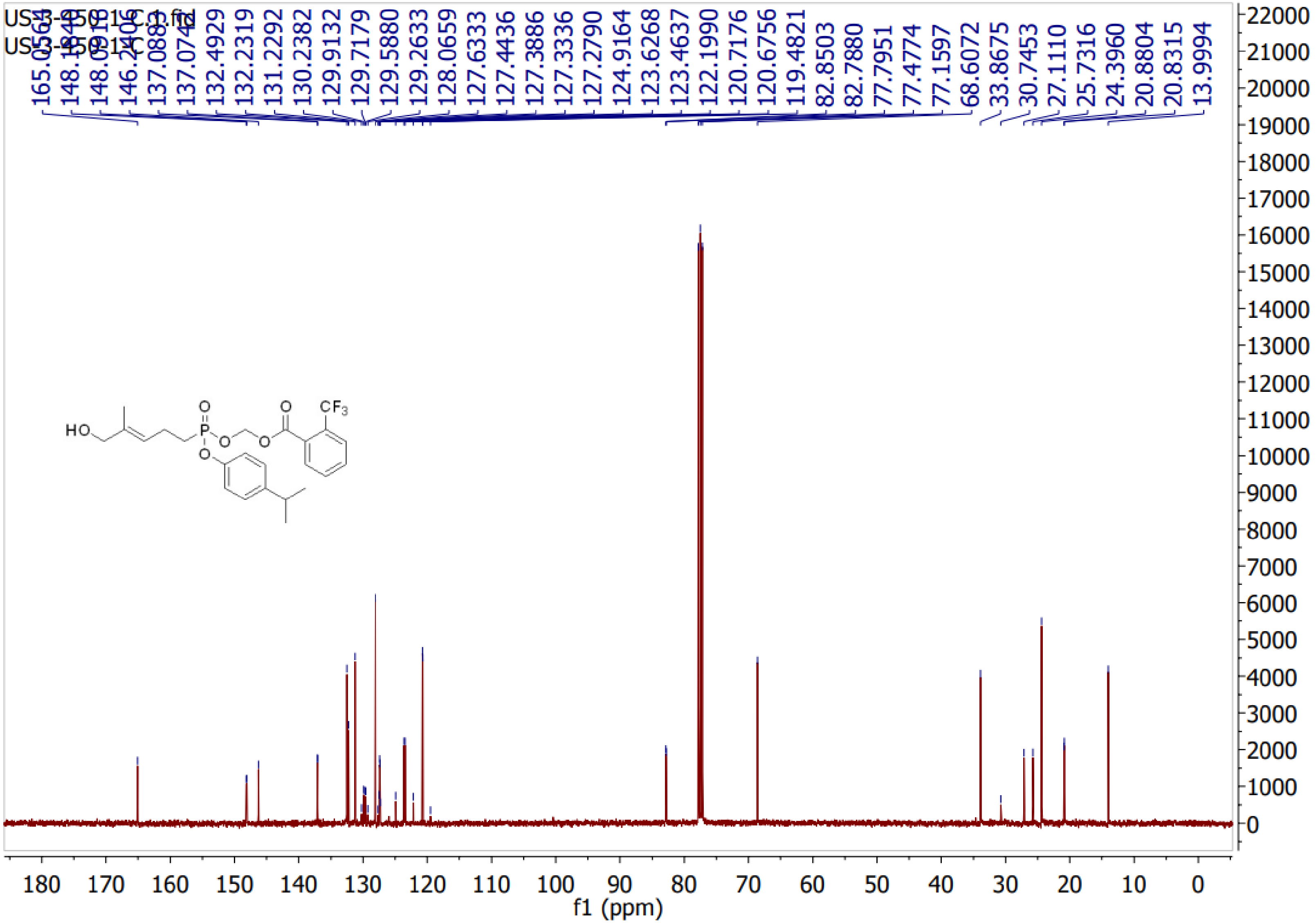

^{13}C NMR Spectrum of Compound **8b** ($CDCl_3$, 101 MHz)

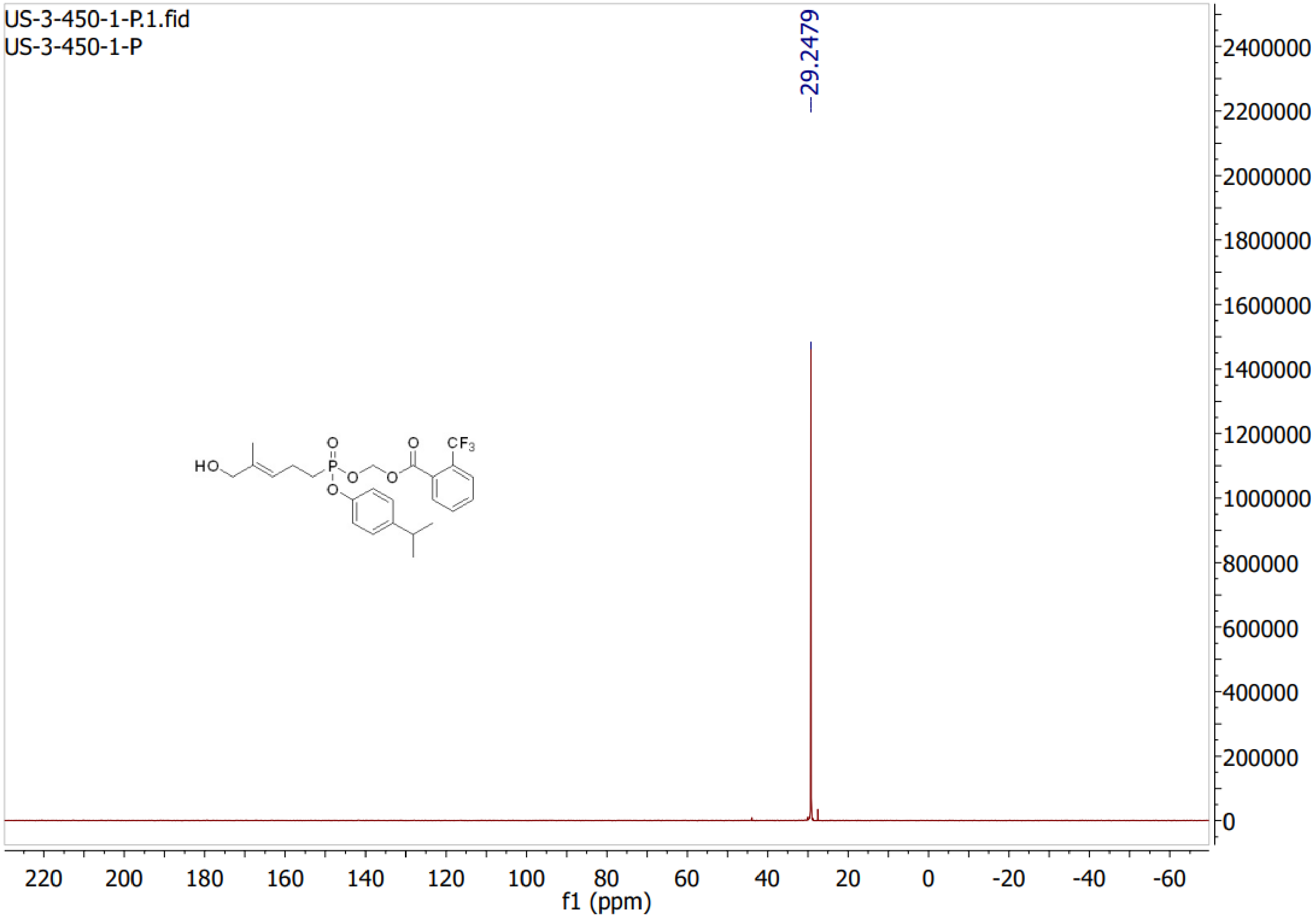

[1]P NMR Spectrum of Compound **8b** ($CDCl_3$, 203 MHz)

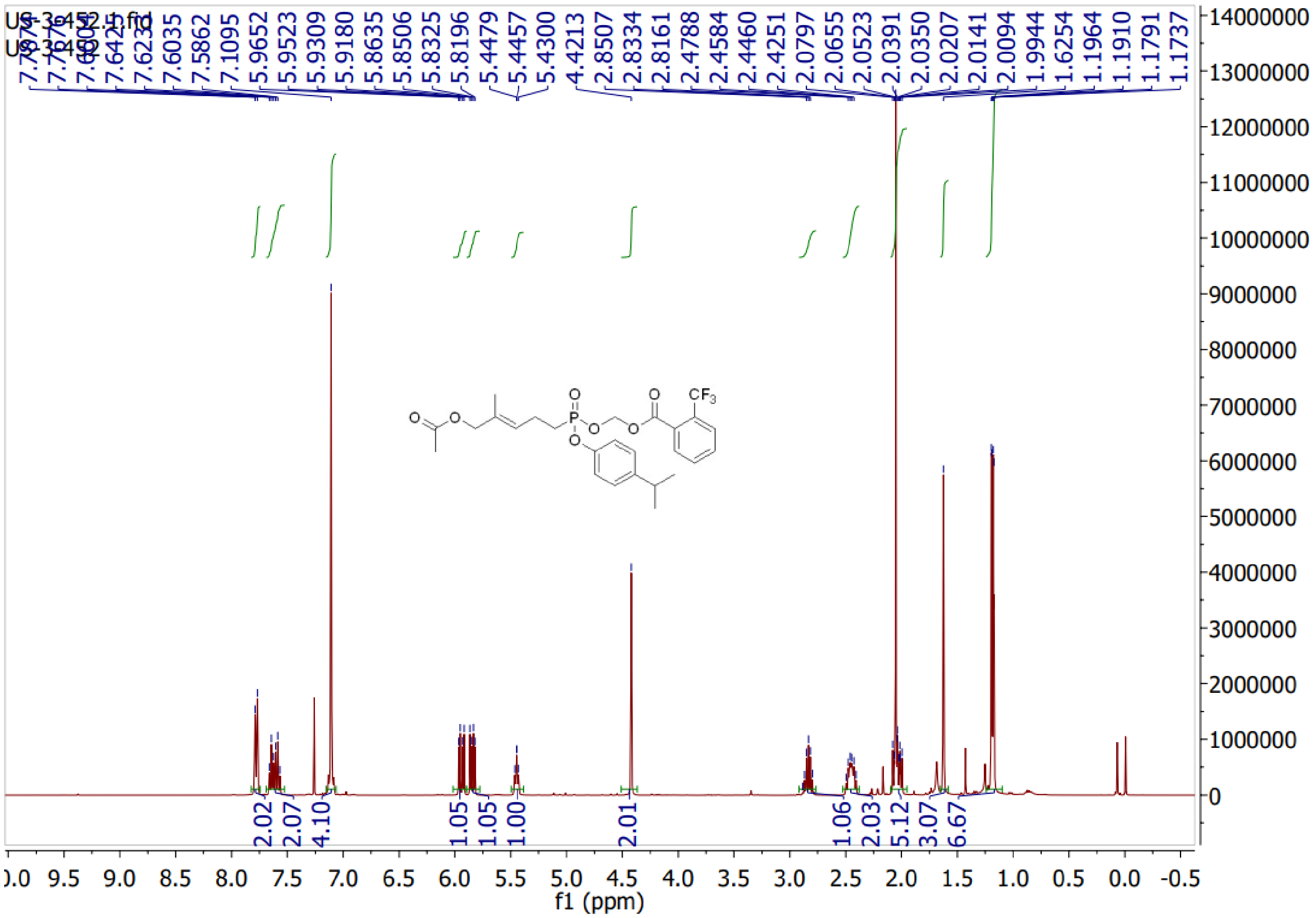

^{1}H NMR Spectrum of Compound **9b** ($CDCl_3$, 400 MHz)

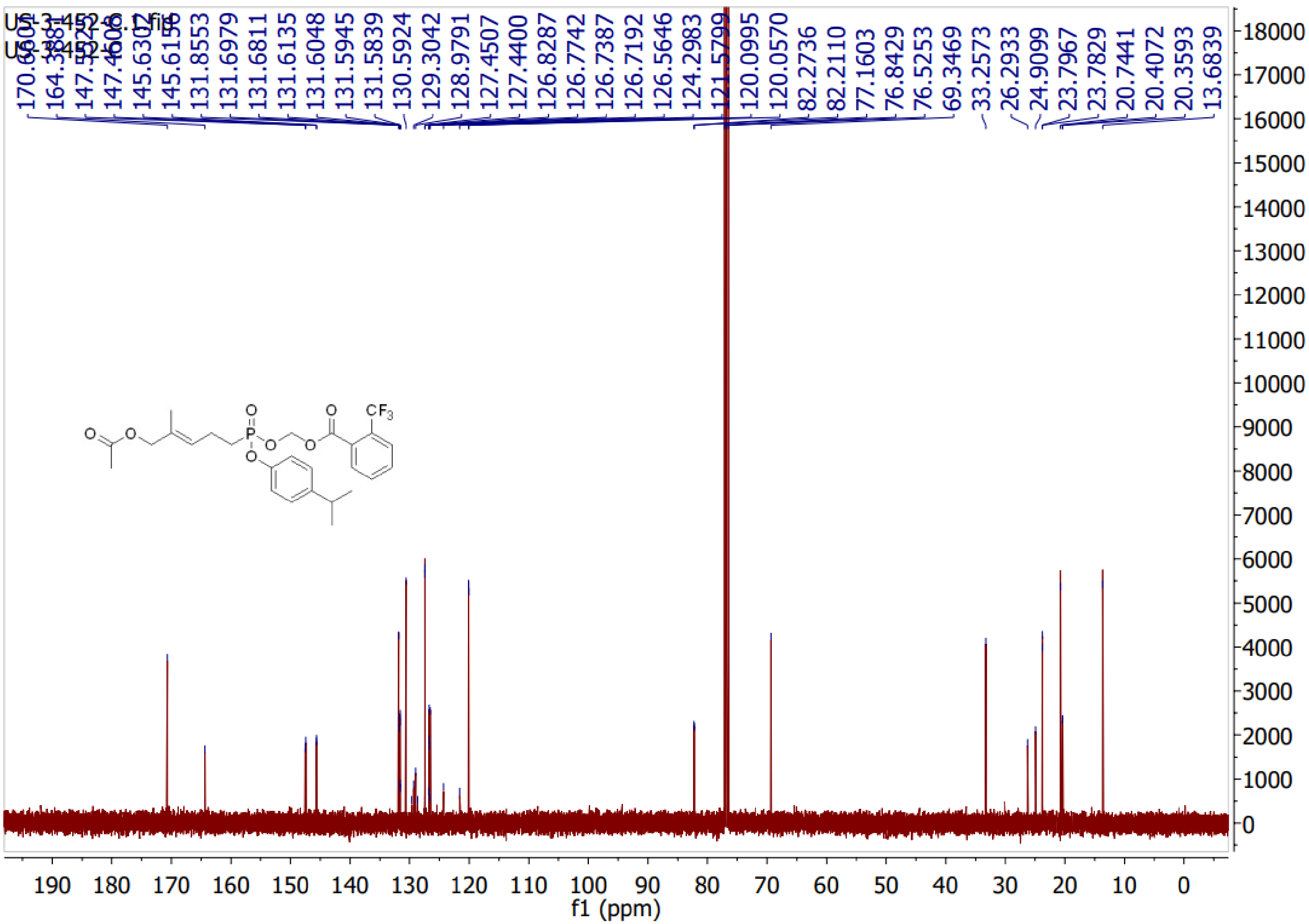

^{13}C NMR Spectrum of Compound **9b** ($CDCl_3$, 101 MHz)

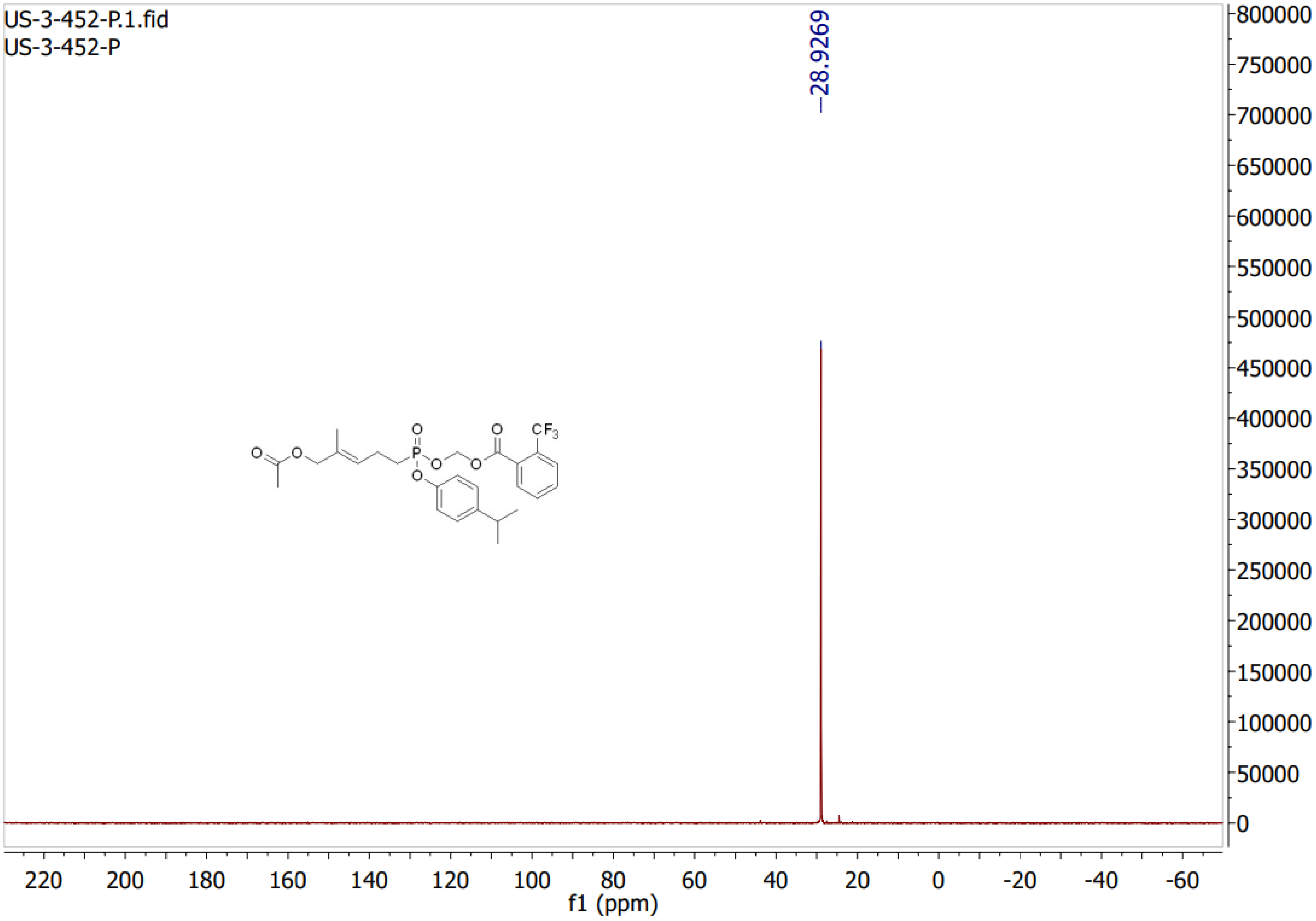

^{31}P NMR Spectrum of Compound **9b** ($CDCl_3$, 162 MHz)

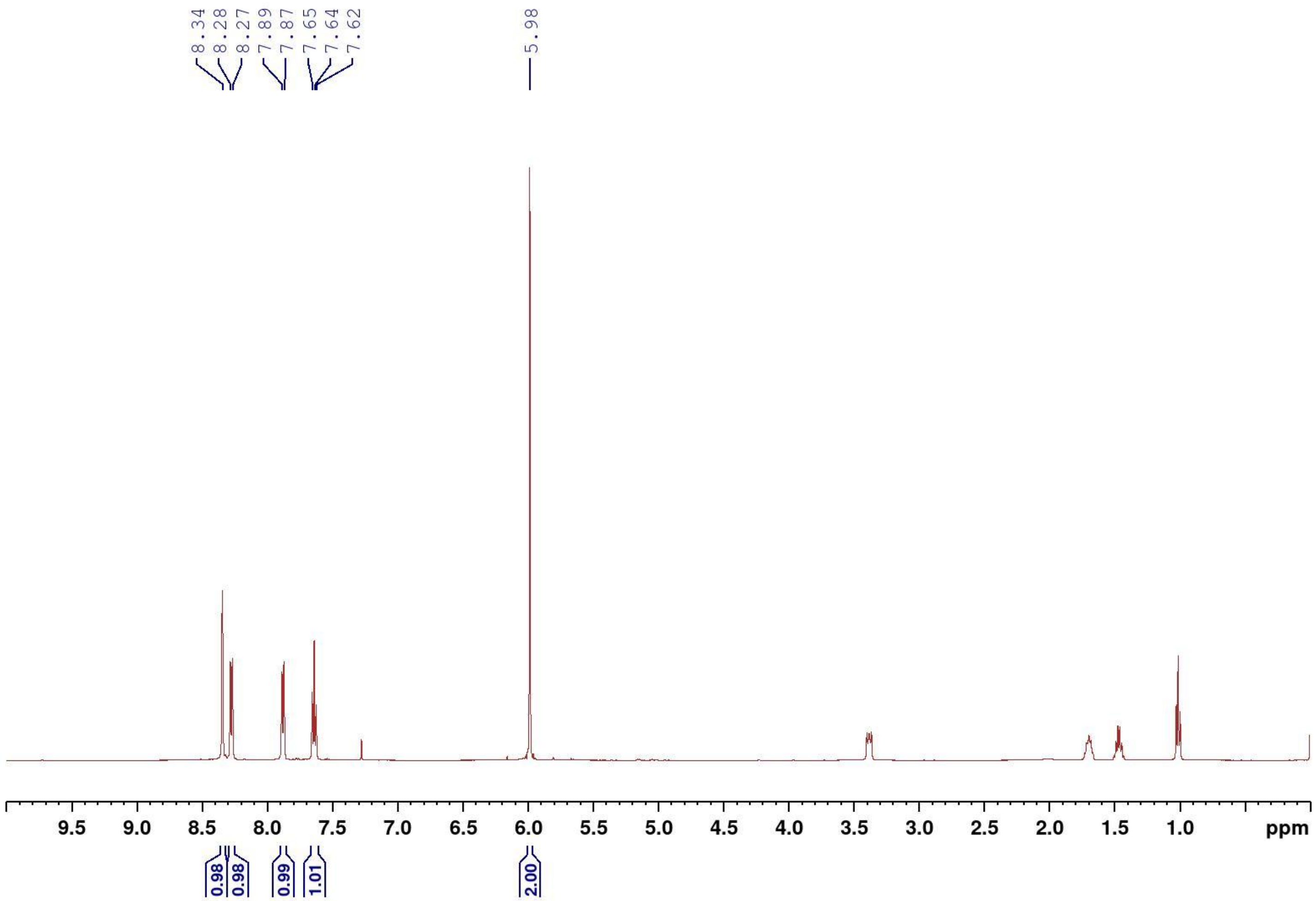

^{1}H NMR Spectrum of Compound **5c** ($CDCl_3$, 500 MHz)

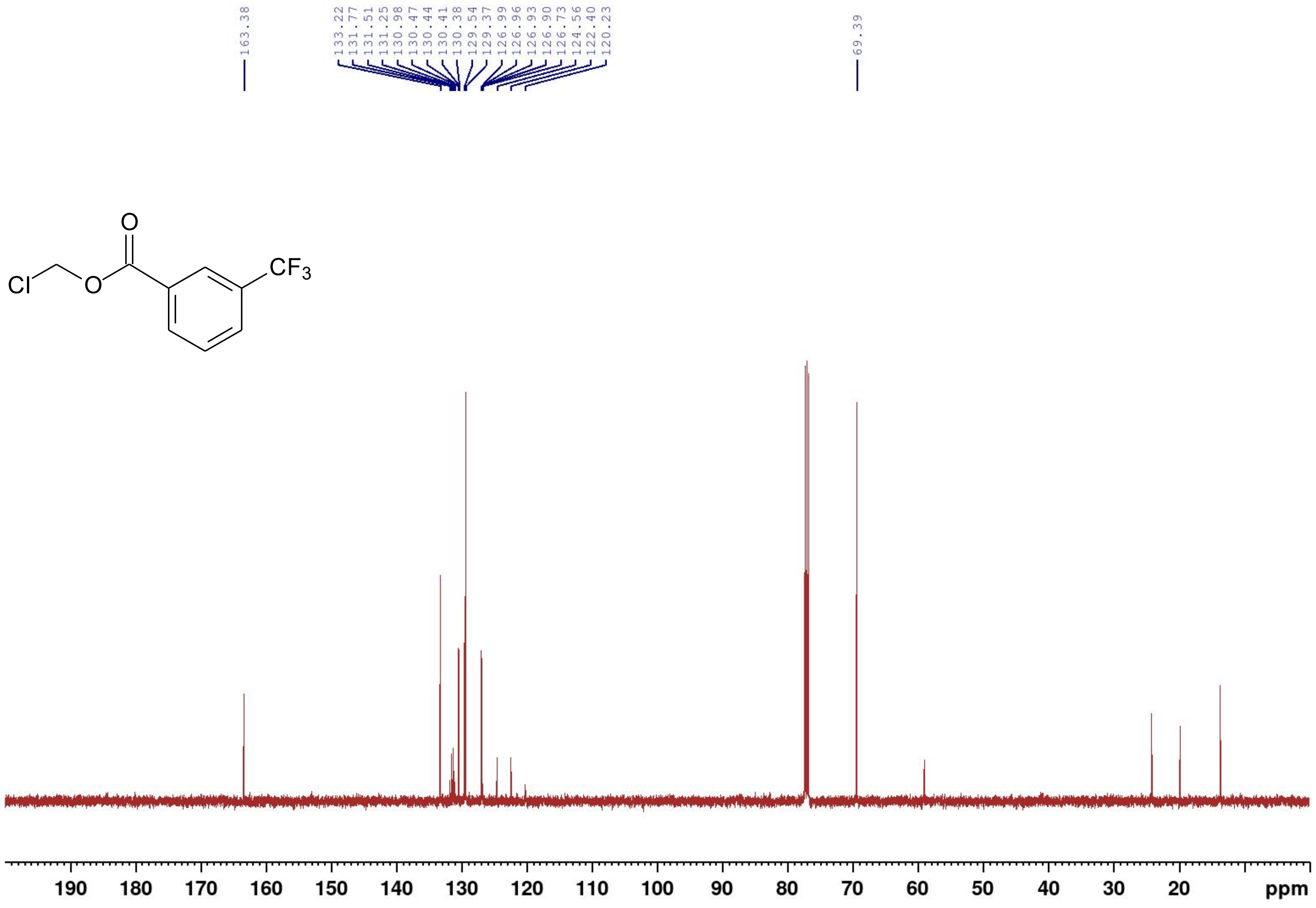

^{13}C NMR Spectrum of Compound **5c** ($CDCl_3$, 126 MHz)

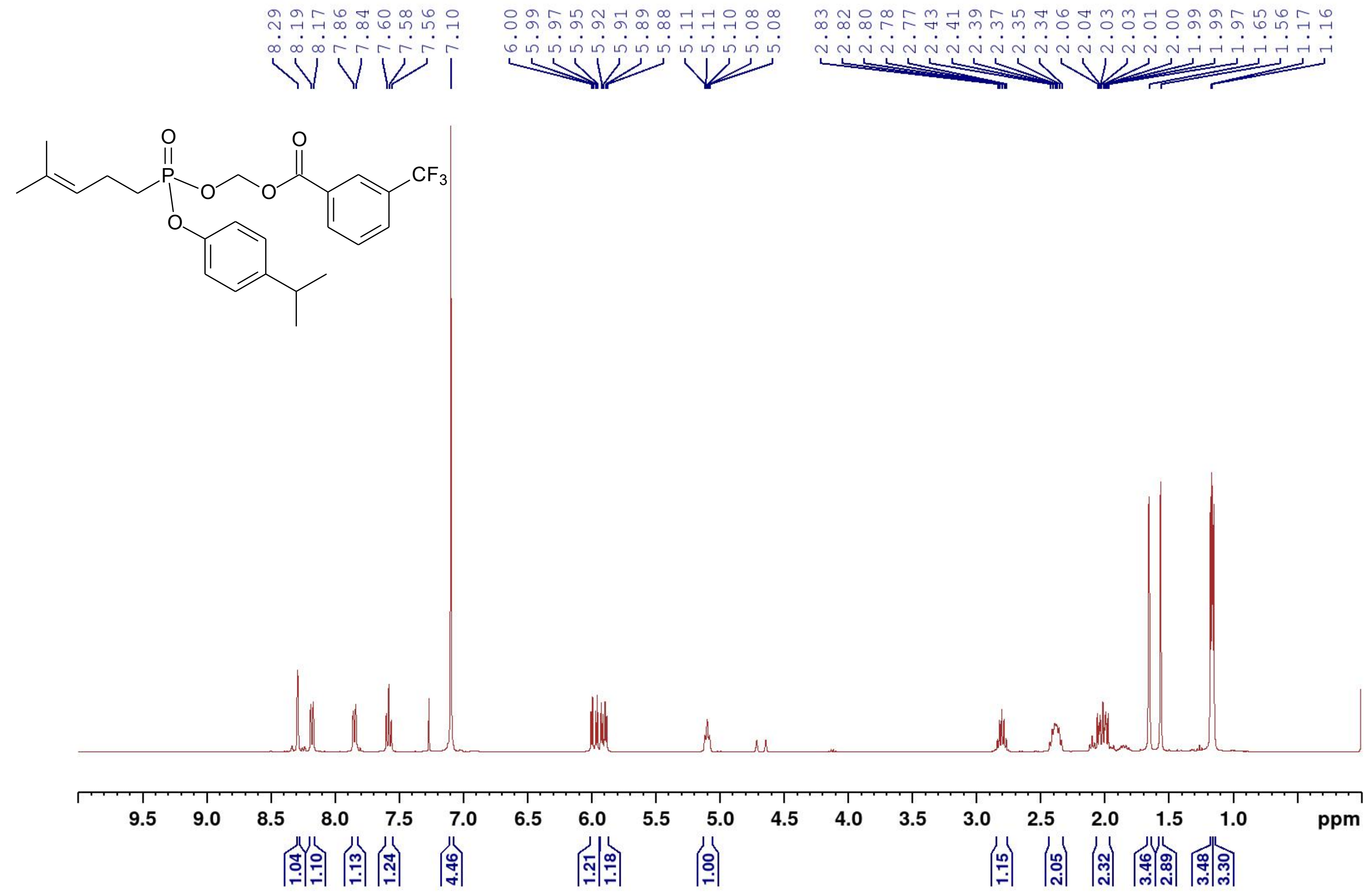

^{1}H NMR Spectrum of Compound **7c** ($CDCl_3$, 400 MHz)

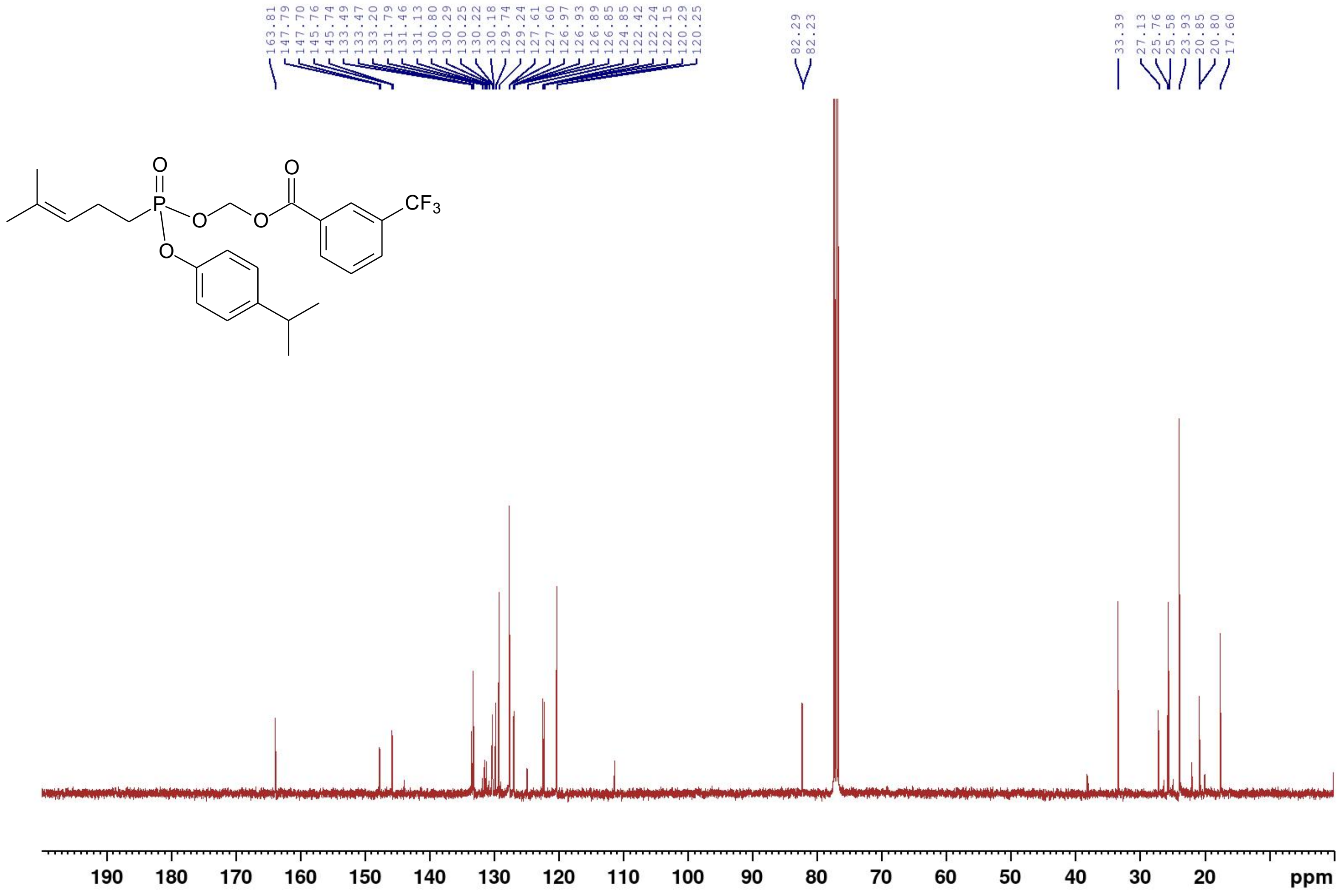

^{13}C NMR Spectrum of Compound **7c** ($CDCl_3$, 101 MHz)

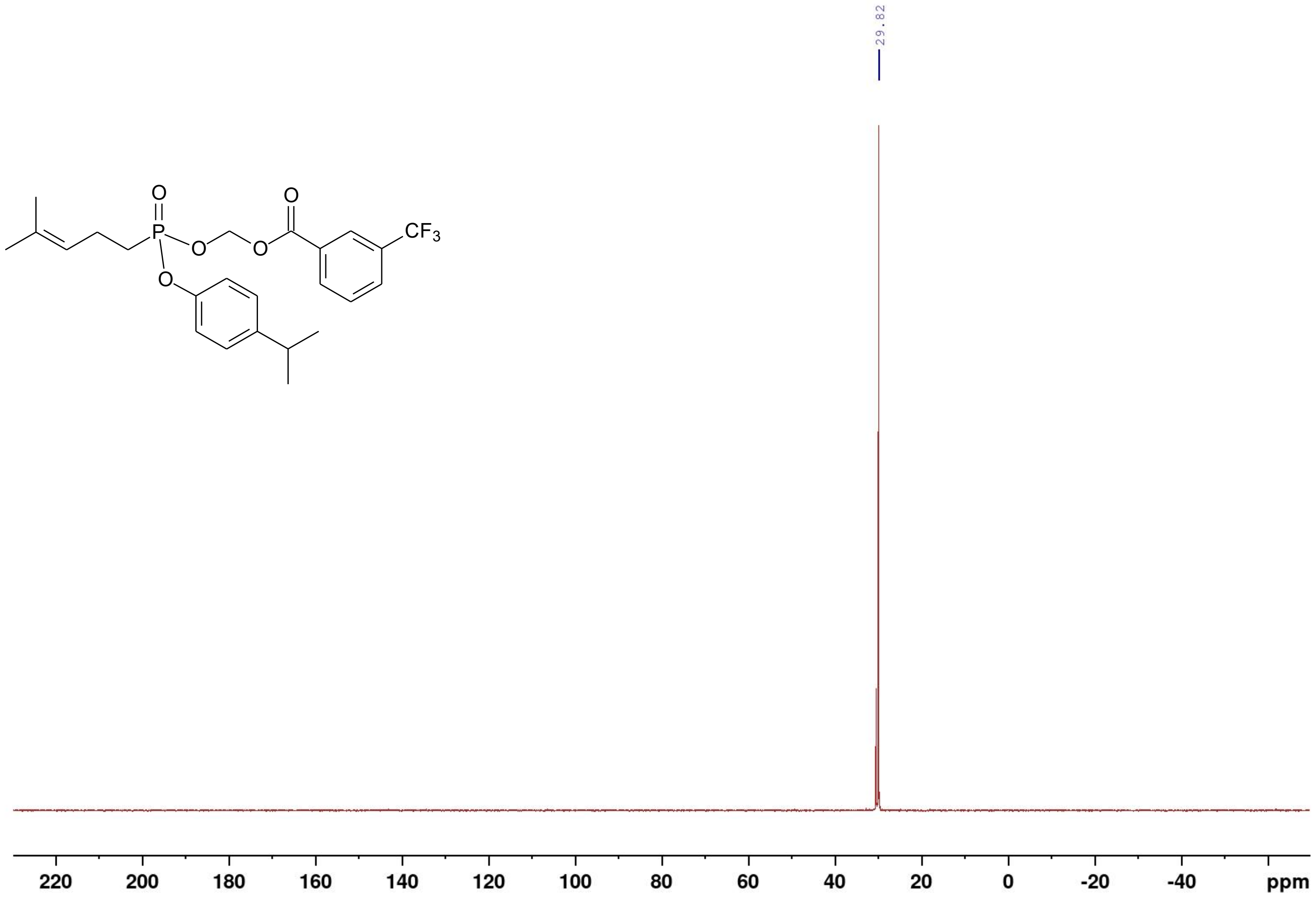

^{31}P NMR Spectrum of Compound **7c** ($CDCl_3$, 162 MHz)

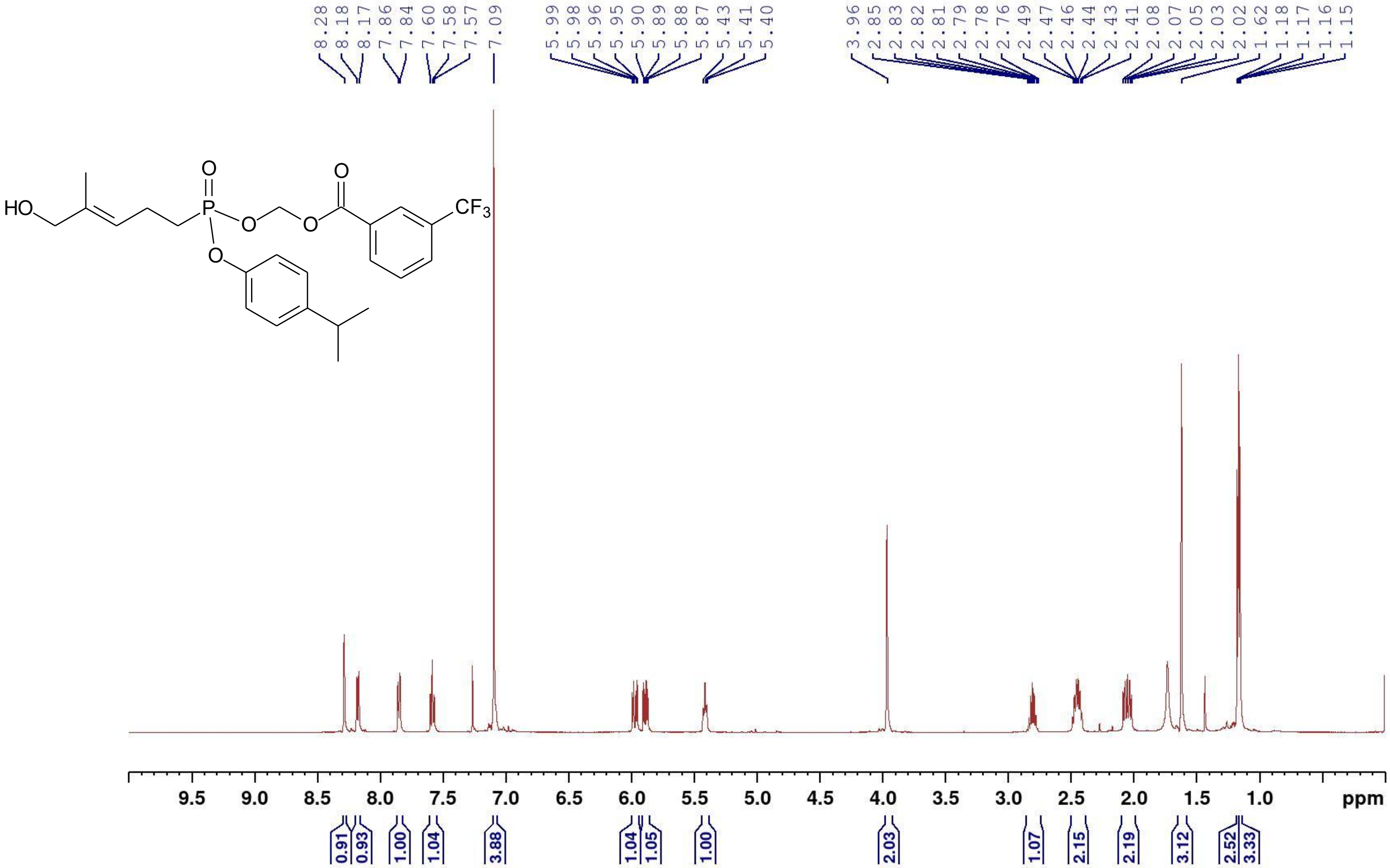

^{1}H NMR Spectrum of Compound **8c** ($CDCl_3$, 500 MHz)

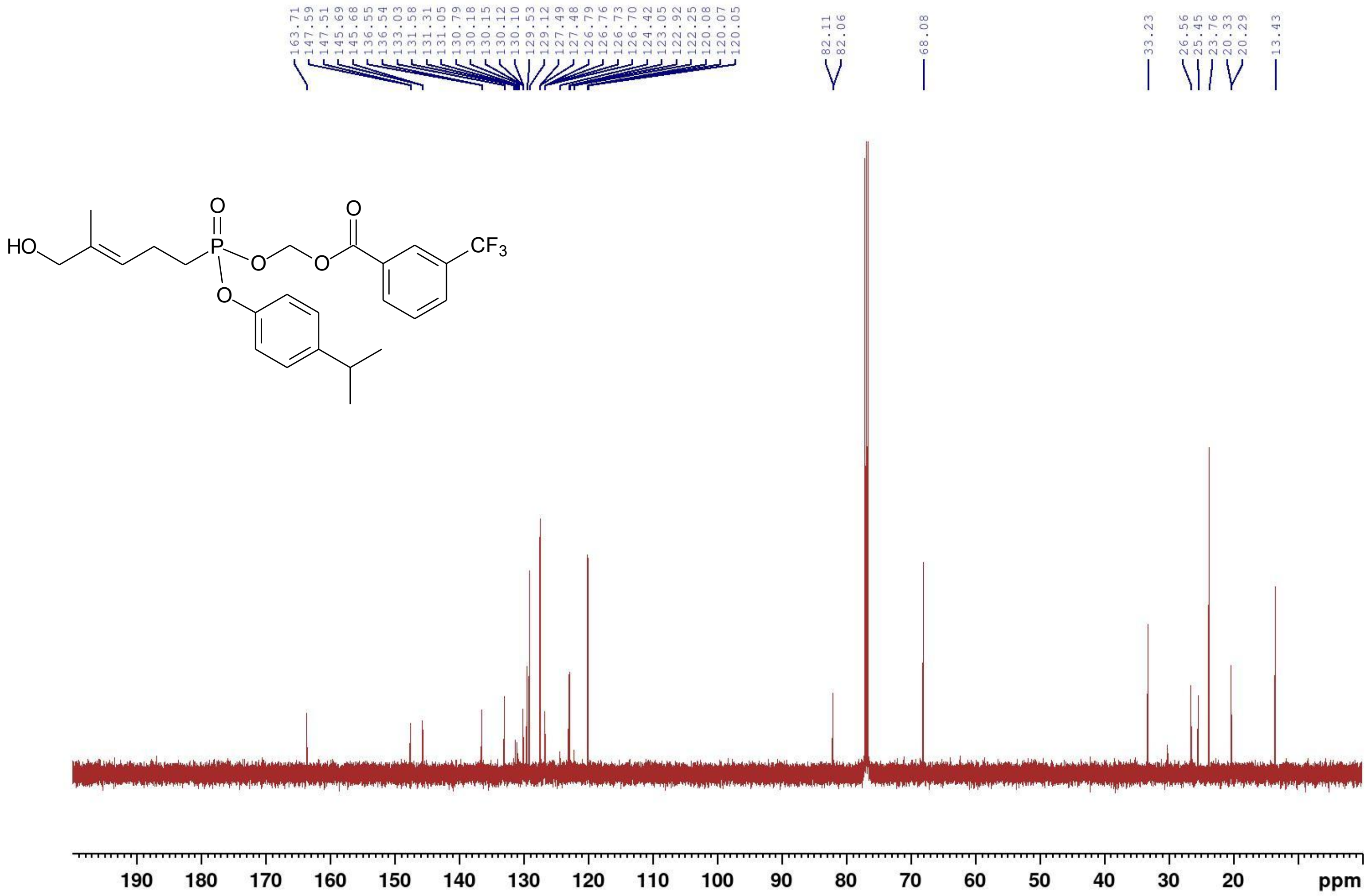

^{13}C NMR Spectrum of Compound **8c** ($CDCl_3$, 126 MHz)

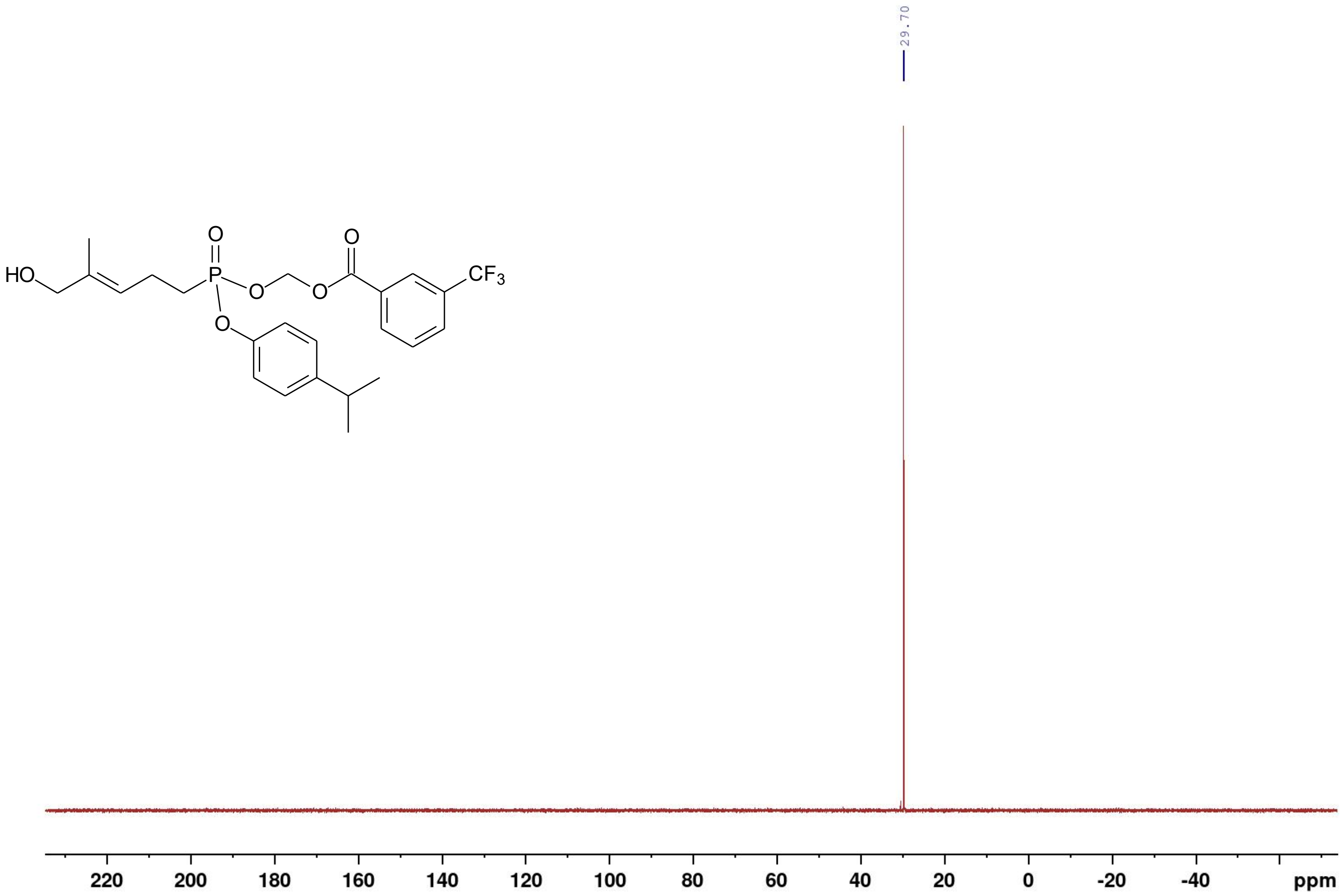

^{31}P NMR Spectrum of Compound **8c** ($CDCl_3$, 203 MHz)

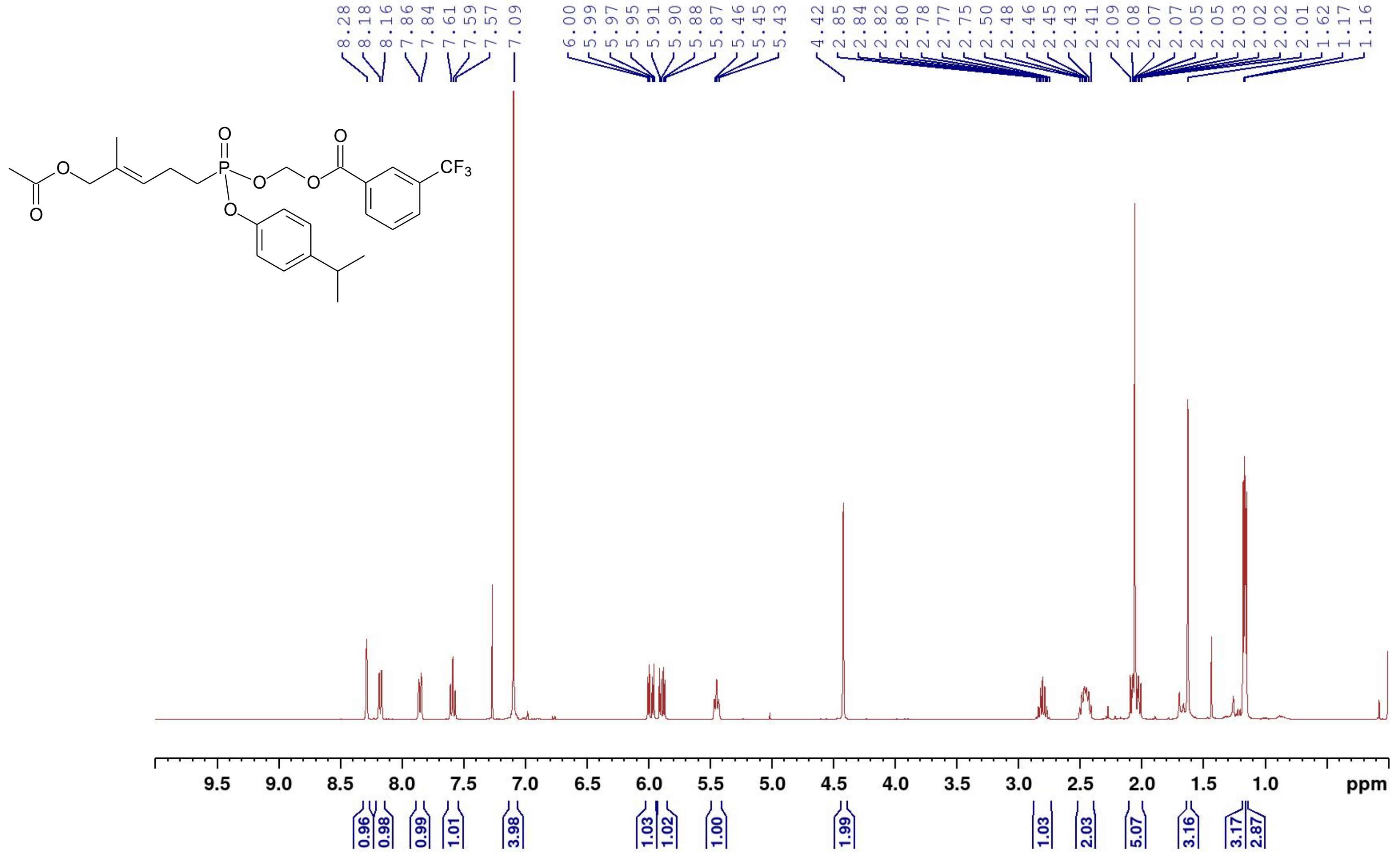

^{1}H NMR Spectrum of Compound **9c** ($CDCl_3$, 400 MHz)

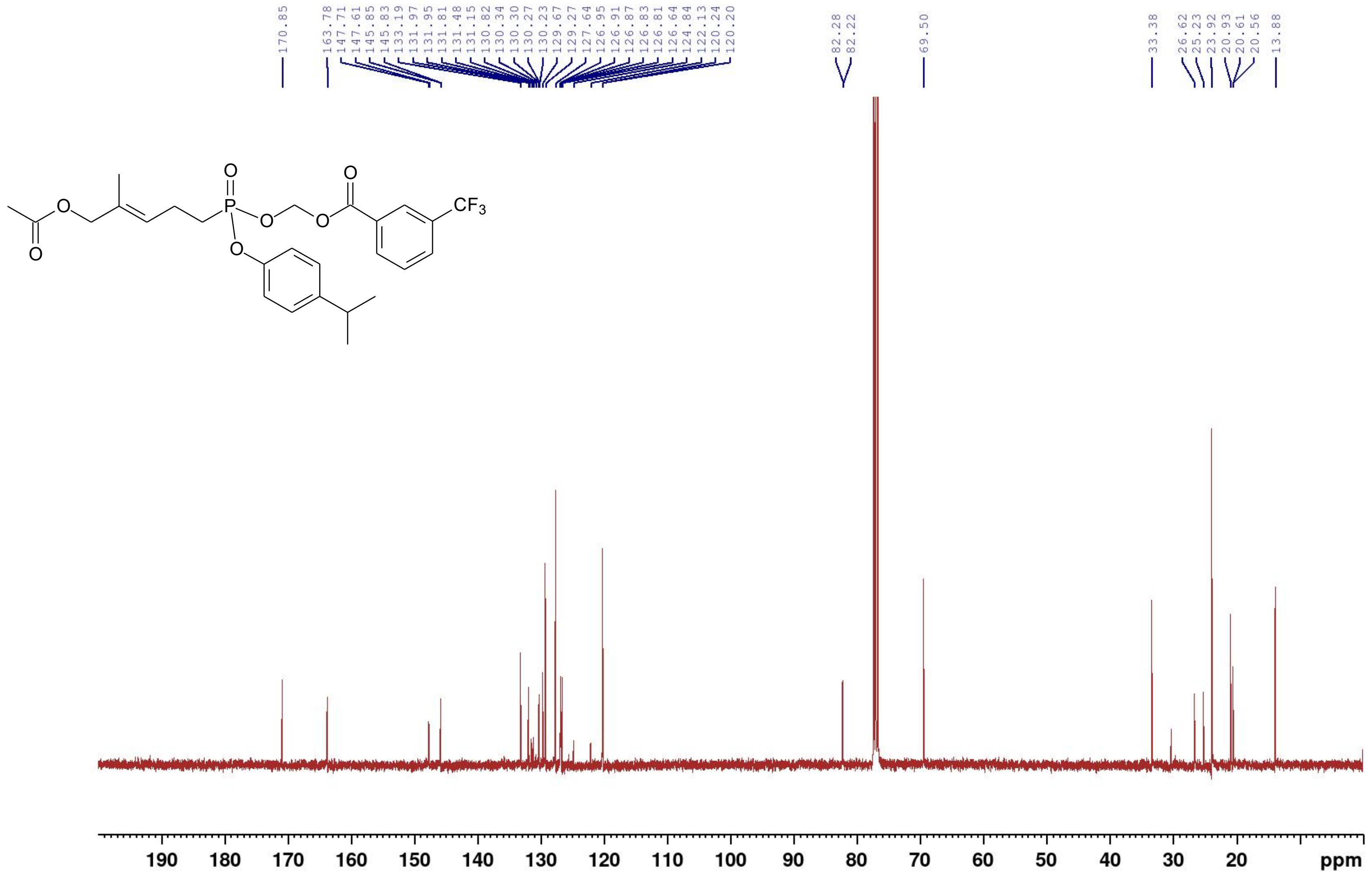

^{13}C NMR Spectrum of Compound **9c** ($CDCl_3$, 101 MHz)

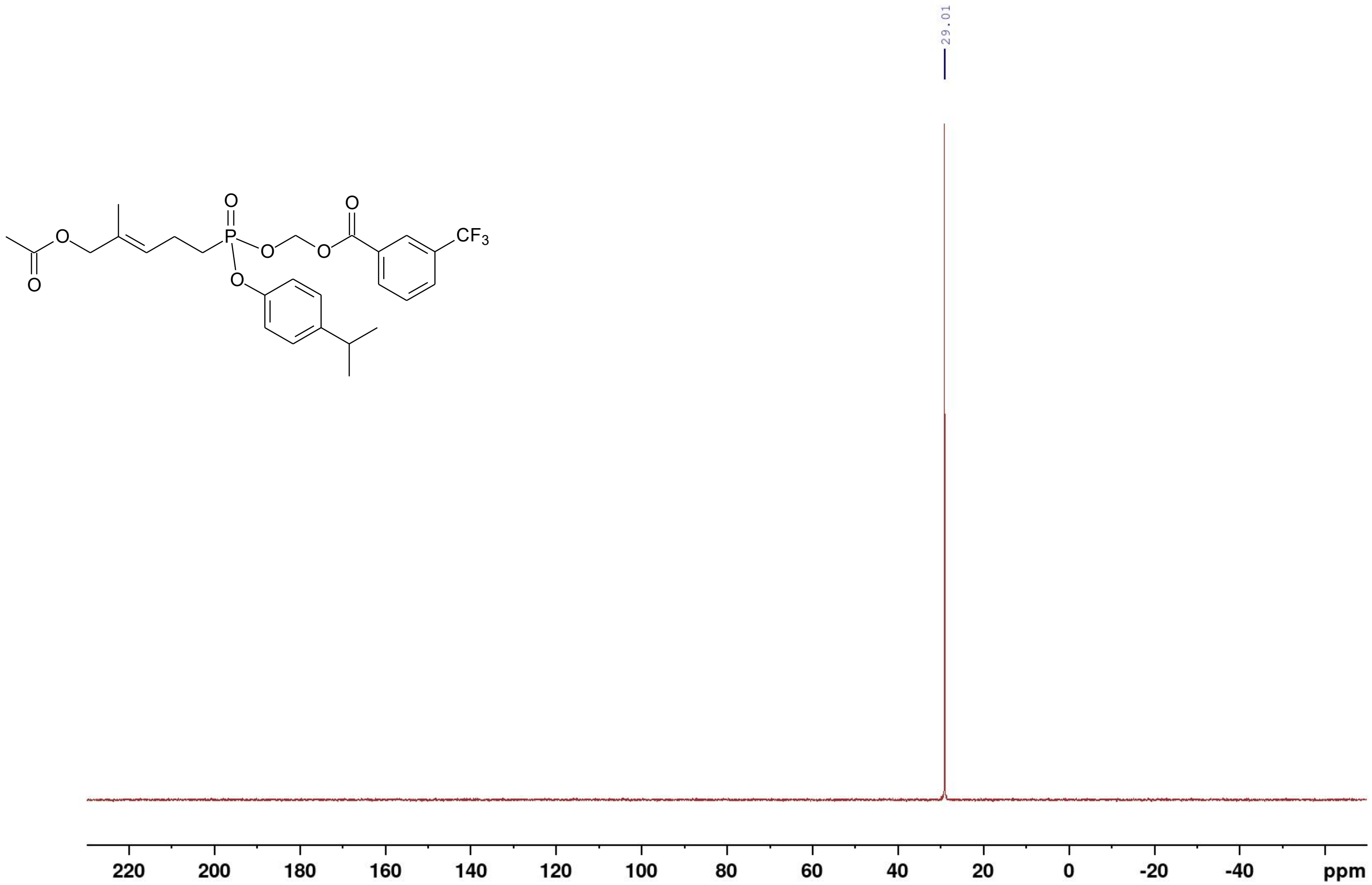

^{31}P NMR Spectrum of Compound **9c** ($CDCl_3$, 162 MHz)

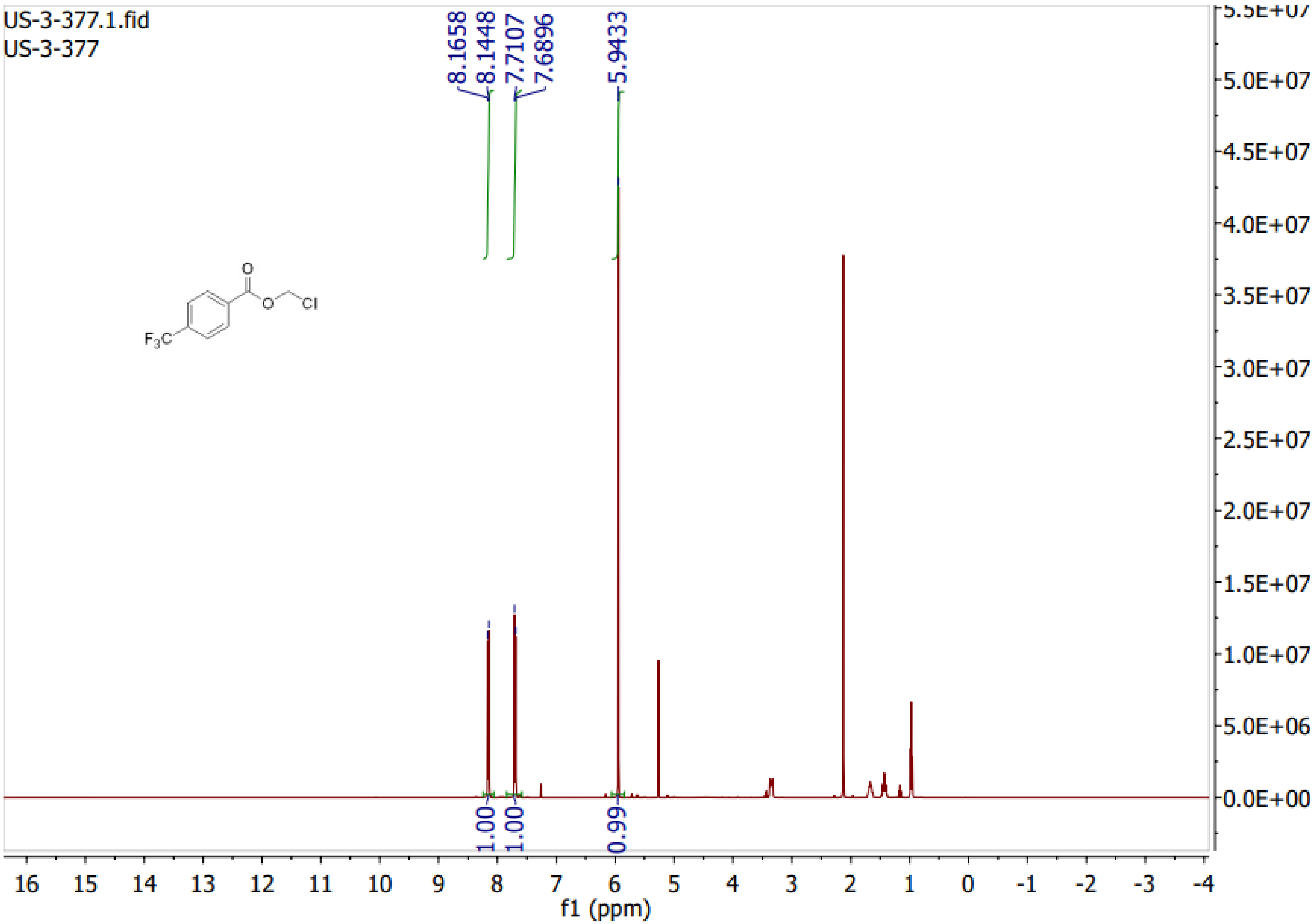

^{1}H NMR Spectrum of Compound **5d** ($CDCl_3$, 400 MHz)

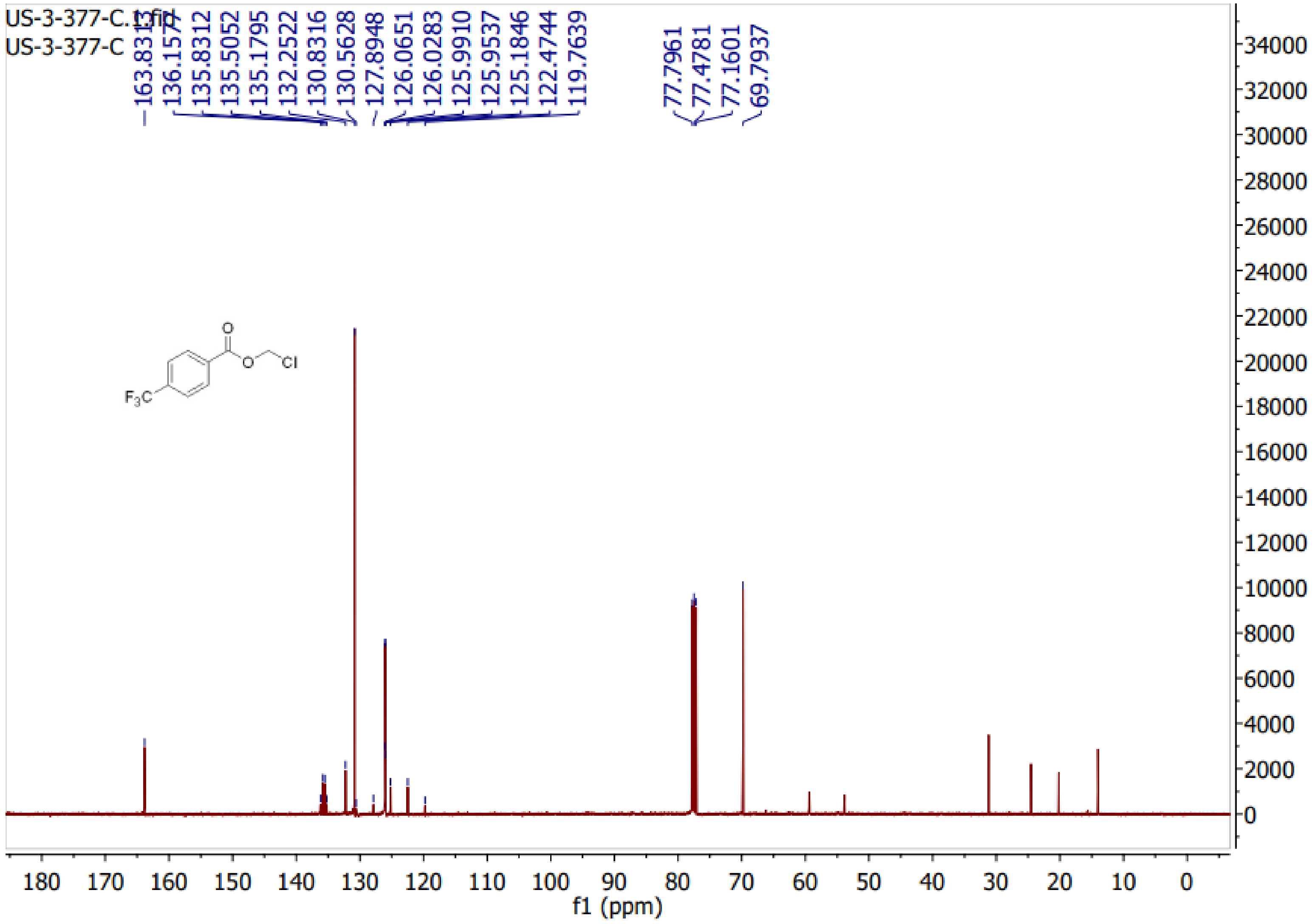

^{13}C NMR Spectrum of Compound **5d** ($CDCl_3$, 101 MHz)

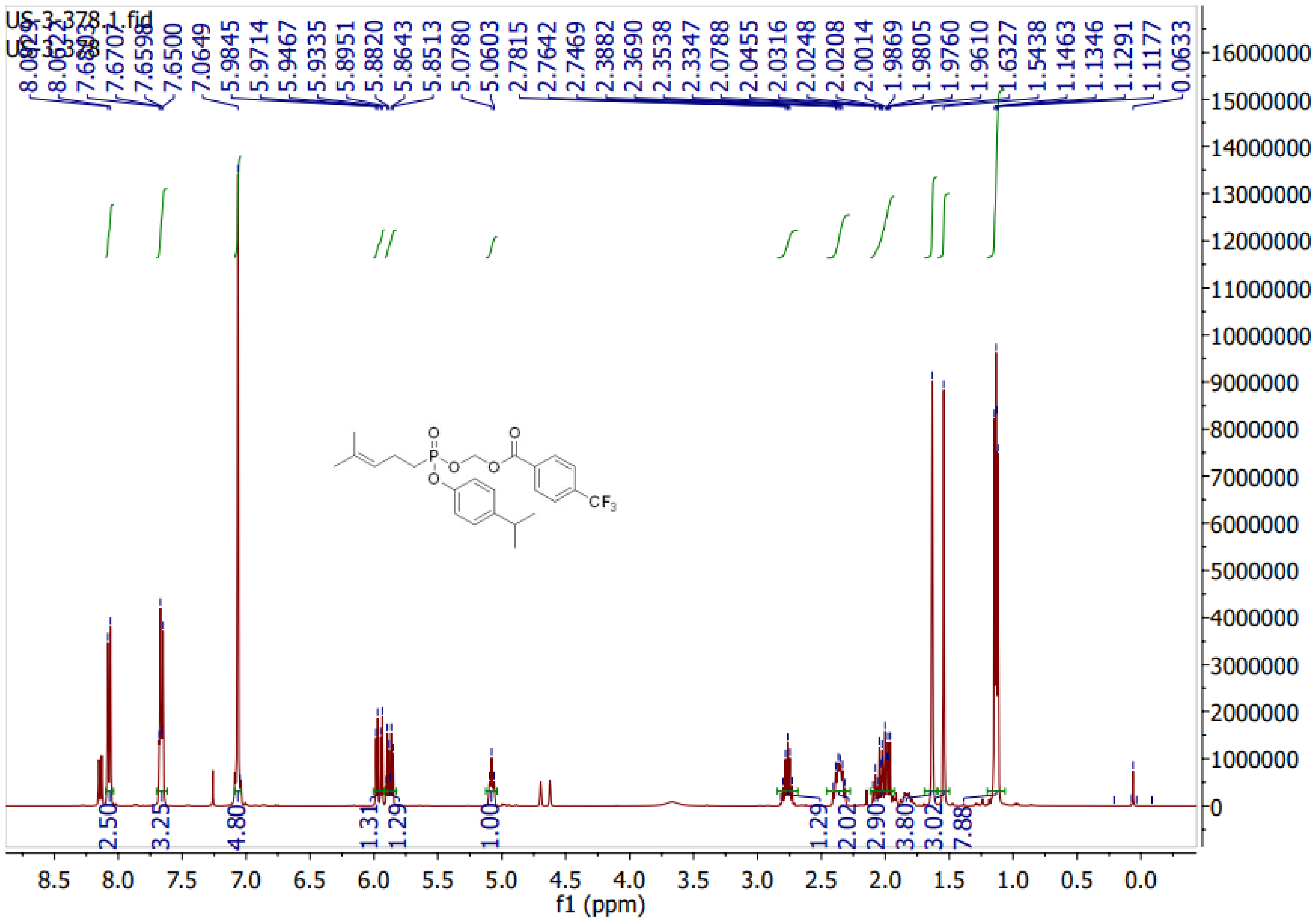

^{1}H NMR Spectrum of Compound **7d** ($CDCl_3$, 400 MHz)

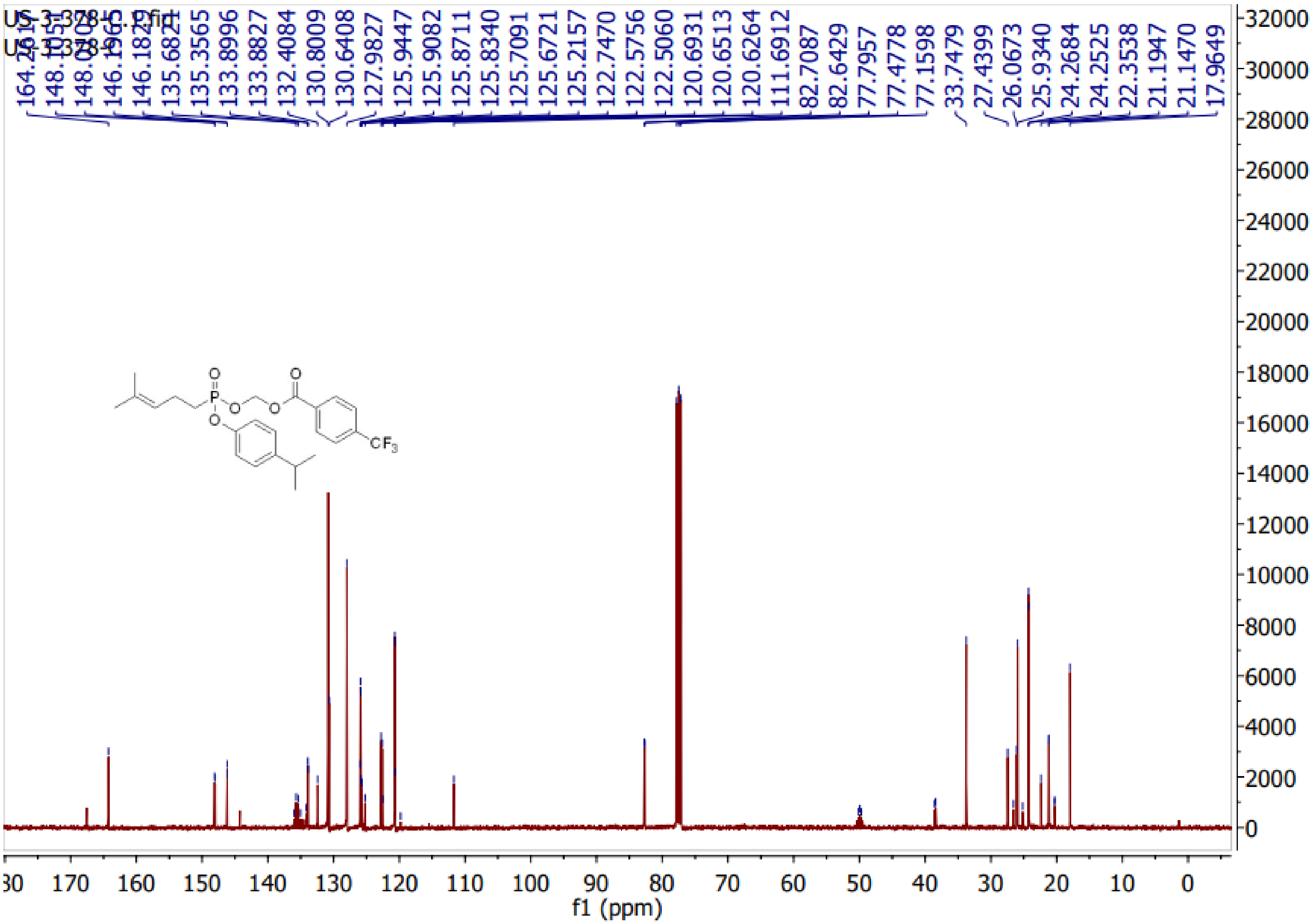

^{13}C NMR Spectrum of Compound **7d** ($CDCl_3$, 101 MHz)

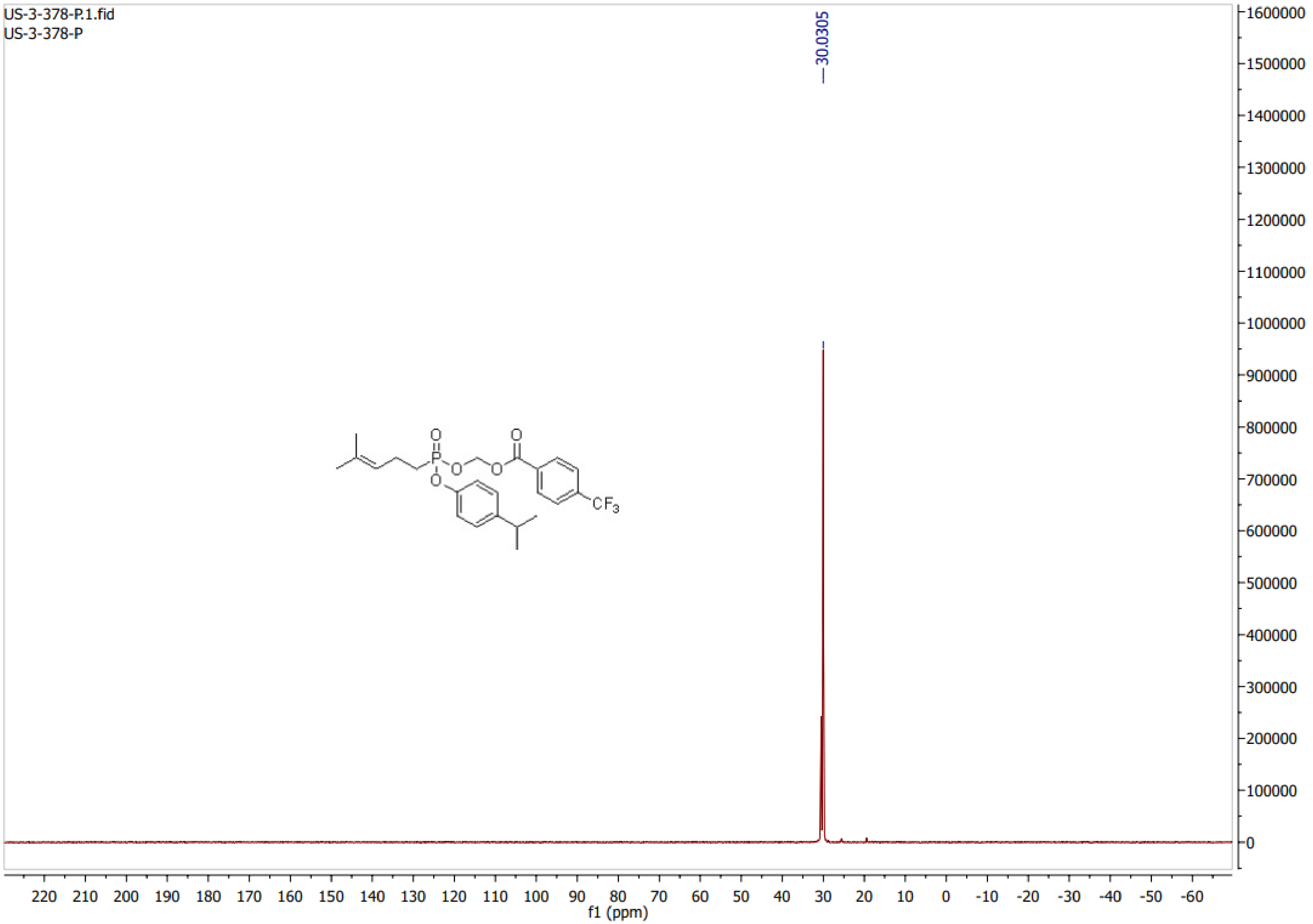

^{31}P NMR Spectrum of Compound **7d** ($CDCl_3$, 162 MHz)

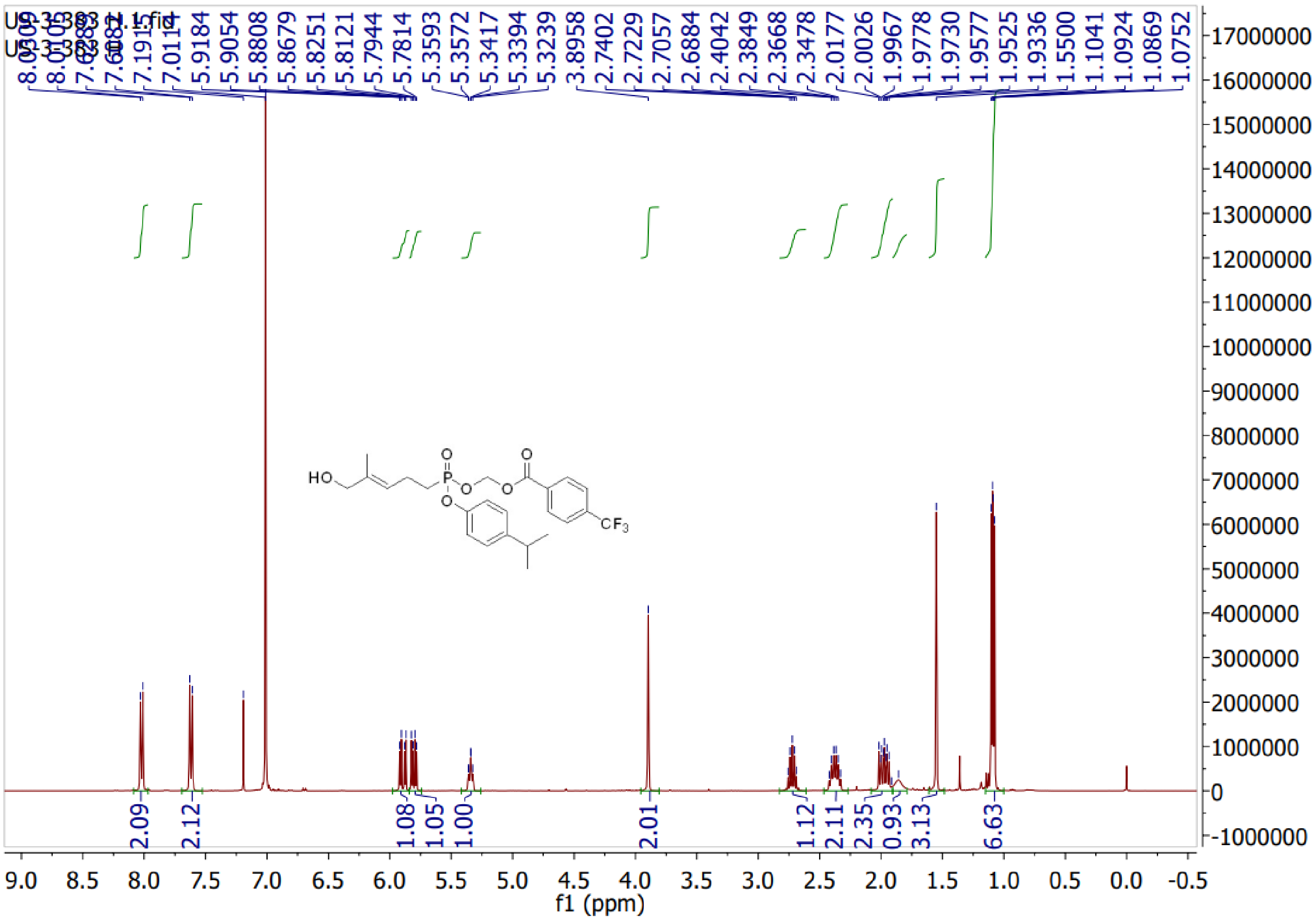

^{1}H NMR Spectrum of Compound **8d** ($CDCl_3$, 400 MHz)

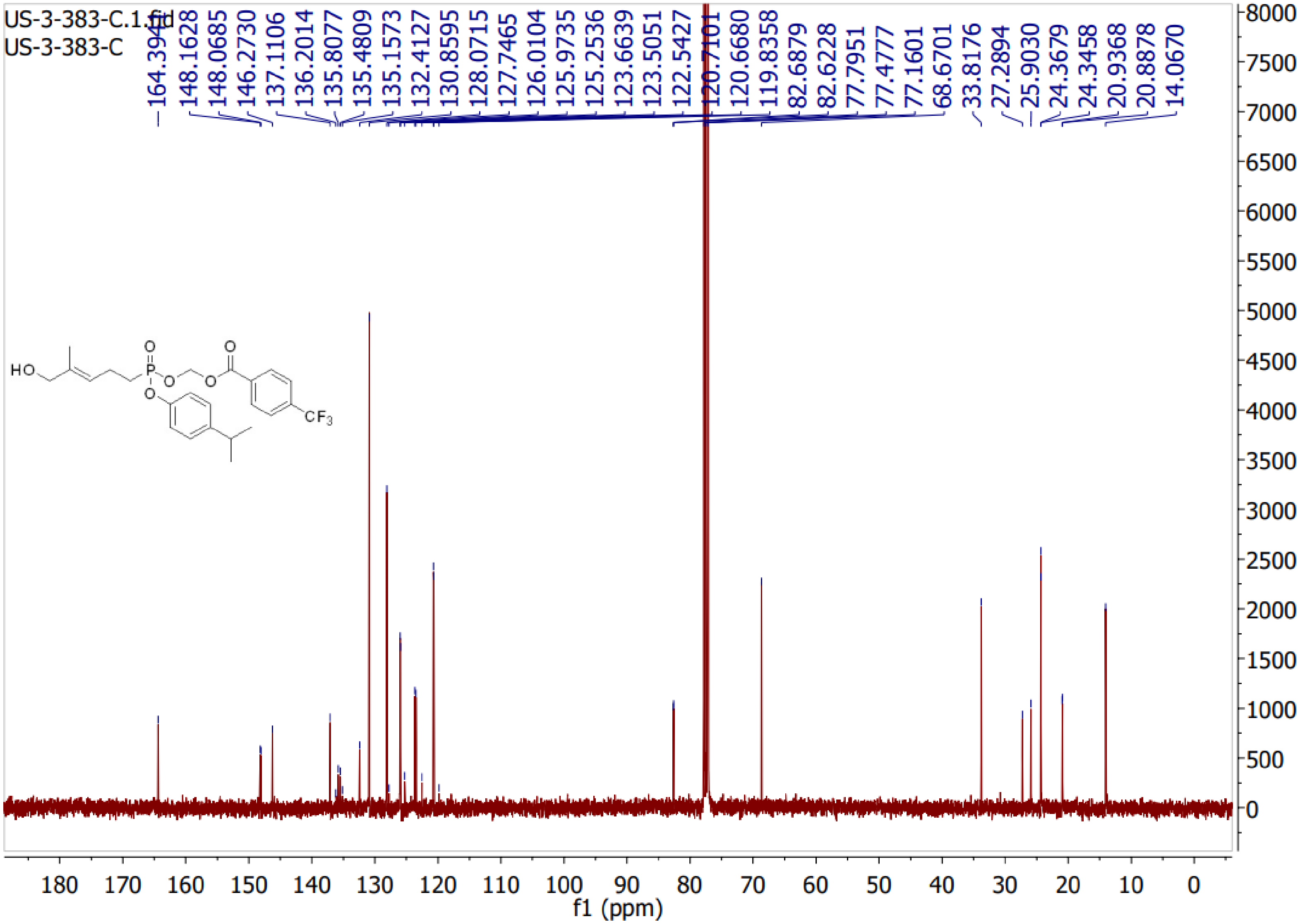

^{13}C NMR Spectrum of Compound **8d** ($CDCl_3$, 101 MHz)

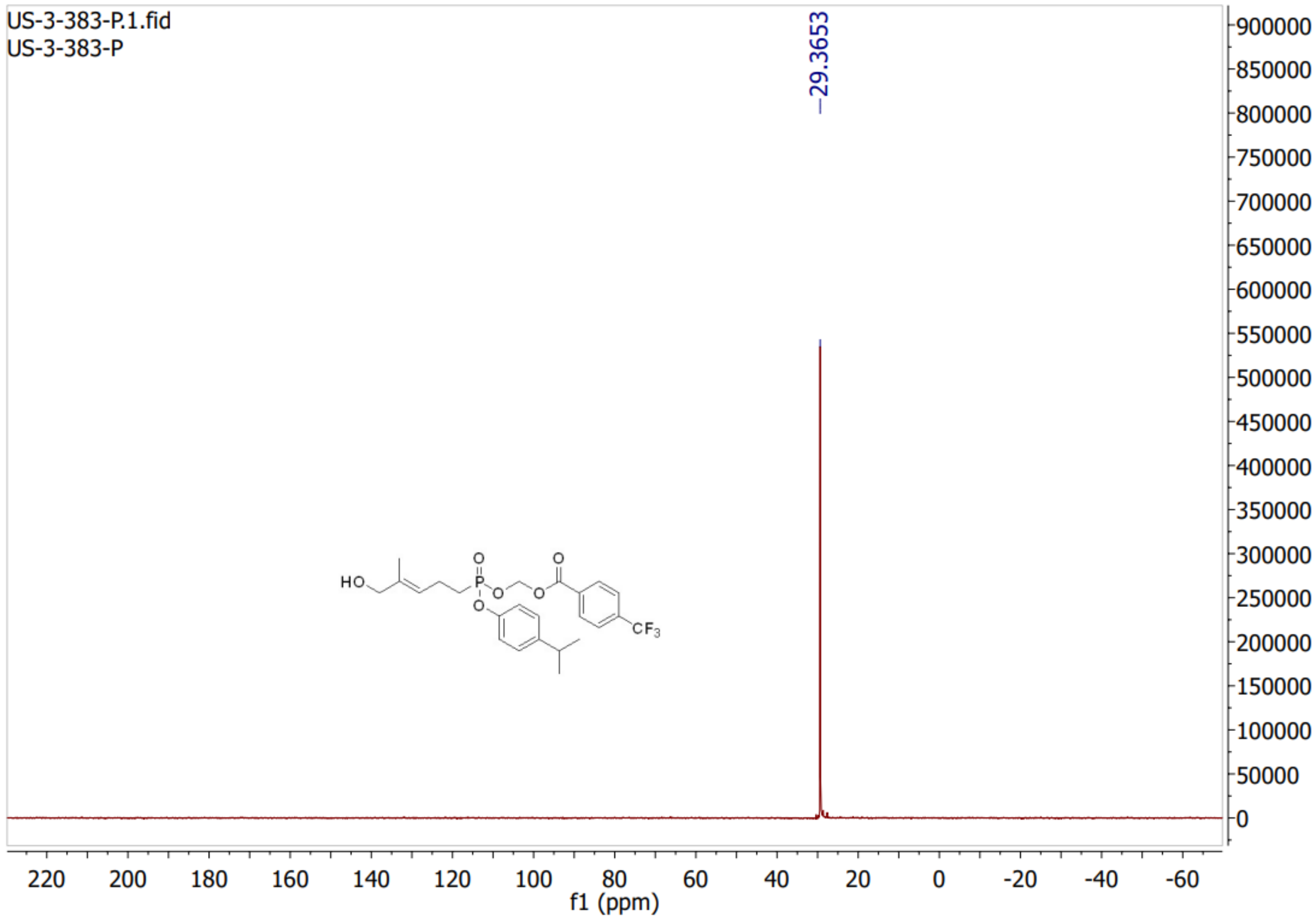

^{31}P NMR Spectrum of Compound **8d** ($CDCl_3$, 162 MHz)

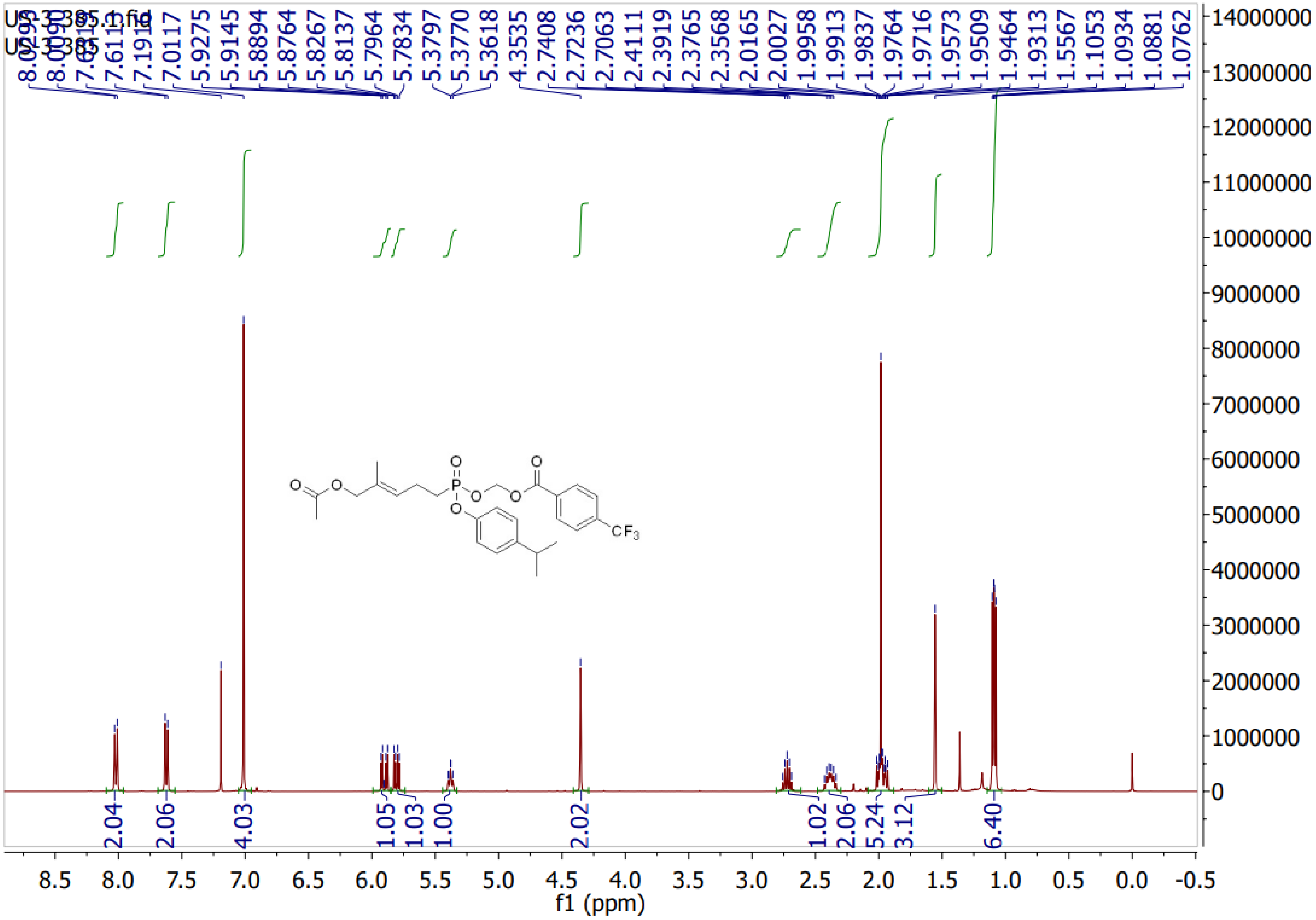

^{1}H NMR Spectrum of Compound **9d** ($CDCl_3$, 400 MHz)

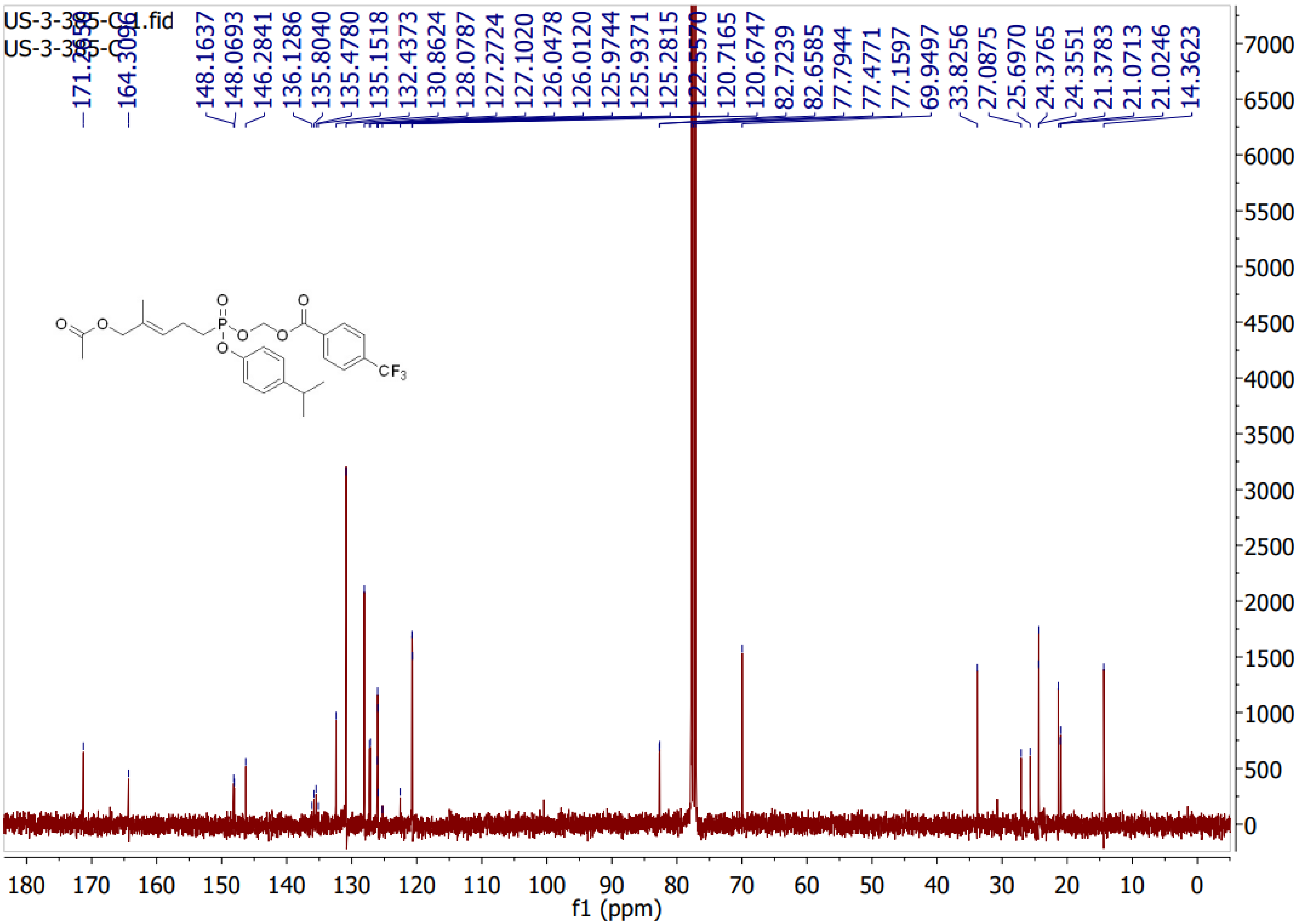

^{13}C NMR Spectrum of Compound **9d** ($CDCl_3$, 101 MHz)

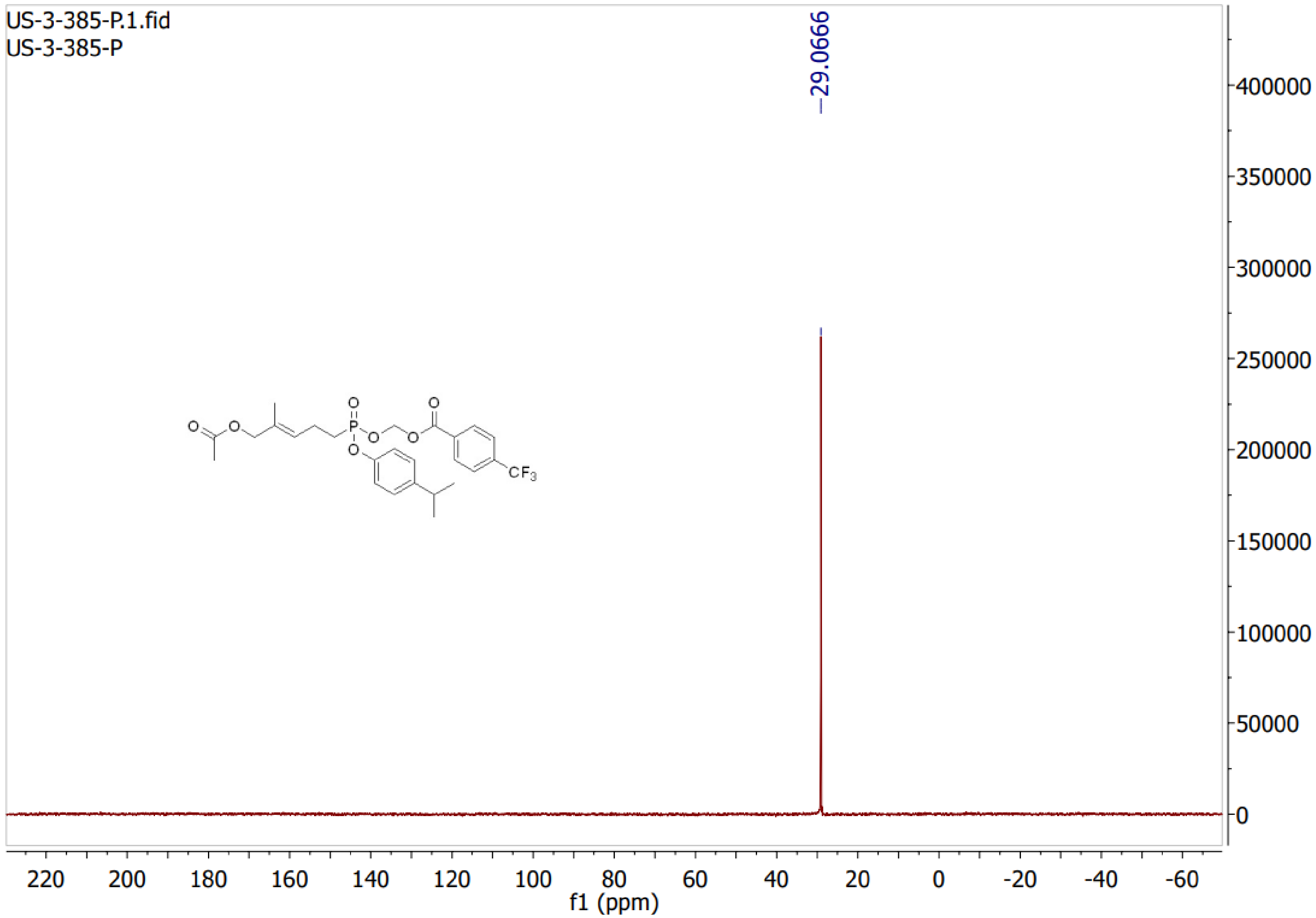

^{31}P NMR Spectrum of Compound **9d** ($CDCl_3$, 162 MHz)

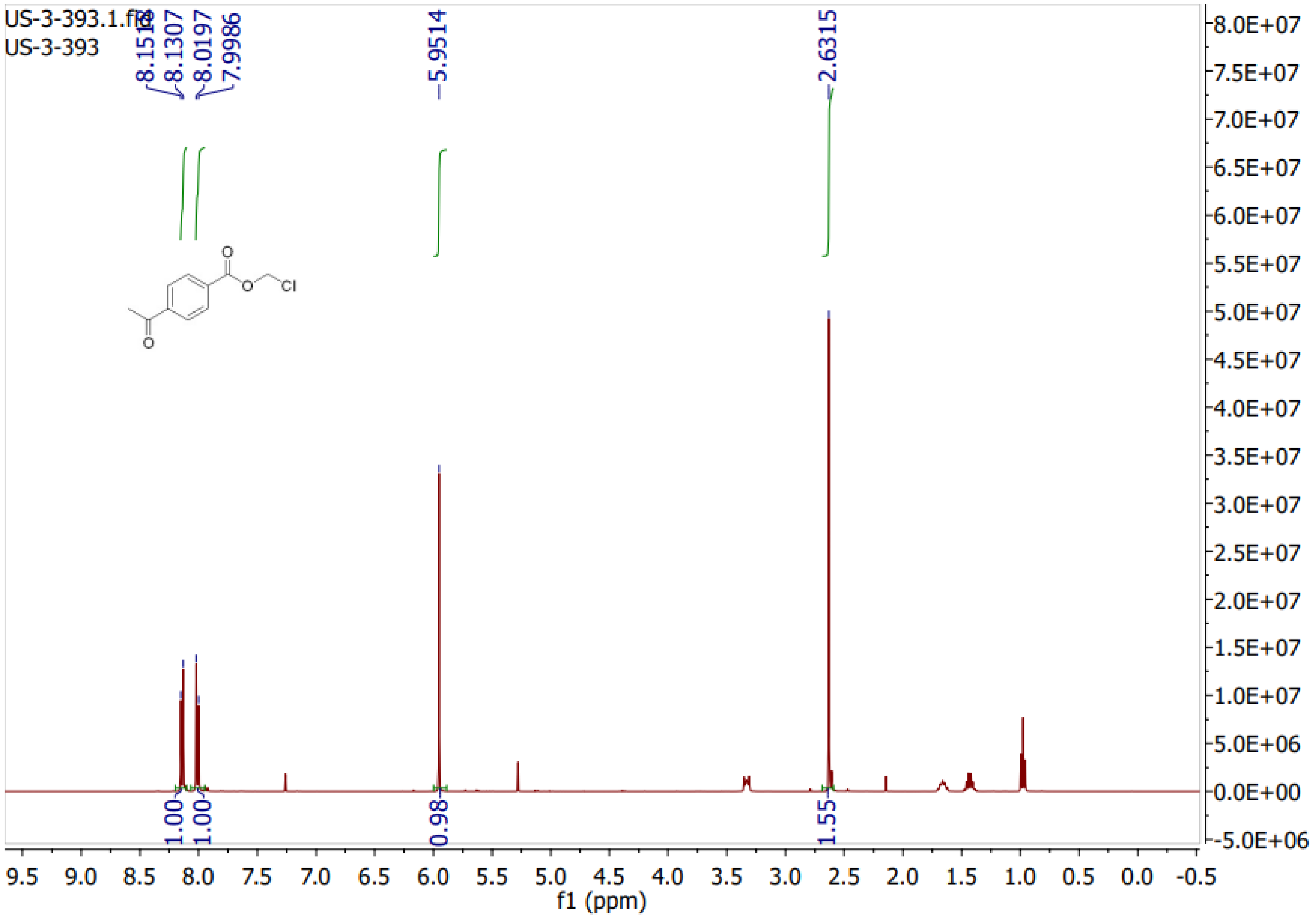

^{1}H NMR Spectrum of Compound **5e** ($CDCl_3$, 400 MHz)

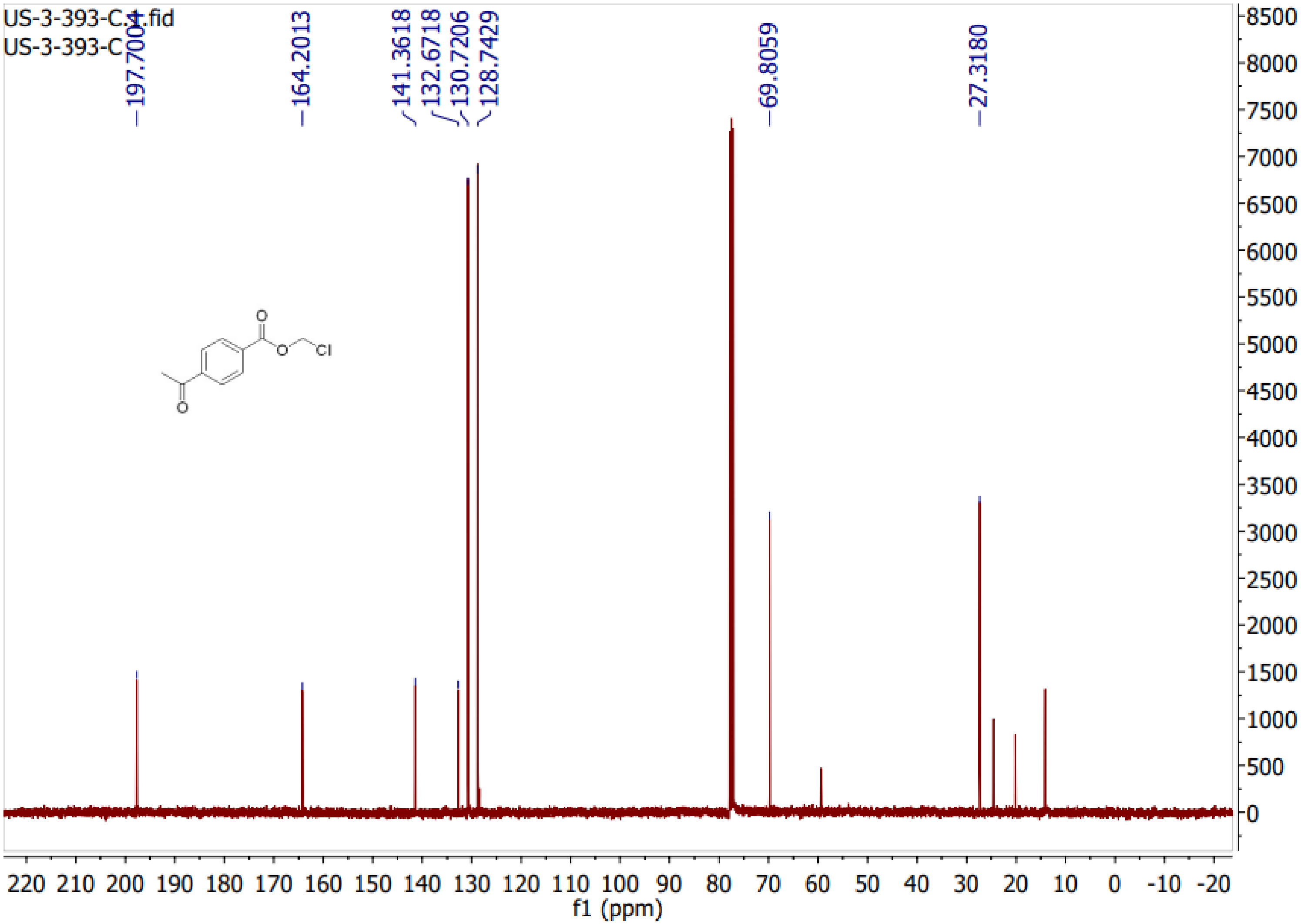

^{13}C NMR Spectrum of Compound **5e** ($CDCl_3$, 101 MHz)

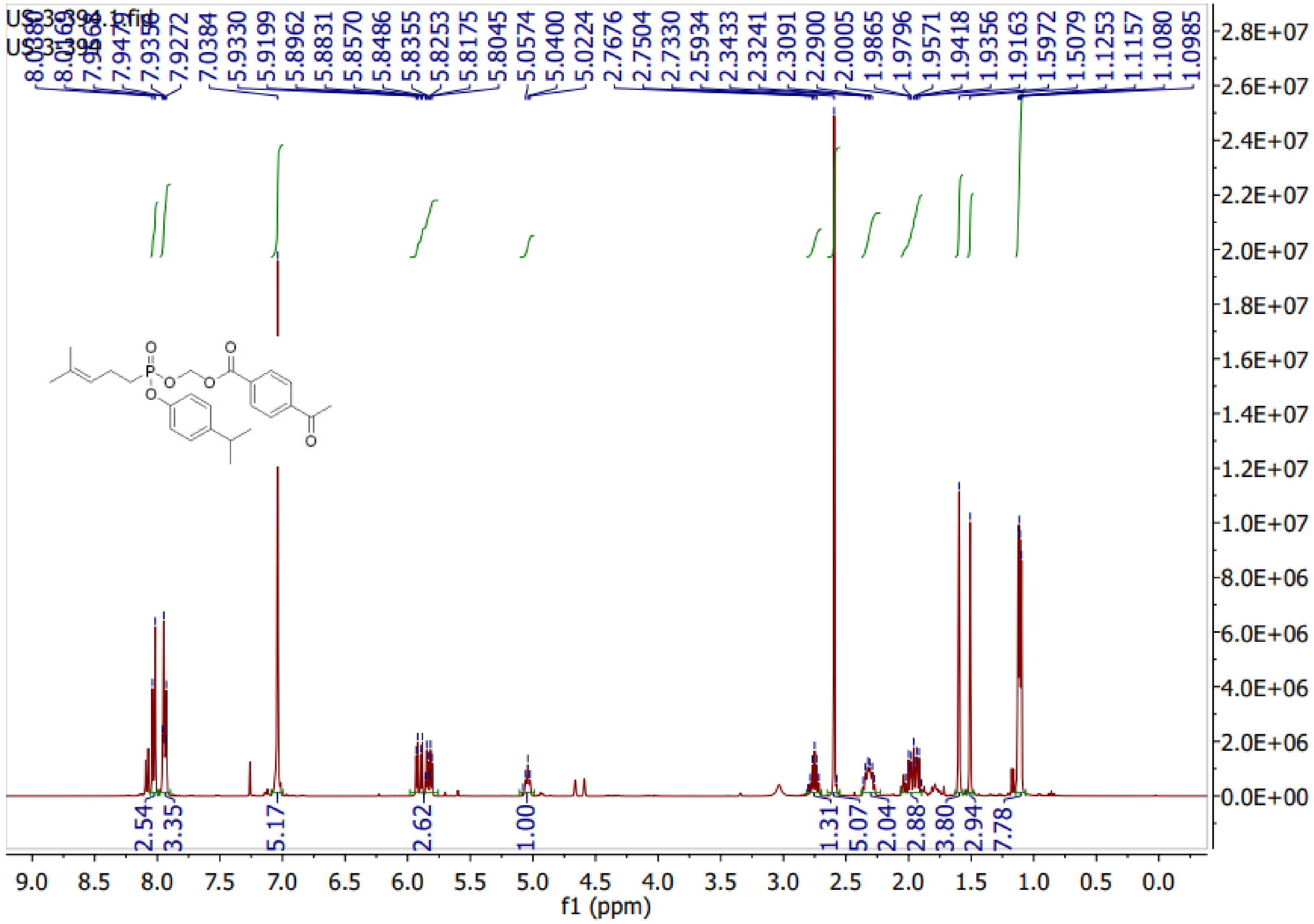

^{1}H NMR Spectrum of Compound **7e** ($CDCl_3$, 400 MHz)

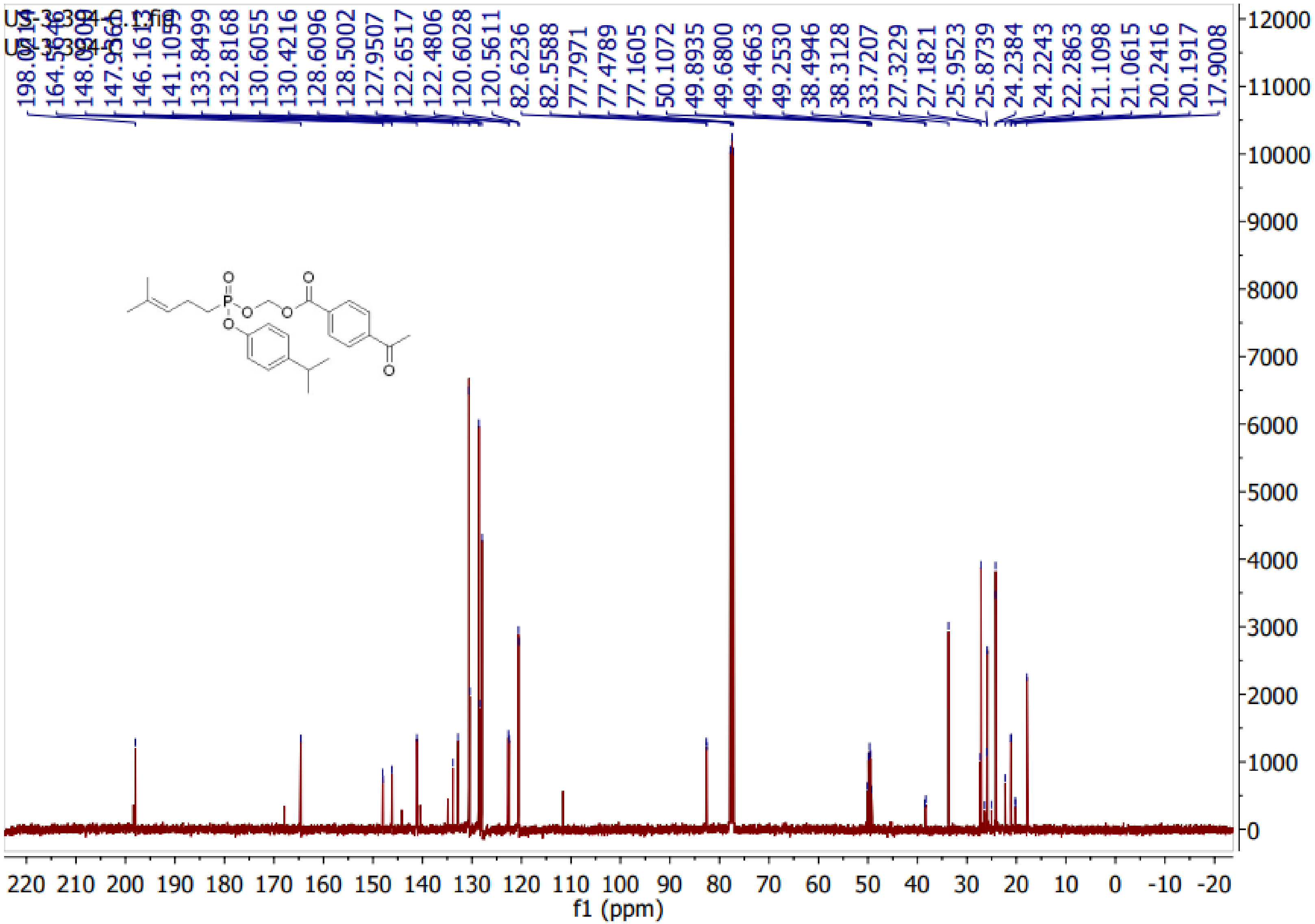

^{13}C NMR Spectrum of Compound **7e** ($CDCl_3$, 101 MHz)

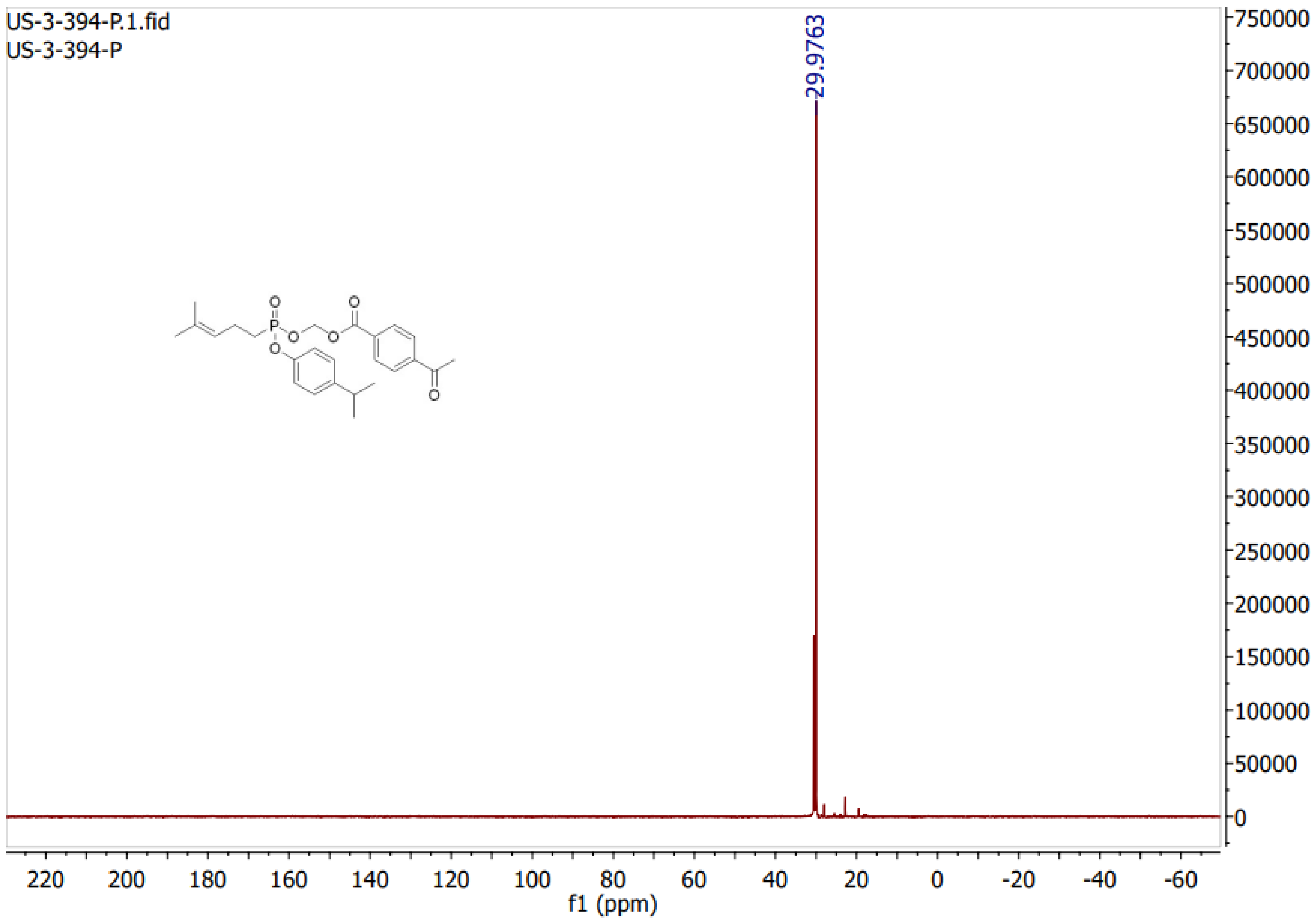

^{31}P NMR Spectrum of Compound **7e** ($CDCl_3$, 162 MHz)

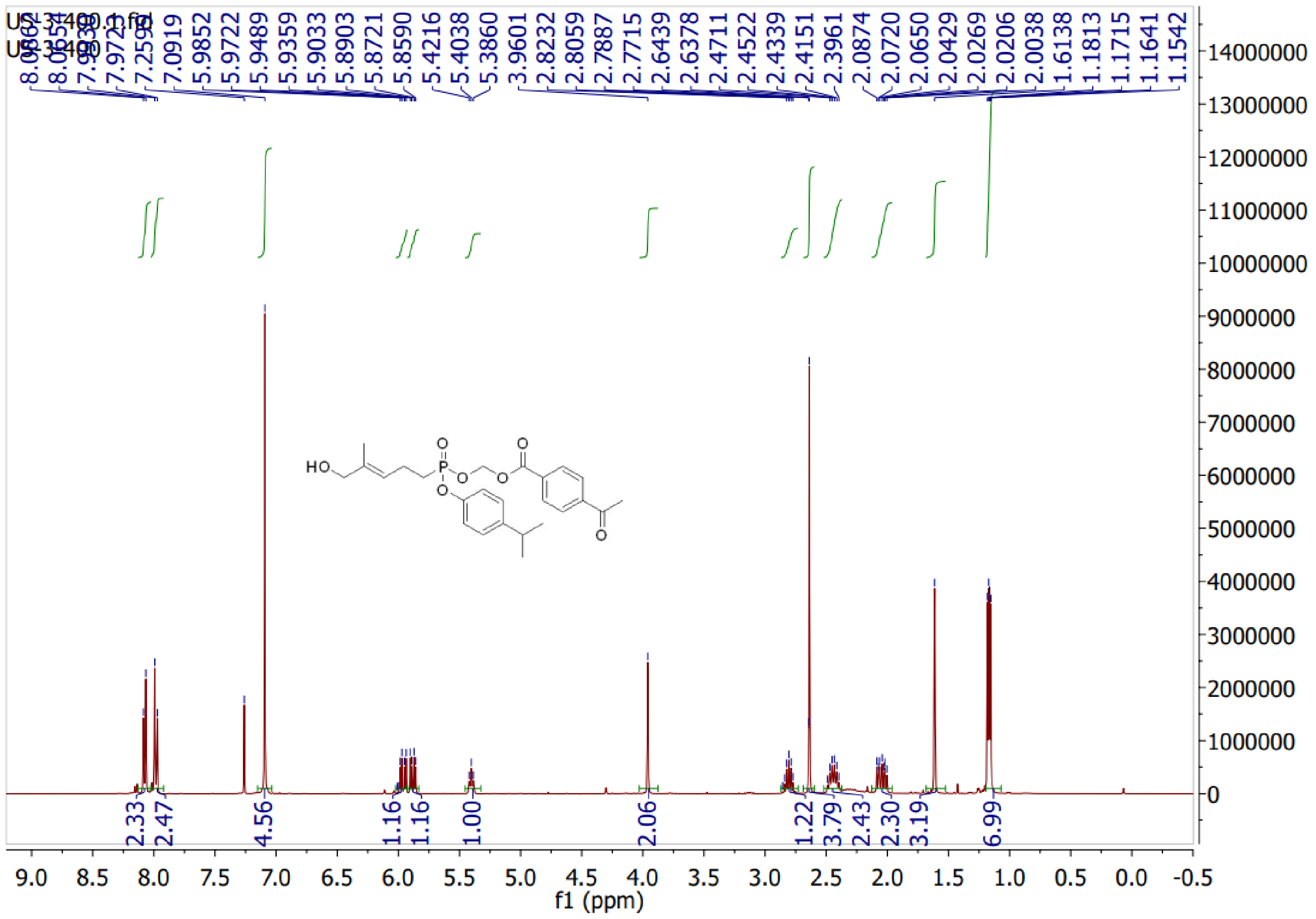

^{1}H NMR Spectrum of Compound **8e** ($CDCl_3$, 400 MHz)

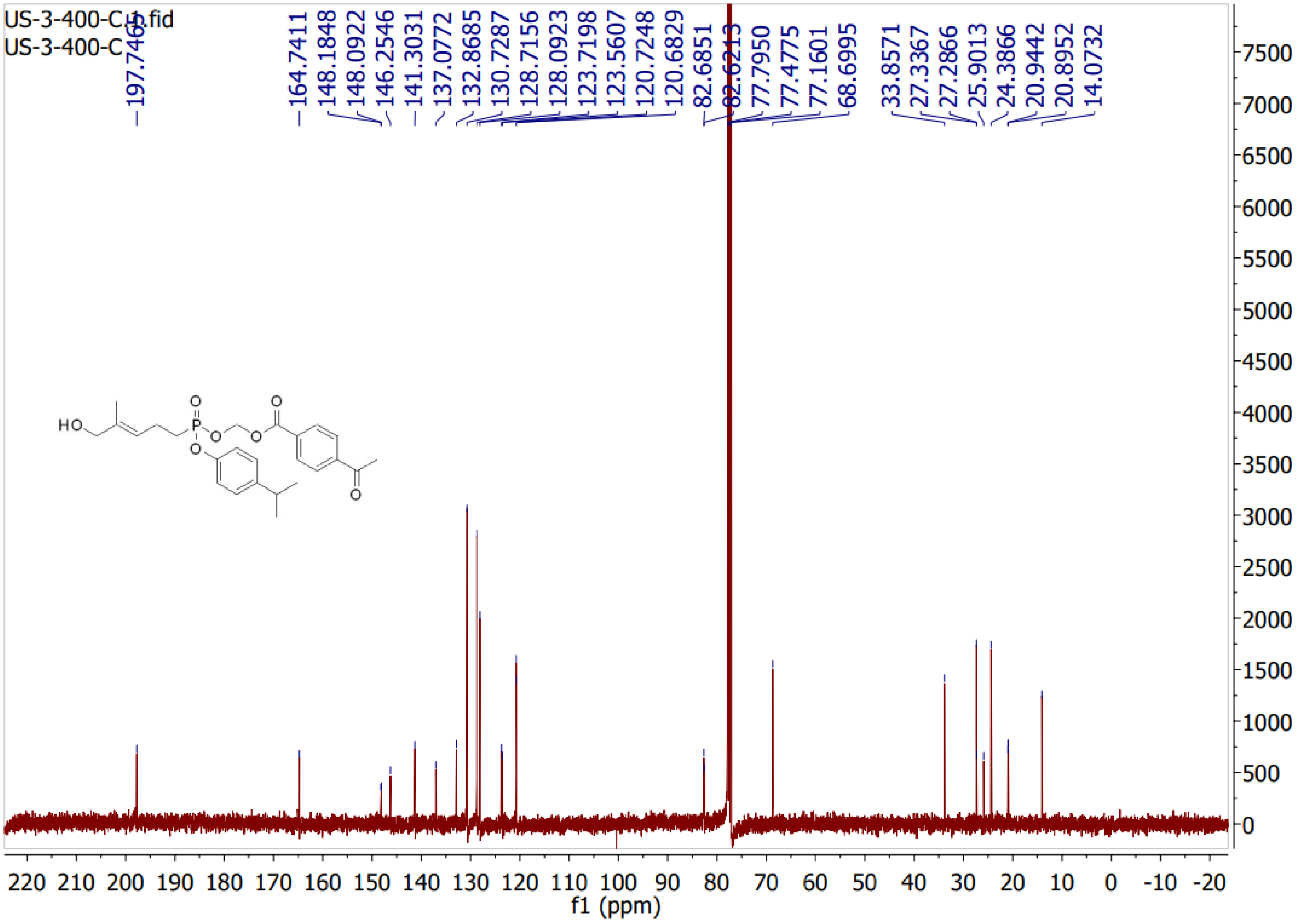

^{13}C NMR Spectrum of Compound **8e** ($CDCl_3$, 101 MHz)

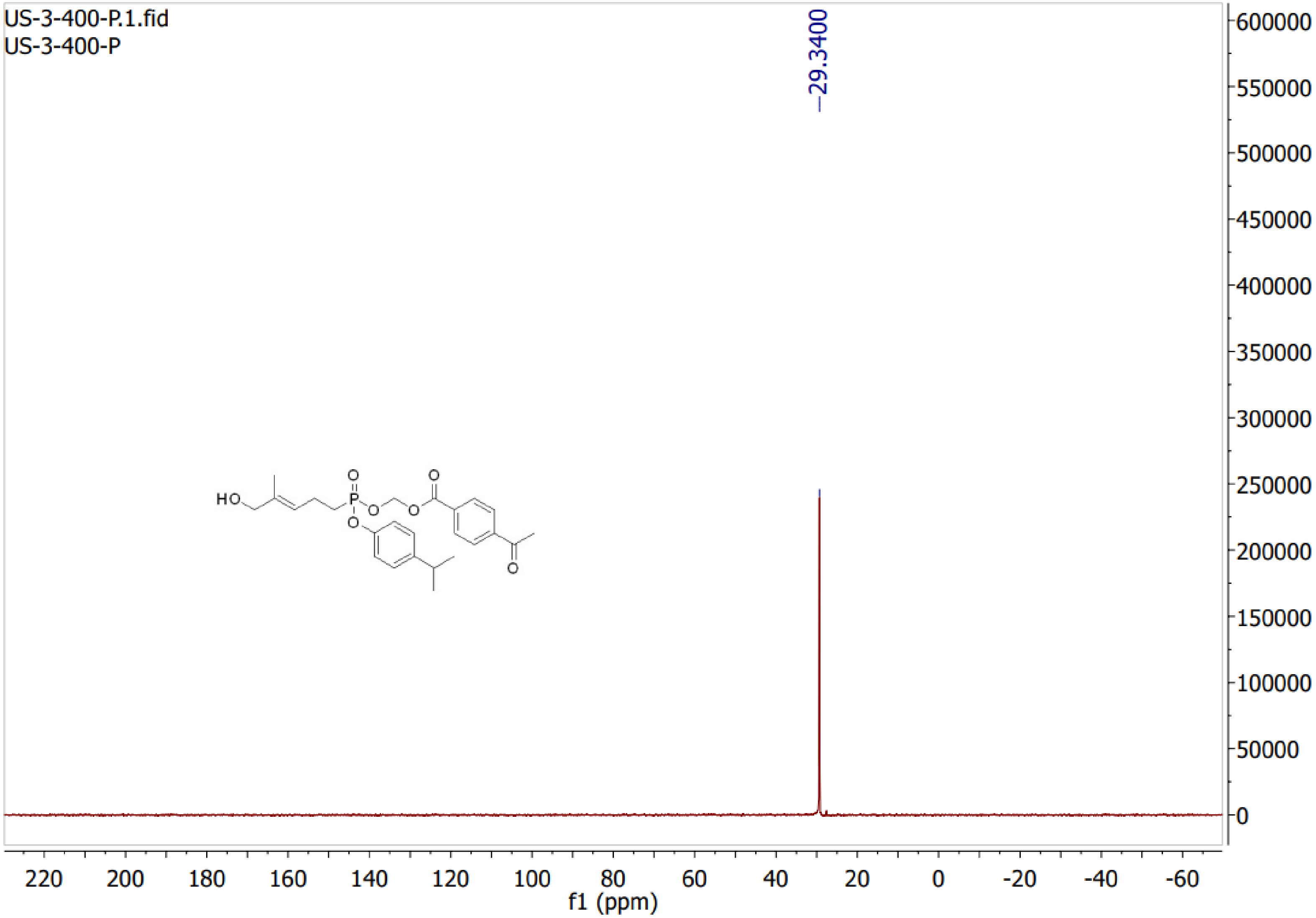

^{31}P NMR Spectrum of Compound **8e** ($CDCl_3$, 162 MHz)

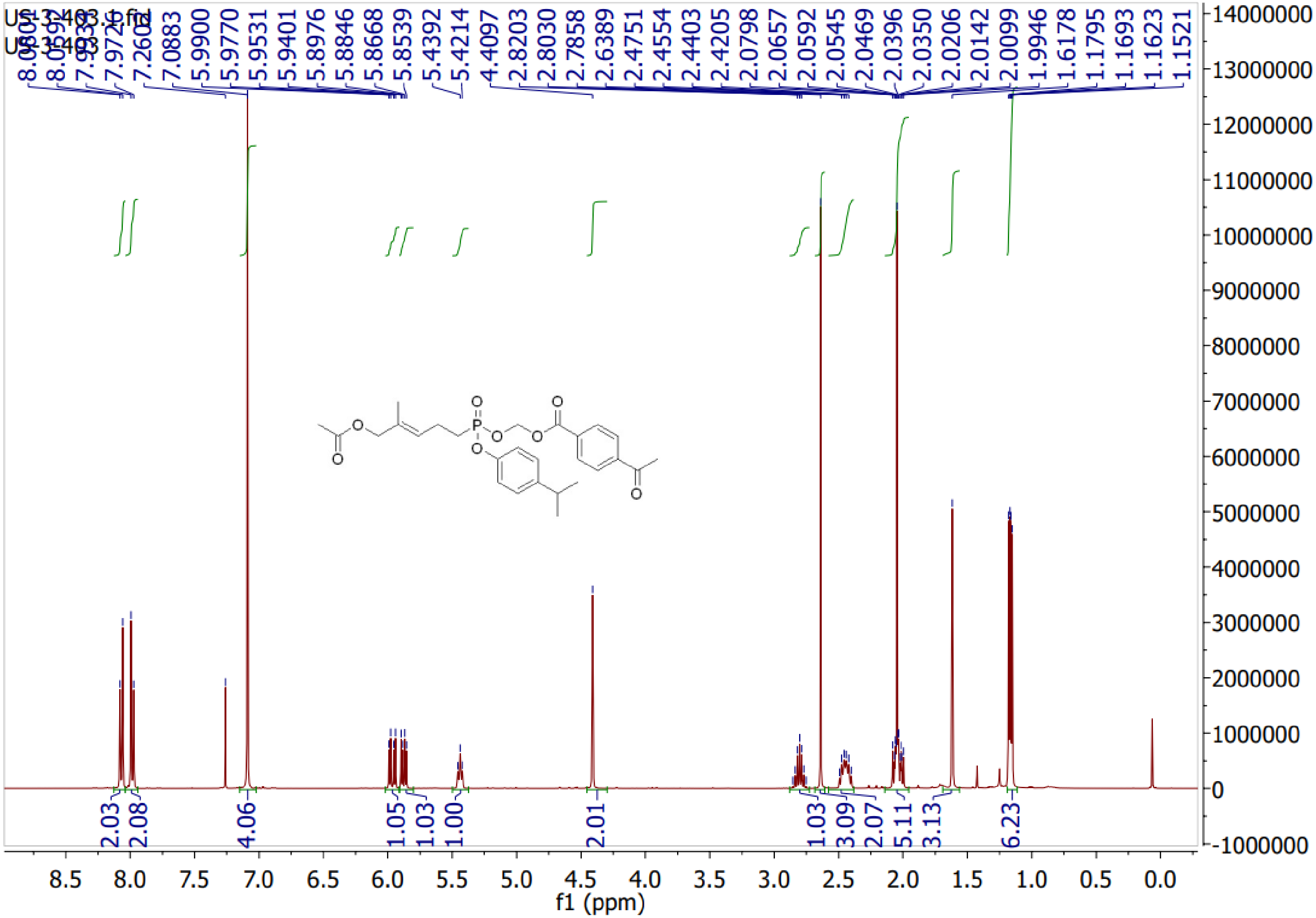

^{1}H NMR Spectrum of Compound **9e** ($CDCl_3$, 400 MHz)

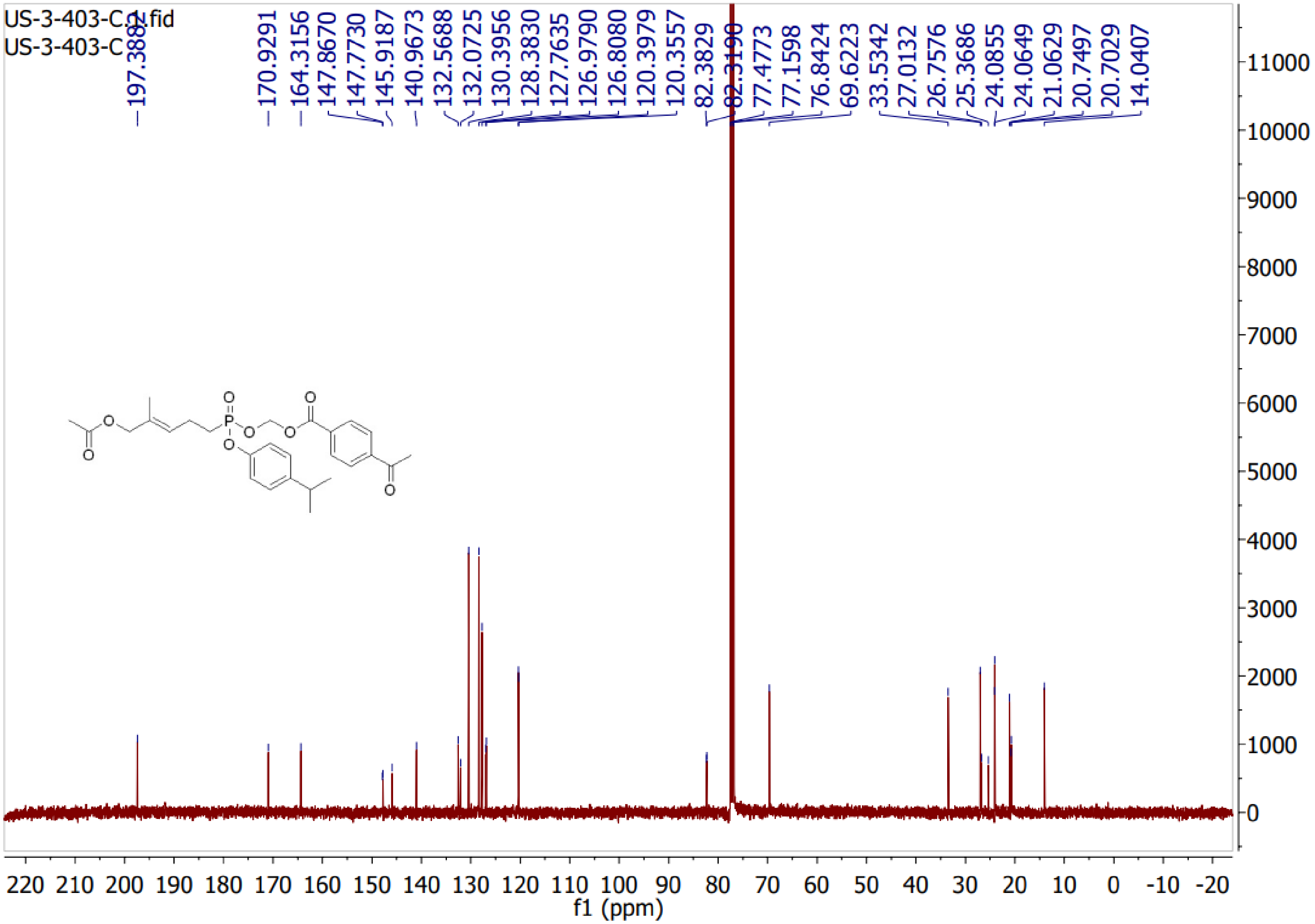

^{13}C NMR Spectrum of Compound **9e** ($CDCl_3$, 101 MHz)

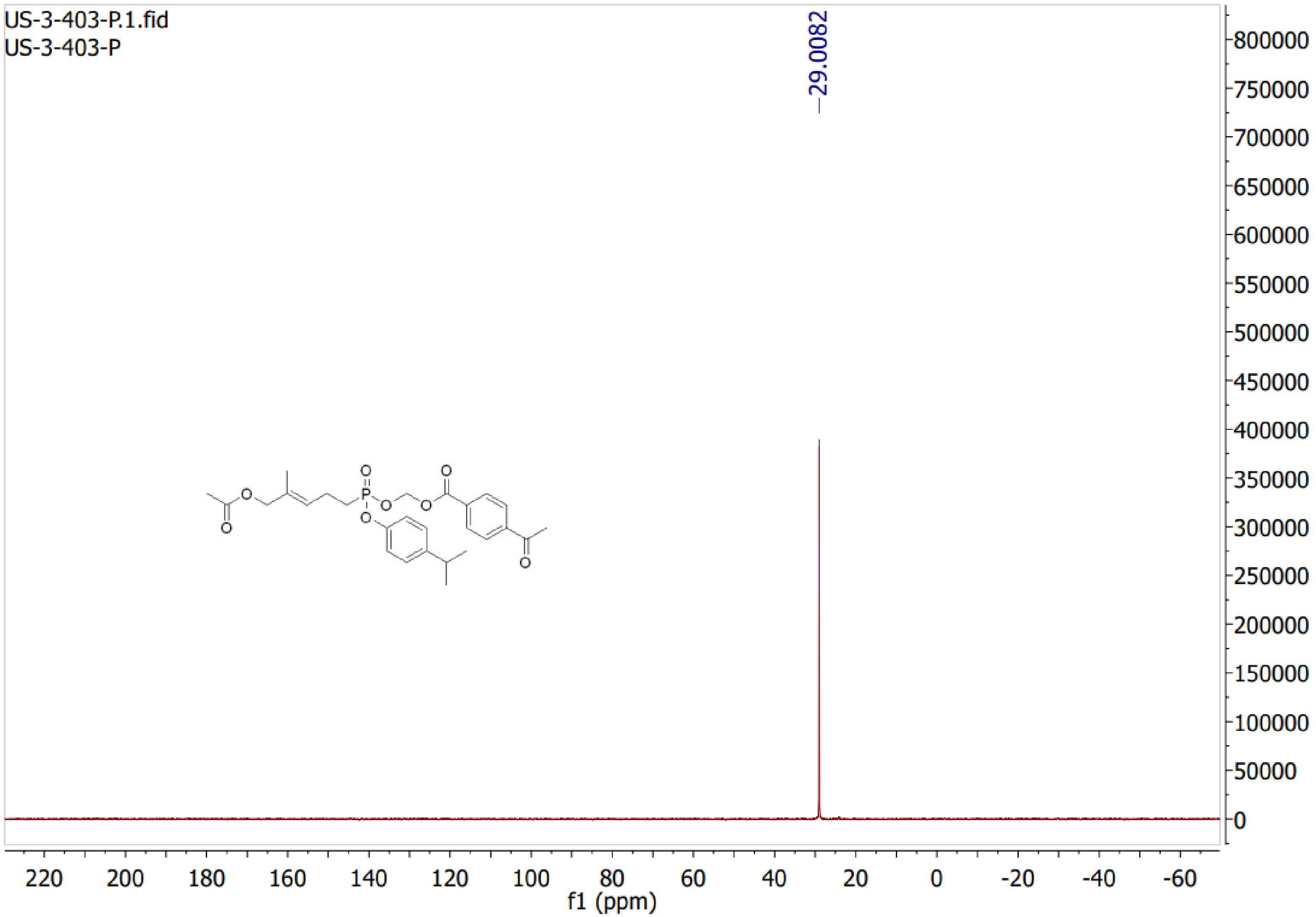

^{31}P NMR Spectrum of Compound **9e** ($CDCl_3$, 162 MHz)

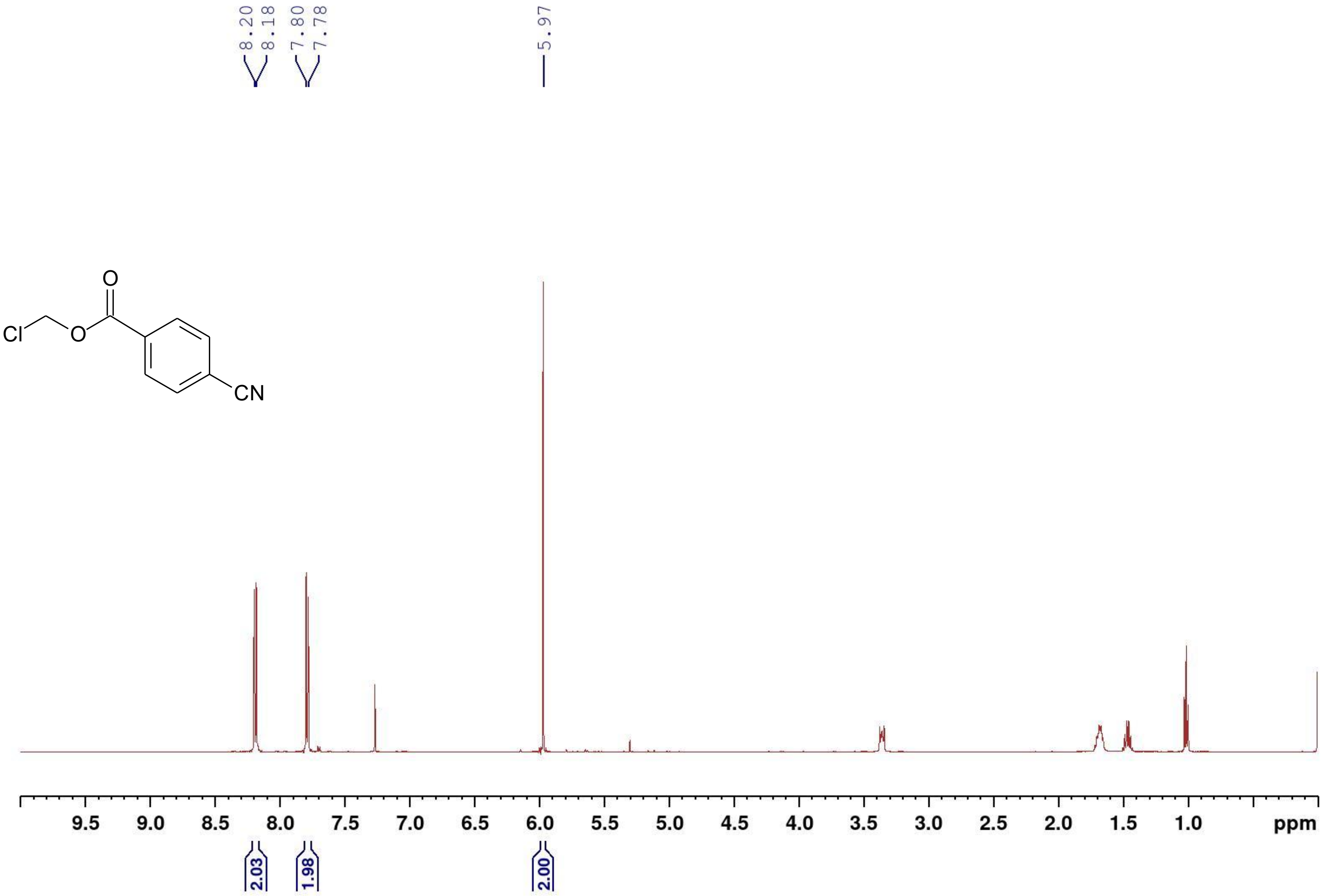

^{1}H NMR Spectrum of Compound **5f** ($CDCl_3$, 500 MHz)

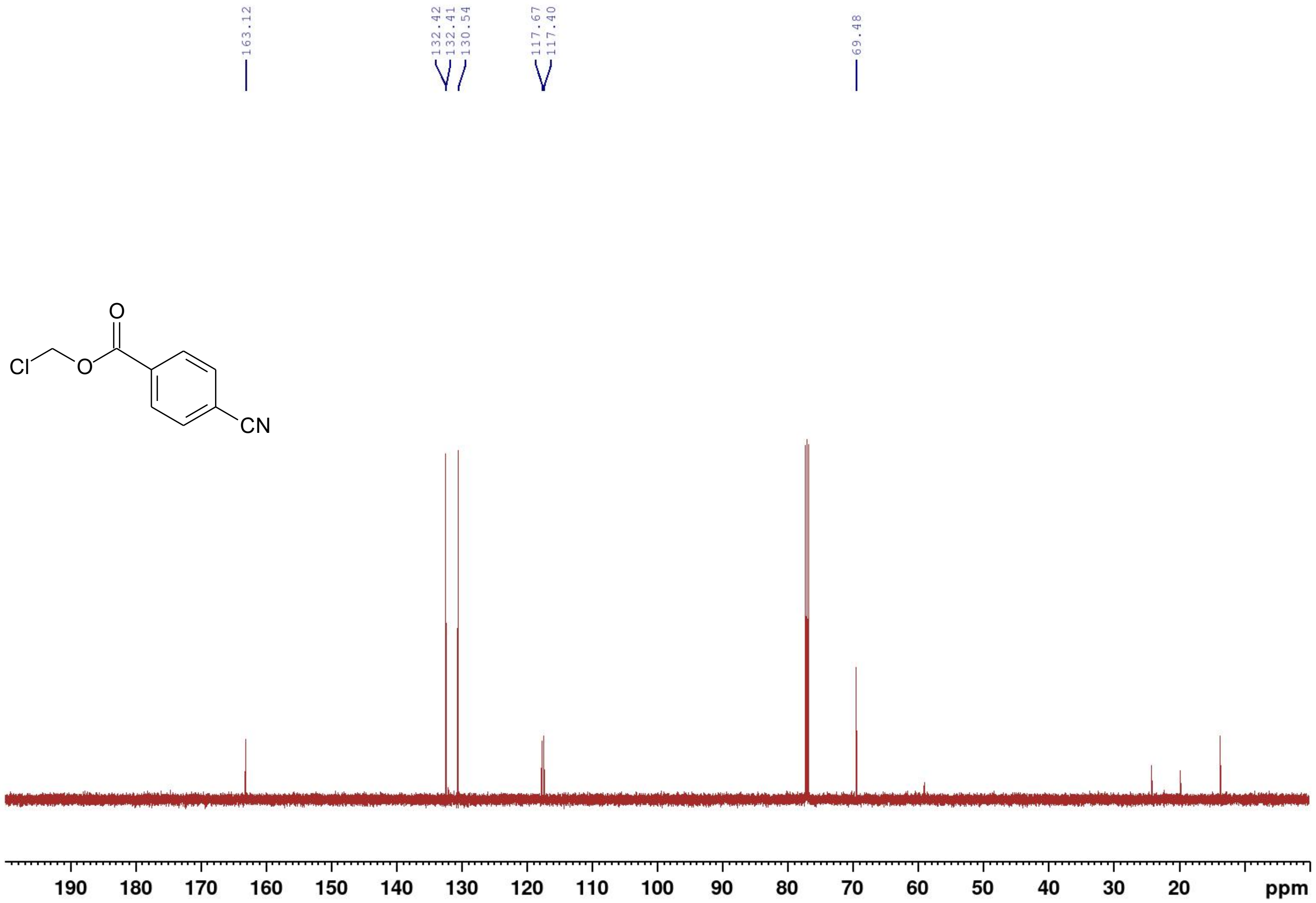

^{13}C NMR Spectrum of Compound **5f** ($CDCl_3$, 126 MHz)

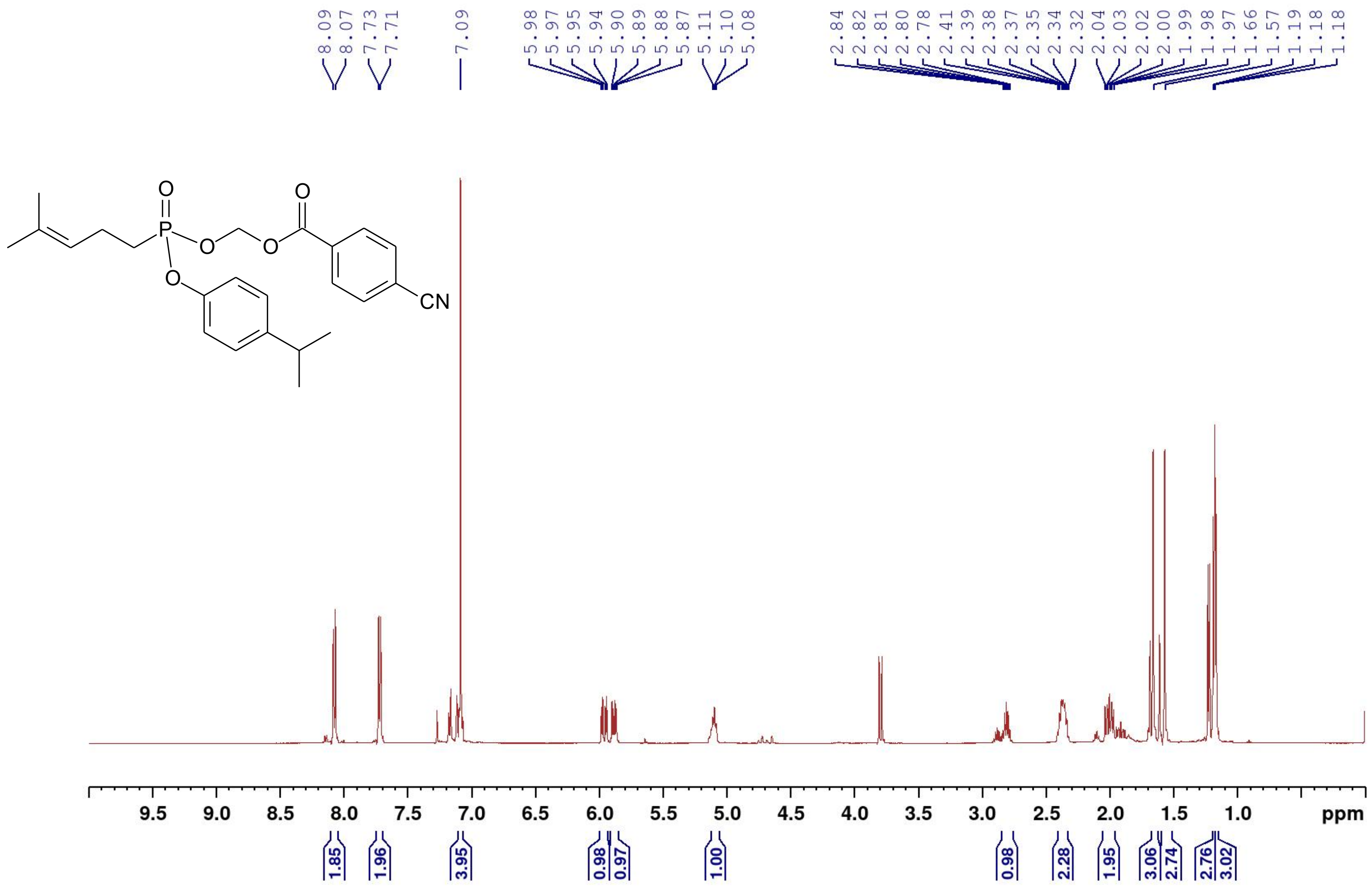

^{1}H NMR Spectrum of Compound **7f** ($CDCl_3$, 500 MHz)

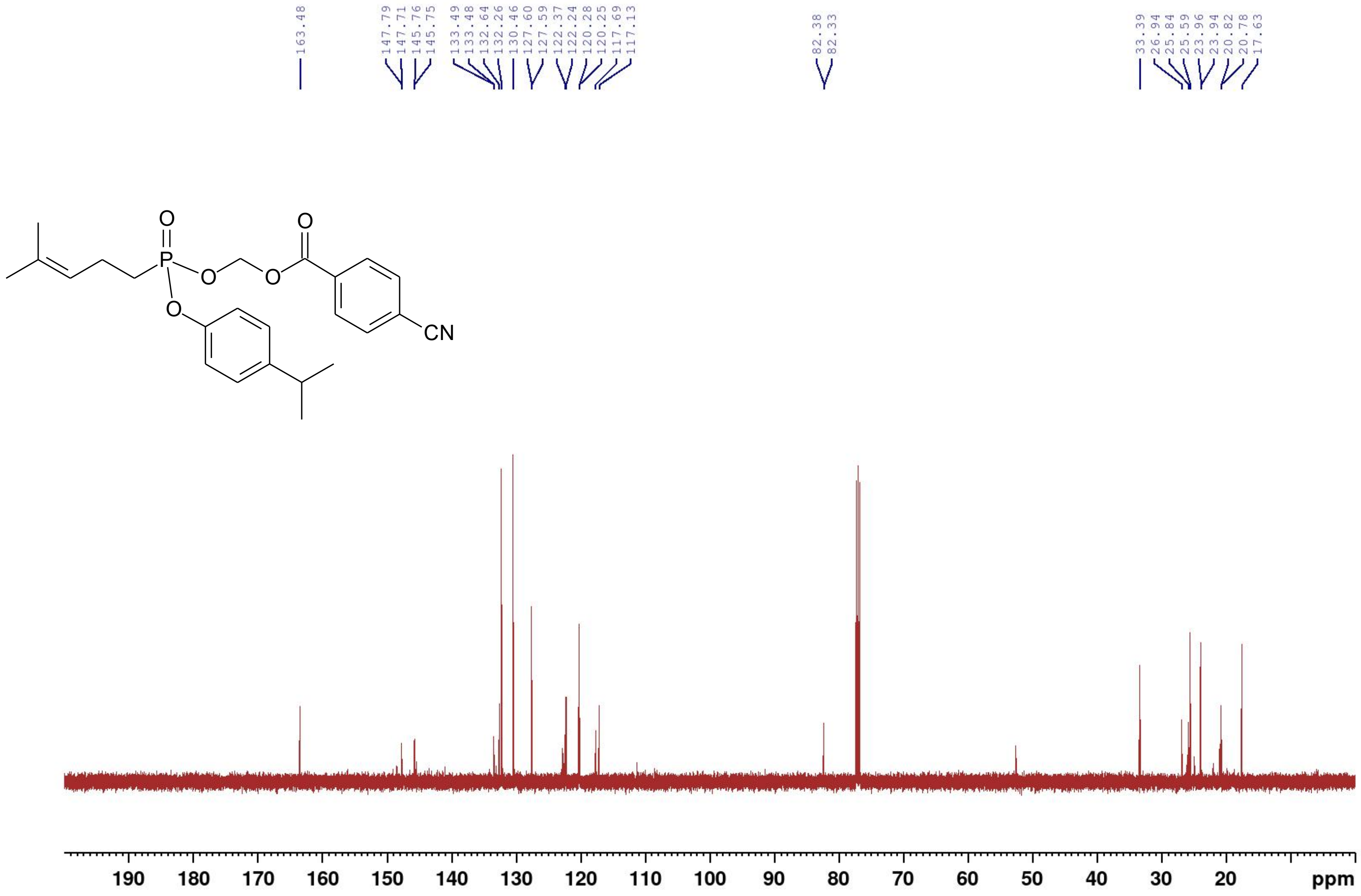

^{13}C NMR Spectrum of Compound **7f** ($CDCl_3$, 126 MHz)

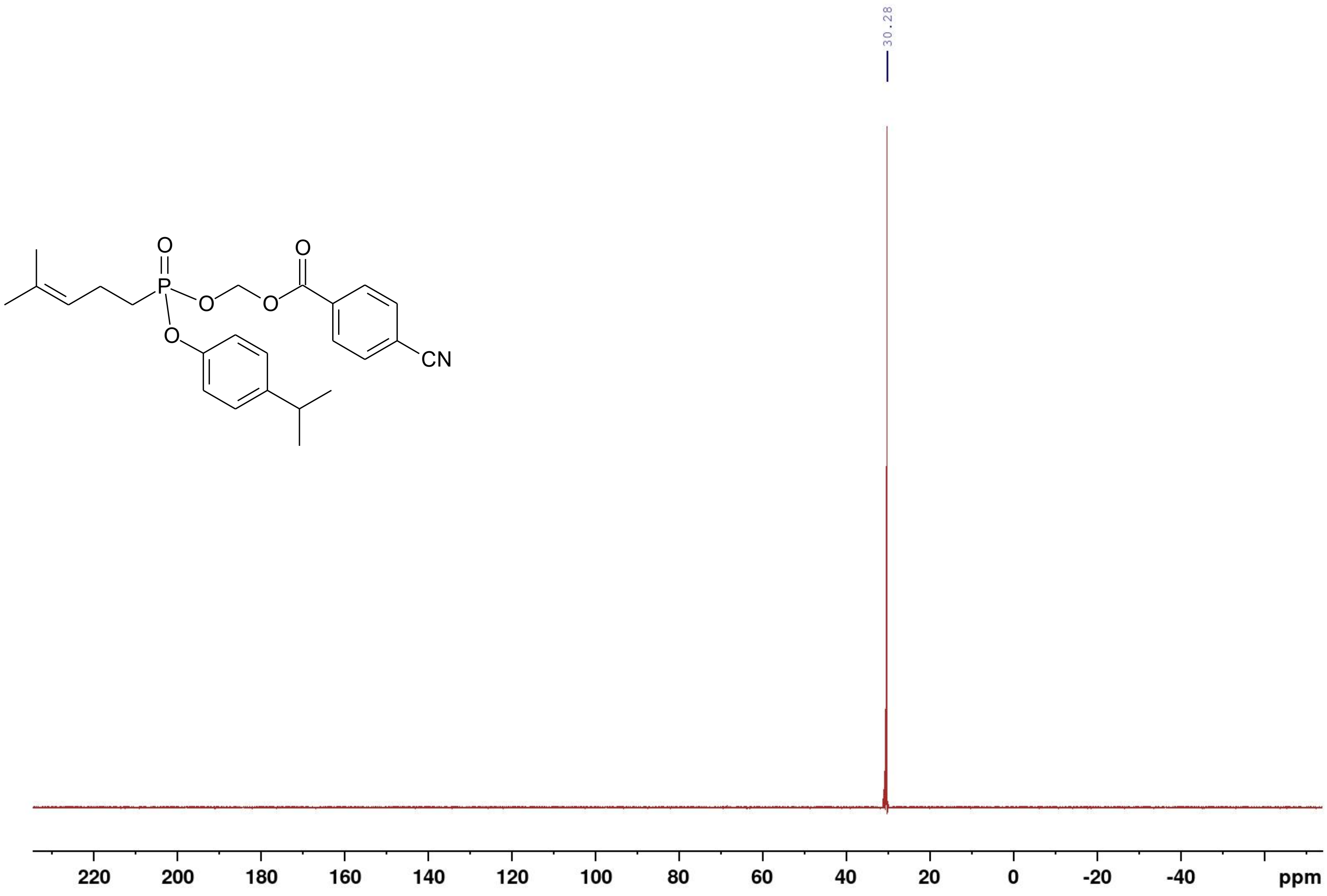

^{31}P NMR Spectrum of Compound **7f** ($CDCl_3$, 203 MHz)

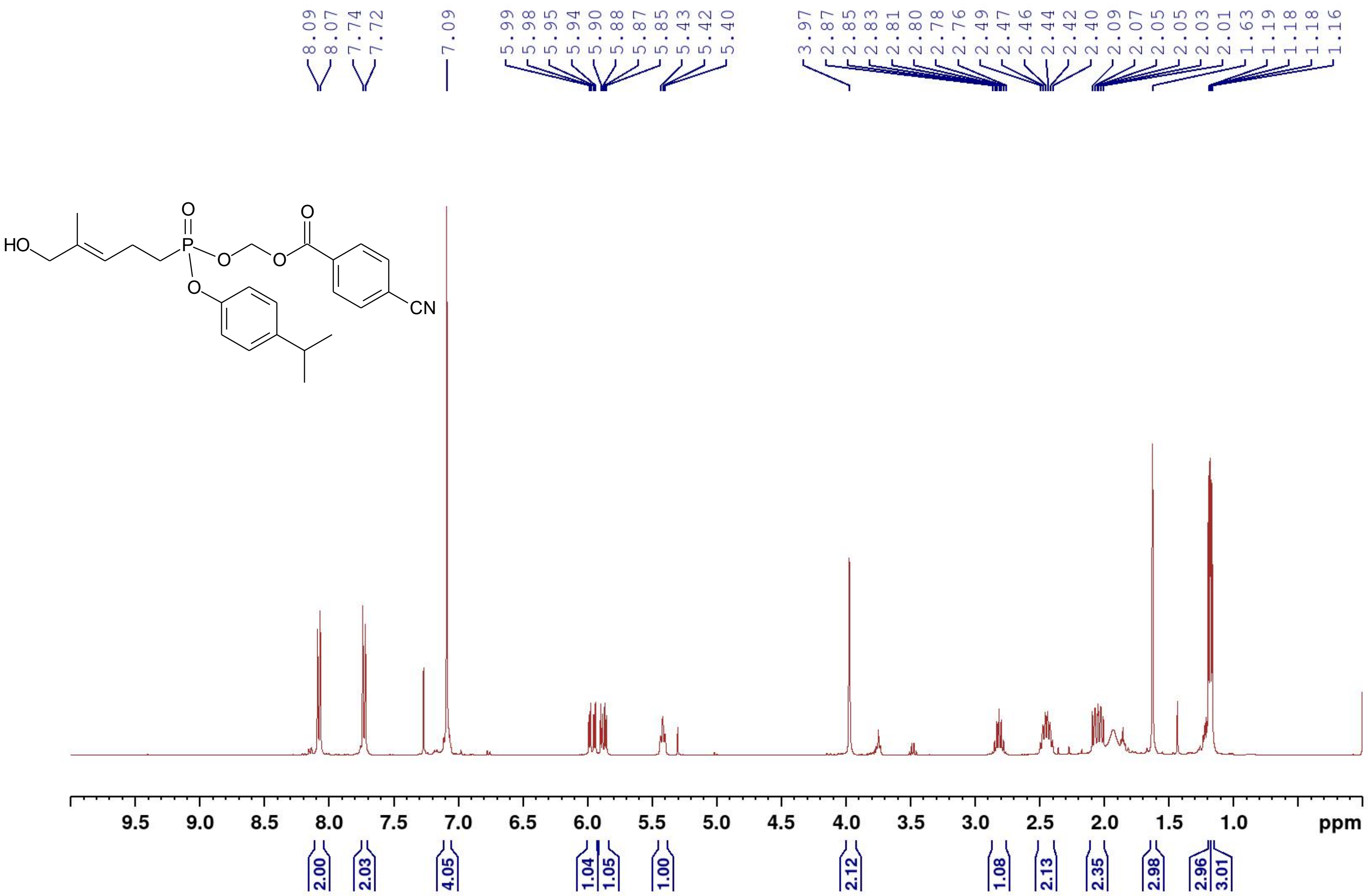

^{1}H NMR Spectrum of Compound **8f** ($CDCl_3$, 400 MHz)

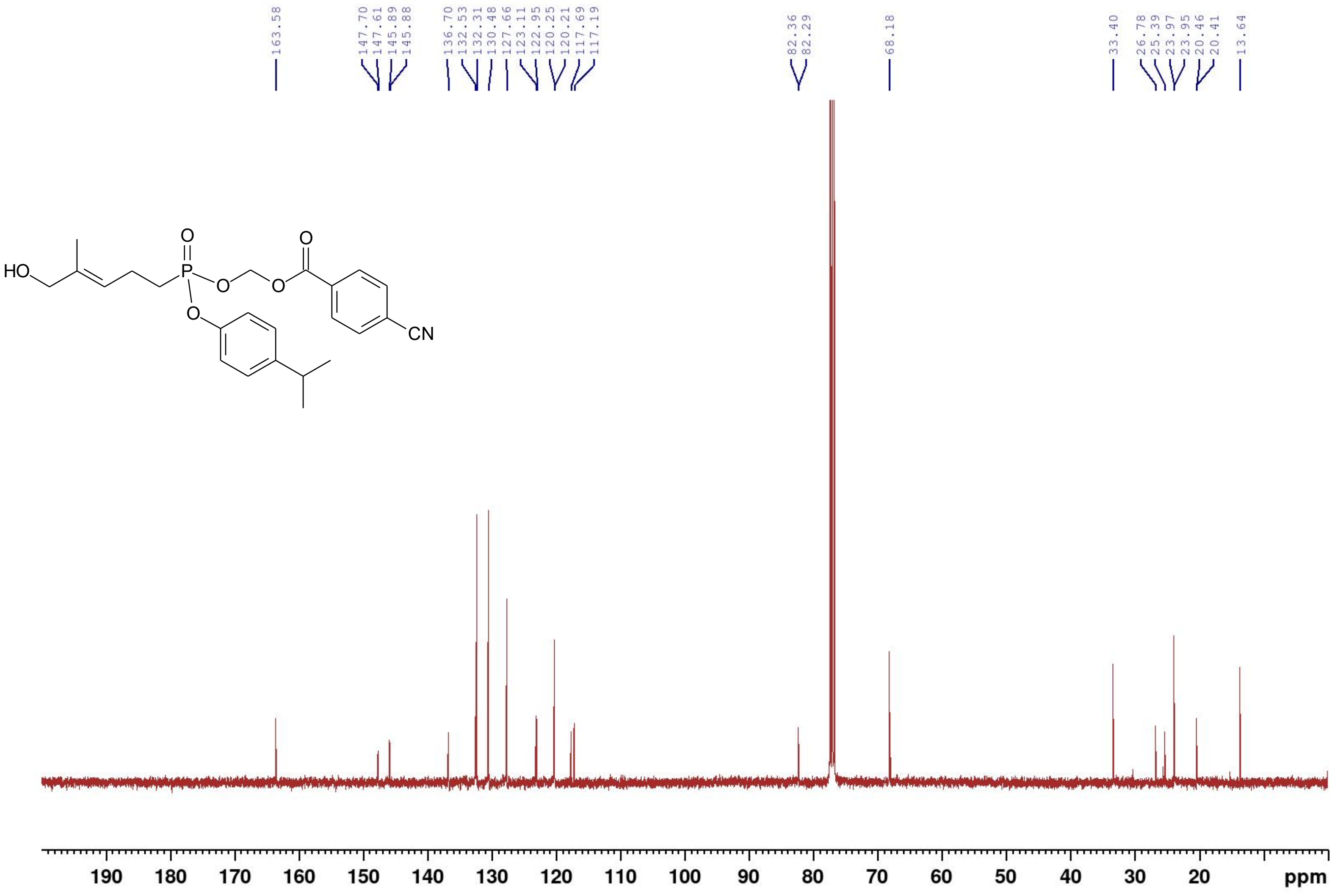

^{13}C NMR Spectrum of Compound **8f** ($CDCl_3$, 101 MHz)

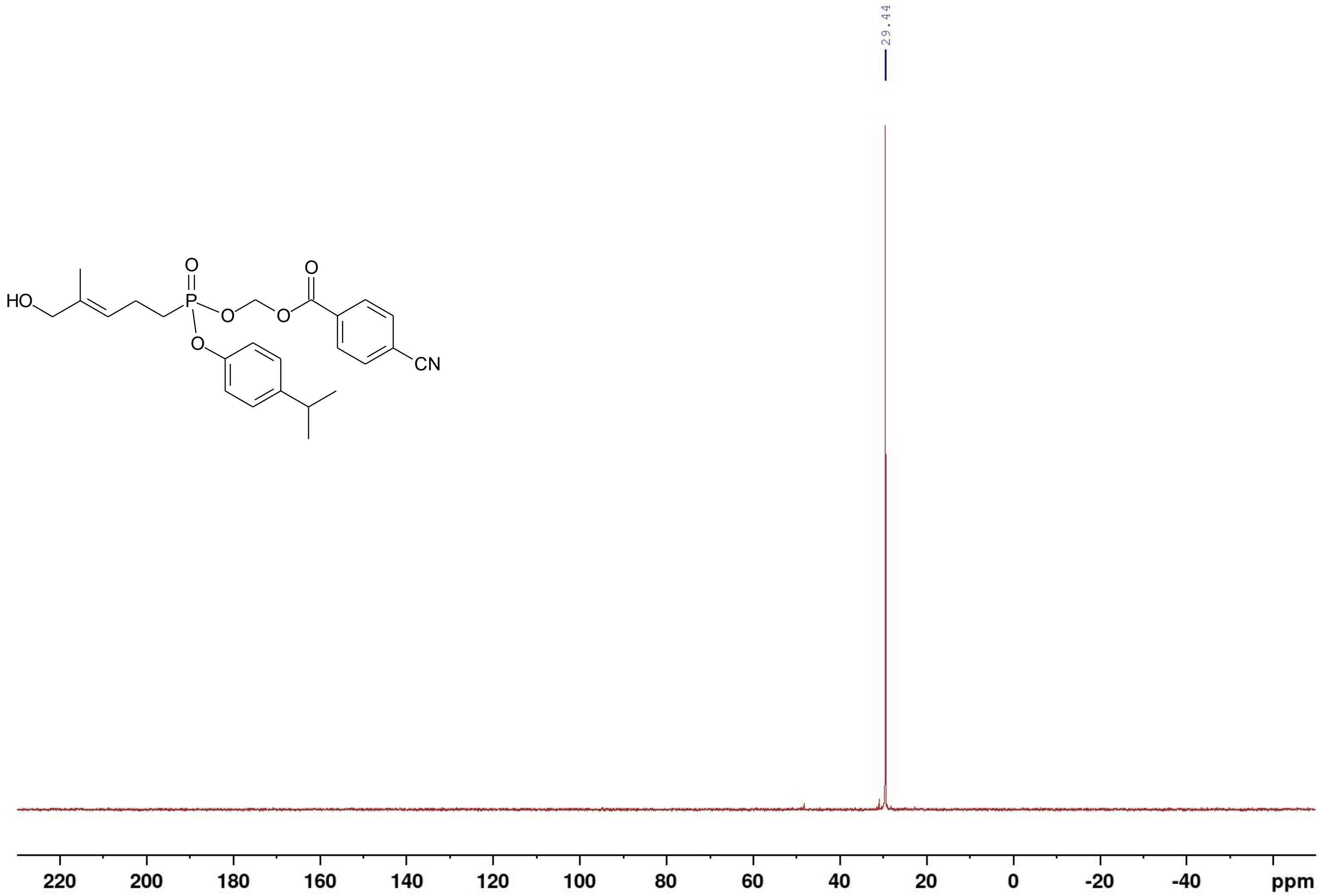

^{31}P NMR Spectrum of Compound **8f** ($CDCl_3$, 162 MHz)

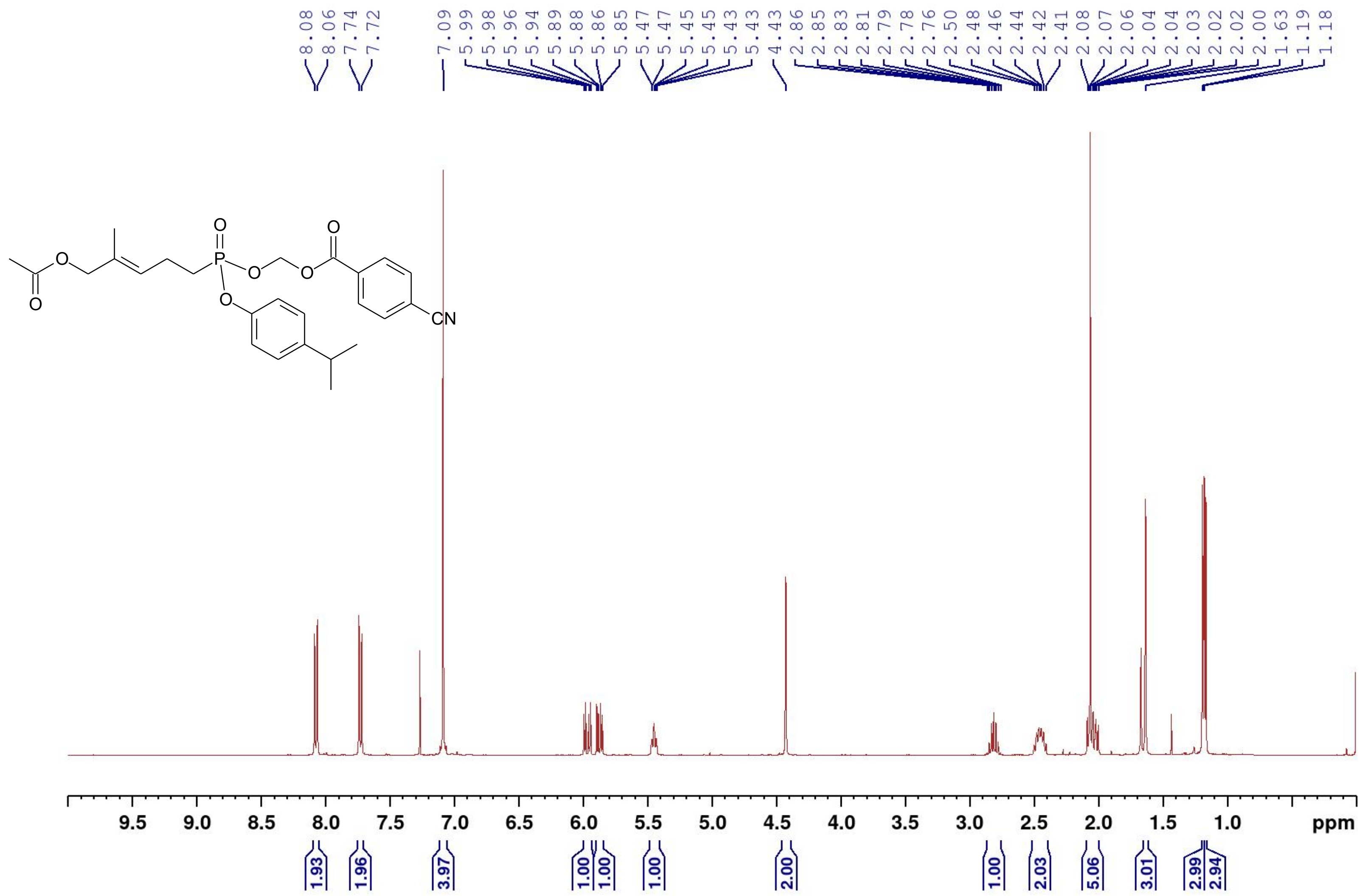

^{1}H NMR Spectrum of Compound **9f** ($CDCl_3$, 400 MHz)

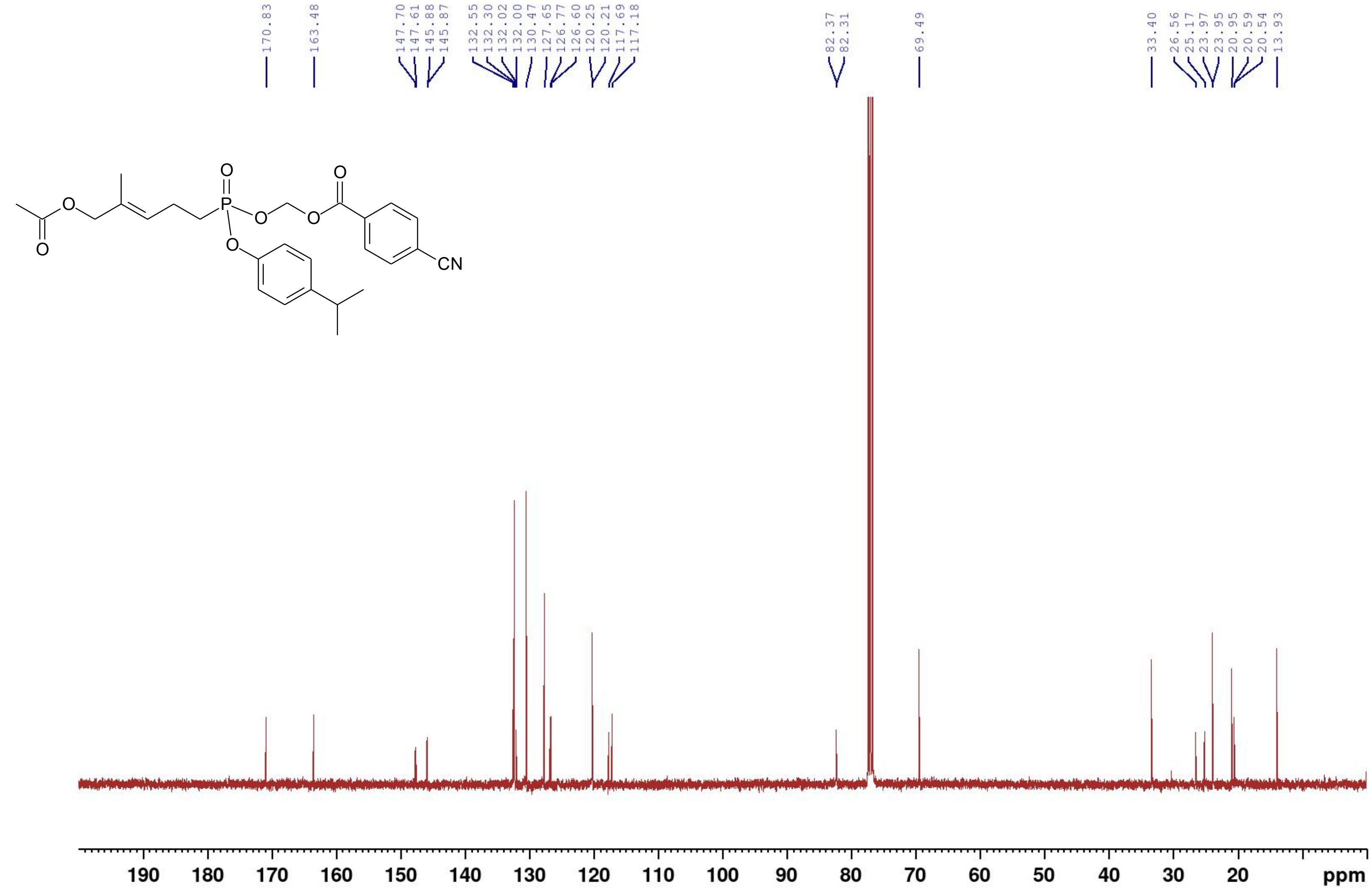

^{13}C NMR Spectrum of Compound **9f** ($CDCl_3$, 101 MHz)

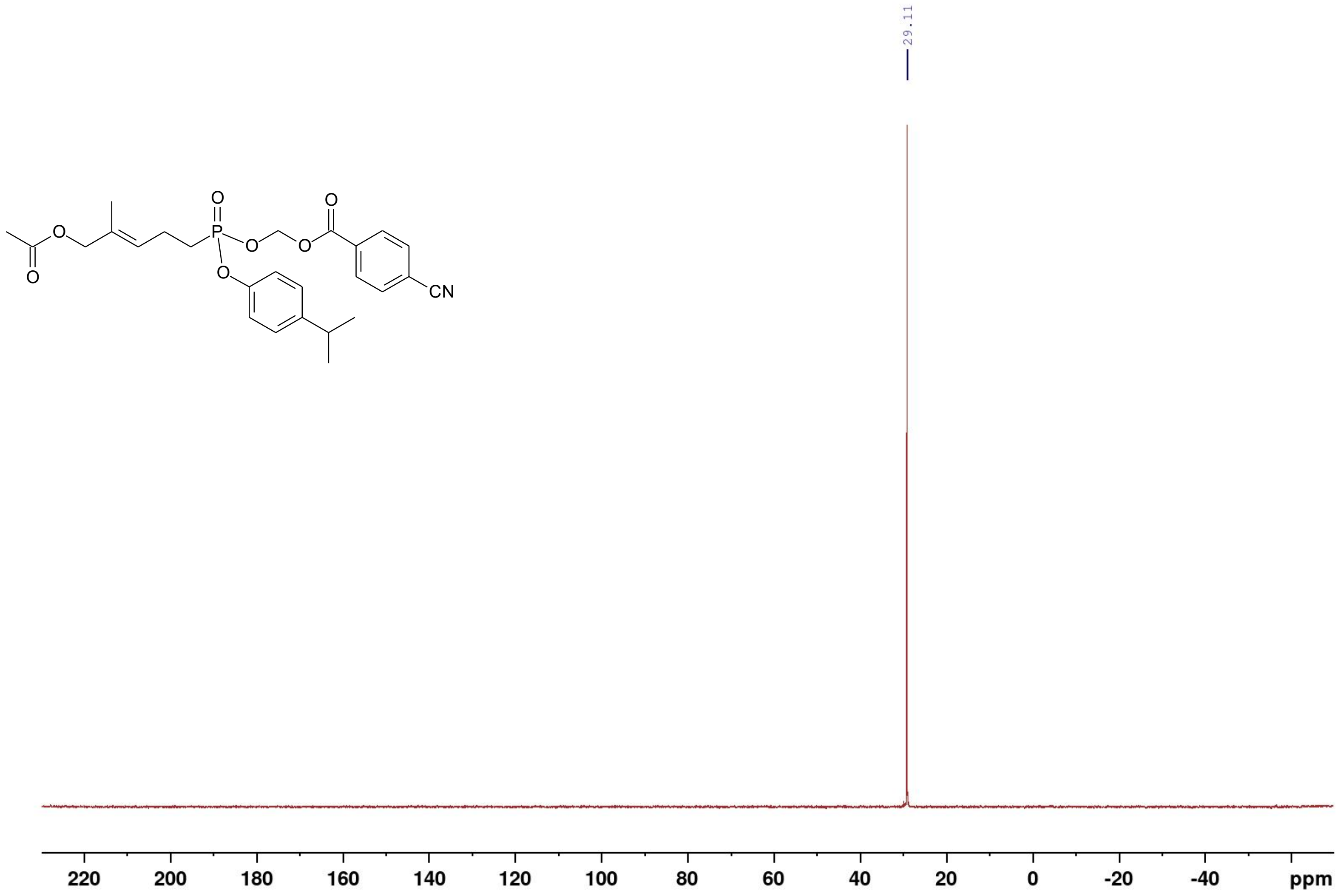

^{31}P NMR Spectrum of Compound **9f** ($CDCl_3$, 162 MHz)

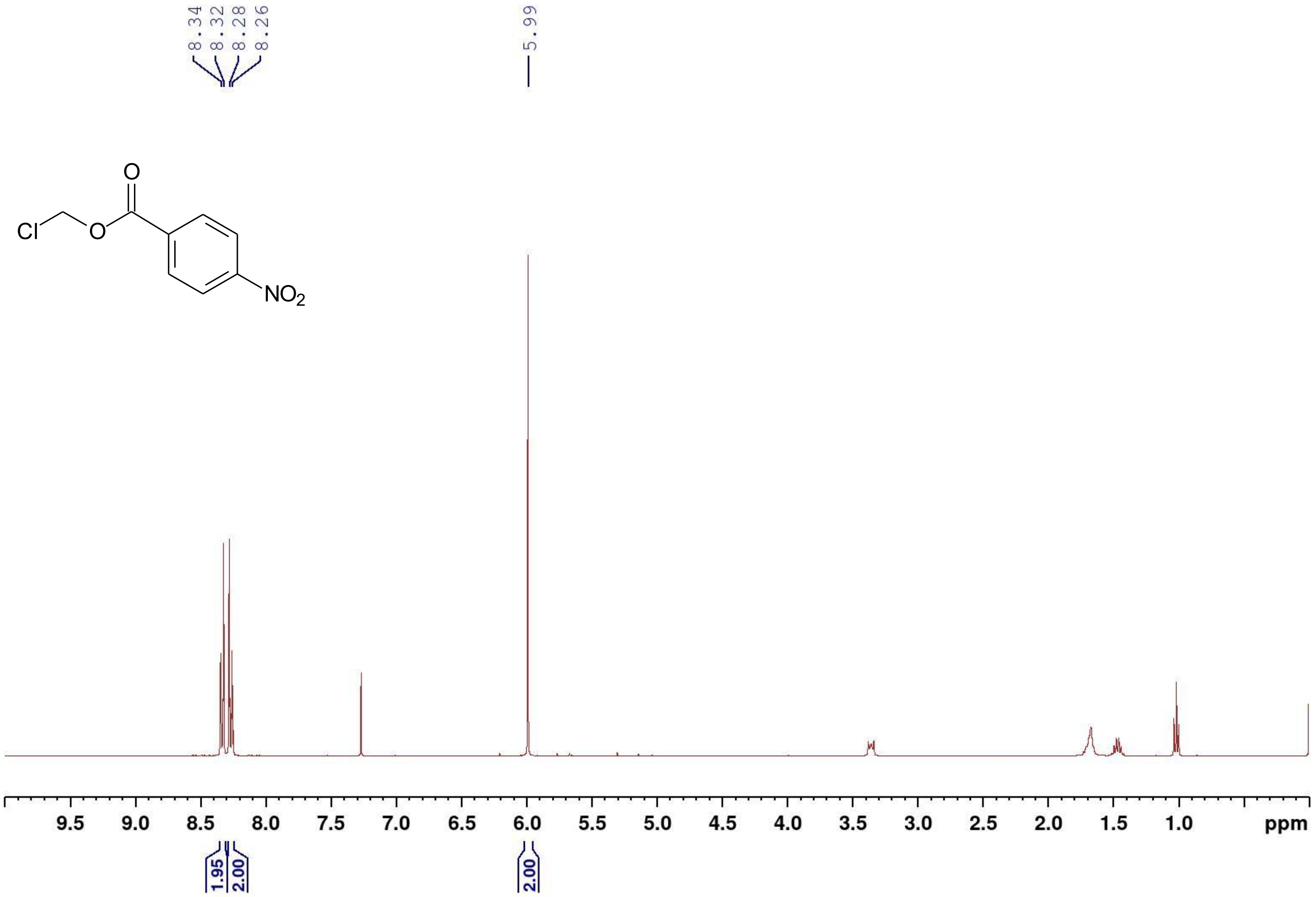

^{1}H NMR Spectrum of Compound **5g** ($CDCl_3$, 400 MHz)

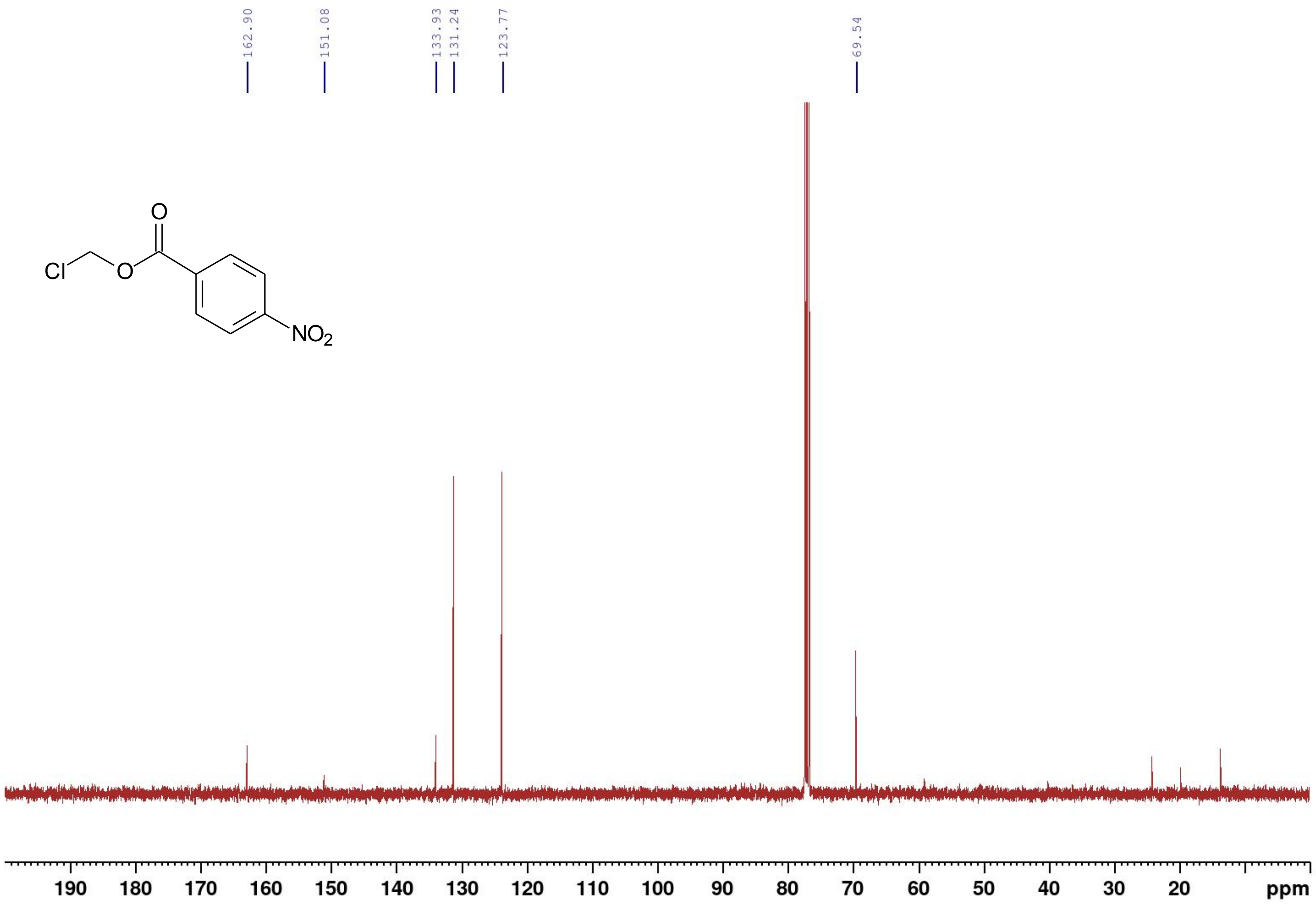

^{13}C NMR Spectrum of Compound **5g** ($CDCl_3$, 101 MHz)

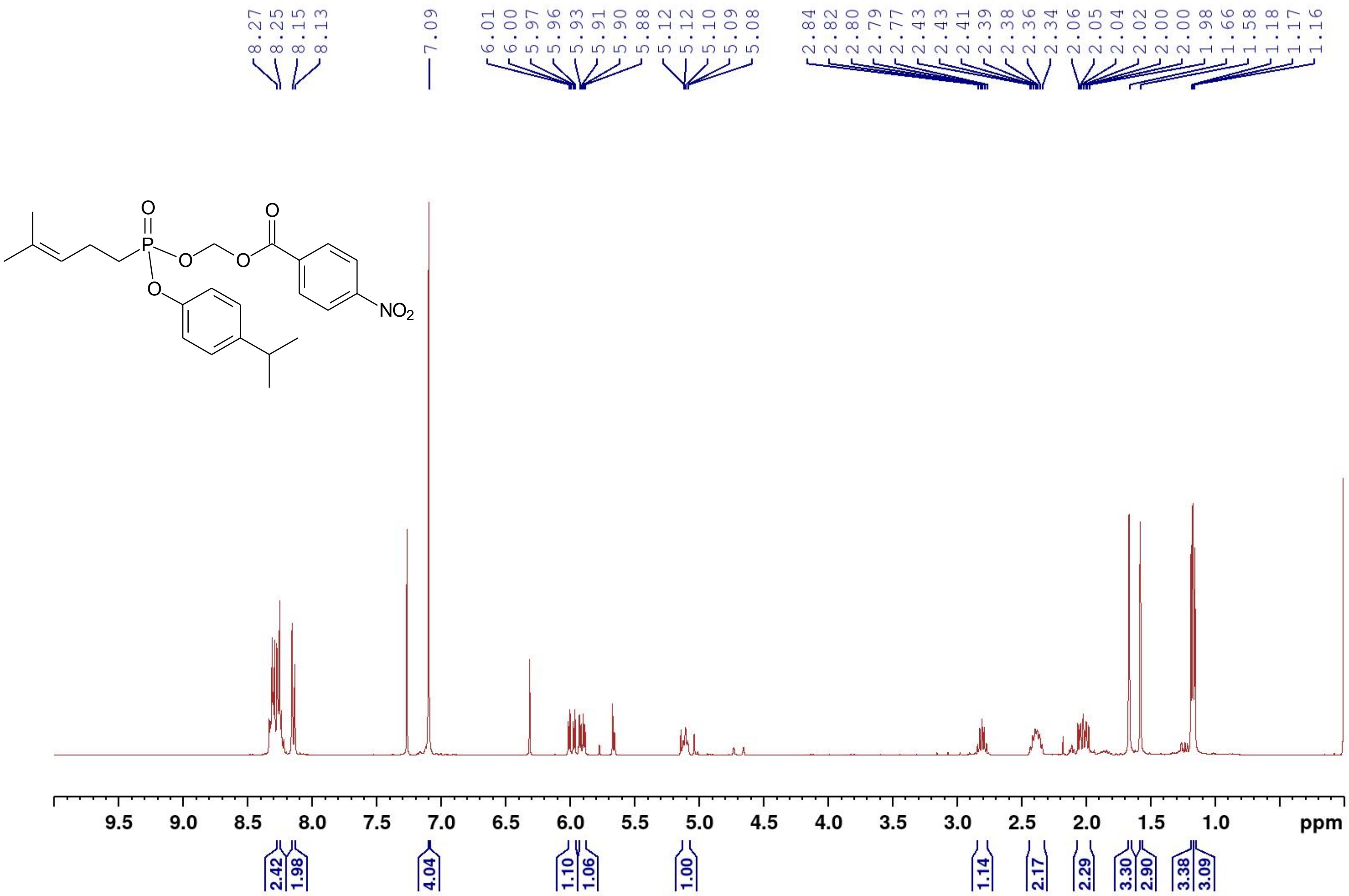

^{1}H NMR Spectrum of Compound **7g** ($CDCl_3$, 400 MHz)

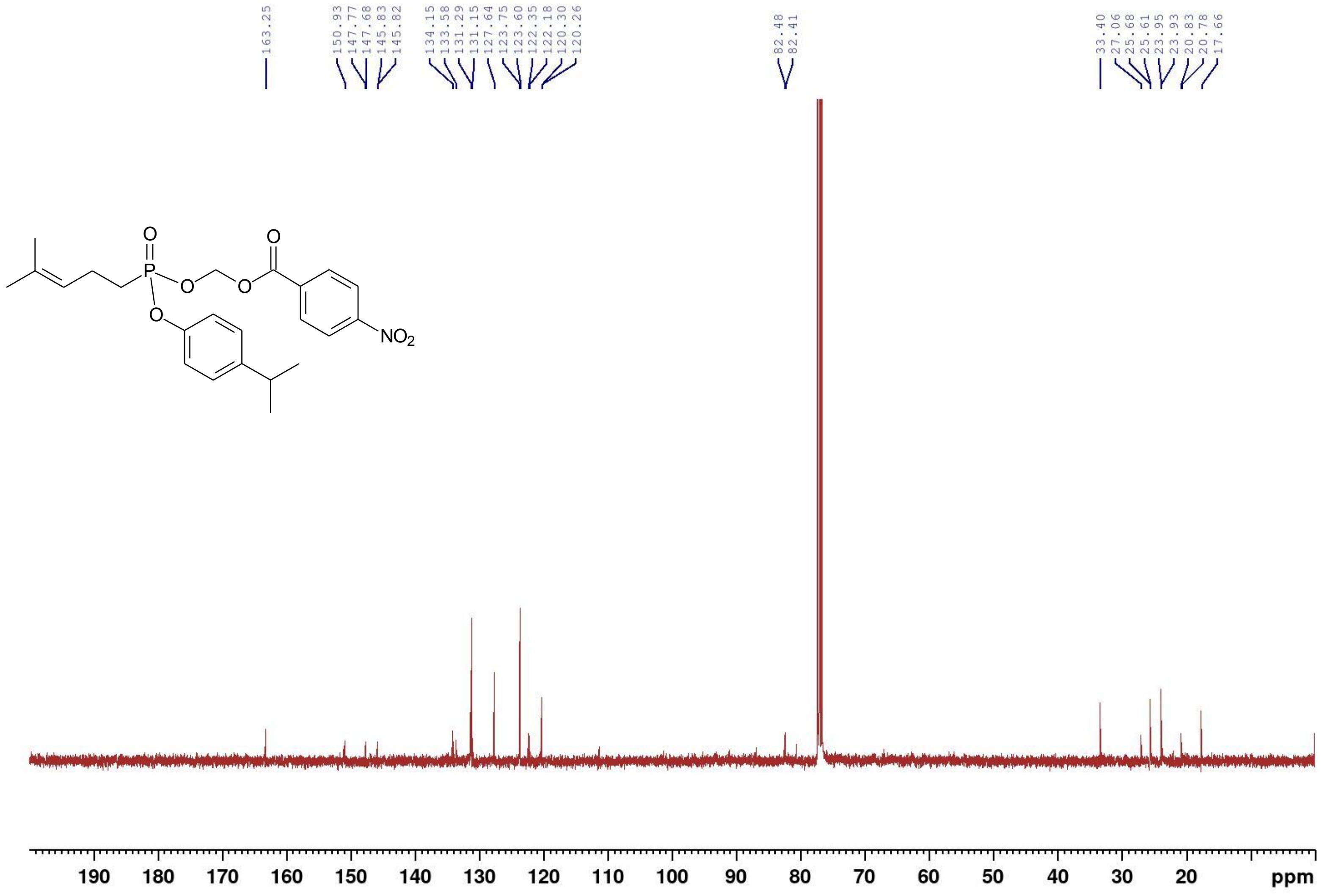

^{13}C NMR Spectrum of Compound **7g** ($CDCl_3$, 101 MHz)

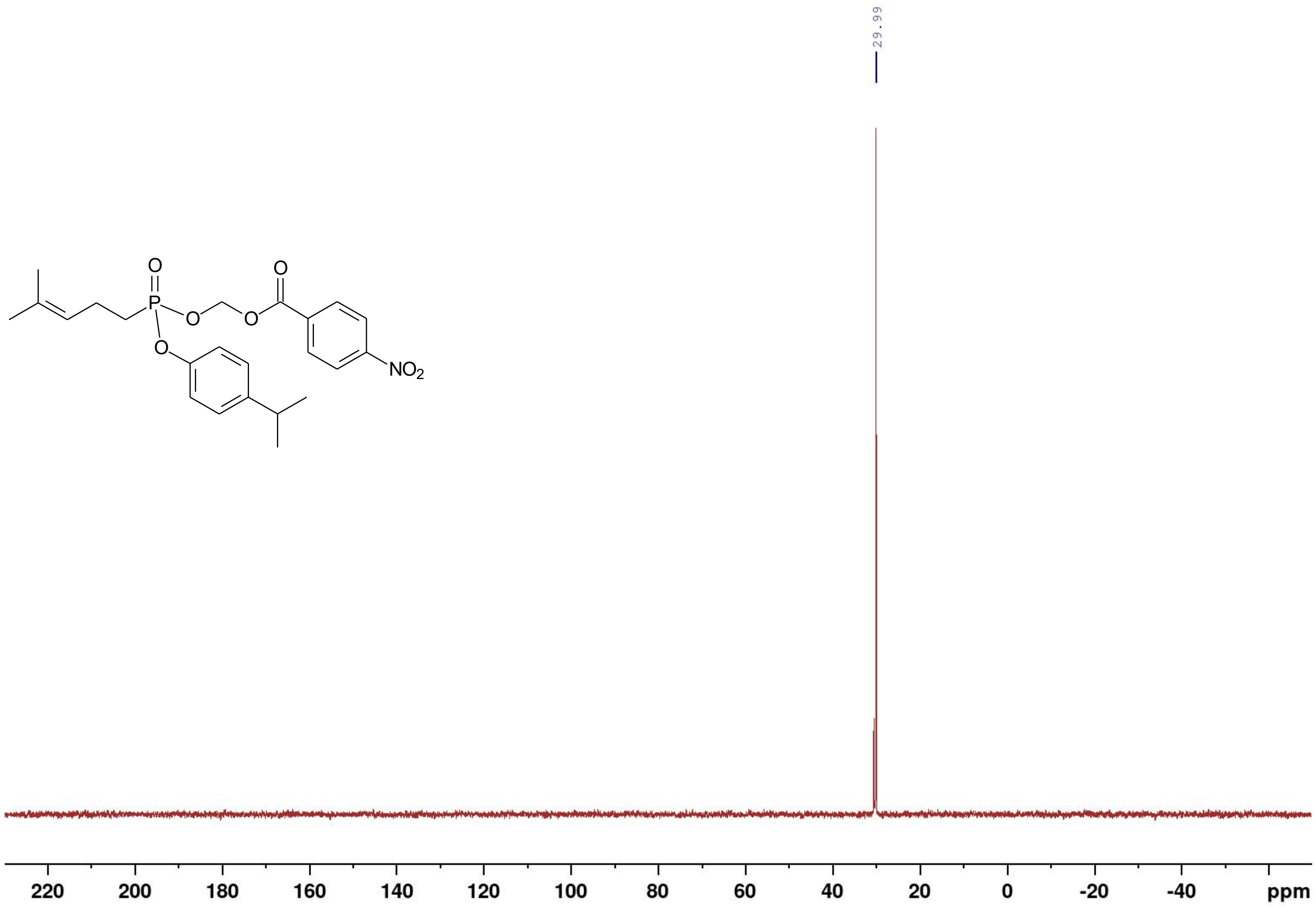

^{31}P NMR Spectrum of Compound **7g** ($CDCl_3$, 162 MHz)

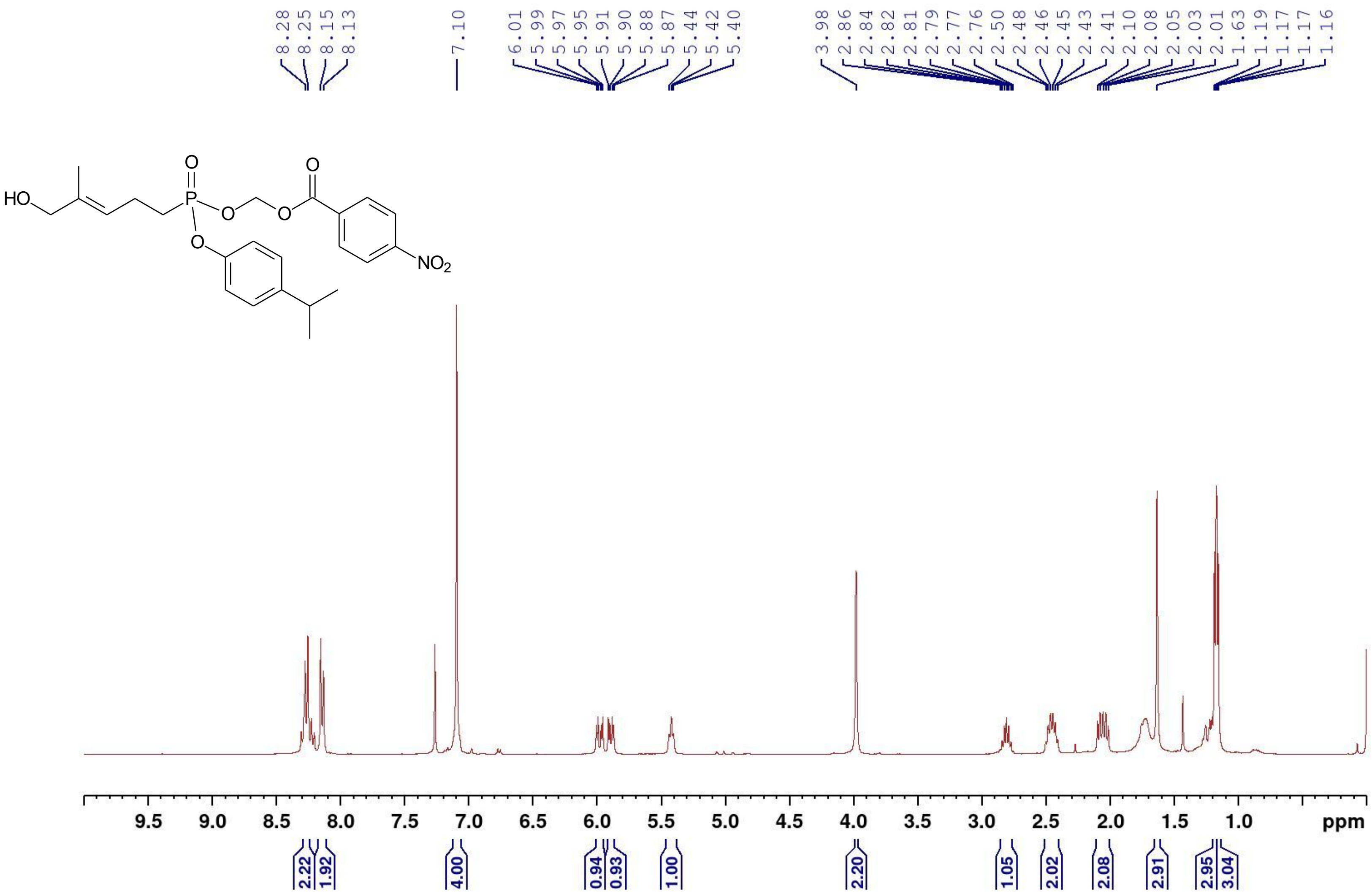

^{1}H NMR Spectrum of Compound **8g** ($CDCl_3$, 400 MHz)

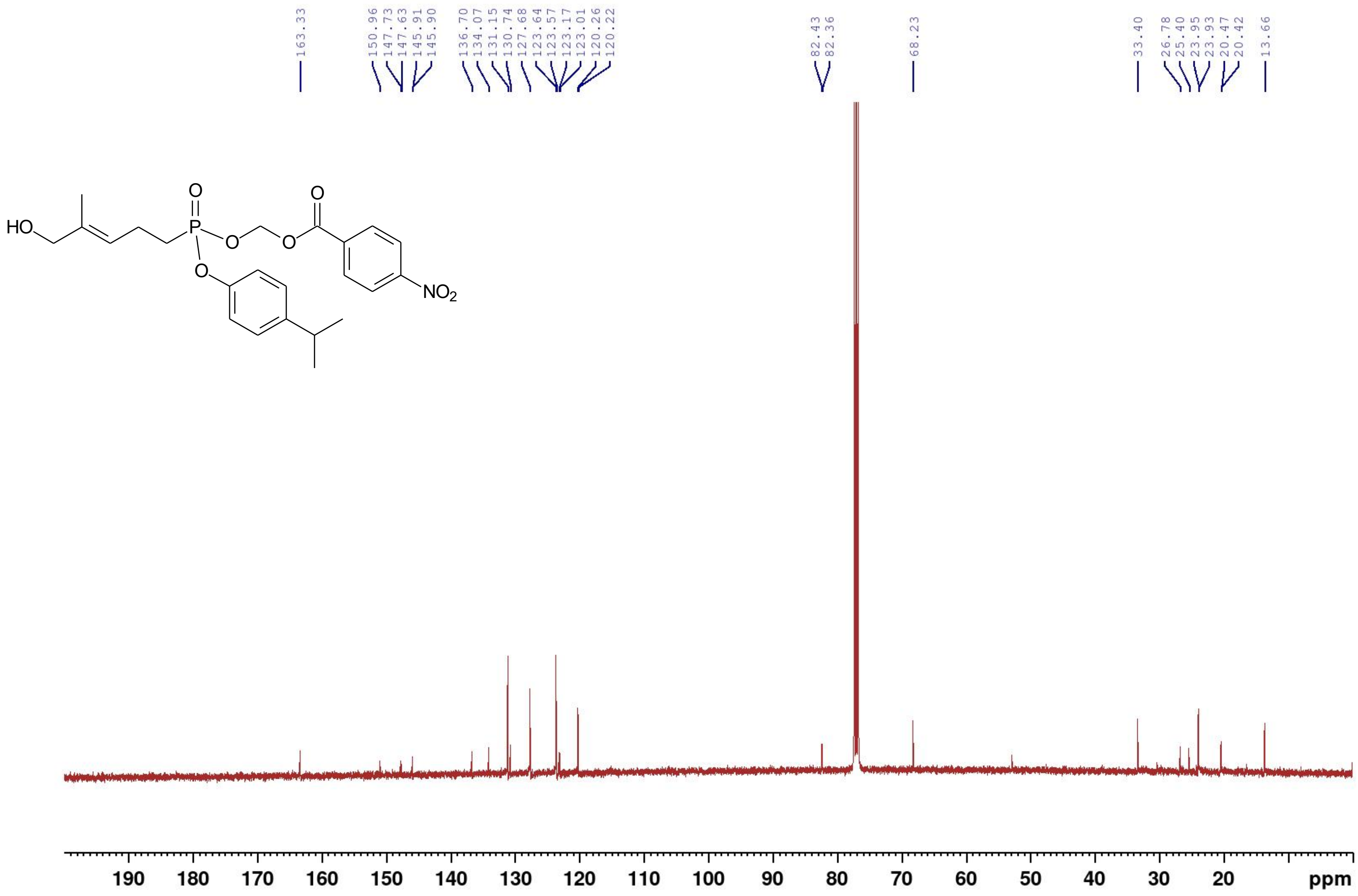

^{13}C NMR Spectrum of Compound **8g** ($CDCl_3$, 101 MHz)

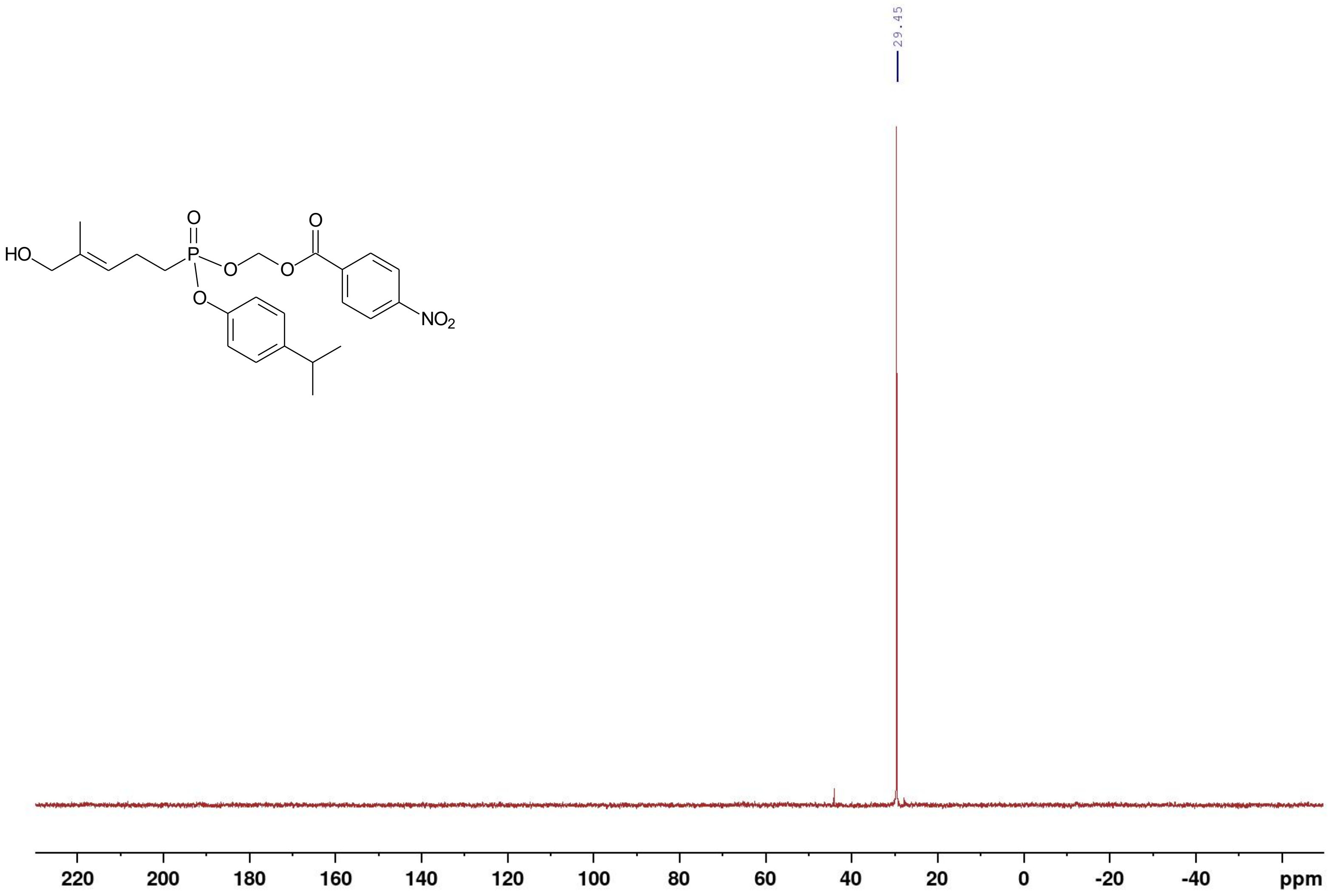

^{31}P NMR Spectrum of Compound **8g** ($CDCl_3$, 162 MHz)

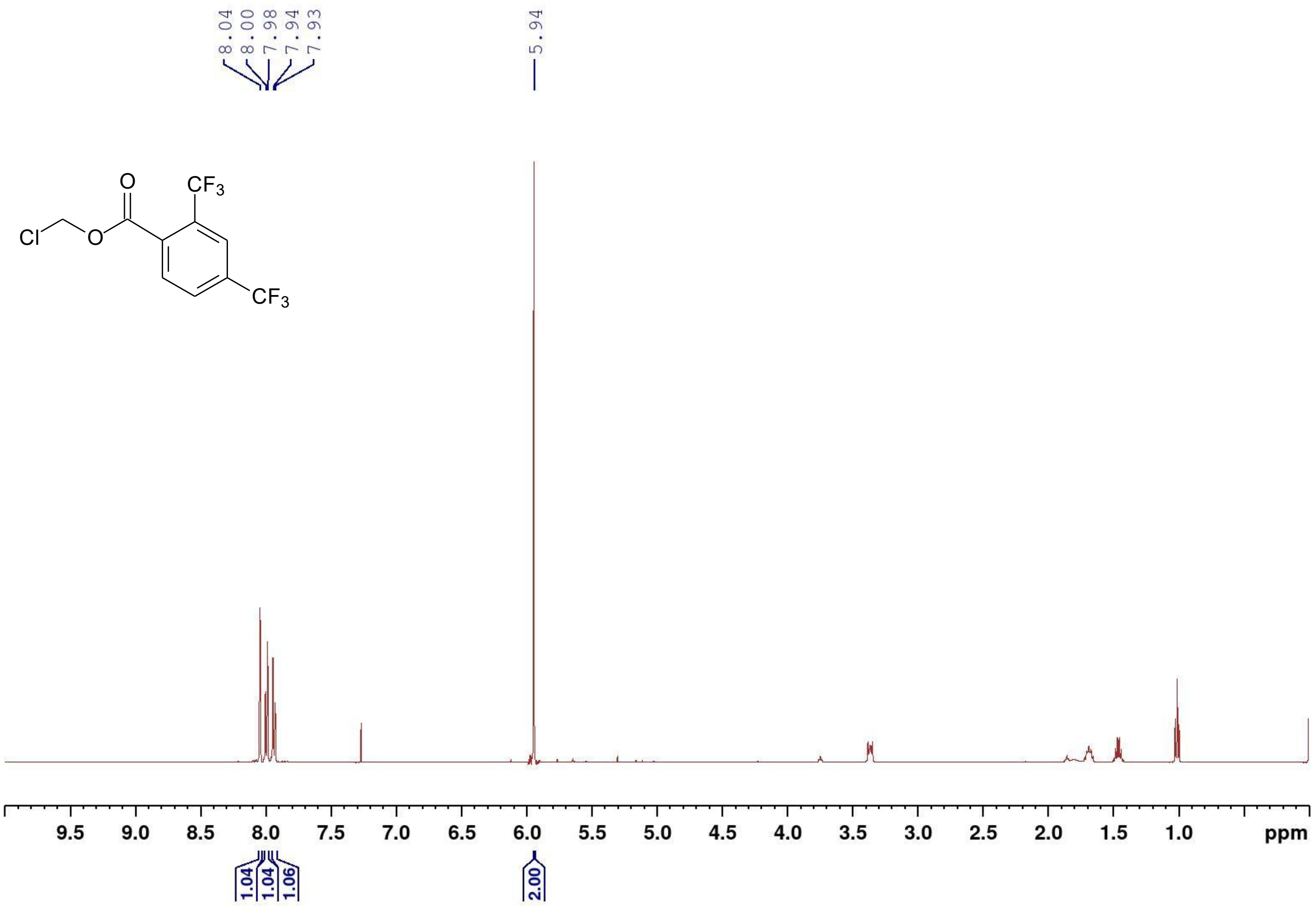

^{1}H NMR Spectrum of Compound **5h** ($CDCl_3$, 500 MHz)

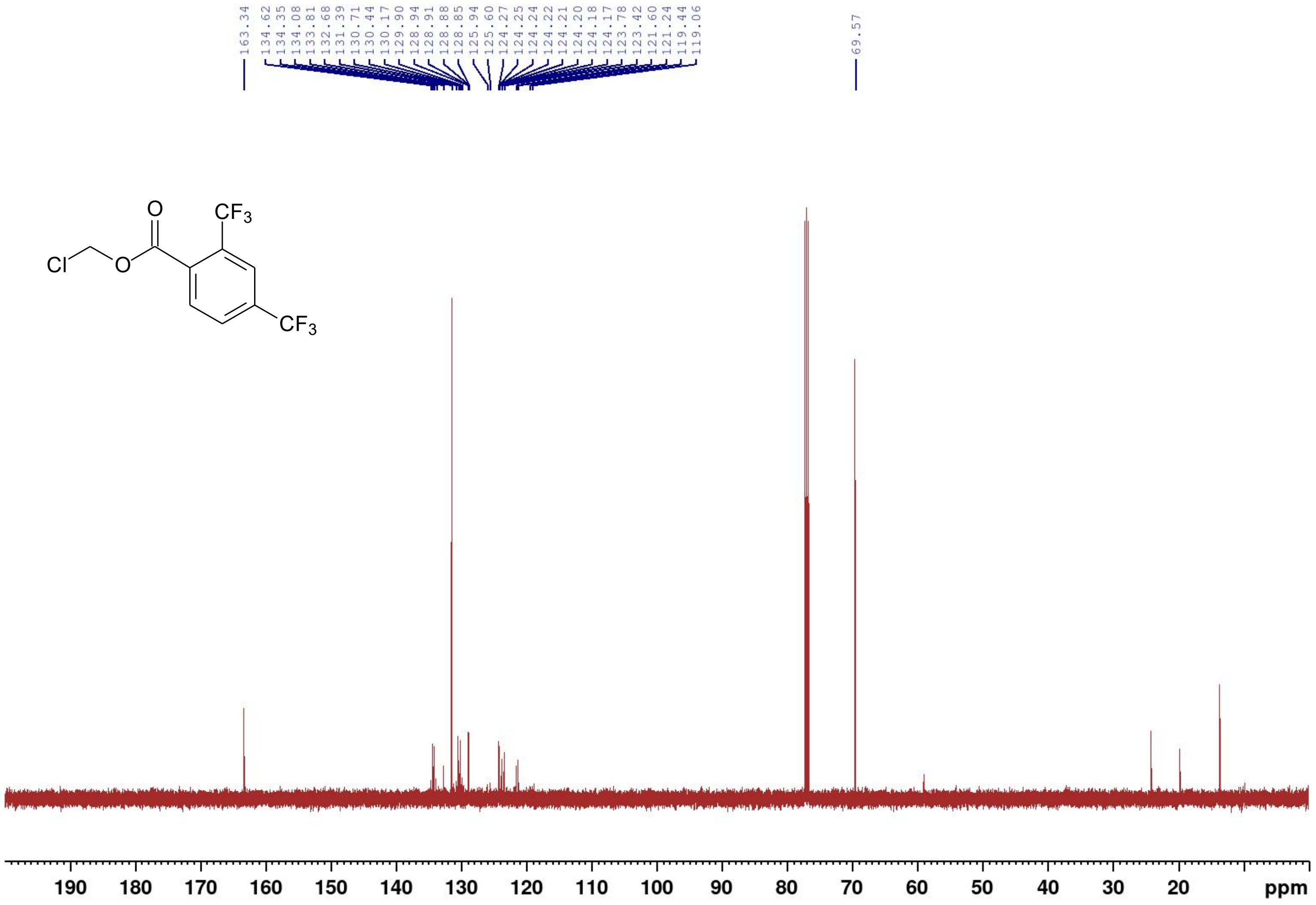

^{13}C NMR Spectrum of Compound **5h** ($CDCl_3$, 126 MHz)

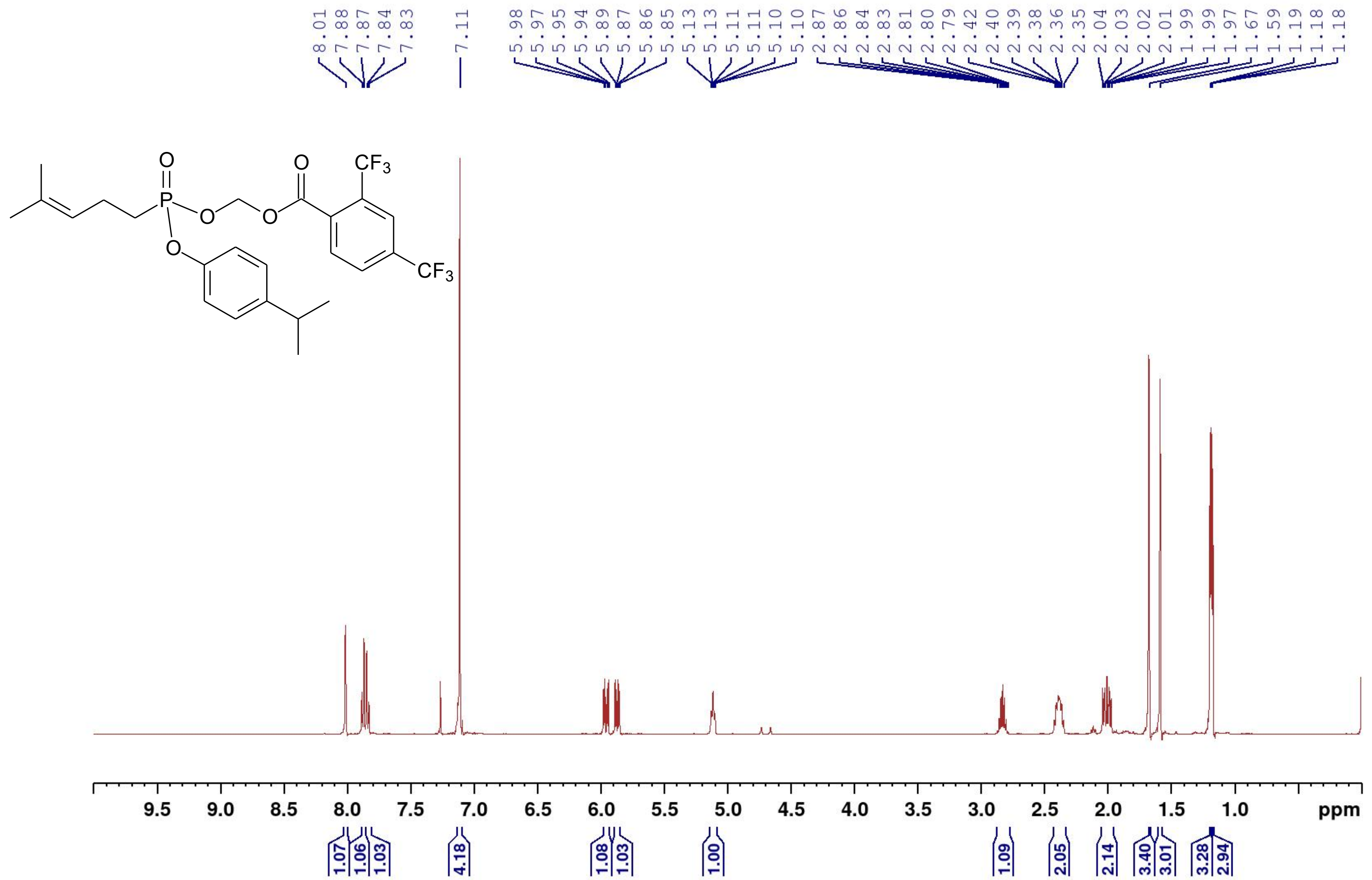

^{1}H NMR Spectrum of Compound **7h** ($CDCl_3$, 500 MHz)

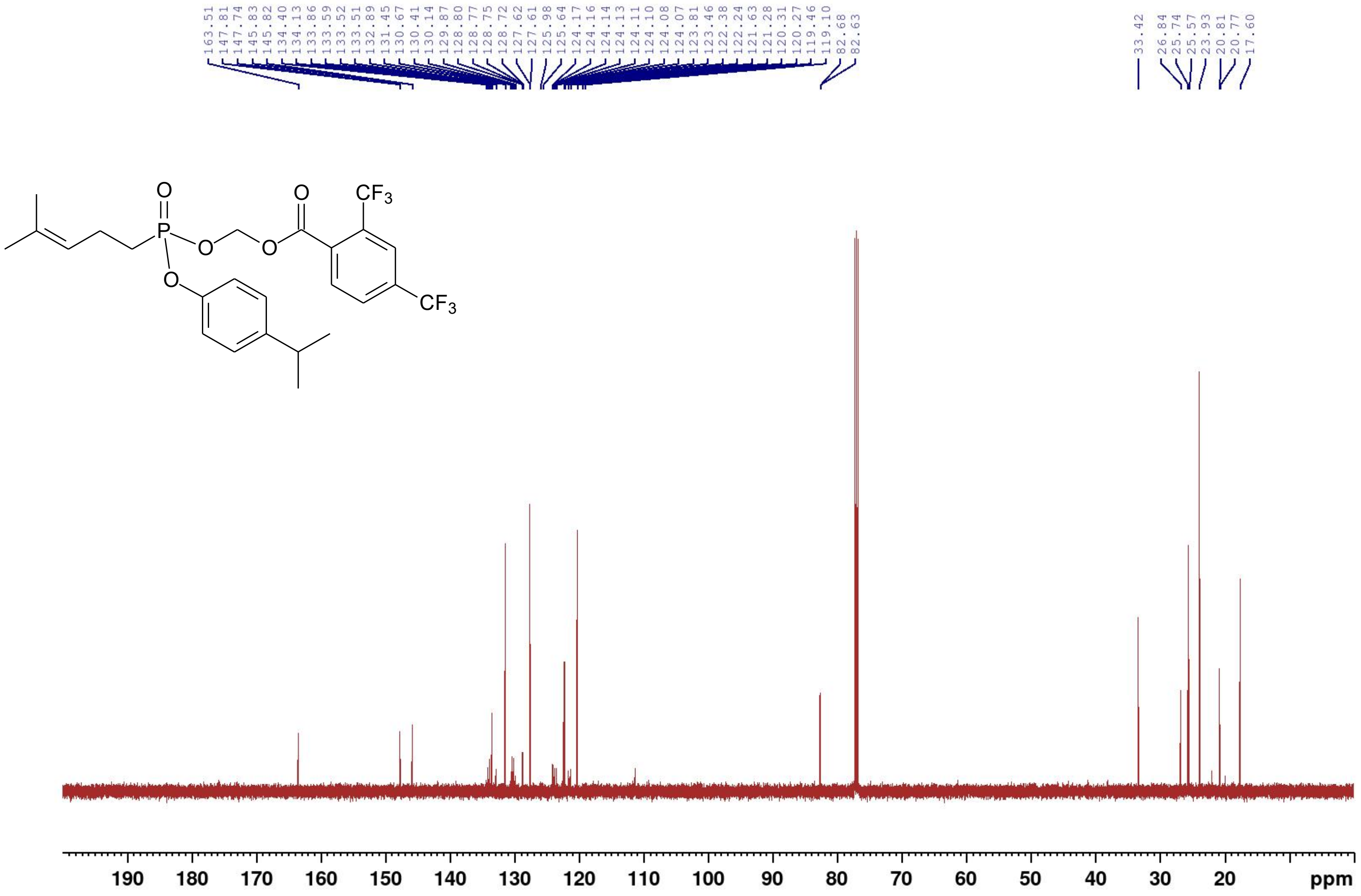

^{13}C NMR Spectrum of Compound **7h** ($CDCl_3$, 126 MHz)

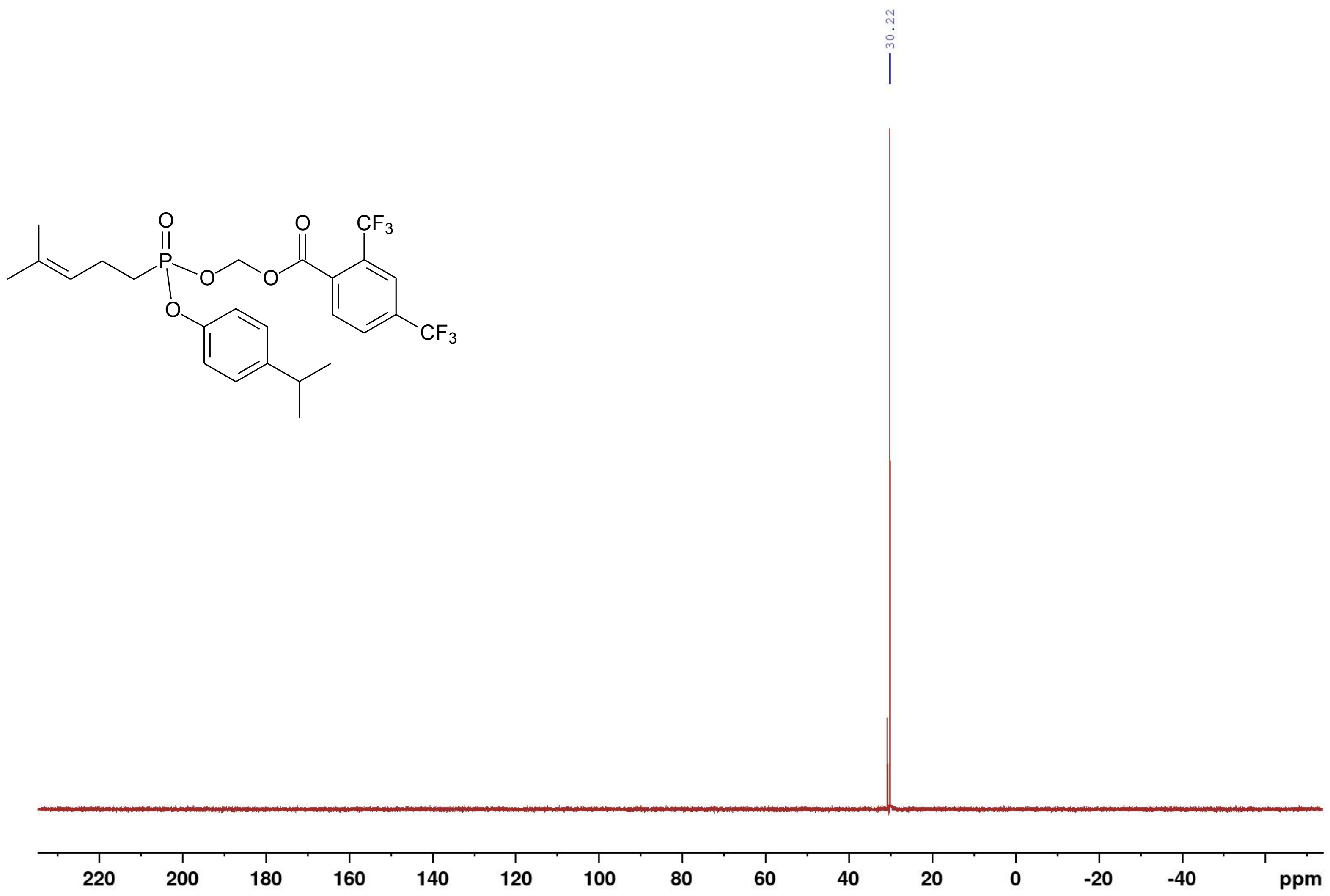

^{31}P NMR Spectrum of Compound **7h** ($CDCl_3$, 203 MHz)

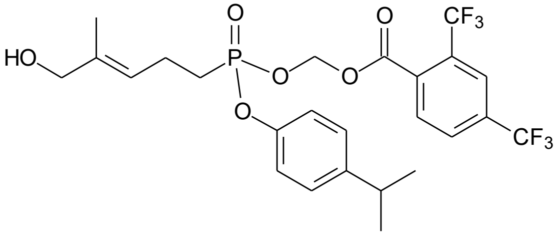

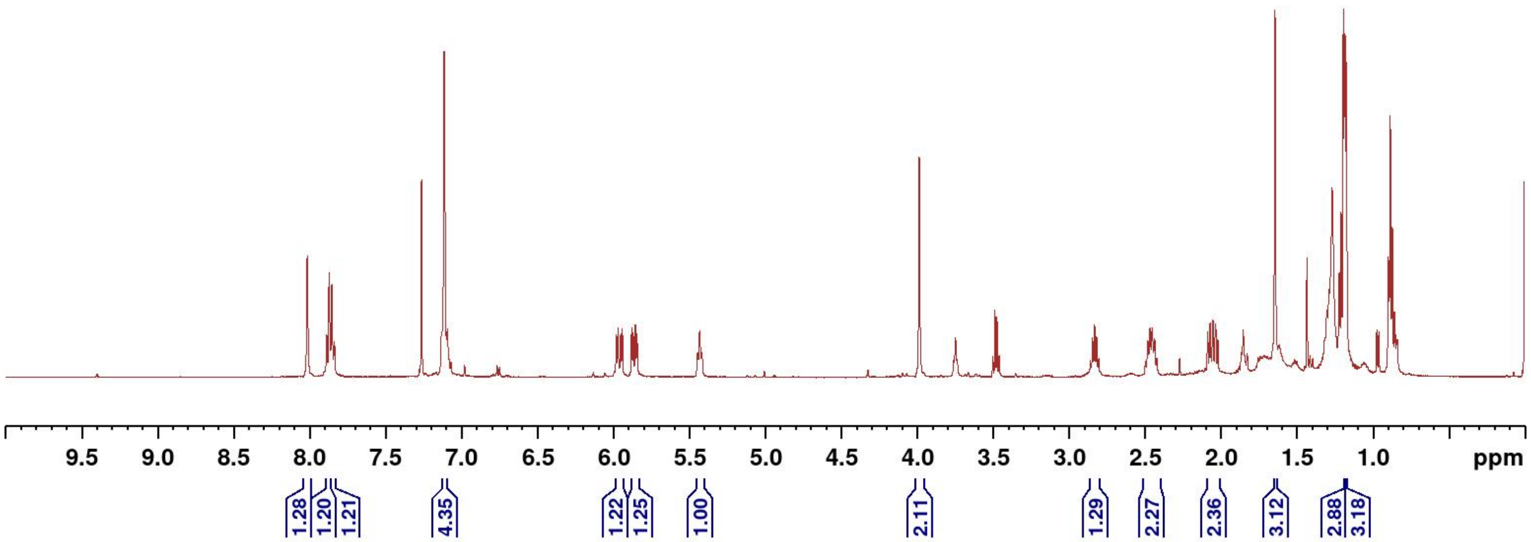

^{1}H NMR Spectrum of Compound **8h** ($CDCl_3$, 500 MHz)

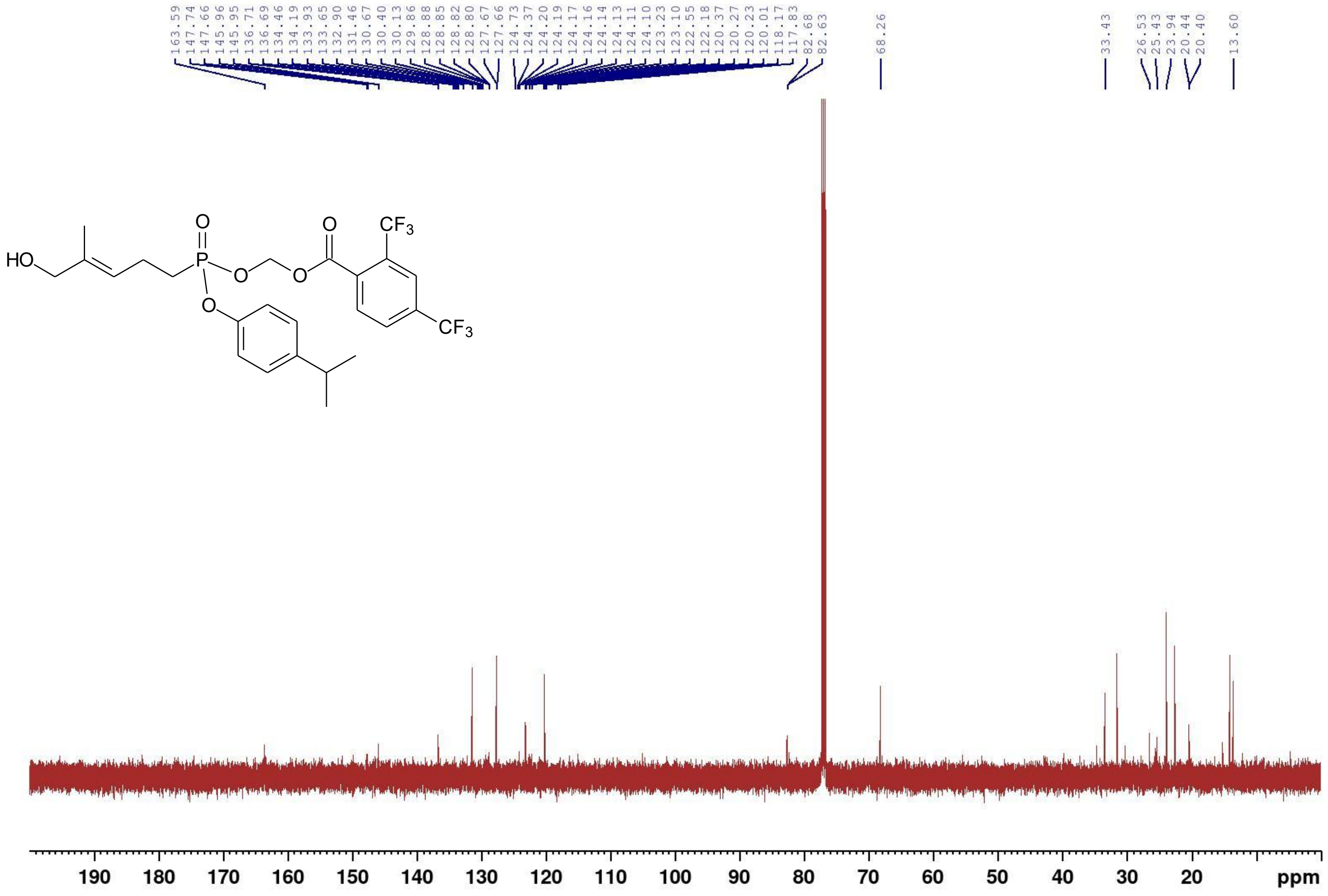

^{13}C NMR Spectrum of Compound **8h** ($CDCl_3$, 126 MHz)

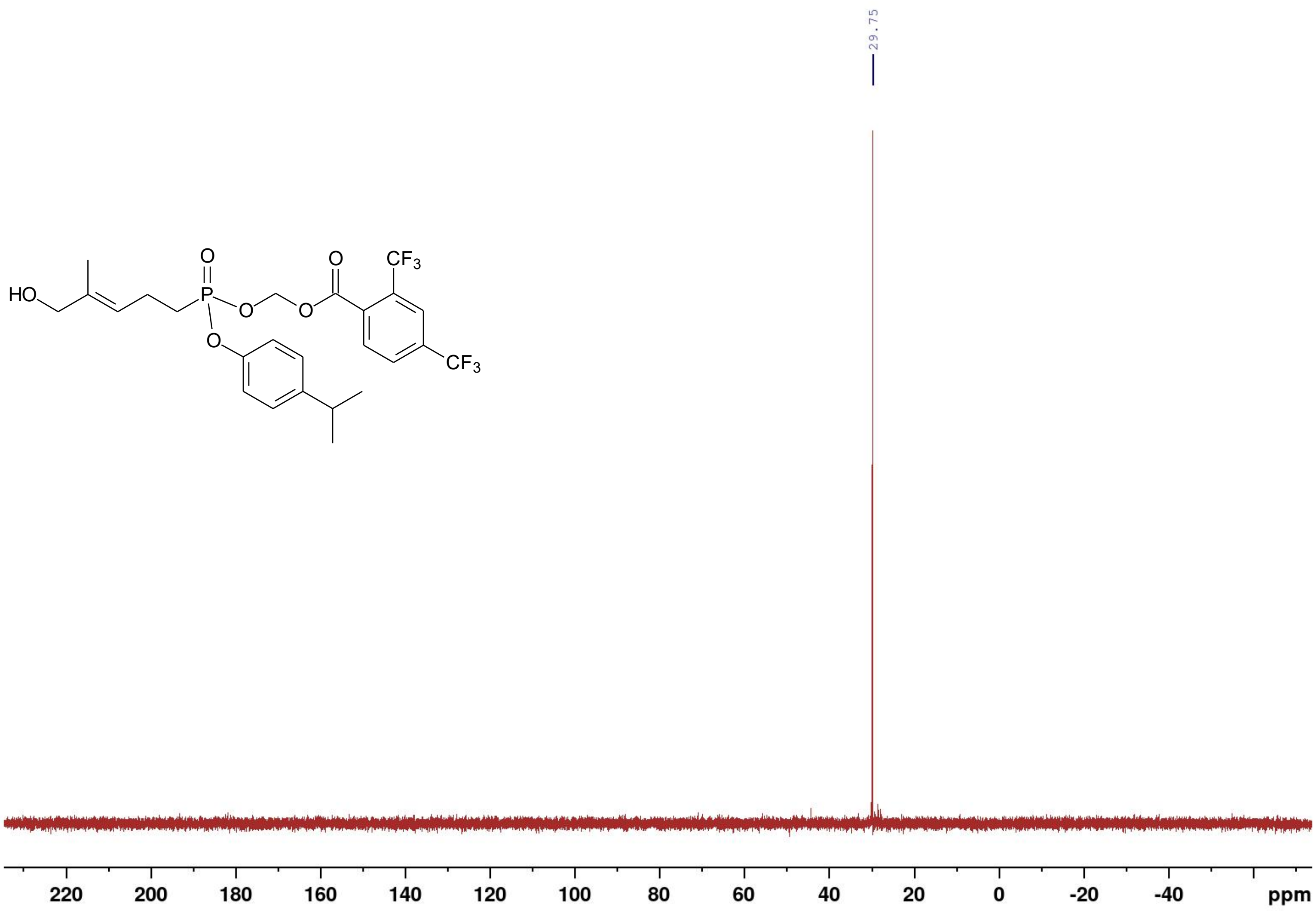

^{31}P NMR Spectrum of Compound **8h** ($CDCl_3$, 203 MHz)

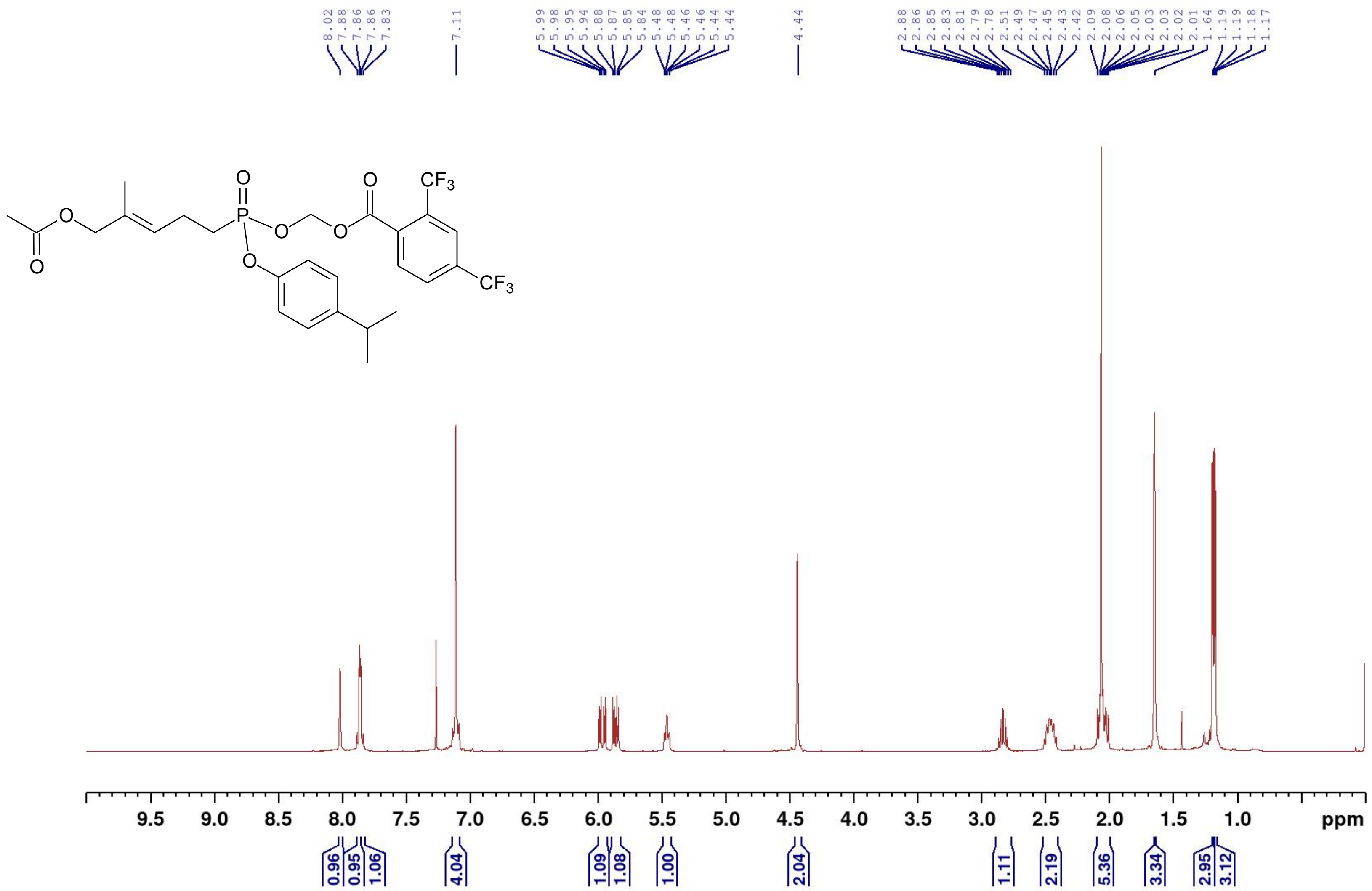

^{1}H NMR Spectrum of Compound **9h** ($CDCl_3$, 400 MHz)

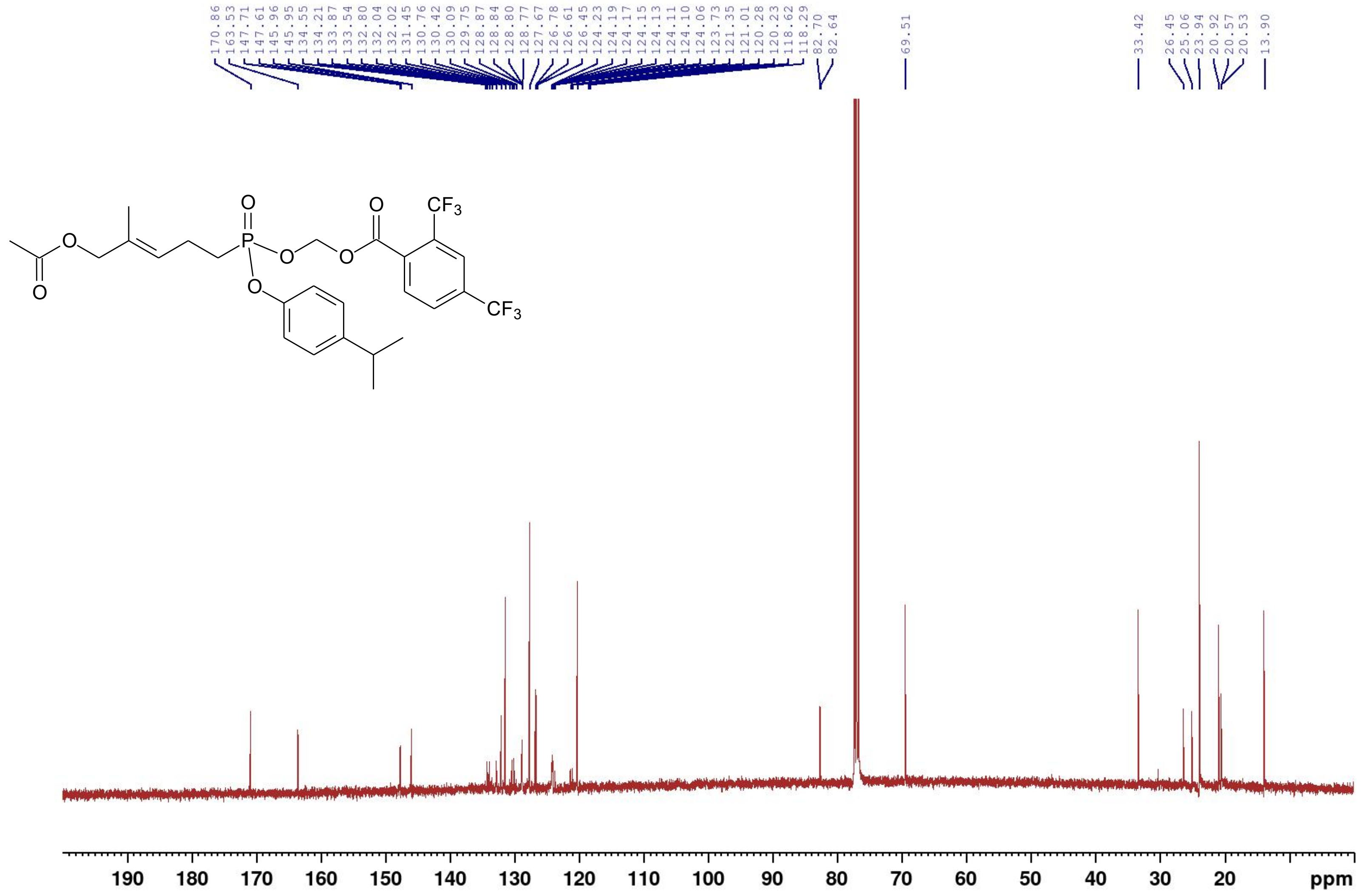

^{13}C NMR Spectrum of Compound **9h** ($CDCl_3$, 101 MHz)

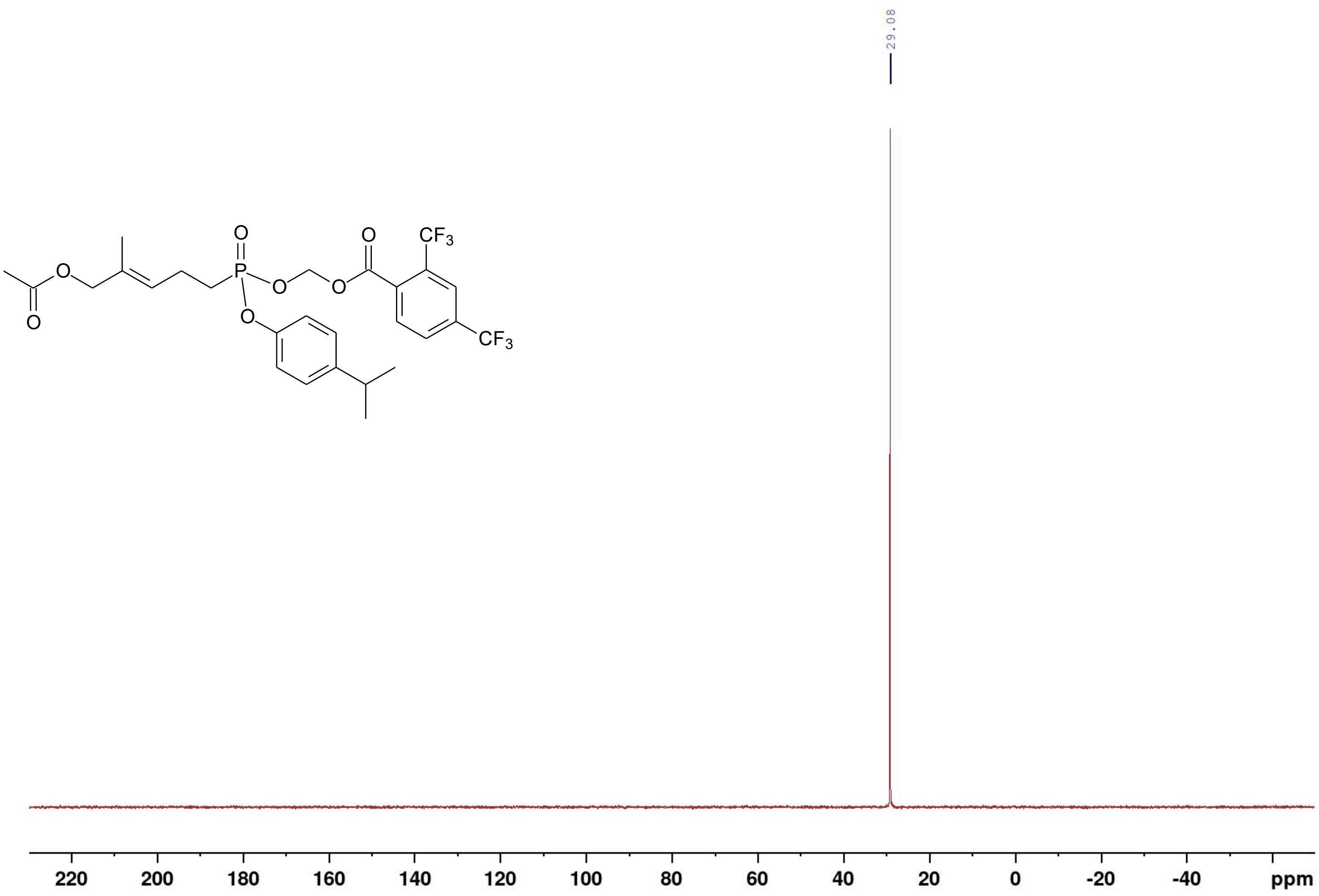

^{31}P NMR Spectrum of Compound **9h** ($CDCl_3$, 162 MHz)

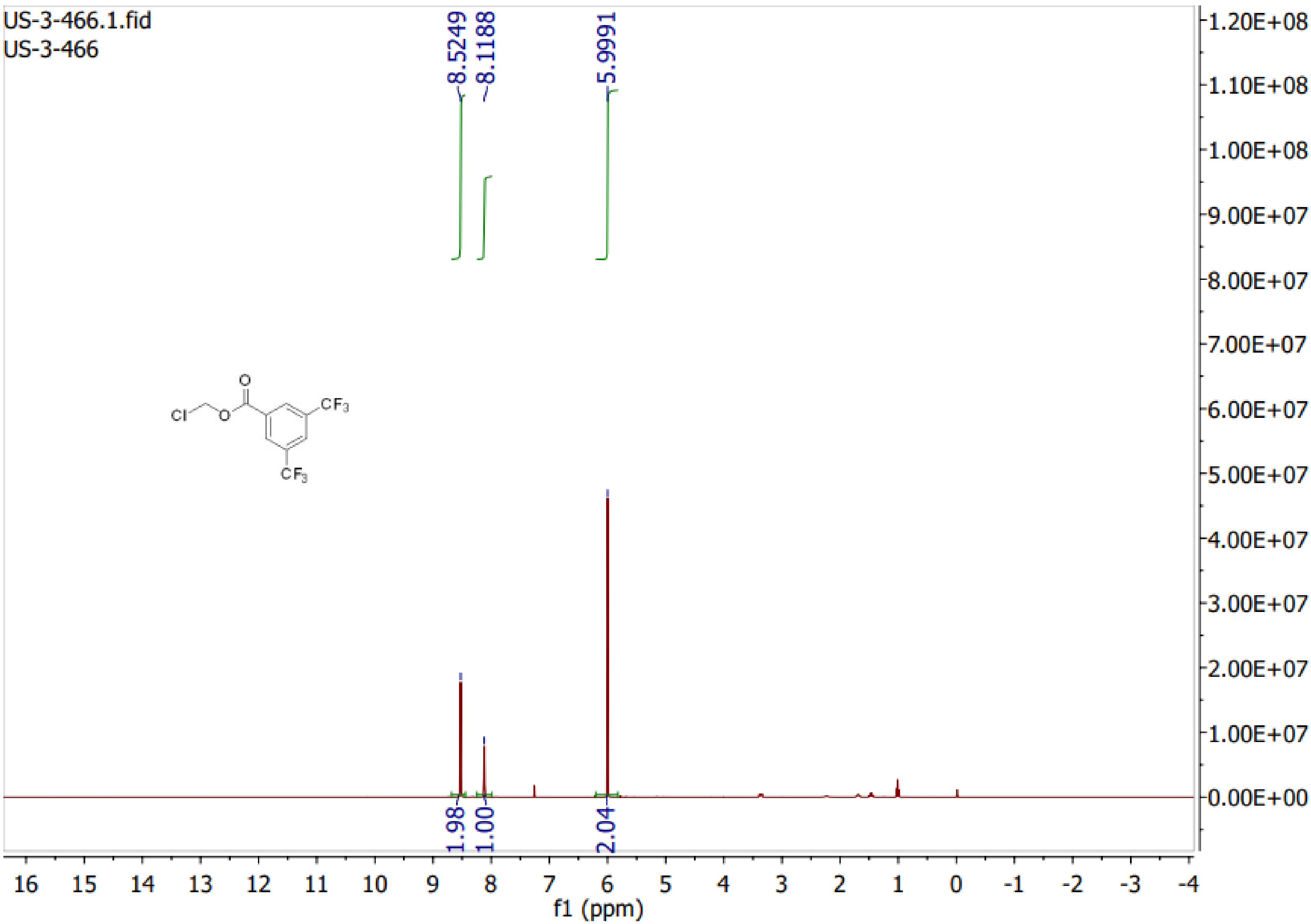

^{1}H NMR Spectrum of Compound **5i** ($CDCl_3$, 400 MHz)

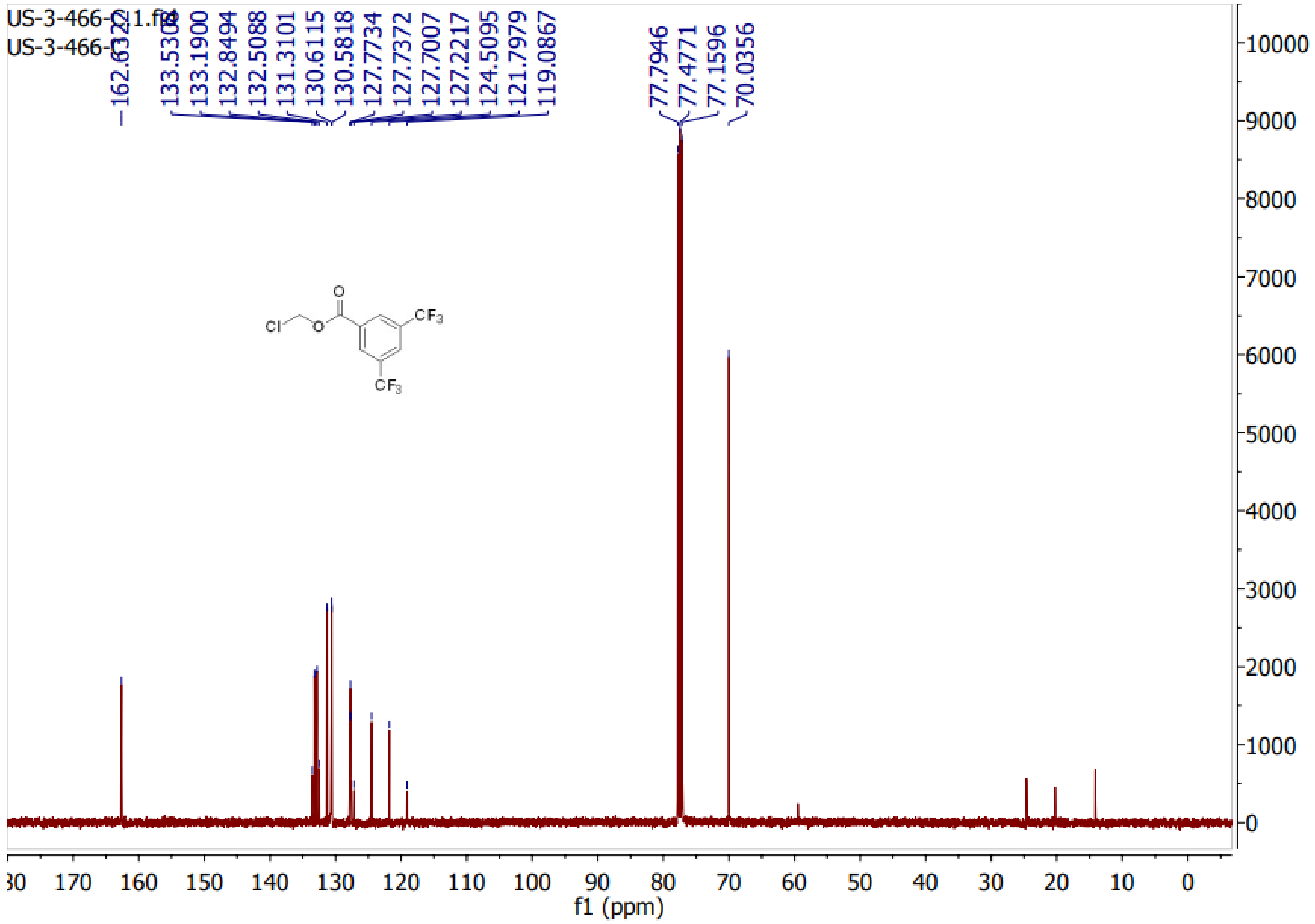

^{13}C NMR Spectrum of Compound **5i** ($CDCl_3$, 101 MHz)

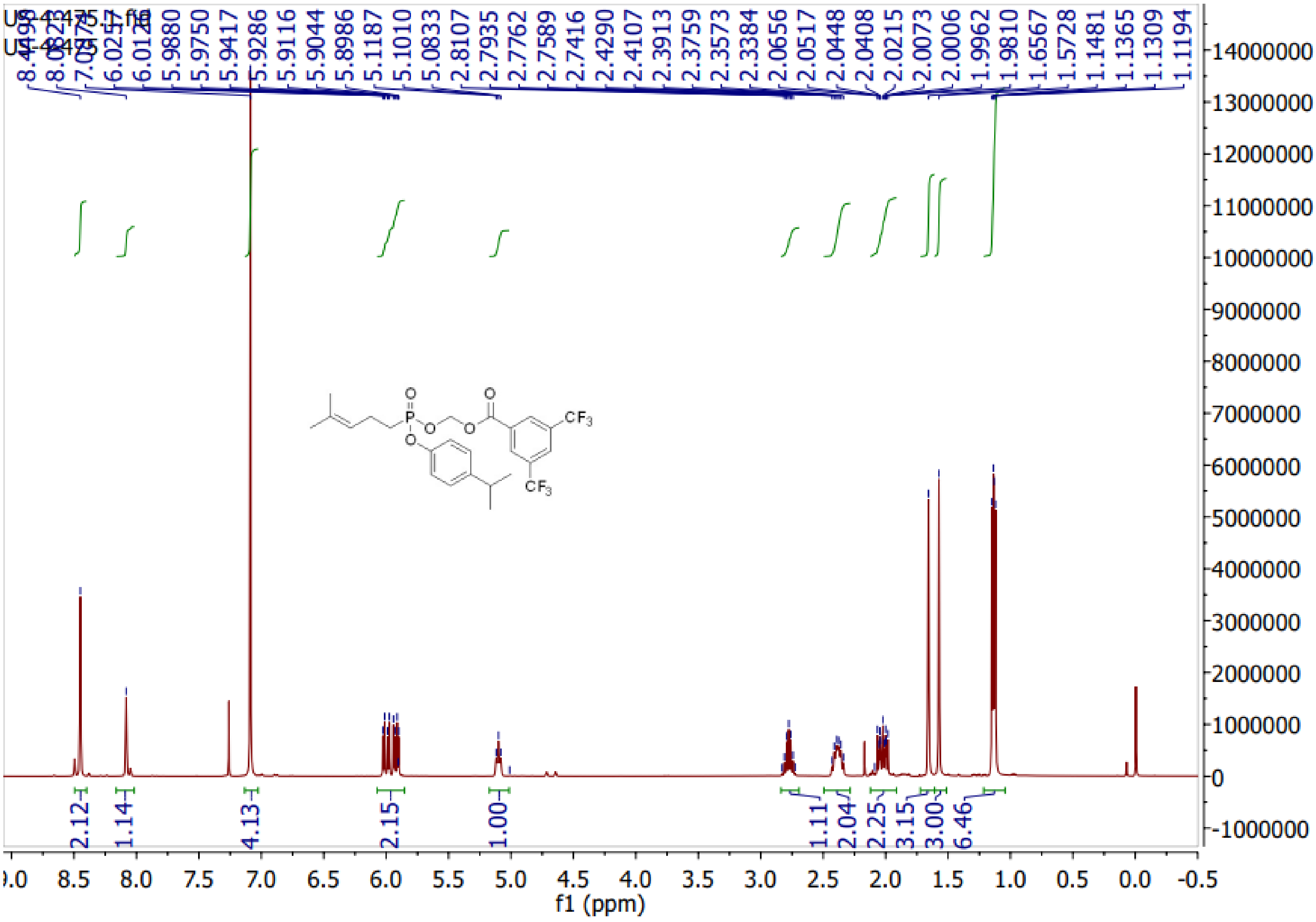

^{1}H NMR Spectrum of Compound **7i** ($CDCl_3$, 400 MHz)

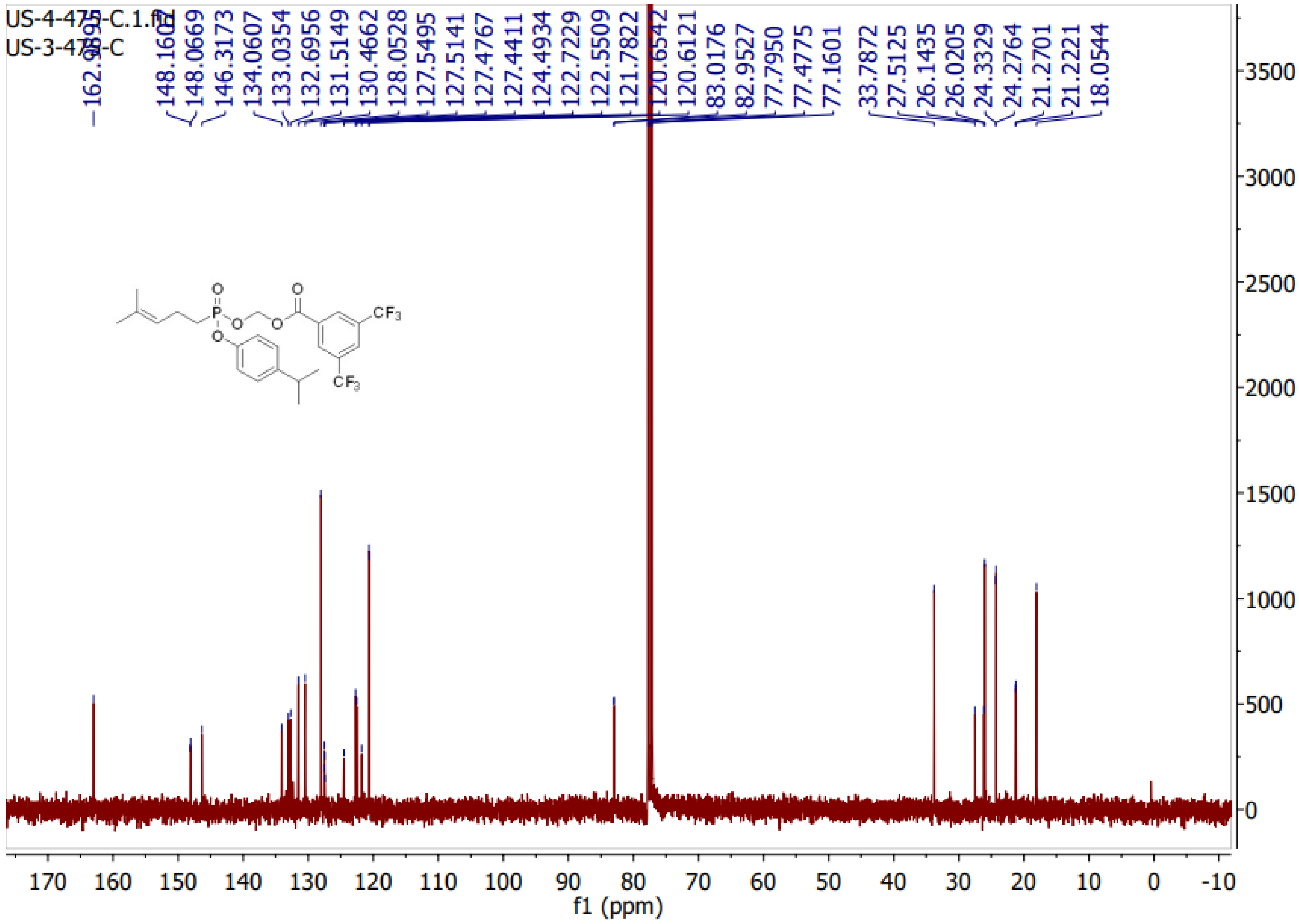

^{13}C NMR Spectrum of Compound **7i** ($CDCl_3$, 101 MHz)

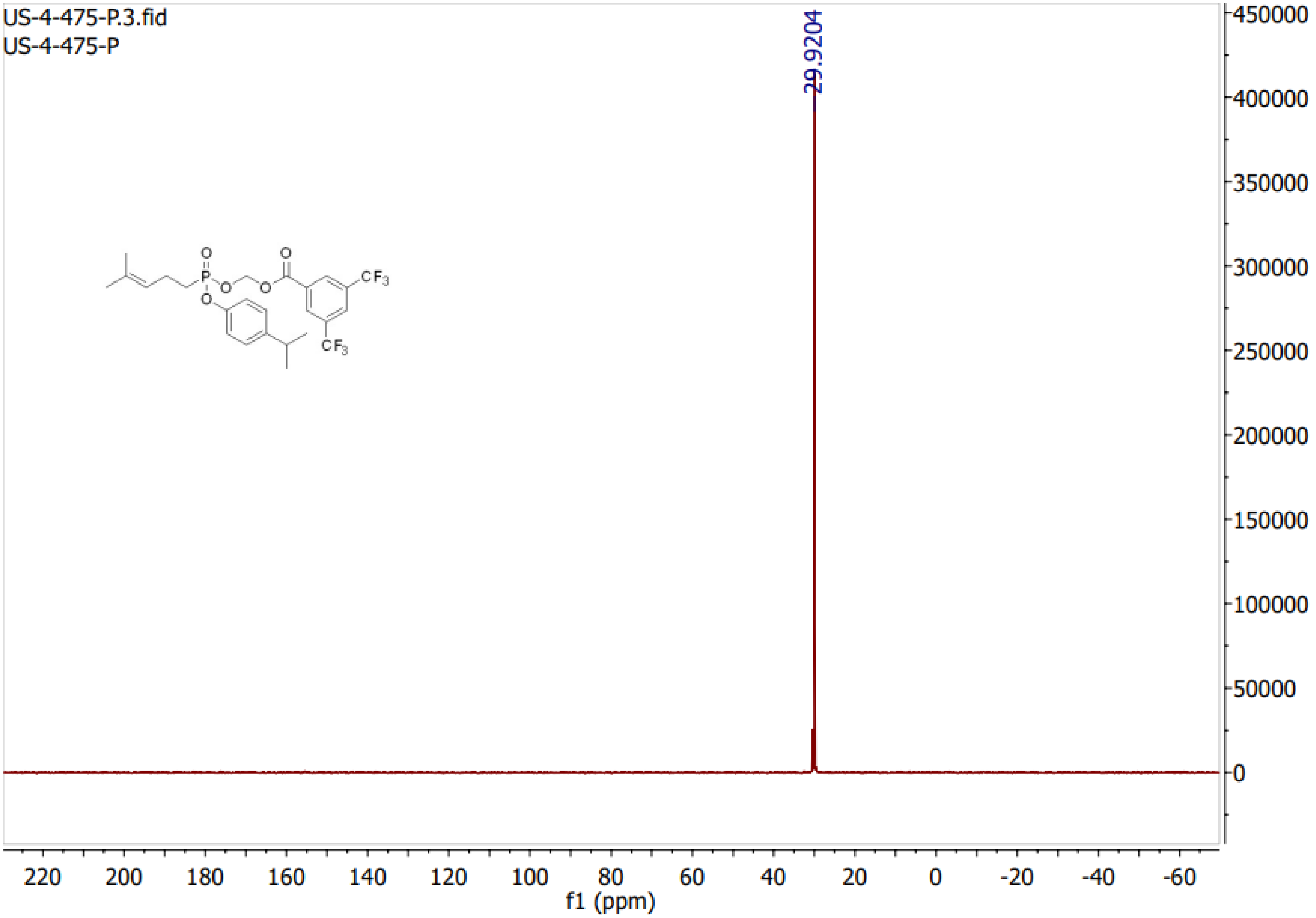

^{31}P NMR Spectrum of Compound **7i** ($CDCl_3$, 162 MHz)

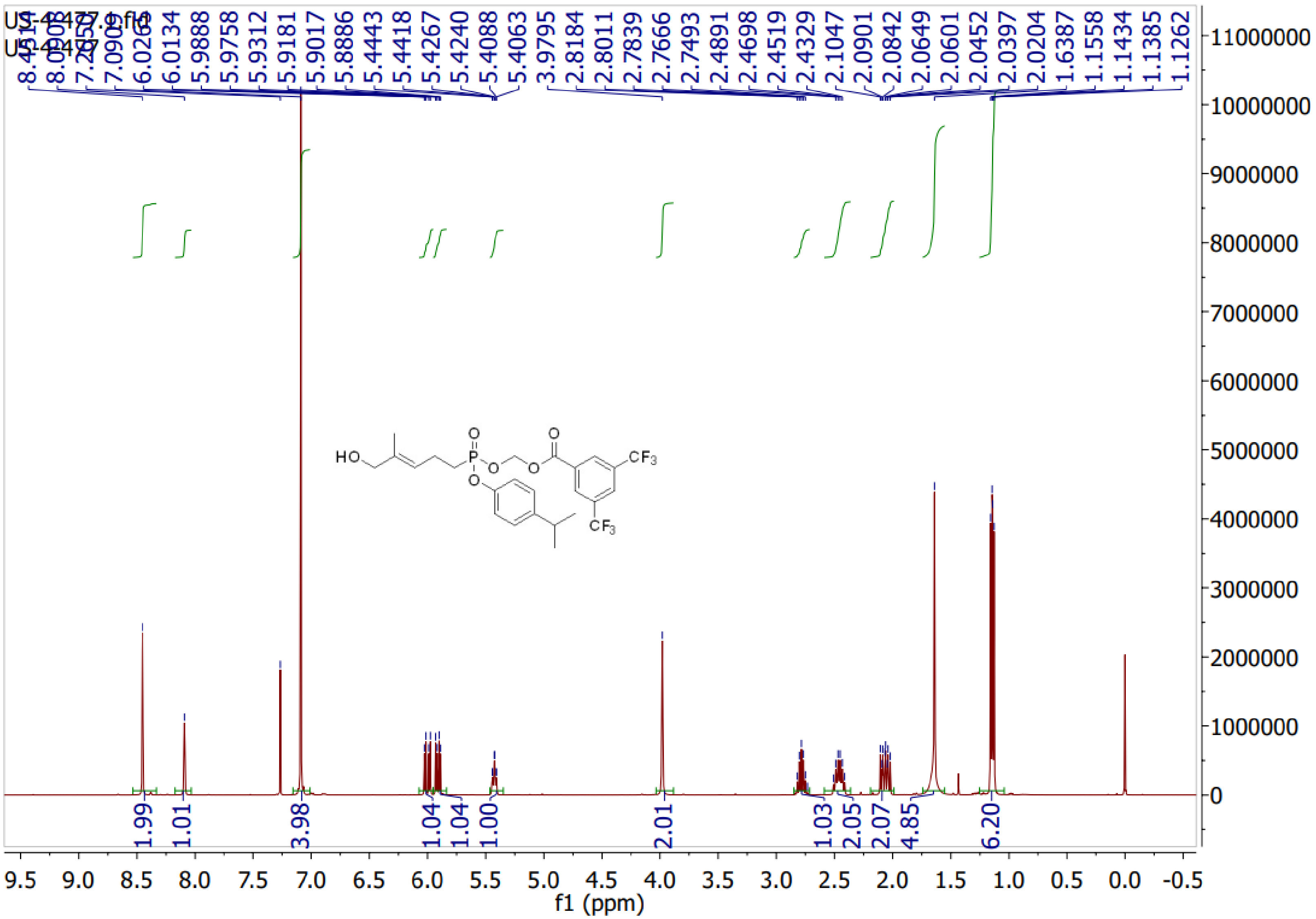

^{1}H NMR Spectrum of Compound **8i** ($CDCl_3$, 400 MHz)

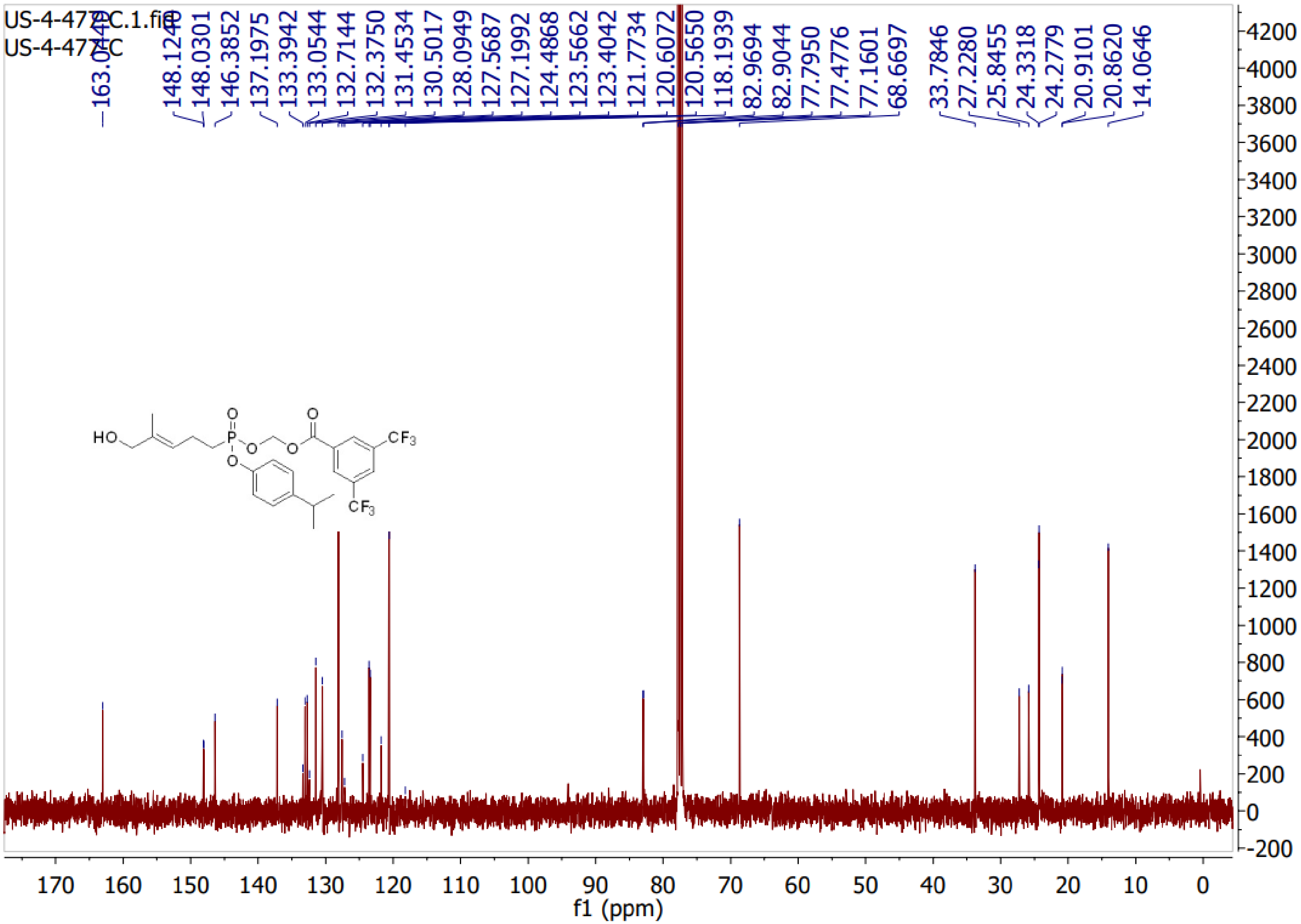

^{13}C NMR Spectrum of Compound **8i** ($CDCl_3$, 101 MHz)

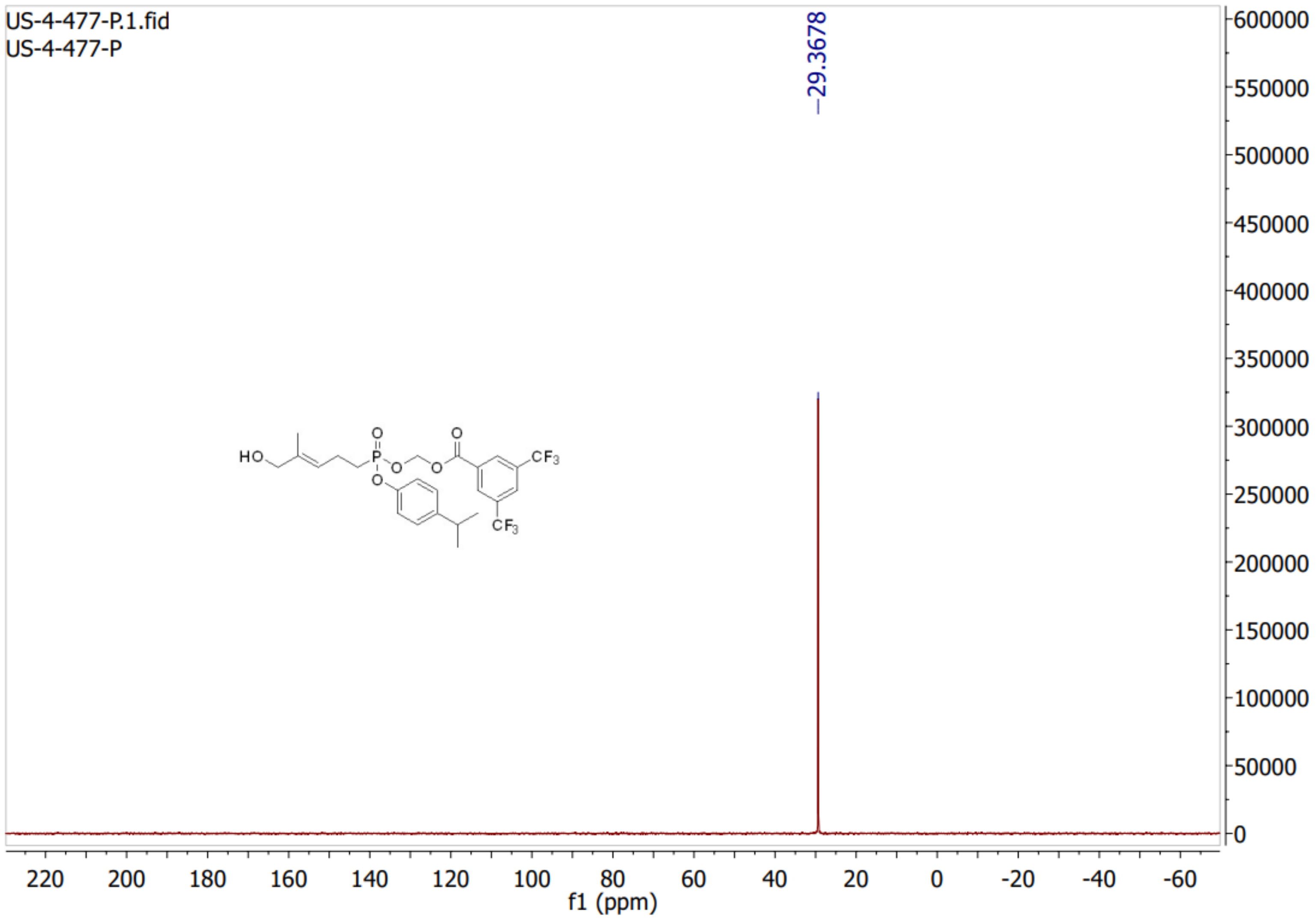

^{31}P NMR Spectrum of Compound **8i** ($CDCl_3$, 162 MHz)

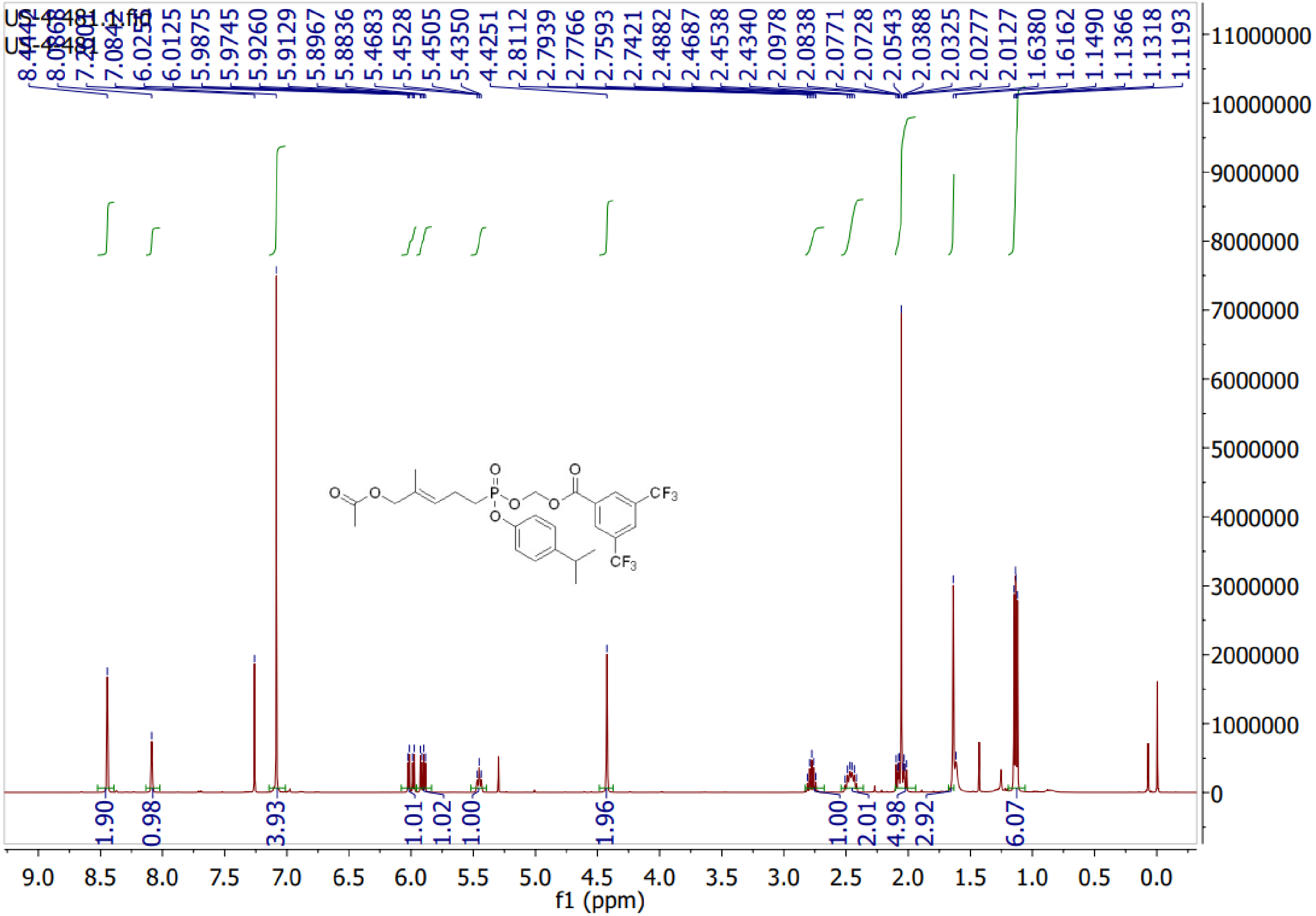

^{1}H NMR Spectrum of Compound **9i** ($CDCl_3$, 400 MHz)

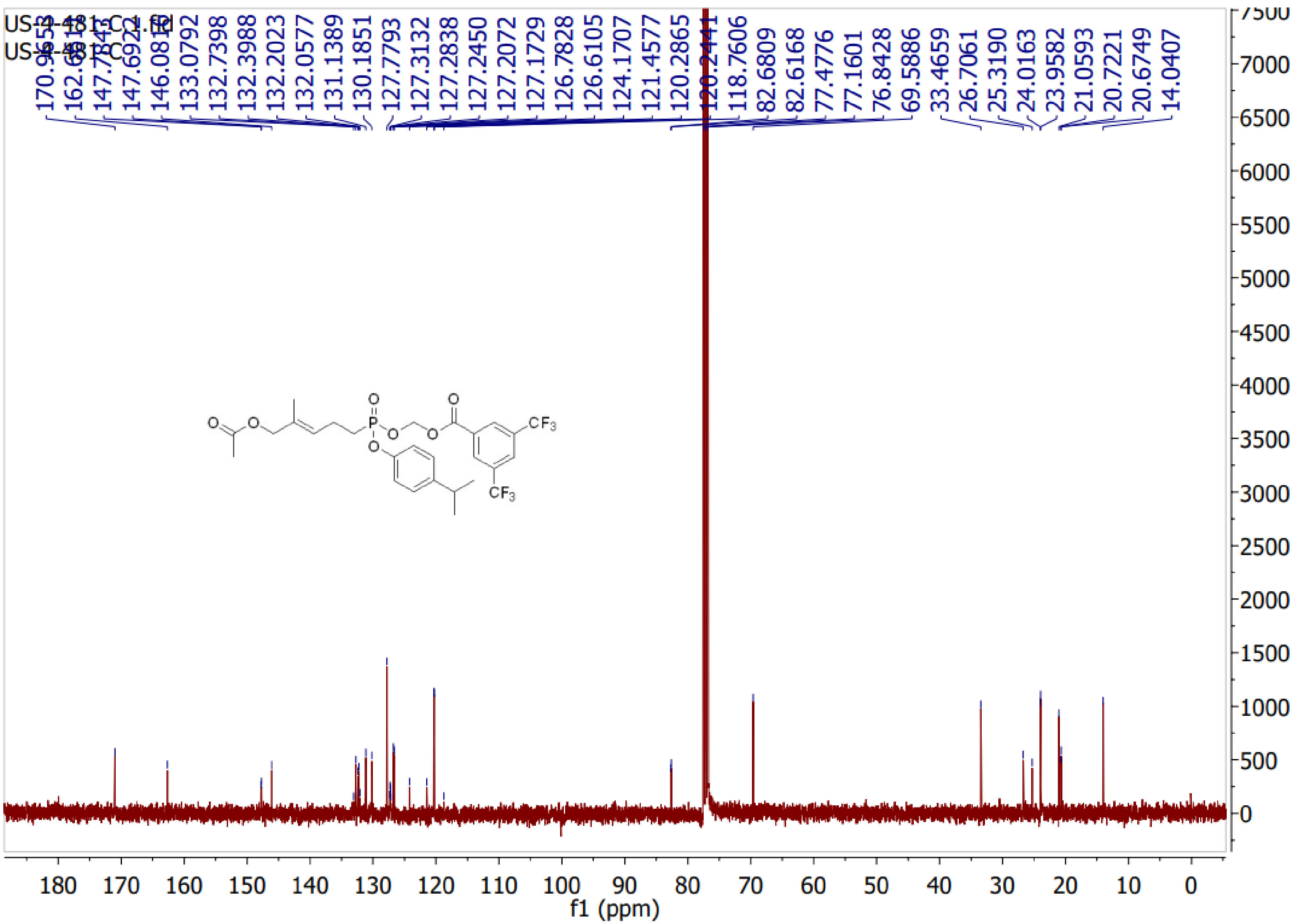

^{13}C NMR Spectrum of Compound **9i** ($CDCl_3$, 101 MHz)

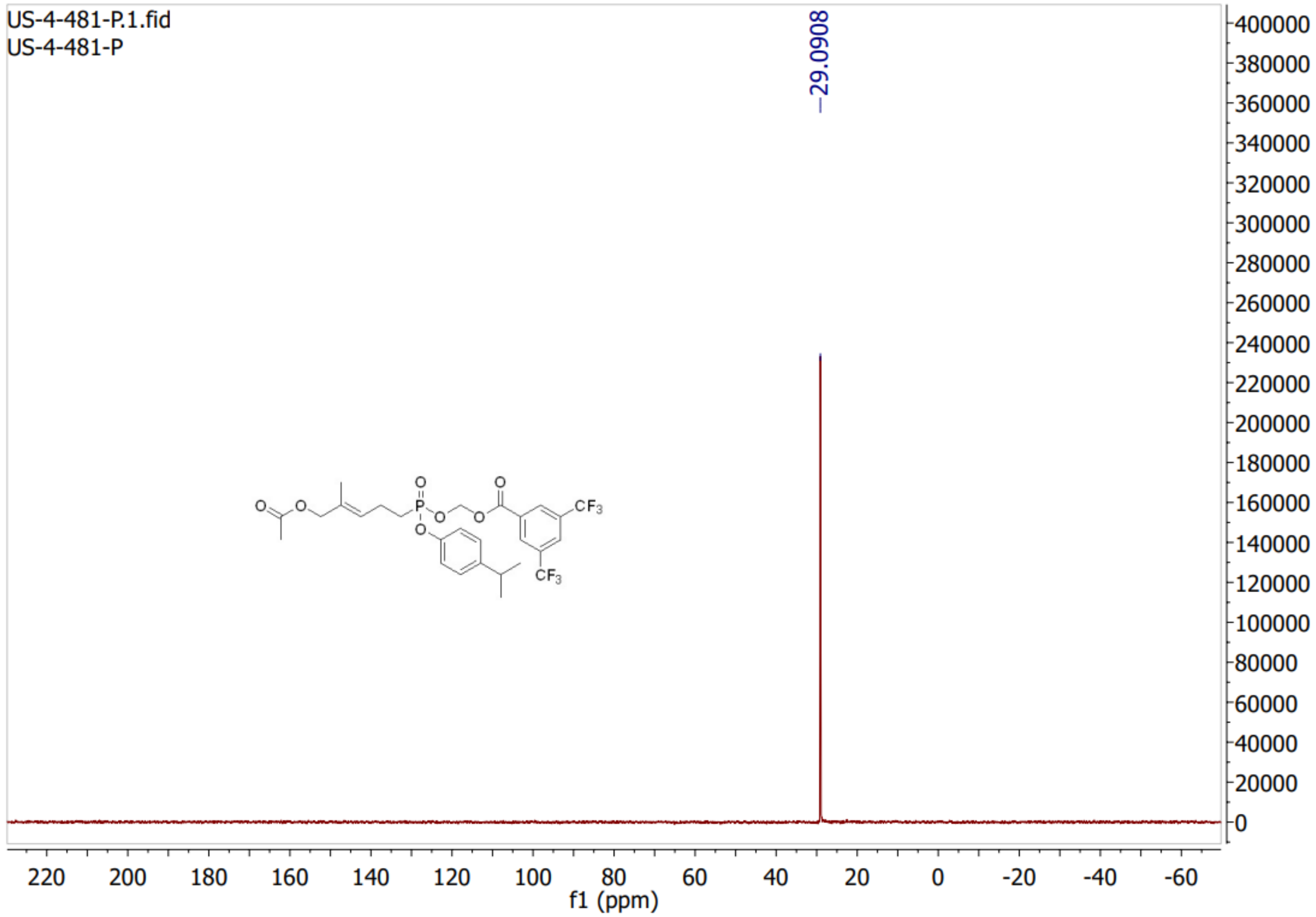

^{31}P NMR Spectrum of Compound 9**i** ($CDCl_3$, 162 MHz)

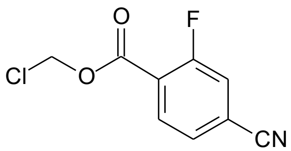

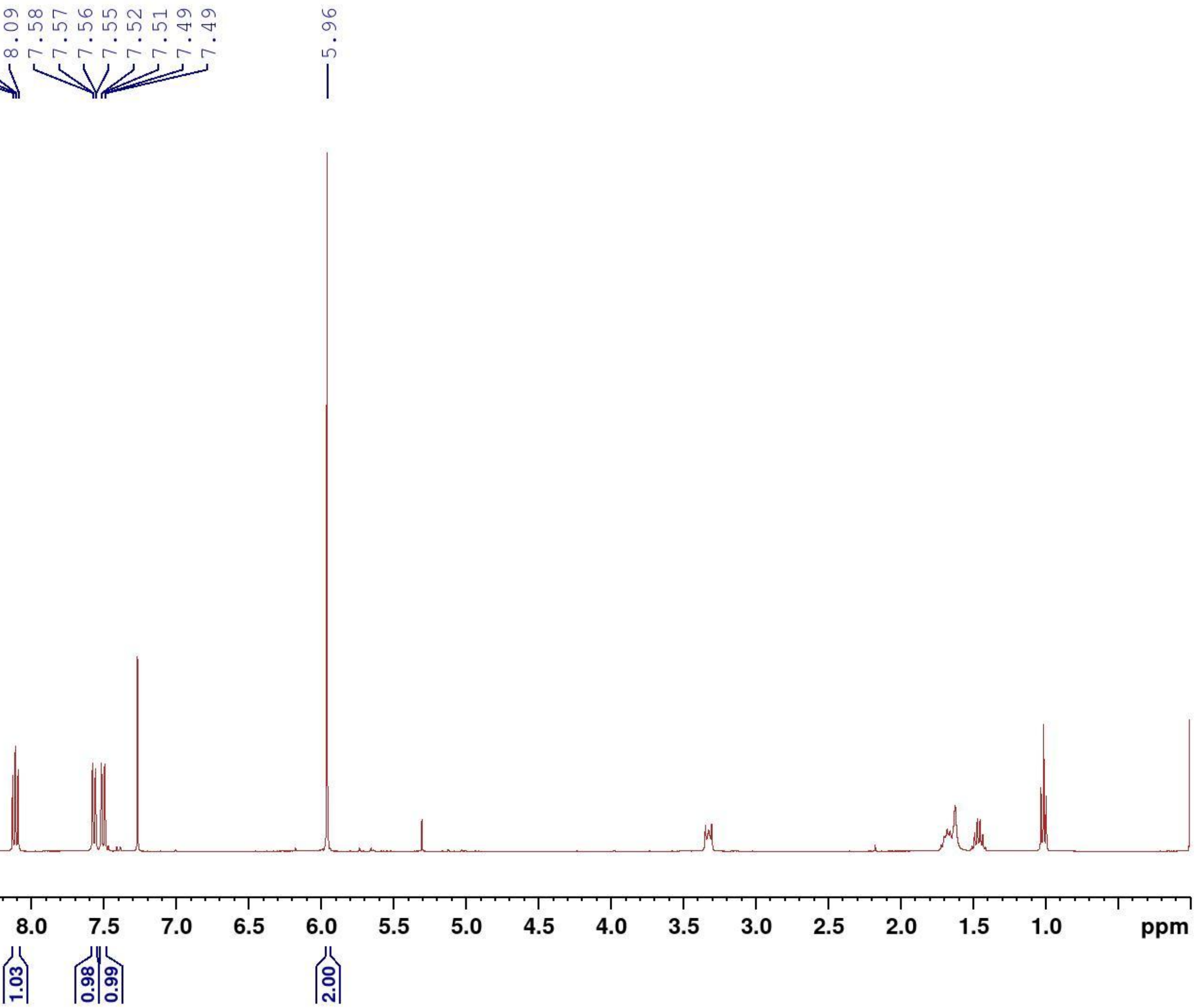

^{1}H NMR Spectrum of Compound **5j** ($CDCl_3$, 400 MHz)

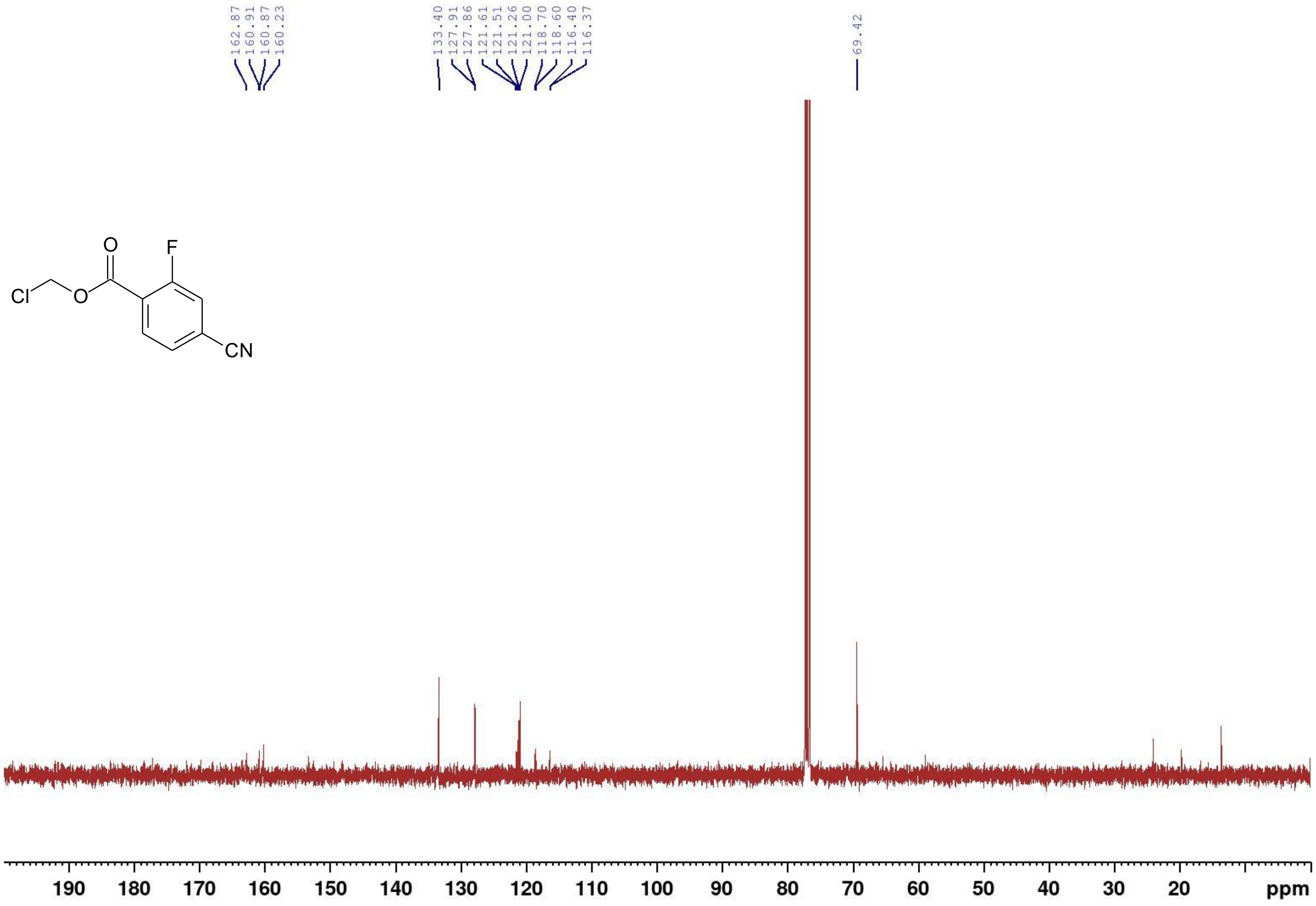

^{31}C NMR Spectrum of Compound **5j** ($CDCl_3$, 101 MHz)

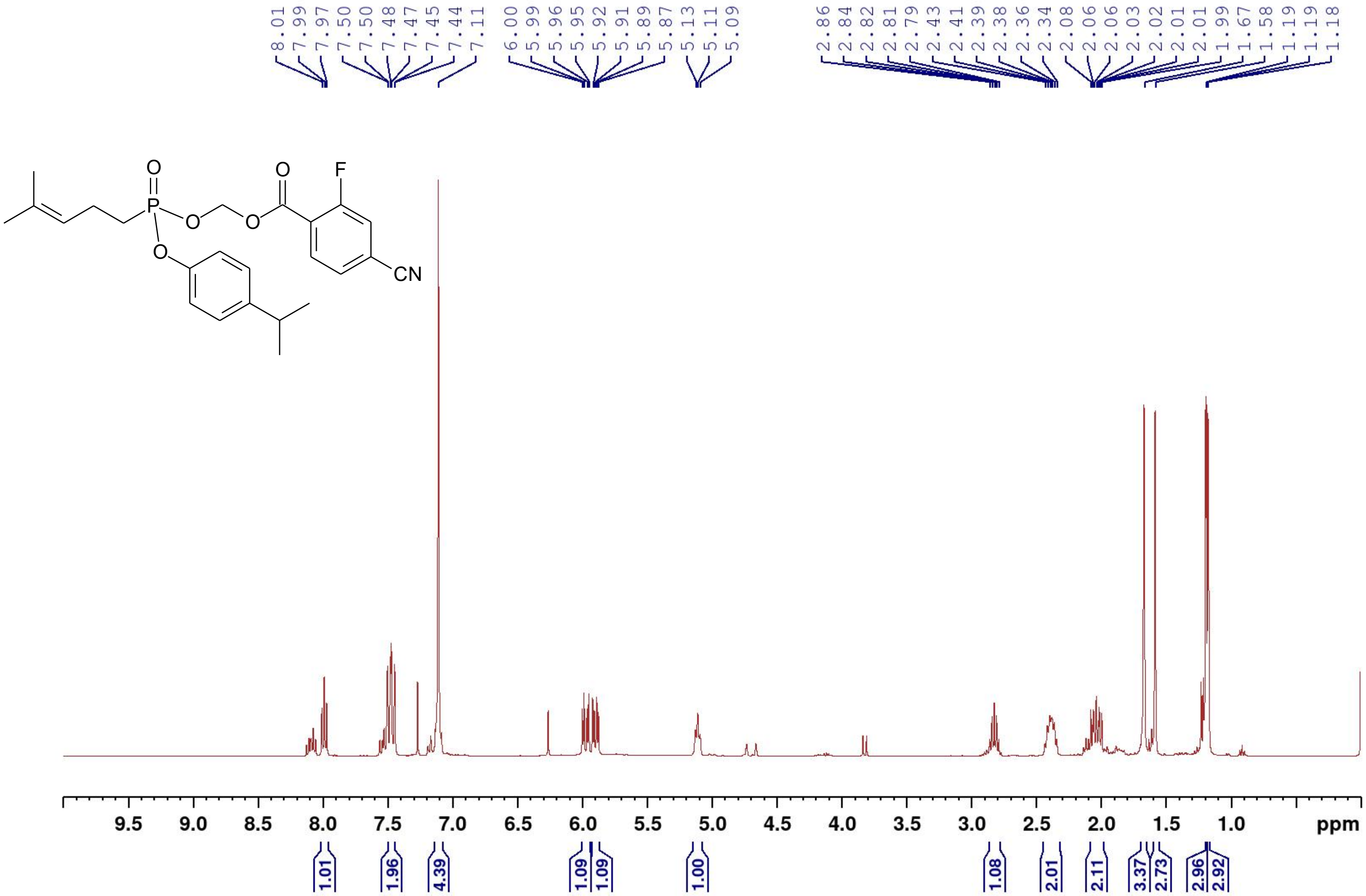

^{1}H NMR Spectrum of Compound **7j** ($CDCl_3$, 400 MHz)

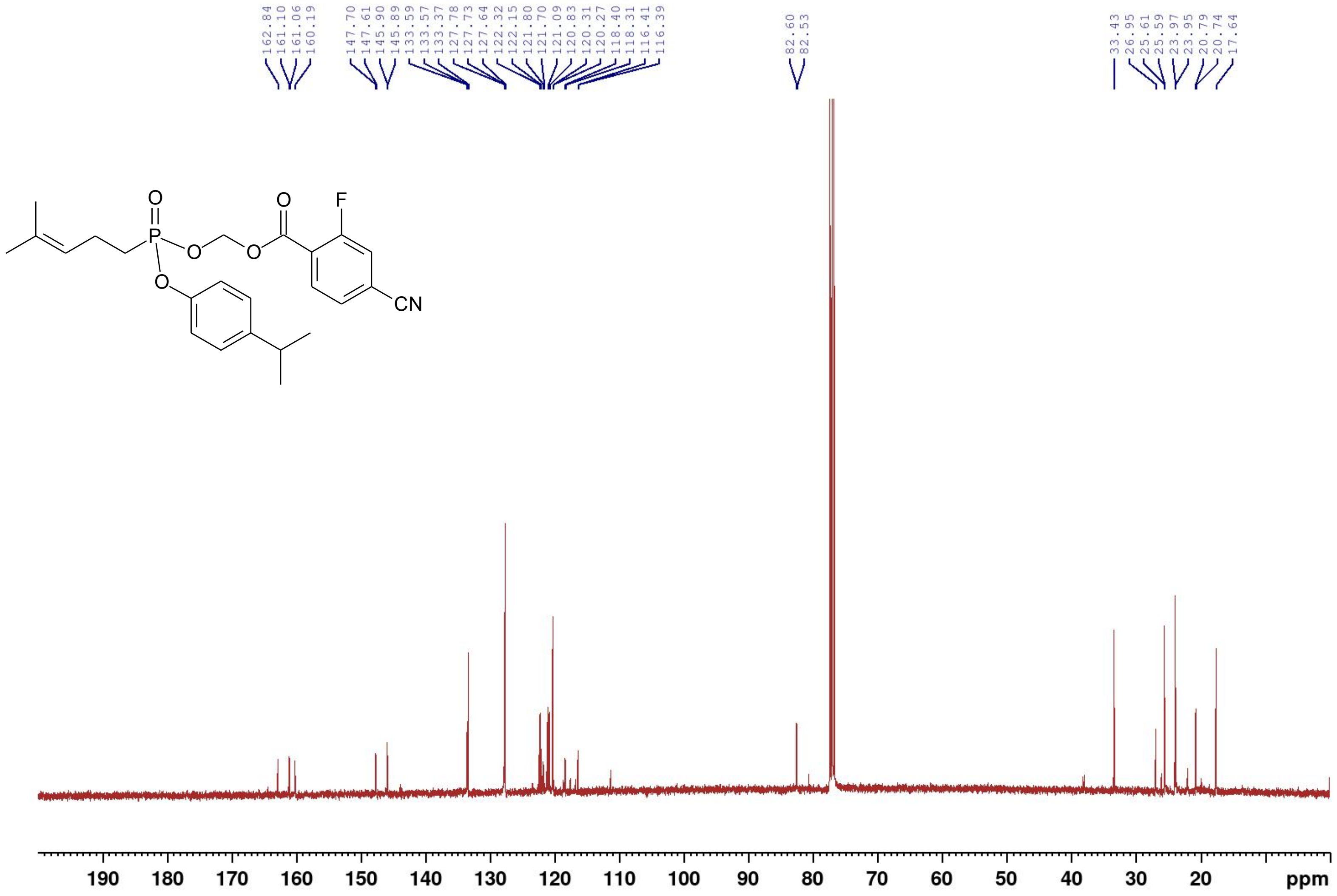

^{13}C NMR Spectrum of Compound **7j** ($CDCl_3$, 101 MHz)

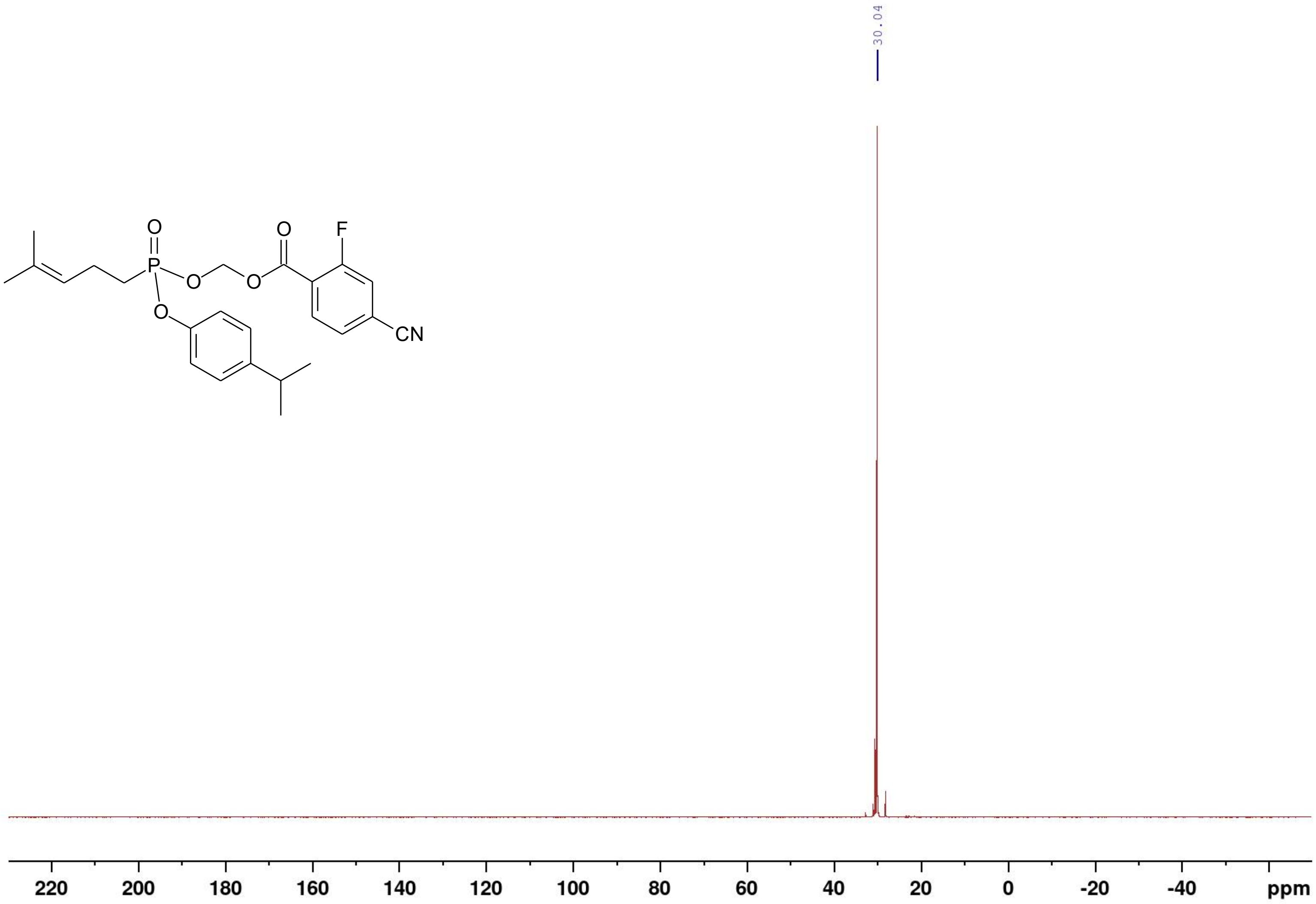

^{31}P NMR Spectrum of Compound **7j** ($CDCl_3$, 162 MHz)

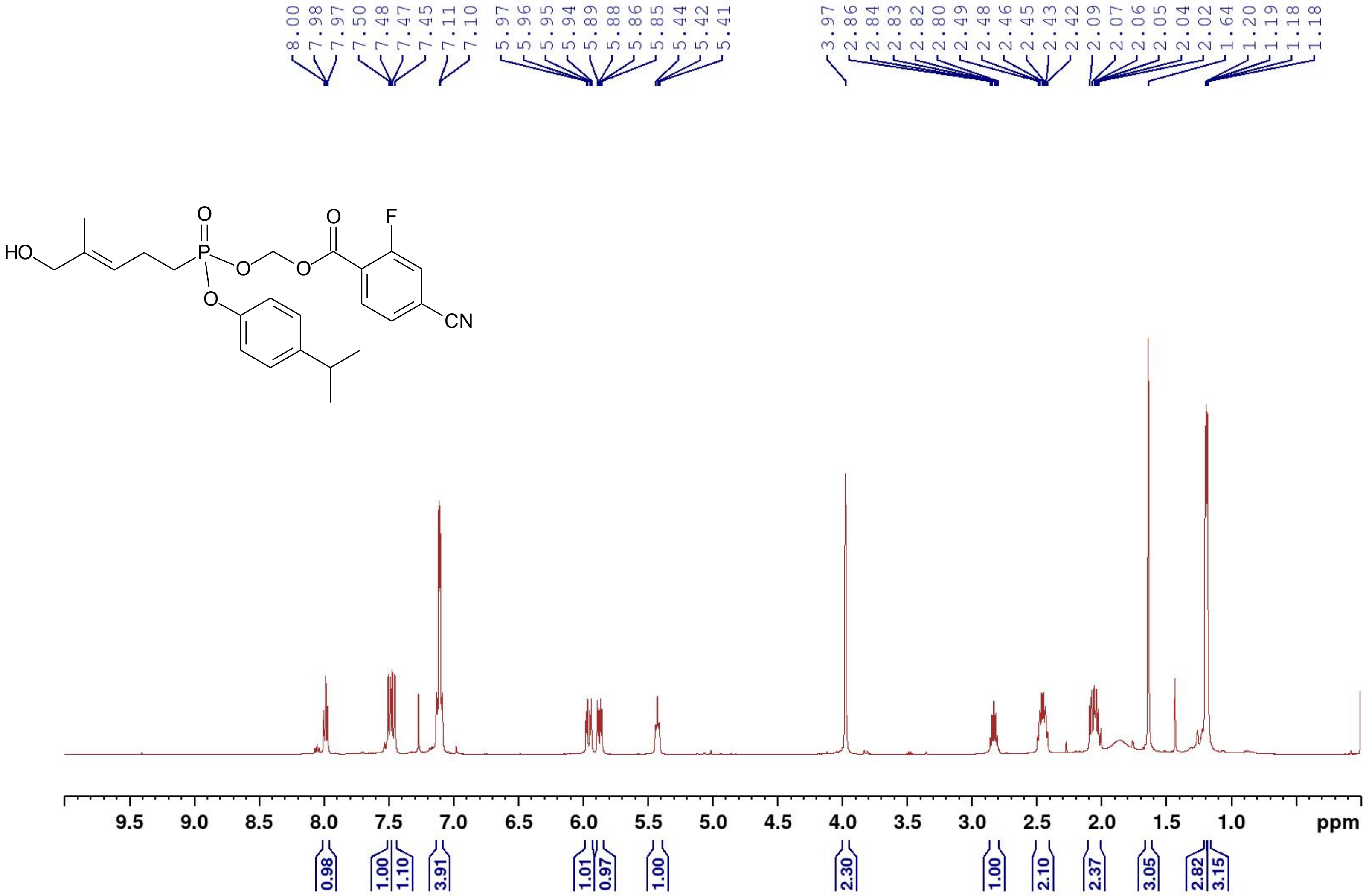

^{1}H NMR Spectrum of Compound **8j** ($CDCl_3$, 500 MHz)

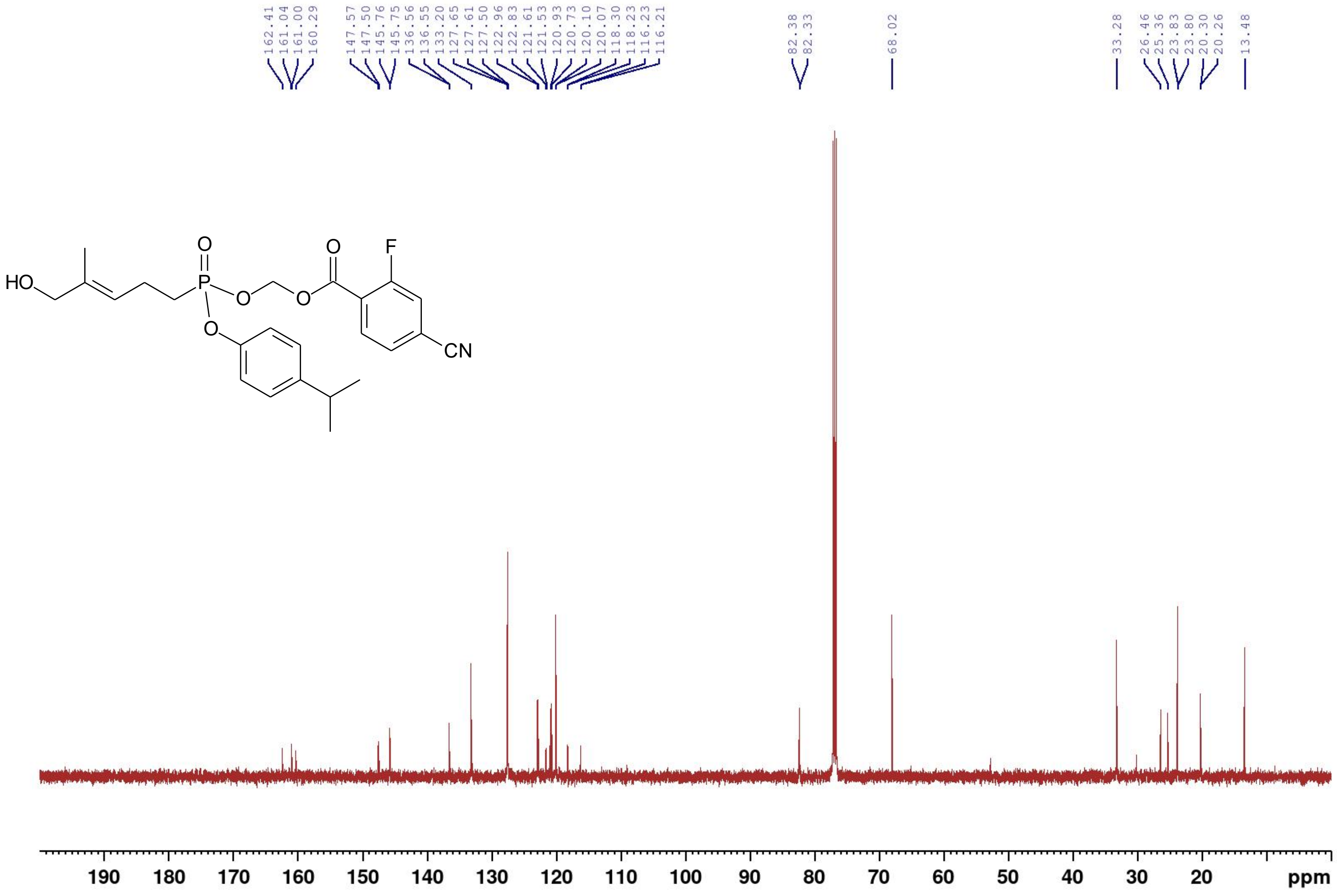

^{13}C NMR Spectrum of Compound **8j** ($CDCl_3$, 126 MHz)

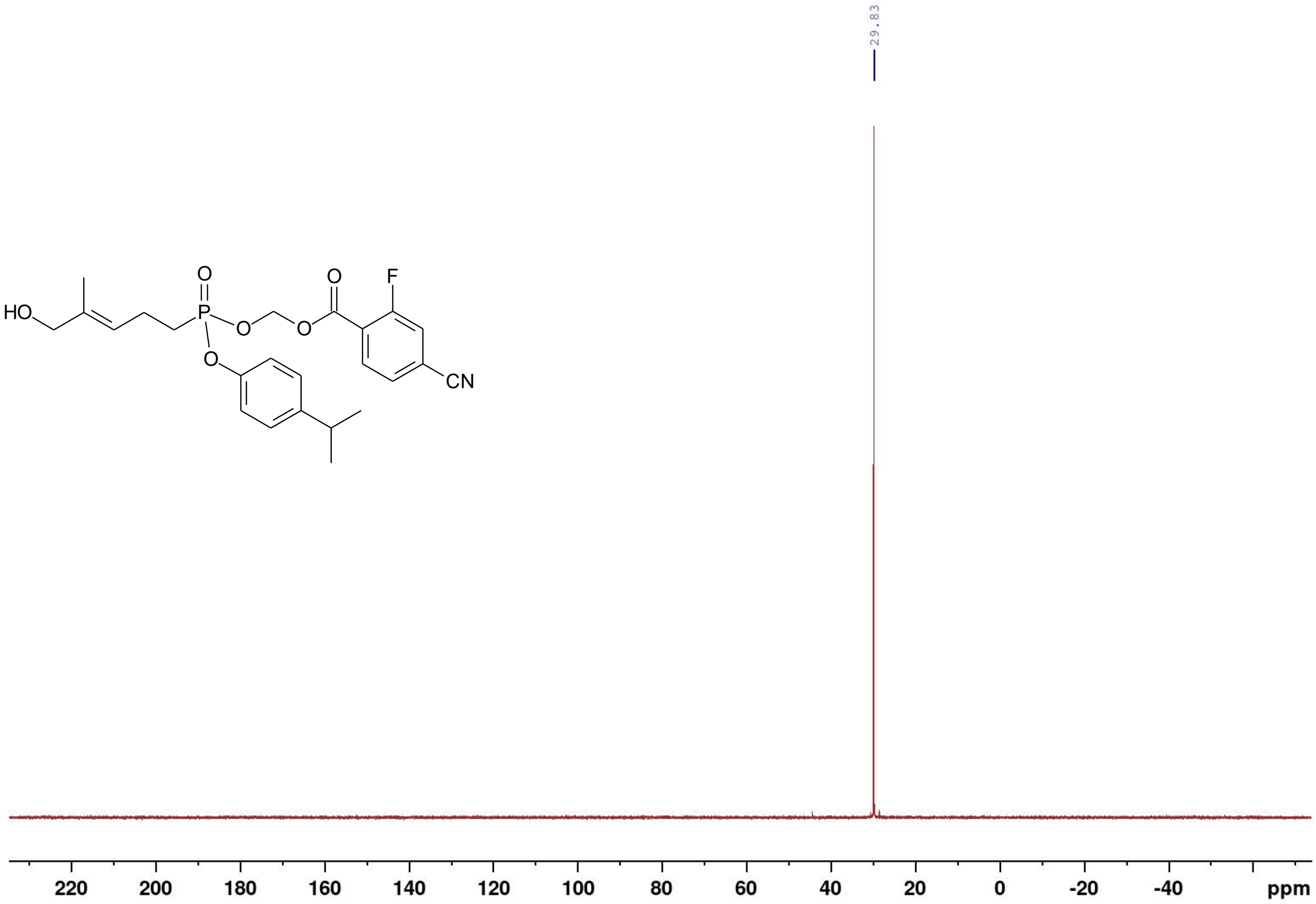

^{31}P NMR Spectrum of Compound **8j** ($CDCl_3$, 203 MHz)

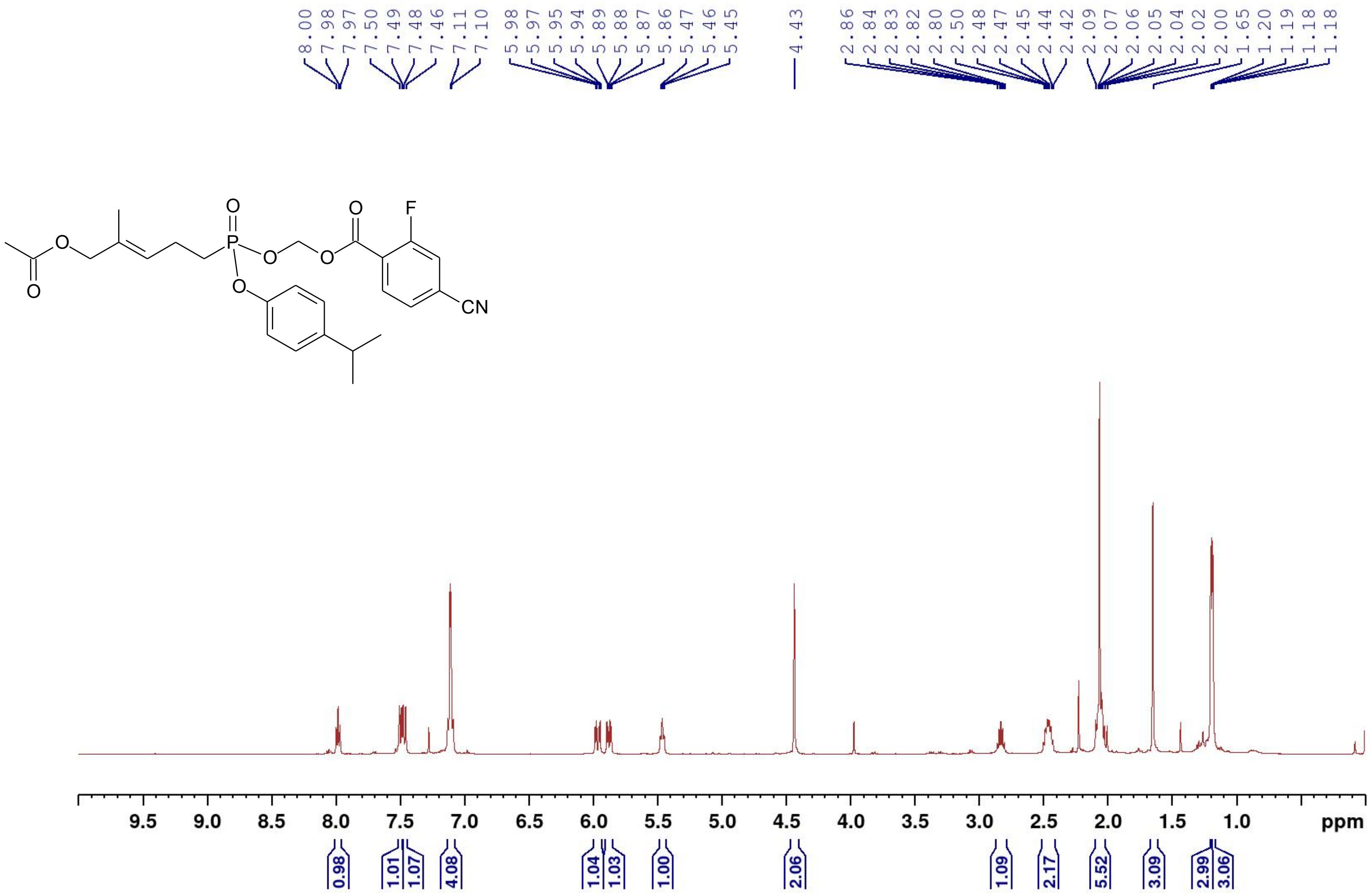

^{1}H NMR Spectrum of Compound **9j** ($CDCl_3$, 500 MHz)

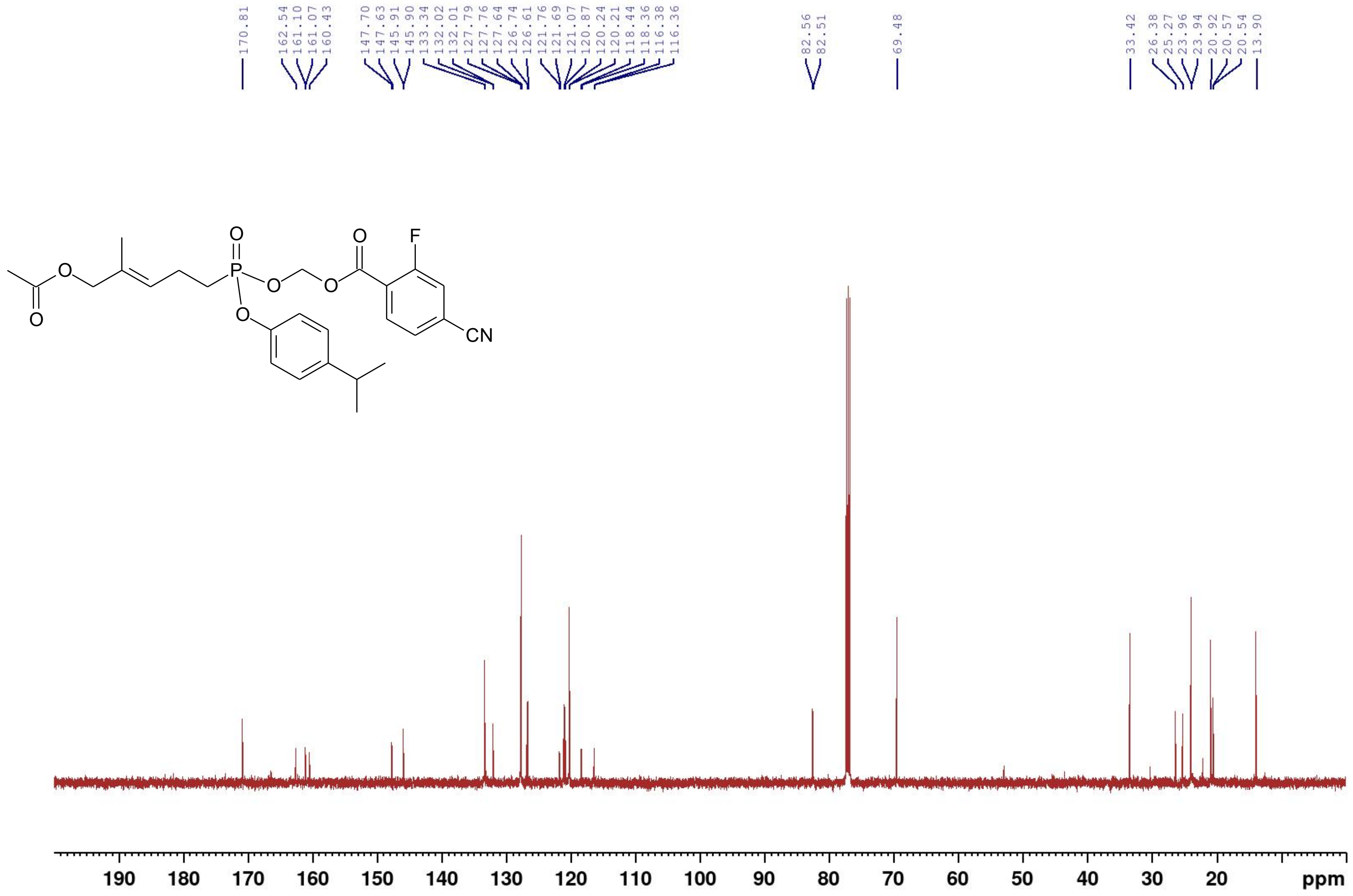

^{13}C NMR Spectrum of Compound **9j** ($CDCl_3$, 126 MHz)

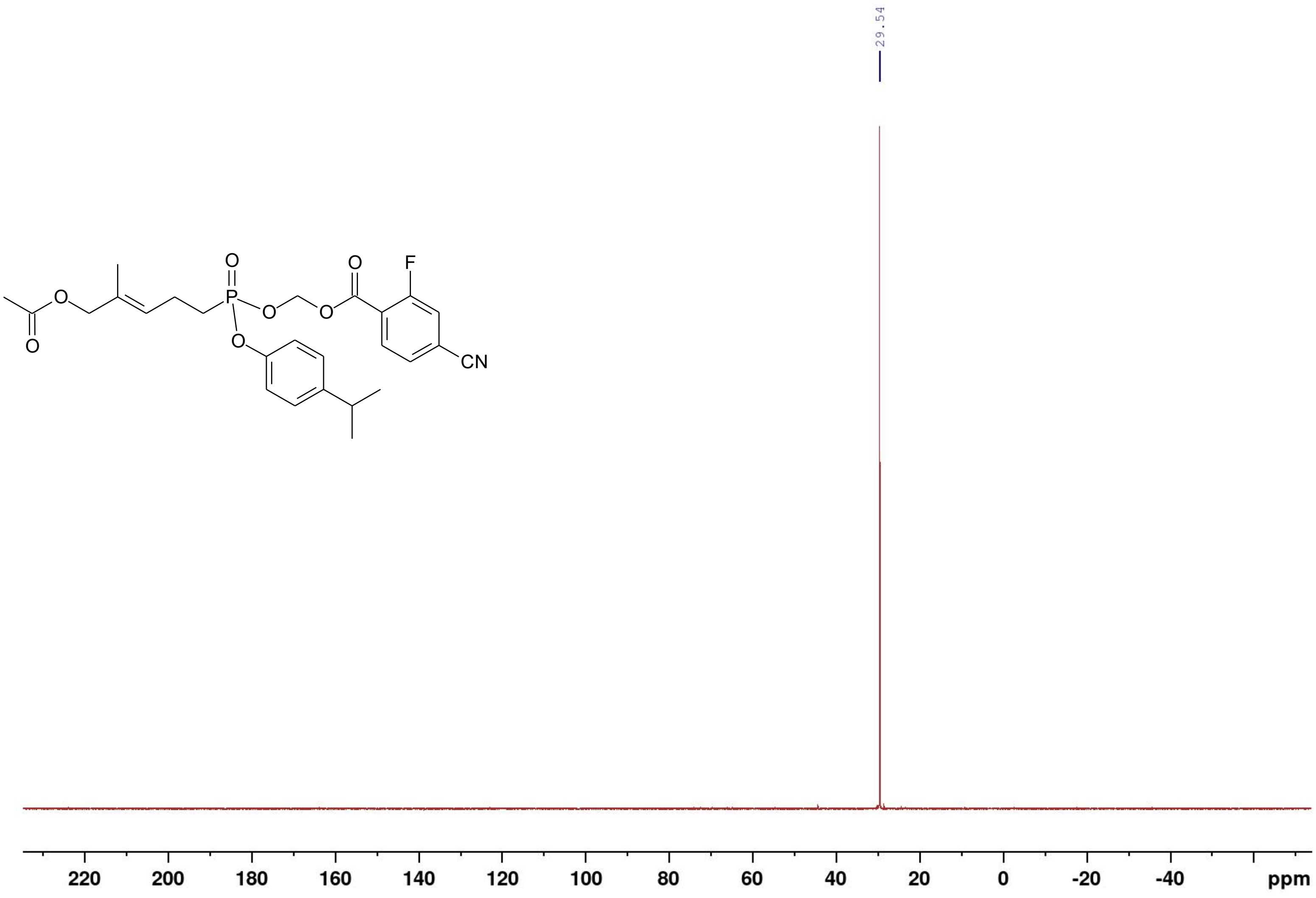

^{31}P NMR Spectrum of Compound **9j** ($CDCl_3$, 203 MHz)

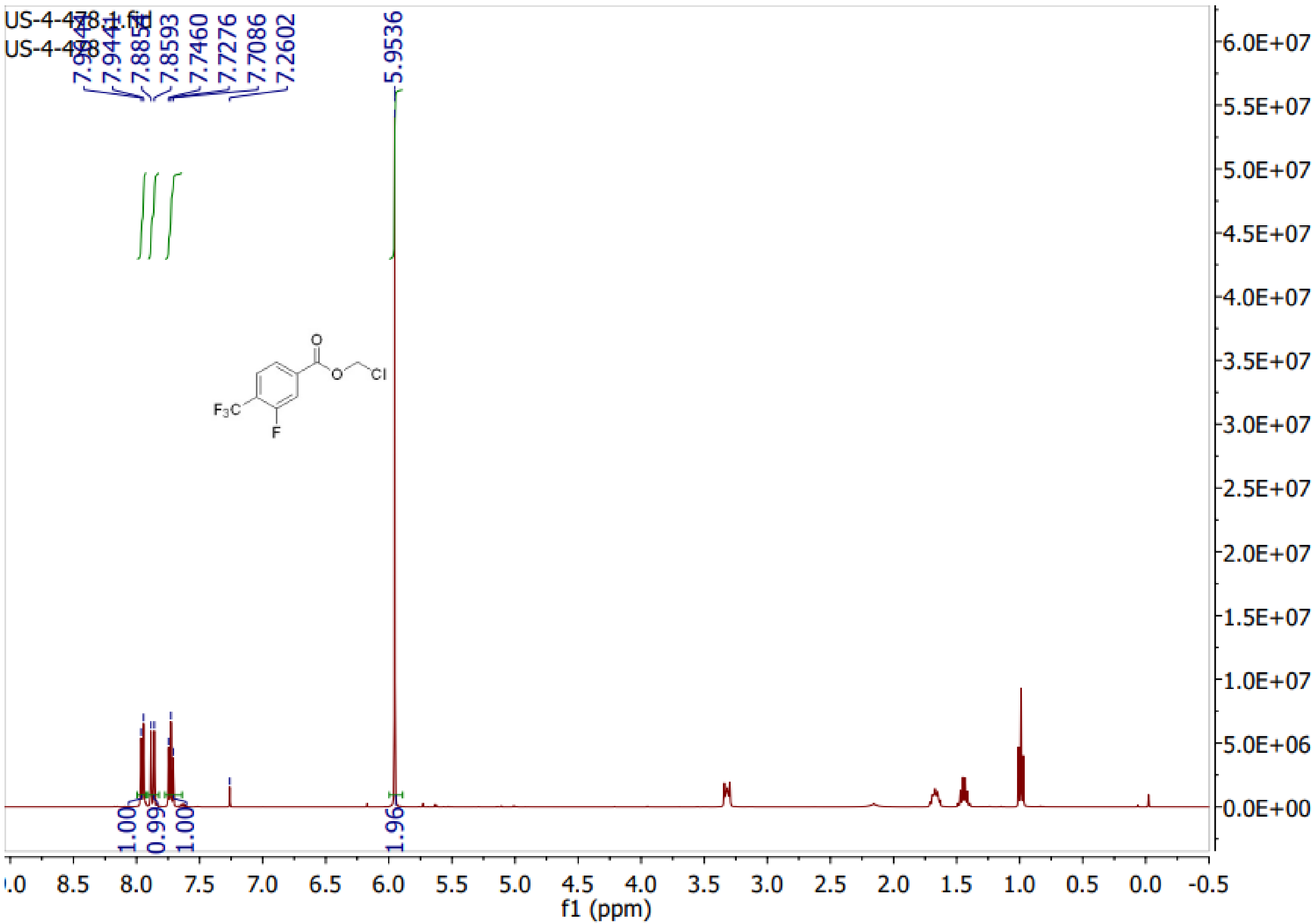

^{1}H NMR Spectrum of Compound **5k** ($CDCl_3$, 400 MHz)

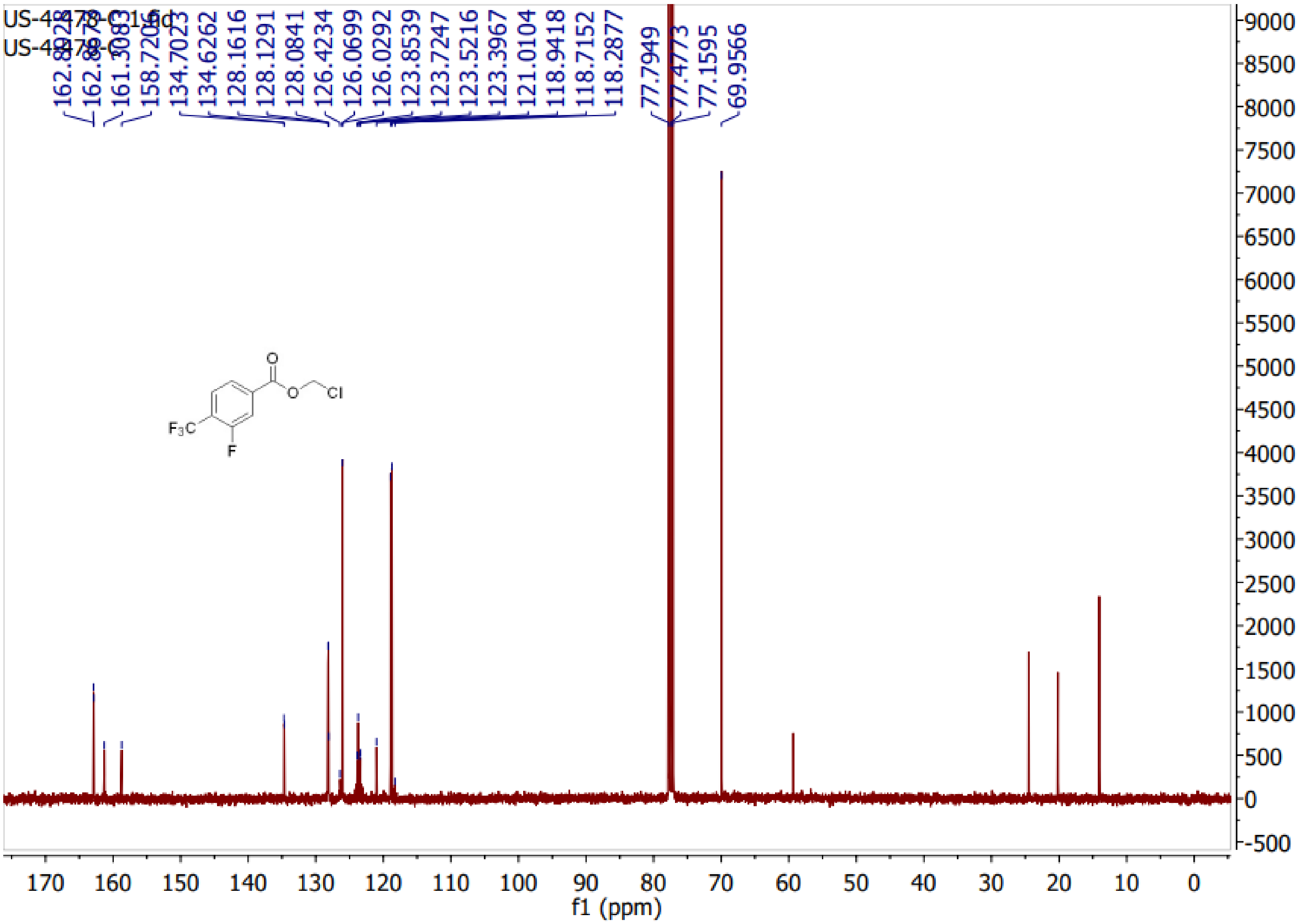

^{13}C NMR Spectrum of Compound **5k** ($CDCl_3$, 126 MHz)

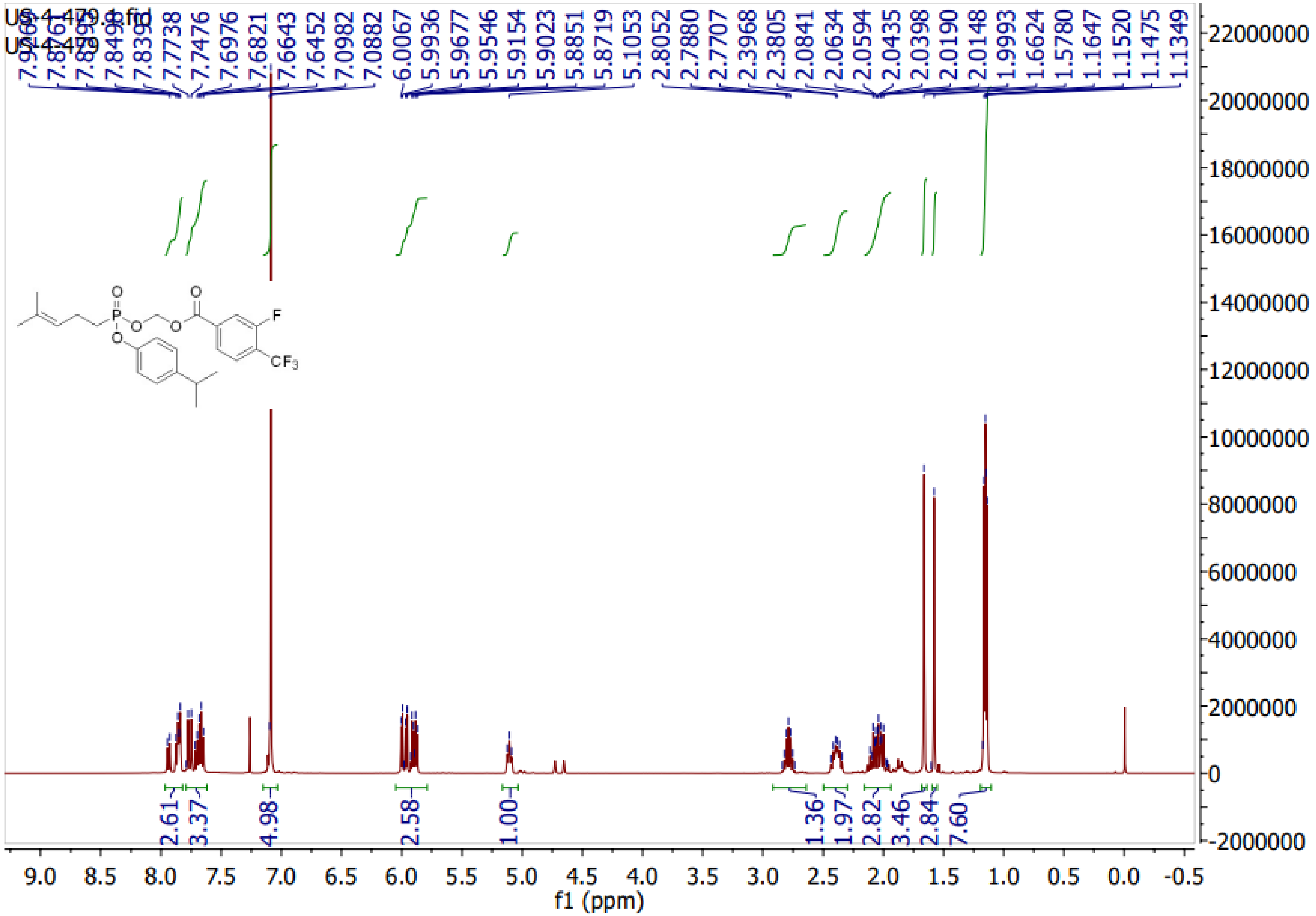

^{1}H NMR Spectrum of Compound **7k** ($CDCl_3$, 400 MHz)

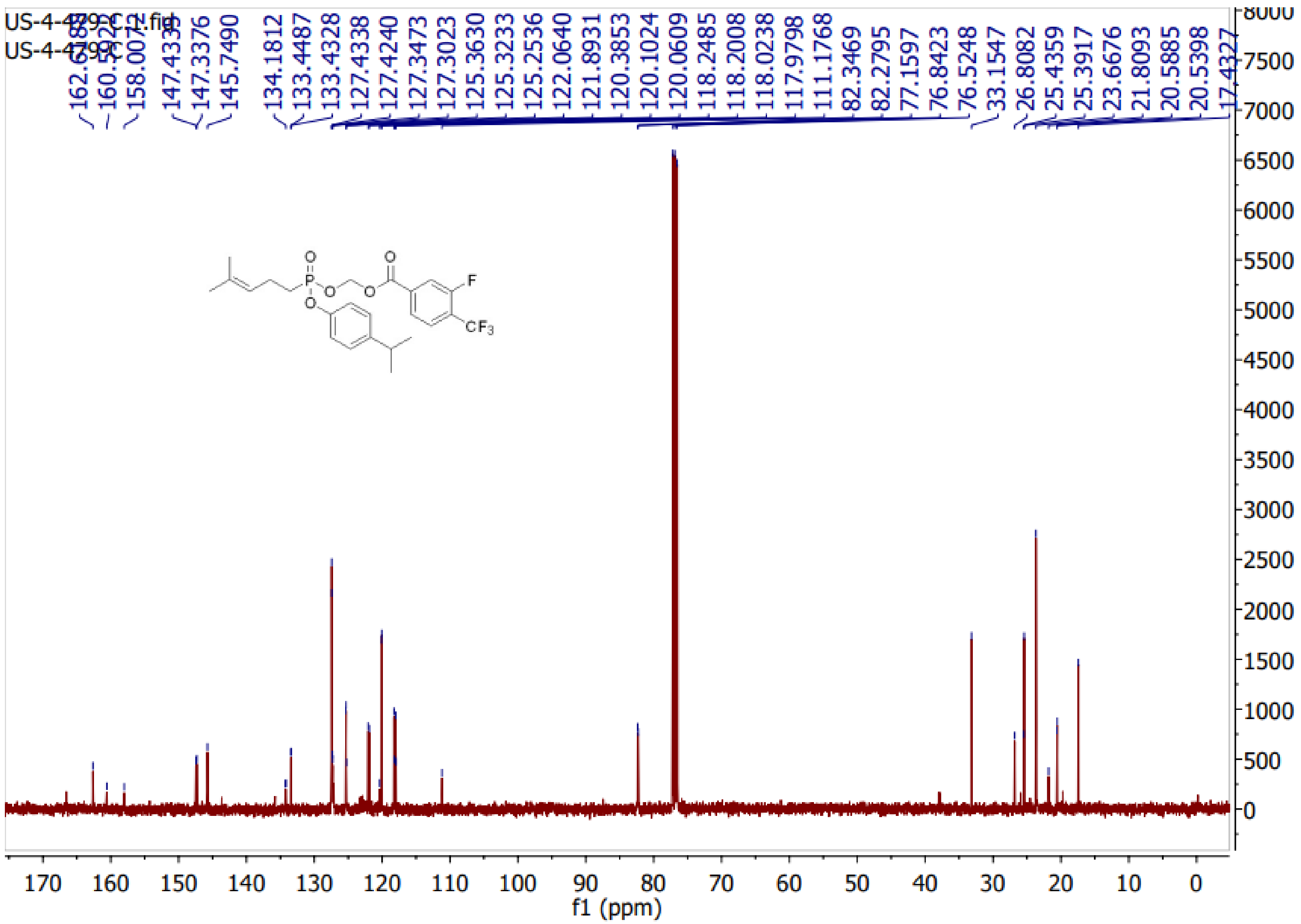

^{13}C NMR Spectrum of Compound **7k** ($CDCl_3$, 101 MHz)

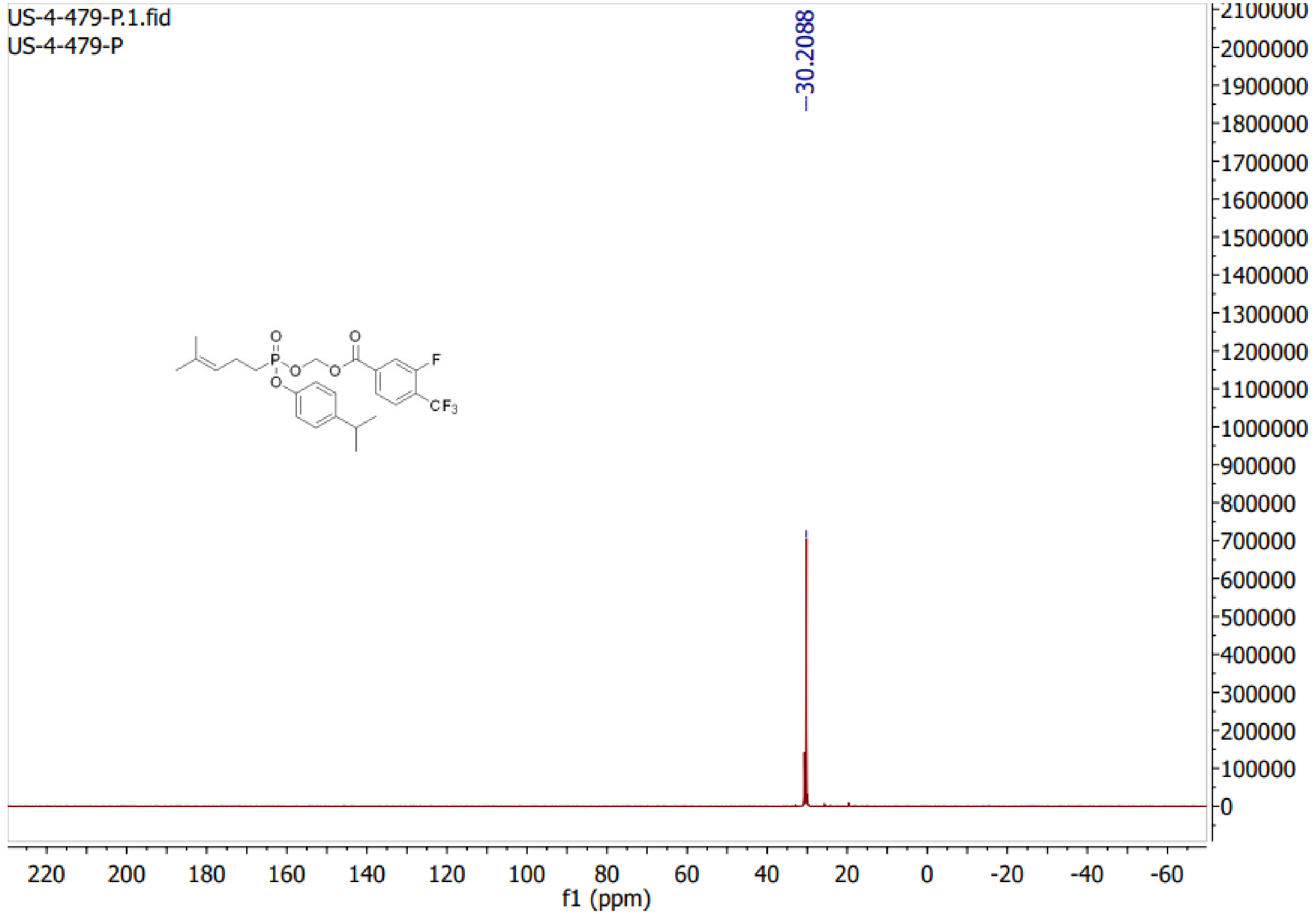

^{31}P NMR Spectrum of Compound **7k** ($CDCl_3$, 203 MHz)

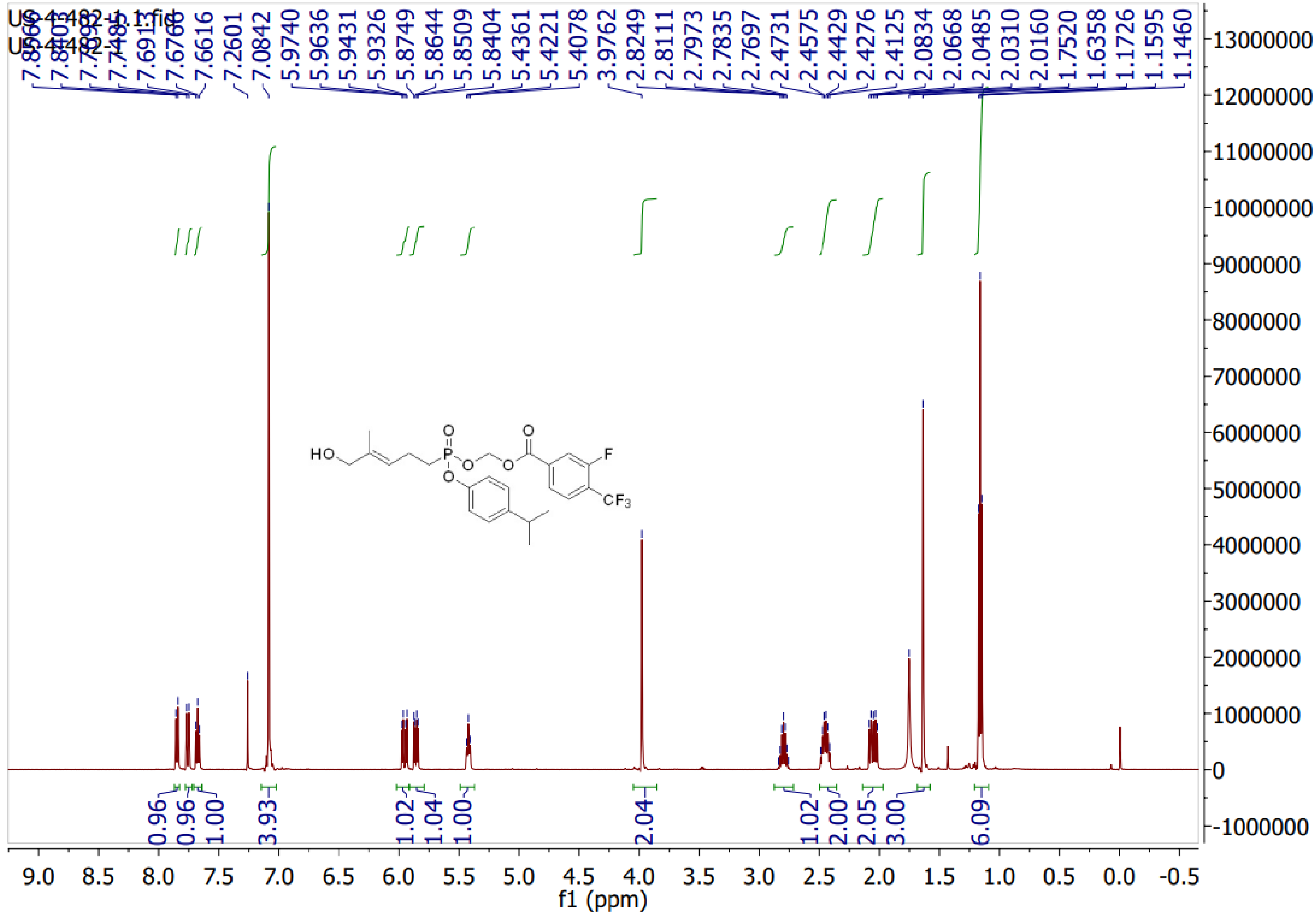

^{1}H NMR Spectrum of Compound **8k** ($CDCl_3$, 500 MHz)

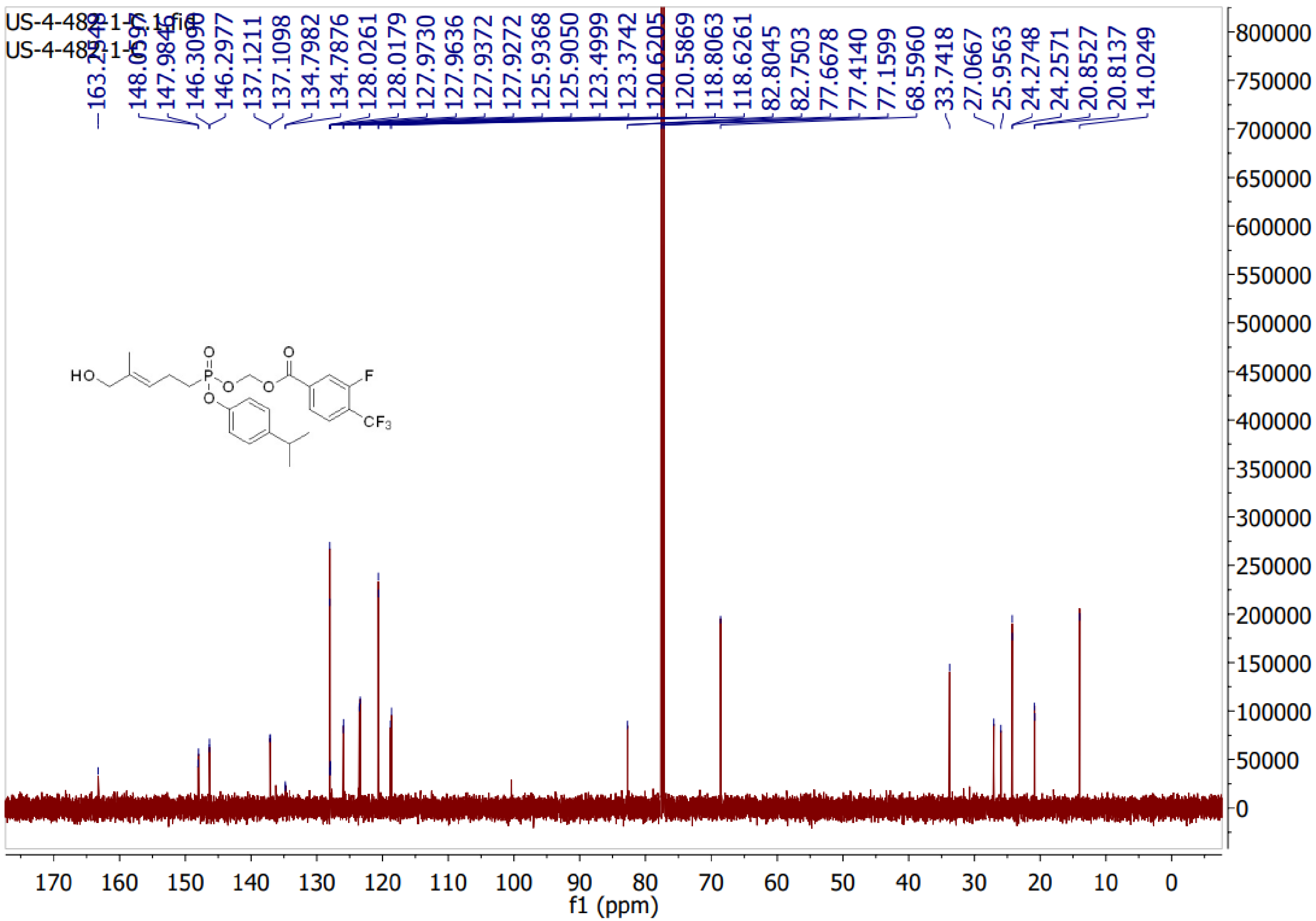

^{13}C NMR Spectrum of Compound **8k** ($CDCl_3$, 126 MHz)

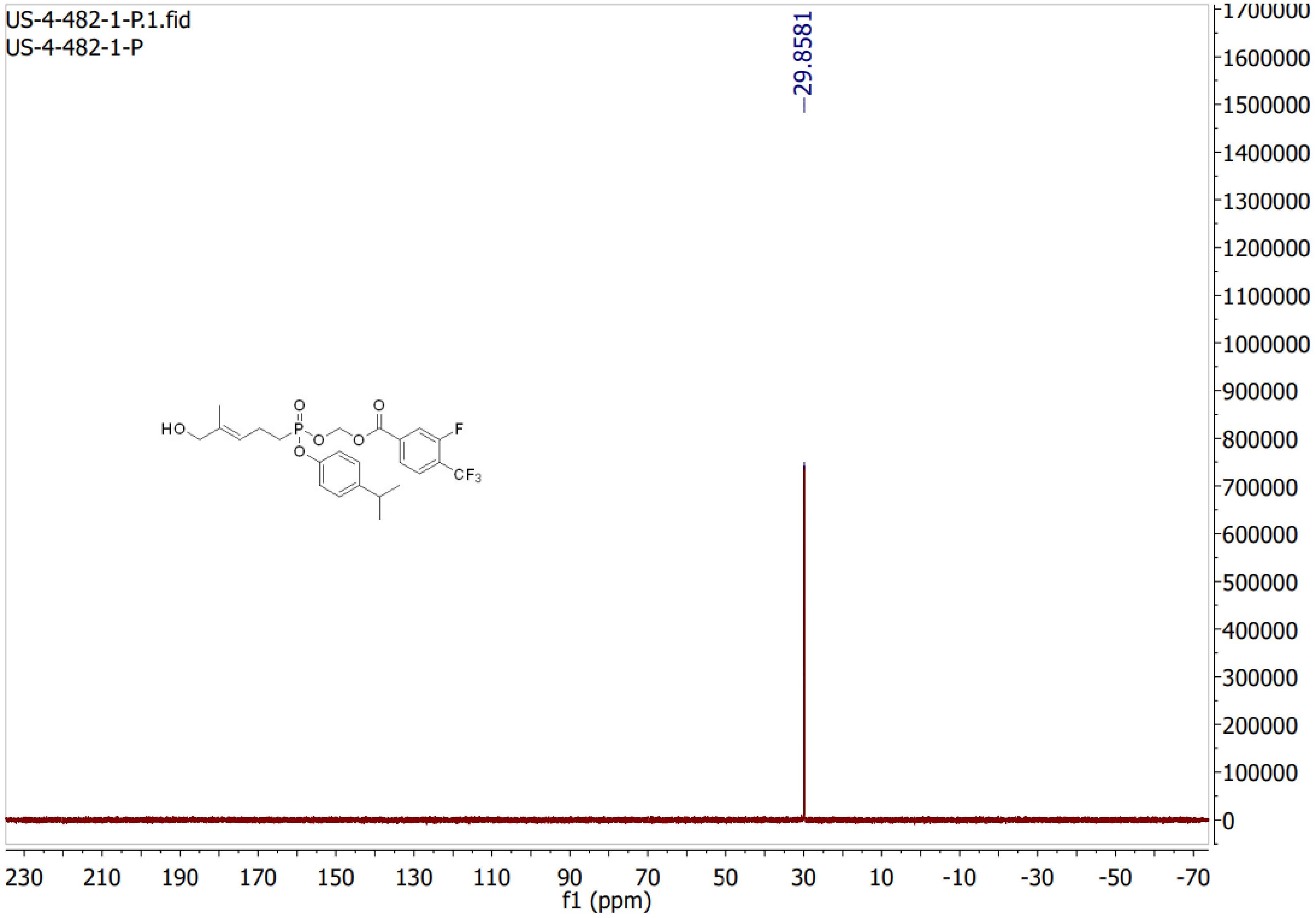

^{31}P NMR Spectrum of Compound **8k** ($CDCl_3$, 203 MHz)

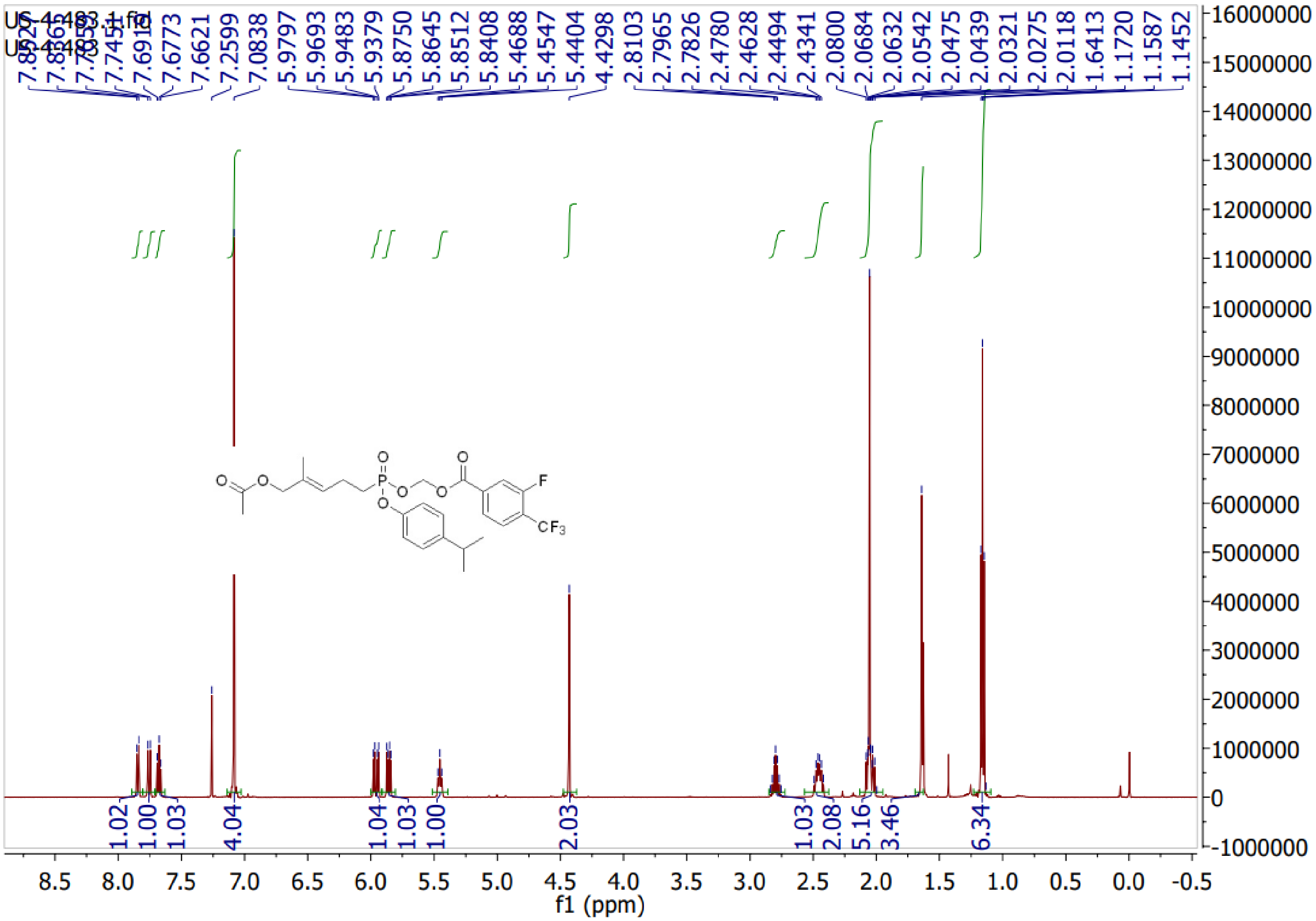

^{1}H NMR Spectrum of Compound **9k** ($CDCl_3$, 500 MHz)

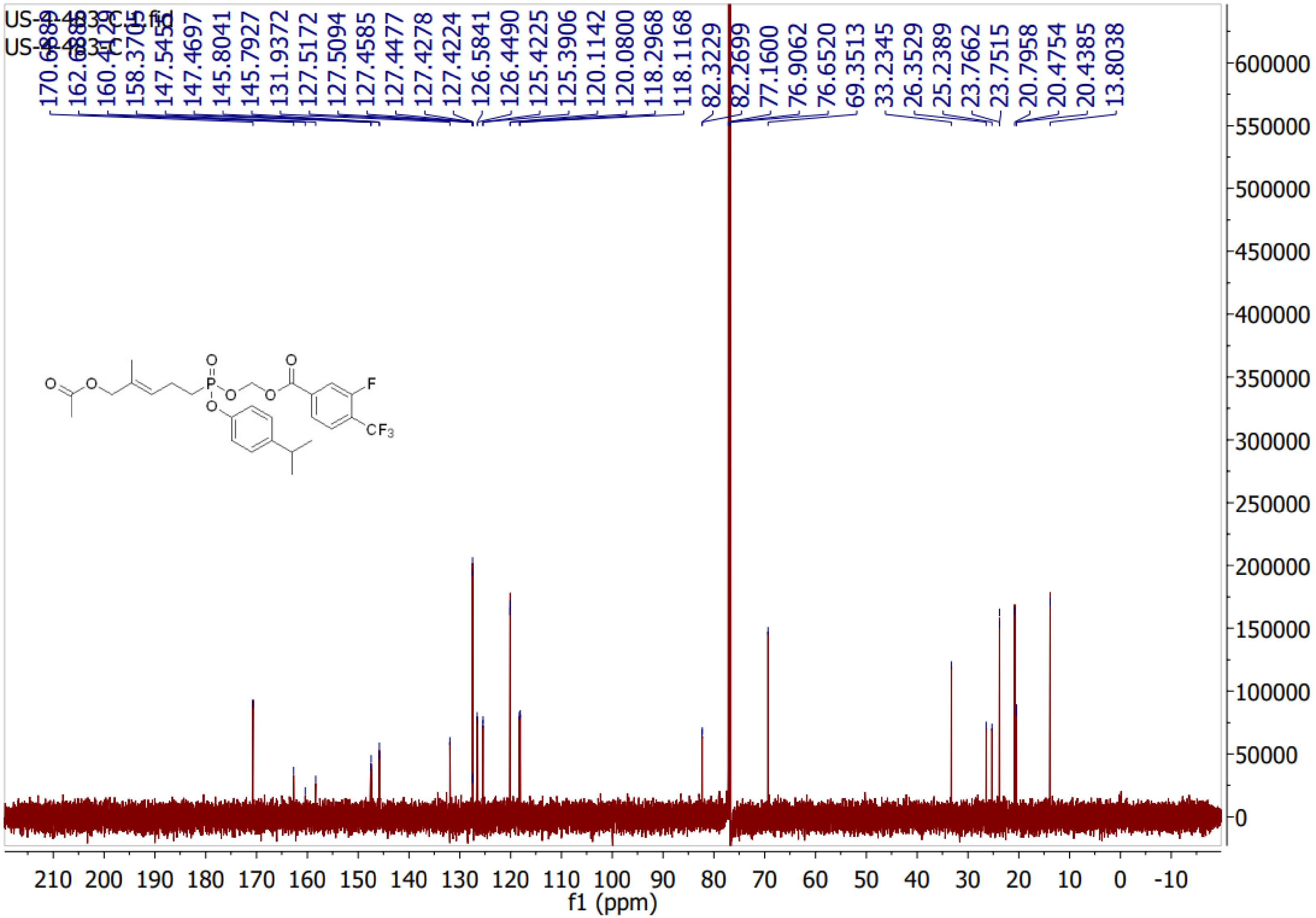

^{13}C NMR Spectrum of Compound **9k** ($CDCl_3$, 126 MHz)

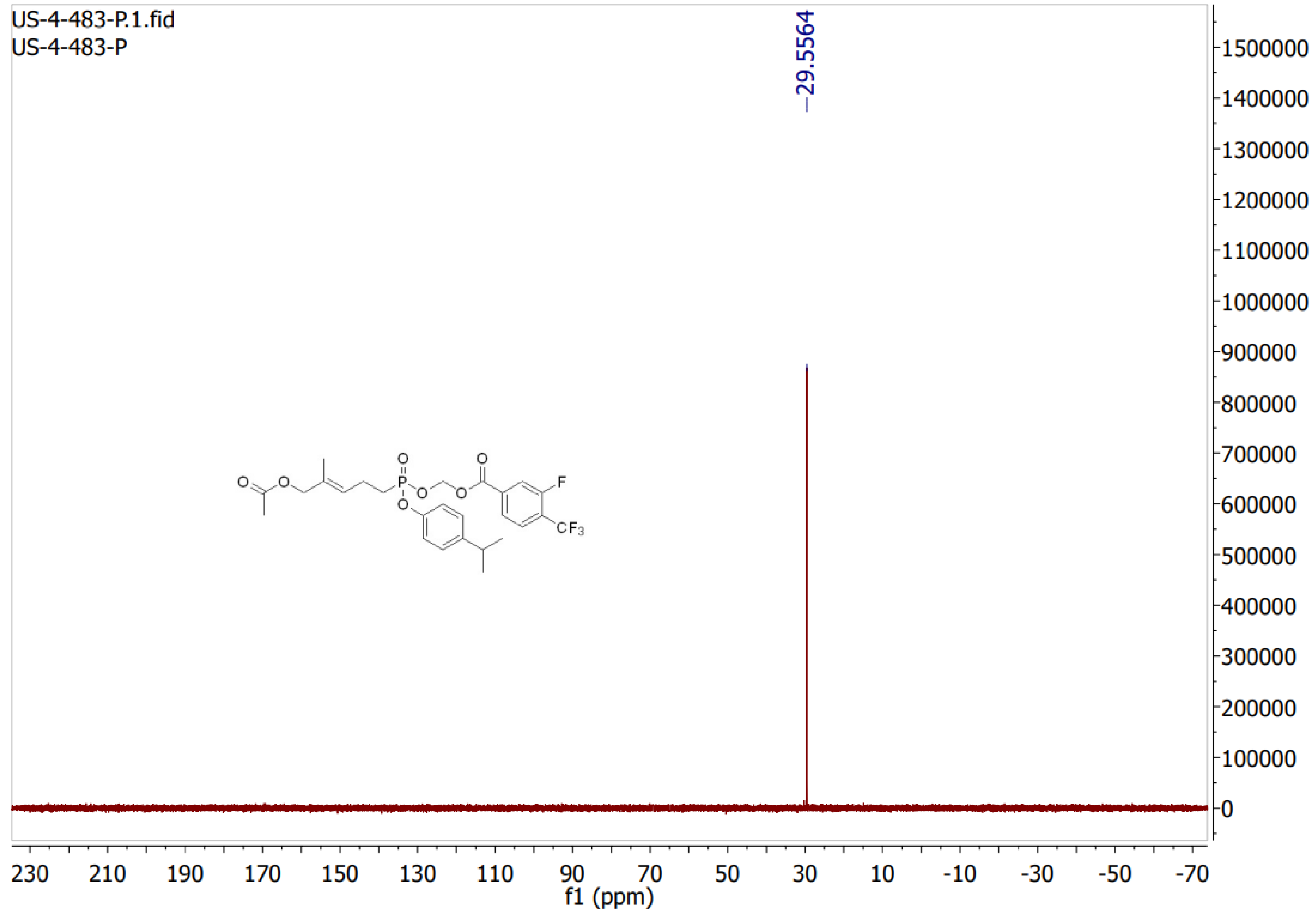

^{31}P NMR Spectrum of Compound **9k** ($CDCl_3$, 203 MHz)

Data File C:\Users\Public\Documents\ChemStation\1\Data\Wiemer\2025-07-0712-15-461PK-137.D
Sample Name: PK-137

===

Acq. Operator : SYSTEM
Sample Operator : SYSTEM
Acq. Instrument : Shared 1220 Location : -
Injection Date : 7/7/2025 12:15:46 PM Inj : 1
Inj Volume : No inj
Acq. Method : C:\Users\Public\Documents\ChemStation\1\Methods\DEF_LC.M
Last changed : 7/7/2025 11:34:15 AM by SYSTEM
(modified after loading)
Analysis Method : C:\Users\Public\Documents\ChemStation\1\Methods\DEF_LC.M
Last changed : 7/7/2025 5:37:30 PM by SYSTEM
(modified after loading)
Sample Info : PK-137

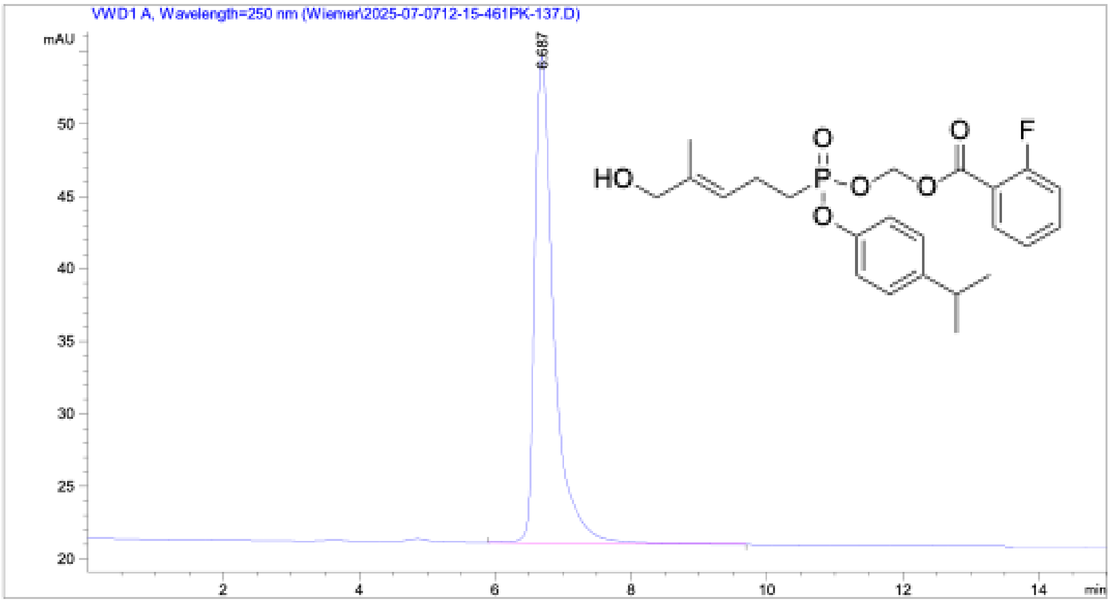

===
Area Percent Report
===

Sorted By : Signal
Multiplier : 1.0000
Dilution : 1.0000
Use Multiplier & Dilution Factor with ISTDs

Signal 1: VWD1 A, Wavelength=250 nm

Peak #	RetTime [min]	Type	Width [min]	Area [mAU*s]	Height [mAU]	Area %
1	6.687	BB	0.2935	657.33411	33.56043	100.0000

Totals : 657.33411 33.56043

HPLC Chromatogram of Compound **8a**

Data File C:\Users\Public\Documents\ChemStation\1\Data\Wiemer\2025-07-0911-17-041PK-141.D
Sample Name: PK-141

===

Acq. Operator : SYSTEM
Sample Operator : SYSTEM
Acq. Instrument : Shared 1220 Location : -
Injection Date : 7/9/2025 11:17:05 AM Inj : 1
Inj Volume : No inj
Method : C:\Users\Public\Documents\ChemStation\1\Methods\DEF_LC.M
Last changed : 7/9/2025 10:57:13 AM by SYSTEM
(modified after loading)
Sample Info : PK-141

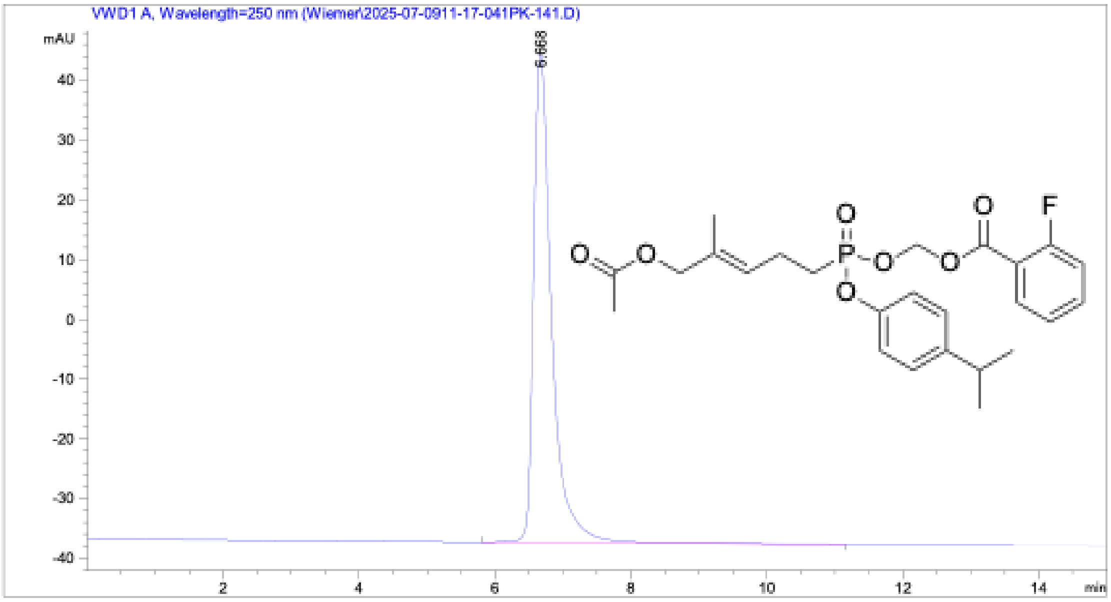

===
Area Percent Report
===

Sorted By : Signal
Multiplier : 1.0000
Dilution : 1.0000
Use Multiplier & Dilution Factor with ISTDs

Signal 1: VWD1 A, Wavelength=250 nm

Peak #	RetTime [min]	Type	Width [min]	Area [mAU*s]	Height [mAU]	Area %
1	6.668	BB	0.2791	1512.00183	81.63802	100.0000

Totals : 1512.00183 81.63802

===
*** End of Report ***

Shared 1220 7/9/2025 11:32:13 AM SYSTEM Page 1 of 1

HPLC Chromatogram of Compound **9a**

Data File C:\Users\P...c\Documents\ChemStation\1\Data\Wiemer\2025-05-1316-04-061US-3-450.D
Sample Name: US-3-450

===

Acq. Operator : SYSTEM
Sample Operator : SYSTEM
Acq. Instrument : Shared 1220 Location : -
Injection Date : 5/13/2025 4:04:06 PM Inj : 1
Inj Volume : No inj
Method : C:\Users\Public\Documents\ChemStation\1\Methods\DEF_LC.M
Last changed : 5/13/2025 4:11:13 PM by SYSTEM
(modified after loading)
Sample Info : US-3-450

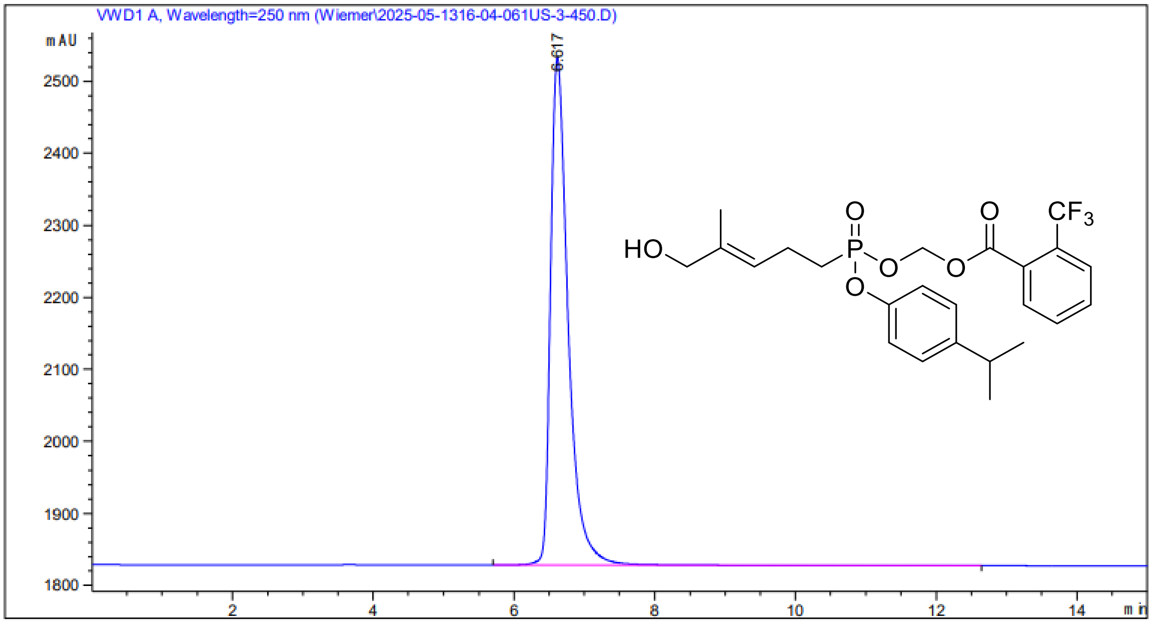

===

Area Percent Report

===

Sorted By : Signal
Multiplier : 1.0000
Dilution : 1.0000
Use Multiplier & Dilution Factor with ISTDs

Signal 1: VWD1 A, Wavelength=250 nm

Peak #	RetTime [min]	Type	Width [min]	Area [mAU*s]	Height [mAU]	Area %
1	6.617	BB	0.2651	1.23329e4	705.01465	100.0000
Totals :				1.23329e4	705.01465	

===

*** End of Report ***

HPLC Chromatogram of Compound **8b**

```
Data File C:\Users\P...c\Documents\ChemStation\1\Data\Wiemer\2025-05-1316-21-421US-3-452.D
Sample Name: US-3-452

=====================================================================
Acq. Operator   : SYSTEM
Sample Operator : SYSTEM
Acq. Instrument : Shared 1220                    Location :     -
Injection Date  : 5/13/2025 4:21:42 PM                Inj :   1
                                               Inj Volume : No inj
Method          : C:\Users\Public\Documents\ChemStation\1\Methods\DEF_LC.M
Last changed    : 5/13/2025 4:11:13 PM by SYSTEM
                  (modified after loading)
Sample Info     : US-3-452
```

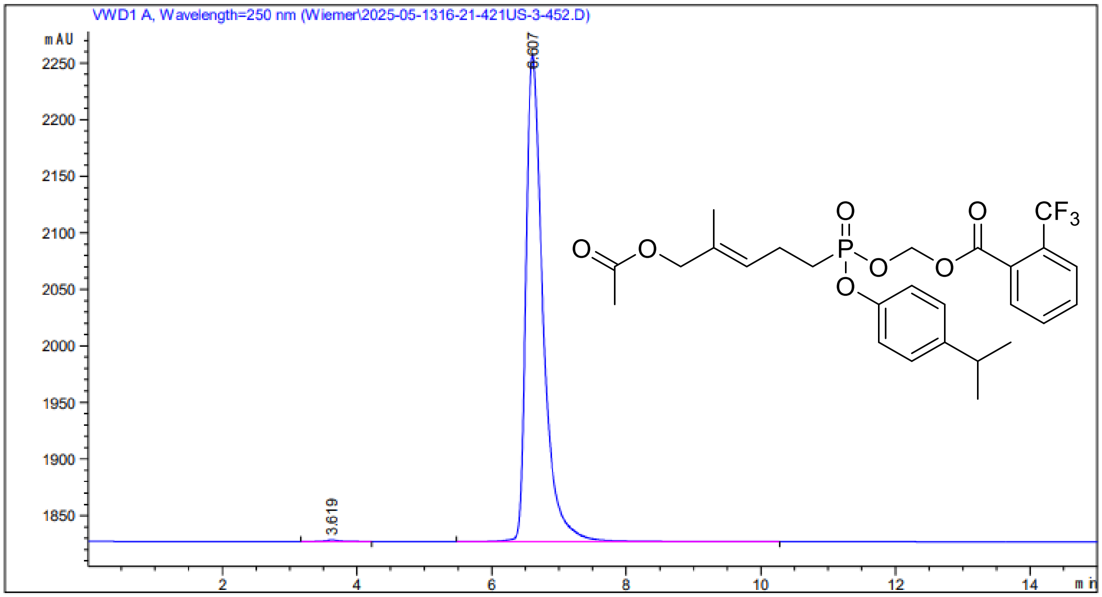


```
=====================================================================
                        Area Percent Report
=====================================================================

Sorted By             :      Signal
Multiplier            :      1.0000
Dilution              :      1.0000
Use Multiplier & Dilution Factor with ISTDs


Signal 1: VWD1 A, Wavelength=250 nm
```

Peak #	RetTime [min]	Type	Width [min]	Area [mAU*s]	Height [mAU]	Area %
1	3.619	BB	0.1884	23.77979	1.71941	0.3110
2	6.607	BB	0.2679	7623.31055	429.90848	99.6890

```
Totals :                 7647.09034  431.62789

=====================================================================
```


HPLC Chromatogram of Compound **9b**

```
Data File C:\Users\Public\Documents\ChemStation\1\Data\Wiemer\2025-07-1009-48-281PK-138.D
Sample Name: PK-138

=====================================================================
Acq. Operator   : SYSTEM
Sample Operator : SYSTEM
Acq. Instrument : Shared 1220                   Location :     -
Injection Date  : 7/10/2025 9:48:29 AM               Inj :   1
                                              Inj Volume : No inj
Method          : C:\Users\Public\Documents\ChemStation\1\Methods\DEF_LC.M
Last changed    : 7/10/2025 9:22:04 AM by SYSTEM
                  (modified after loading)
Sample Info     : PK-138
```

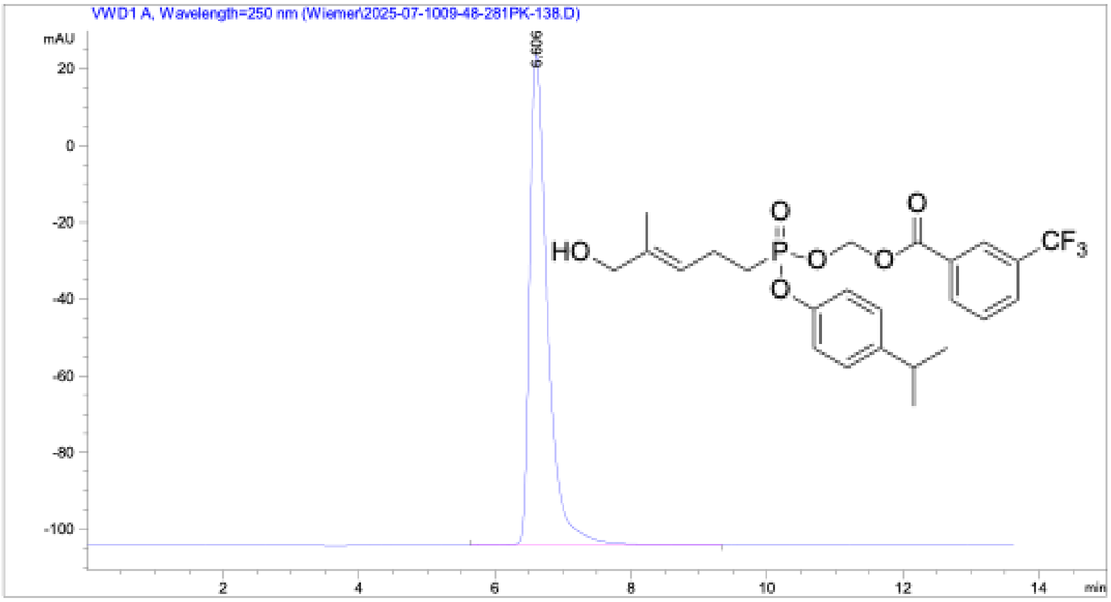


```
=====================================================================
                         Area Percent Report
=====================================================================

Sorted By             :      Signal
Multiplier            :      1.0000
Dilution              :      1.0000
Use Multiplier & Dilution Factor with ISTDs


Signal 1: VWD1 A, Wavelength=250 nm

Peak RetTime Type  Width     Area      Height     Area
  #   [min]        [min]   [mAU*s]     [mAU]        %
----|-------|----|-------|----------|----------|--------|
   1   6.606 BB    0.2757 2339.35474  127.72917 100.0000

Totals :                  2339.35474  127.72917


=====================================================================
                          *** End of Report ***
```

Shared 1220 7/10/2025 10:03:33 AM SYSTEM   Page 1 of 1

HPLC Chromatogram of Compound **8c**

Data File C:\Users\Public\Documents\ChemStation\1\Data\Wiemer\2025-07-1009-04-281PK-142.D
Sample Name: PK-142

===
Acq. Operator : SYSTEM
Sample Operator : SYSTEM
Acq. Instrument : Shared 1220 Location : -
Injection Date : 7/10/2025 9:04:28 AM Inj : 1
Inj Volume : No inj
Acq. Method : C:\Users\Public\Documents\ChemStation\1\Methods\DEF_LC.M
Last changed : 7/10/2025 7:42:12 AM by SYSTEM
(modified after loading)
Analysis Method : C:\Users\Public\Documents\ChemStation\1\Methods\DEF_LC.M
Last changed : 7/10/2025 9:22:04 AM by SYSTEM
(modified after loading)
Sample Info : PK-142

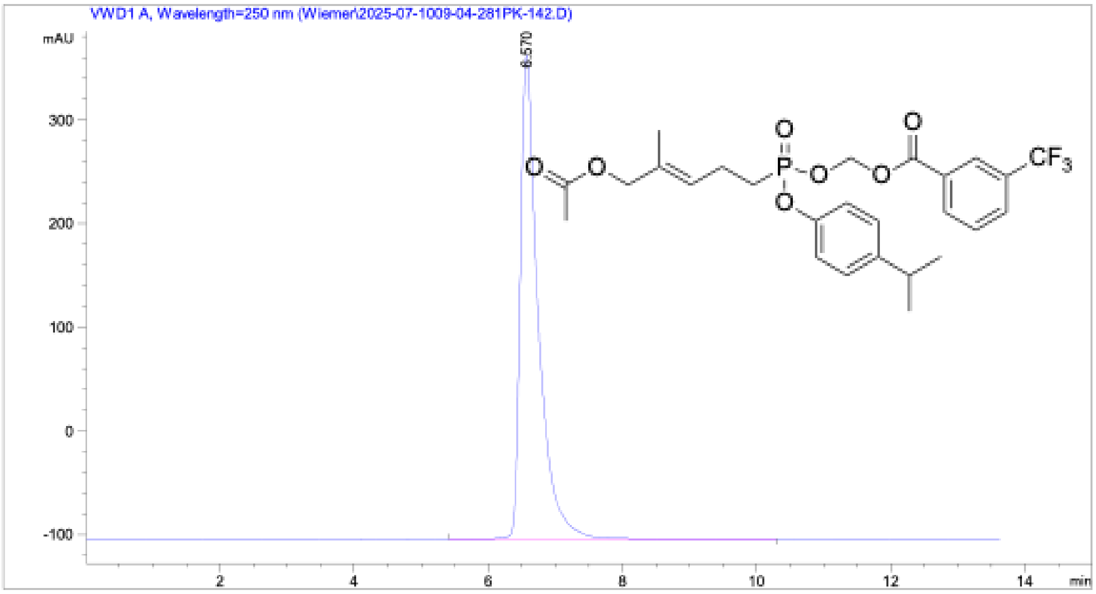

===
Area Percent Report
===

Sorted By : Signal
Multiplier : 1.0000
Dilution : 1.0000
Use Multiplier & Dilution Factor with ISTDs

Signal 1: VWD1 A, Wavelength=250 nm

Peak #	RetTime [min]	Type	Width [min]	Area [mAU*s]	Height [mAU]	Area %
1	6.570	BB	0.2699	8672.56738	466.56262	100.0000

Totals : 8672.56738 466.56262

HPLC Chromatogram of Compound **9c**

```
Data File C:\Users\P...c\Documents\ChemStation\1\Data\Wiemer\2025-01-0312-14-081US-3-383.D
Sample Name: US-3-383

    =====================================================================
    Acq. Operator   : SYSTEM
    Sample Operator : SYSTEM
    Acq. Instrument : Shared 1220                    Location :     -
    Injection Date  : 1/3/2025 12:14:09 PM                Inj :    1
                                                   Inj Volume : No inj
    Method          : C:\USERS\PUBLIC\DOCUMENTS\CHEMSTATION\1\METHODS\MAA-A209.M
    Last changed    : 1/3/2025 12:22:39 PM by SYSTEM
                      (modified after loading)
    Sample Info     : US-3-383
```

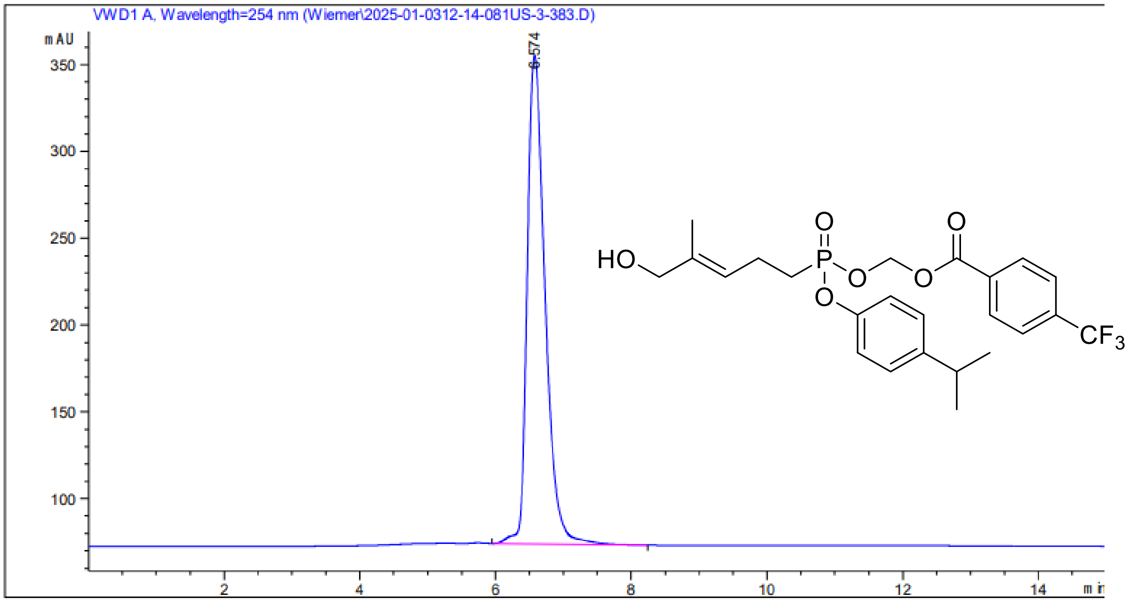


```
    =====================================================================
                         Area Percent Report
    =====================================================================

    Sorted By             :      Signal
    Multiplier            :      1.0000
    Dilution              :      1.0000
    Use Multiplier & Dilution Factor with ISTDs


    Signal 1: VWD1 A, Wavelength=254 nm

    Peak RetTime Type  Width     Area      Height     Area
      #   [min]        [min]   [mAU*s]     [mAU]        %
    ----|-------|----|-------|----------|----------|--------|
       1   6.574 BB    0.2801 5136.97217  281.27286 100.0000

    Totals :                  5136.97217  281.27286


    =====================================================================
                          *** End of Report ***

Shared 1220 1/3/2025 12:29:15 PM SYSTEM                                  Page    1 of 1
```

HPLC Chromatogram of Compound **8d**

```
Data File C:\Users\P...c\Documents\ChemStation\1\Data\Wiemer\2025-01-0312-33-401US-3-385.D
Sample Name: US-3-385

=====================================================================
Acq. Operator   : SYSTEM
Sample Operator : SYSTEM
Acq. Instrument : Shared 1220                     Location :     -
Injection Date  : 1/3/2025 12:33:40 PM                 Inj :   1
                                               Inj Volume : No inj
Method          : C:\USERS\PUBLIC\DOCUMENTS\CHEMSTATION\1\METHODS\MAA-A209.M
Last changed    : 1/3/2025 12:22:39 PM by SYSTEM
                  (modified after loading)
Sample Info     : US-3-385
```

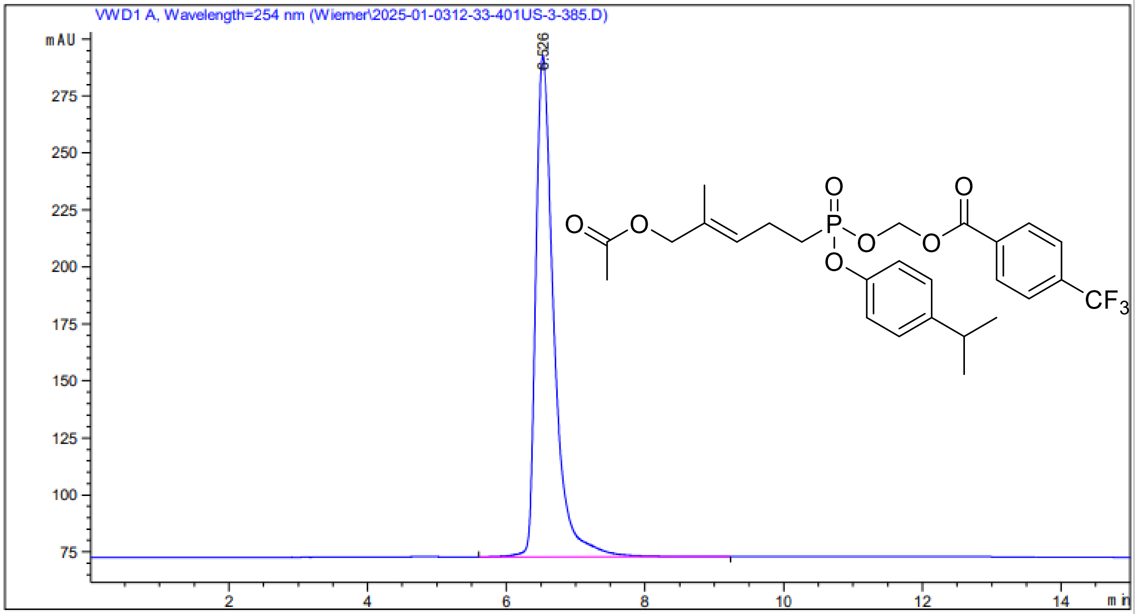


```
=====================================================================
                         Area Percent Report
=====================================================================

Sorted By             :      Signal
Multiplier            :      1.0000
Dilution              :      1.0000
Use Multiplier & Dilution Factor with ISTDs


Signal 1: VWD1 A, Wavelength=254 nm

Peak RetTime Type  Width     Area      Height     Area
  #   [min]        [min]   [mAU*s]     [mAU]       %
----|-------|----|-------|----------|----------|--------|
   1   6.526 BB    0.2823 4046.30884  219.31018 100.0000

Totals :                  4046.30884  219.31018


=====================================================================
                         *** End of Report ***
```

Shared 1220 1/3/2025 12:48:45 PM SYSTEM Page 1 of 1

HPLC Chromatogram of Compound **9d**

Data File C:\Users\P...c\Documents\ChemStation\1\Data\Wiemer\2025-02-1215-16-151US-3-400.D
Sample Name: US-3-400

===

Acq. Operator : SYSTEM
Sample Operator : SYSTEM
Acq. Instrument : Shared 1220 Location : -
Injection Date : 2/12/2025 3:16:15 PM Inj : 1
Inj Volume : No inj
Method : C:\USERS\PUBLIC\DOCUMENTS\CHEMSTATION\1\METHODS\MAA-A209.M
Last changed : 2/12/2025 2:58:33 PM by SYSTEM
(modified after loading)
Sample Info : US-3-400

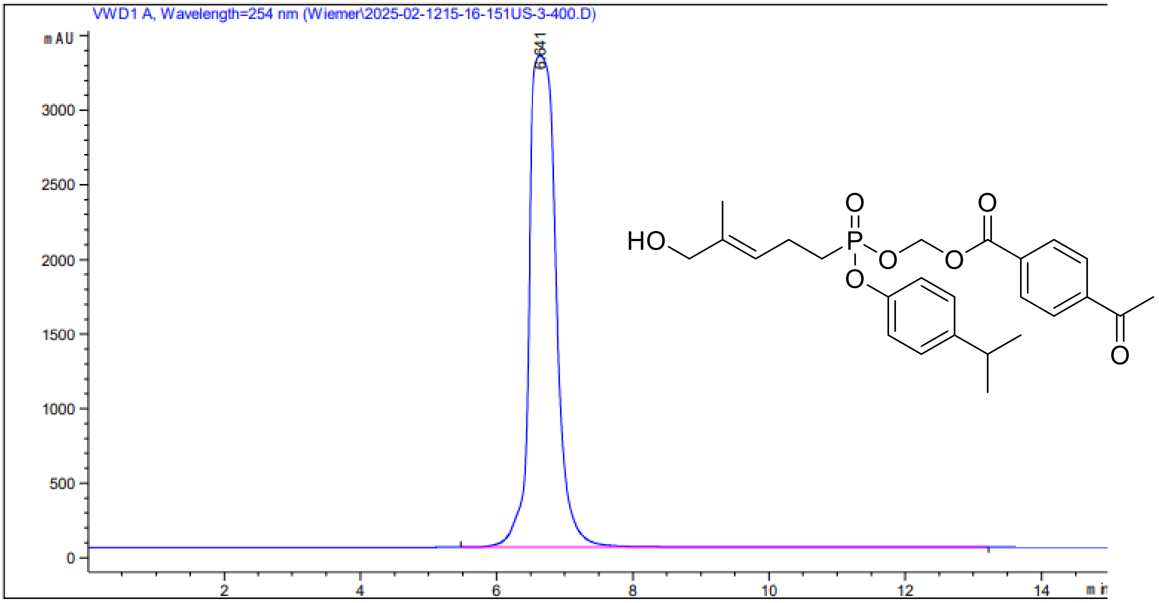

===

Area Percent Report

===

Sorted By : Signal
Multiplier : 1.0000
Dilution : 1.0000
Use Multiplier & Dilution Factor with ISTDs

Signal 1: VWD1 A, Wavelength=254 nm

Peak #	RetTime [min]	Type	Width [min]	Area [mAU*s]	Height [mAU]	Area %
1	6.641	BB	0.4311	9.07466e4	3297.33203	100.0000

Totals : 9.07466e4 3297.33203

===

*** End of Report ***

Shared 1220 2/12/2025 3:31:20 PM SYSTEM Page 1 of 1

HPLC Chromatogram of Compound **8e**

Data File C:\Users\P...c\Documents\ChemStation\1\Data\Wiemer\2025-02-1214-57-411US-3-403.D
Sample Name: US-3-403

===

Acq. Operator : SYSTEM
Sample Operator : SYSTEM
Acq. Instrument : Shared 1220 Location : -
Injection Date : 2/12/2025 2:57:41 PM Inj : 1
Inj Volume : No inj
Method : C:\USERS\PUBLIC\DOCUMENTS\CHEMSTATION\1\METHODS\MAA-A209.M
Last changed : 2/12/2025 2:58:33 PM by SYSTEM
(modified after loading)
Sample Info : US-3-403

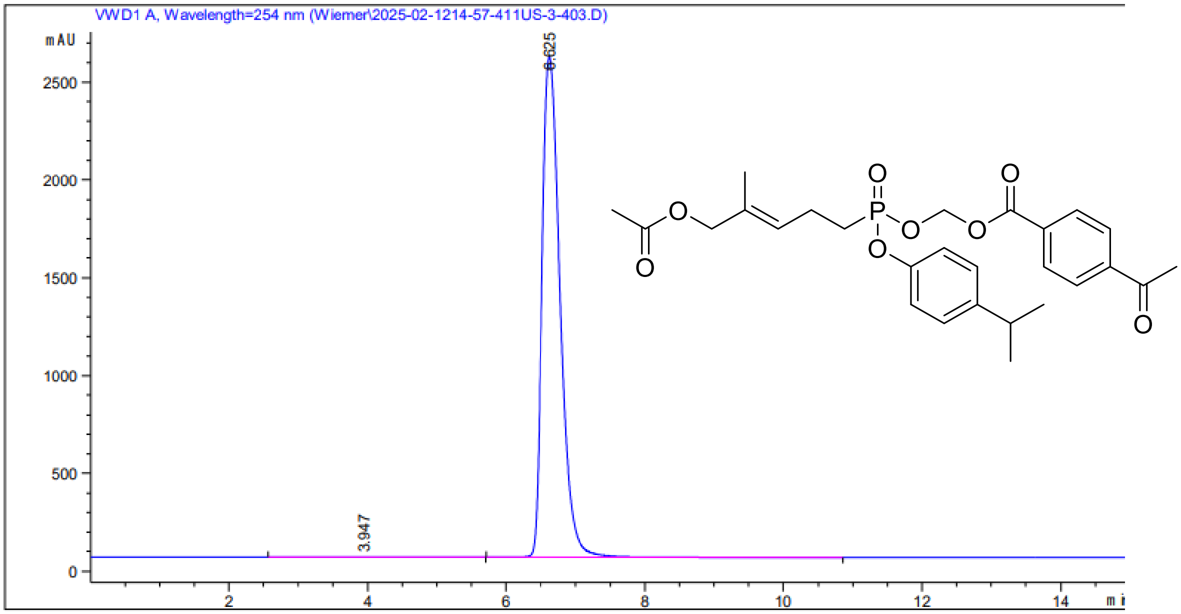

===

Area Percent Report

===

Sorted By : Signal
Multiplier : 1.0000
Dilution : 1.0000
Use Multiplier & Dilution Factor with ISTDs

Signal 1: VWD1 A, Wavelength=254 nm

Peak #	RetTime [min]	Type	Width [min]	Area [mAU*s]	Height [mAU]	Area %
1	3.947	BB	1.0204	176.17079	2.03830	0.3814
2	6.625	BB	0.2808	4.60163e4	2559.21411	99.6186

Totals : 4.61925e4 2561.25241

===

HPLC Chromatogram of Compound **9e**

Data File C:\Users\Public\Documents\ChemStation\1\Data\Wiemer\2025-07-2414-36-331PK-148.D
Sample Name: PK-148

===

Acq. Operator : SYSTEM
Sample Operator : SYSTEM
Acq. Instrument : Shared 1220 Location : -
Injection Date : 7/24/2025 2:36:34 PM Inj : 1
Inj Volume : No inj
Method : C:\Users\Public\Documents\ChemStation\1\Methods\DEF_LC.M
Last changed : 7/24/2025 1:34:02 PM by SYSTEM
(modified after loading)
Sample Info : PK-148

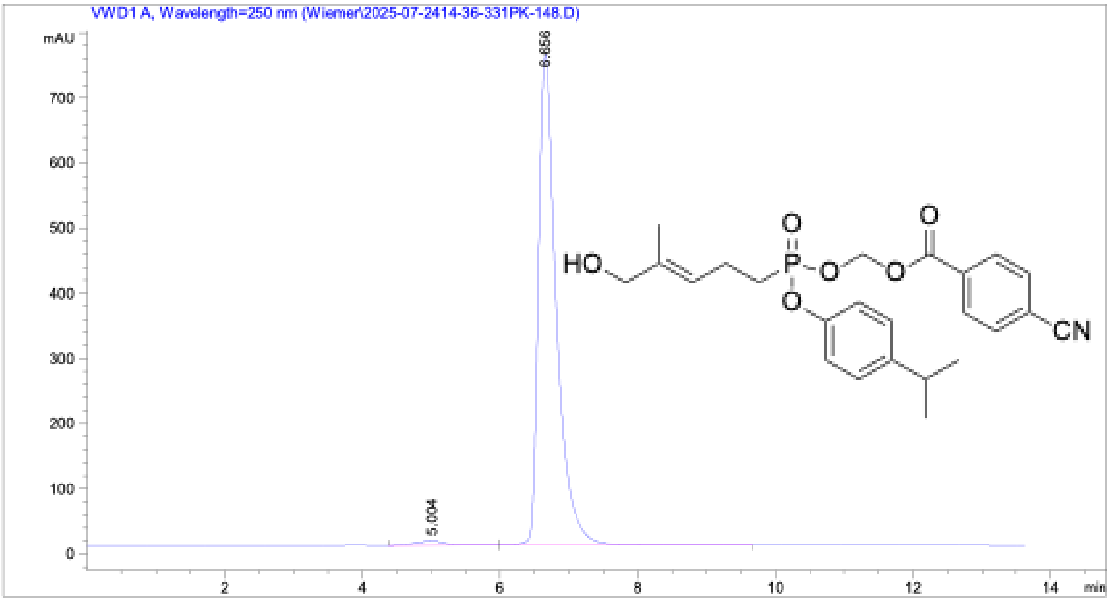

===

Area Percent Report

===

Sorted By : Signal
Multiplier : 1.0000
Dilution : 1.0000
Use Multiplier & Dilution Factor with ISTDs

Signal 1: VWD1 A, Wavelength=250 nm

Peak #	RetTime [min]	Type	Width [min]	Area [mAU*s]	Height [mAU]	Area %
1	5.004	BB	0.3541	204.22292	7.58440	1.4581
2	6.656	BB	0.2757	1.38022e4	753.40833	98.5419

Totals : 1.40065e4 760.99273

===

Shared 1220 7/24/2025 2:51:39 PM SYSTEM Page 1 of 2

HPLC Chromatogram of Compound **8f**

Data File C:\Users\Public\Documents\ChemStation\1\Data\Wiemer\2025-07-2413-54-471PK-149.D
Sample Name: PK-149

```
=====================================================================
Acq. Operator   : SYSTEM
Sample Operator : SYSTEM
Acq. Instrument : Shared 1220                    Location :     -
Injection Date  : 7/24/2025 1:54:48 PM                Inj :    1
                                               Inj Volume : No inj
Method          : C:\Users\Public\Documents\ChemStation\1\Methods\DEF_LC.M
Last changed    : 7/24/2025 1:34:02 PM by SYSTEM
                  (modified after loading)
Sample Info     : PK-149
```

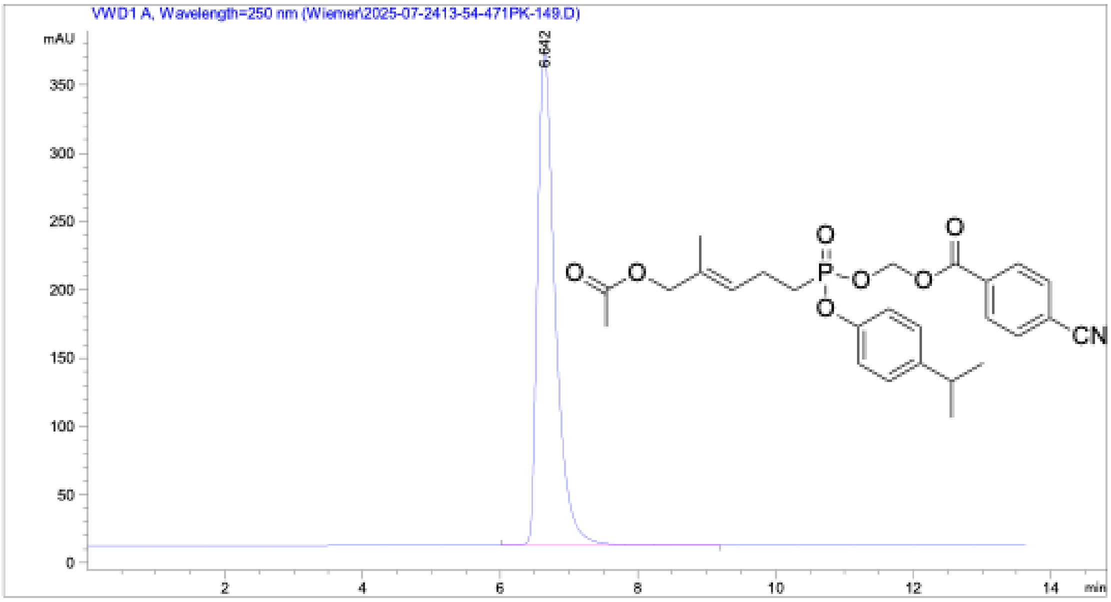


```
=====================================================================
                         Area Percent Report
=====================================================================

Sorted By             :      Signal
Multiplier            :      1.0000
Dilution              :      1.0000
Use Multiplier & Dilution Factor with ISTDs


Signal 1: VWD1 A, Wavelength=250 nm

Peak RetTime Type  Width     Area      Height     Area
  #   [min]        [min]   [mAU*s]     [mAU]        %
----|-------|----|-------|----------|----------|--------|
   1   6.642 BB    0.2719 6428.04150  359.04904 100.0000

Totals :                  6428.04150  359.04904


=====================================================================
                          *** End of Report ***
```


HPLC Chromatogram of Compound **9f**

Data File C:\Users\Public\Documents\ChemStation\1\Data\Wiemer\2025-06-2610-44-271PK-126.D
Sample Name: PK-126

```
=====================================================================
Acq. Operator   : SYSTEM
Sample Operator : SYSTEM
Acq. Instrument : Shared 1220                   Location :     -
Injection Date  : 6/26/2025 10:44:28 AM              Inj :    1
                                              Inj Volume : No inj
Method          : C:\Users\Public\Documents\ChemStation\1\Methods\DEF_LC.M
Last changed    : 6/26/2025 9:33:46 AM by SYSTEM
                  (modified after loading)
Sample Info     : PK-126
```

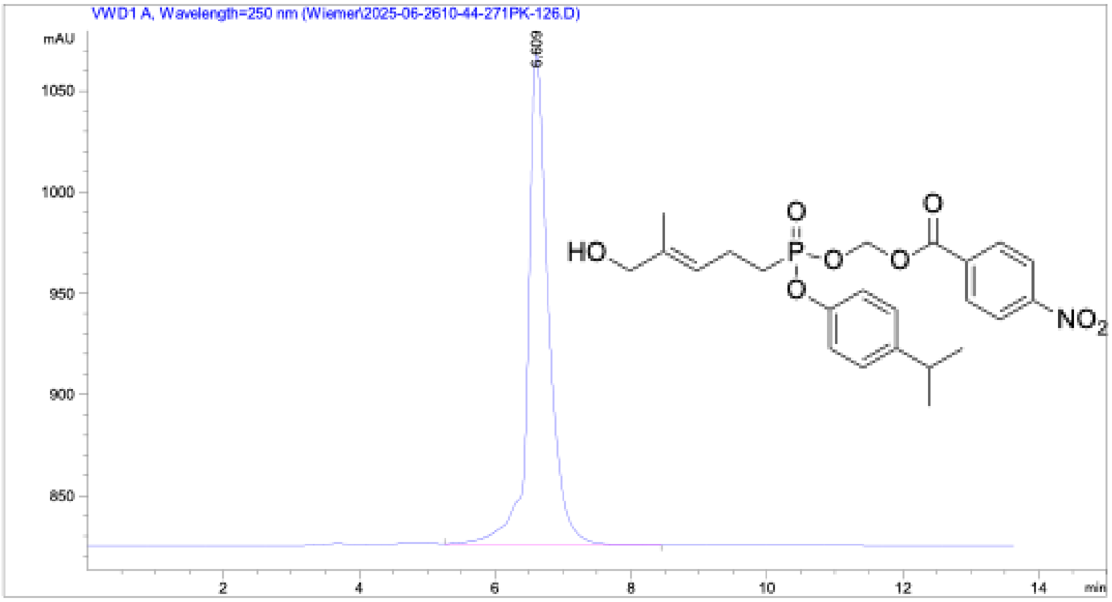


```
=====================================================================
                         Area Percent Report
=====================================================================

Sorted By             :      Signal
Multiplier            :      1.0000
Dilution              :      1.0000
Use Multiplier & Dilution Factor with ISTDs


Signal 1: VWD1 A, Wavelength=250 nm

Peak RetTime Type  Width     Area      Height     Area
  #   [min]        [min]   [mAU*s]     [mAU]        %
----|-------|----|-------|----------|----------|--------|
   1   6.609 BB    0.3072 5104.92139  241.88133 100.0000

Totals :                  5104.92139  241.88133


=====================================================================
                          *** End of Report ***
```

Shared 1220 6/26/2025 10:59:33 AM SYSTEM   Page   1 of 1

HPLC Chromatogram of Compound **8g**

Data File C:\Users\P...c\Documents\ChemStation\1\Data\Wiemer\2025-07-3109-03-581PK-151-2.D
Sample Name: PK-151-2

===

```
Acq. Operator    : SYSTEM
Sample Operator  : SYSTEM
Acq. Instrument  : Shared 1220                    Location :     -
Injection Date   : 7/31/2025 9:03:59 AM                Inj :    1
                                               Inj Volume : No inj
Method           : C:\Users\Public\Documents\ChemStation\1\Methods\DEF_LC.M
Last changed     : 7/31/2025 8:13:22 AM by SYSTEM
                   (modified after loading)
Sample Info      : PK-151-2
```

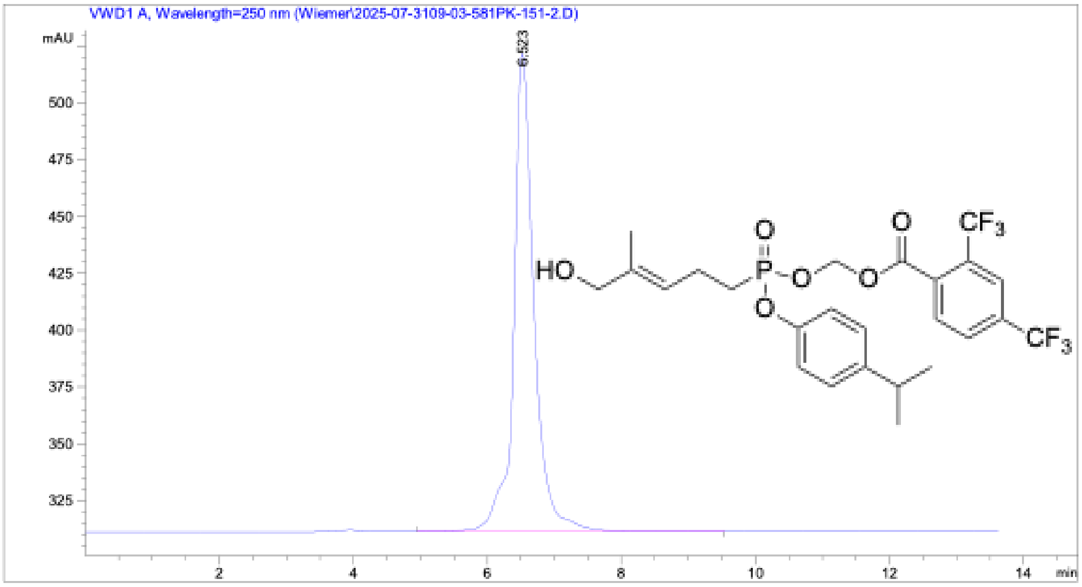

===

Area Percent Report

===

```
Sorted By              :      Signal
Multiplier             :      1.0000
Dilution               :      1.0000
Use Multiplier & Dilution Factor with ISTDs
```

Signal 1: VWD1 A, Wavelength=250 nm

Peak #	RetTime [min]	Type	Width [min]	Area [mAU*s]	Height [mAU]	Area %
1	6.523	BB	0.3109	4450.83887	210.32596	100.0000

Totals : 4450.83887 210.32596

===

*** End of Report ***

HPLC Chromatogram of Compound **8h**

Data File C:\Users\Public\Documents\ChemStation\1\Data\Wiemer\2025-07-3112-53-021PK-152.D
Sample Name: PK-152

===

Acq. Operator : SYSTEM
Sample Operator : SYSTEM
Acq. Instrument : Shared 1220 Location : -
Injection Date : 7/31/2025 12:53:02 PM Inj : 1
Inj Volume : No inj
Method : C:\Users\Public\Documents\ChemStation\1\Methods\DEF_LC.M
Last changed : 7/31/2025 12:06:59 PM by SYSTEM
(modified after loading)
Sample Info : PK-152

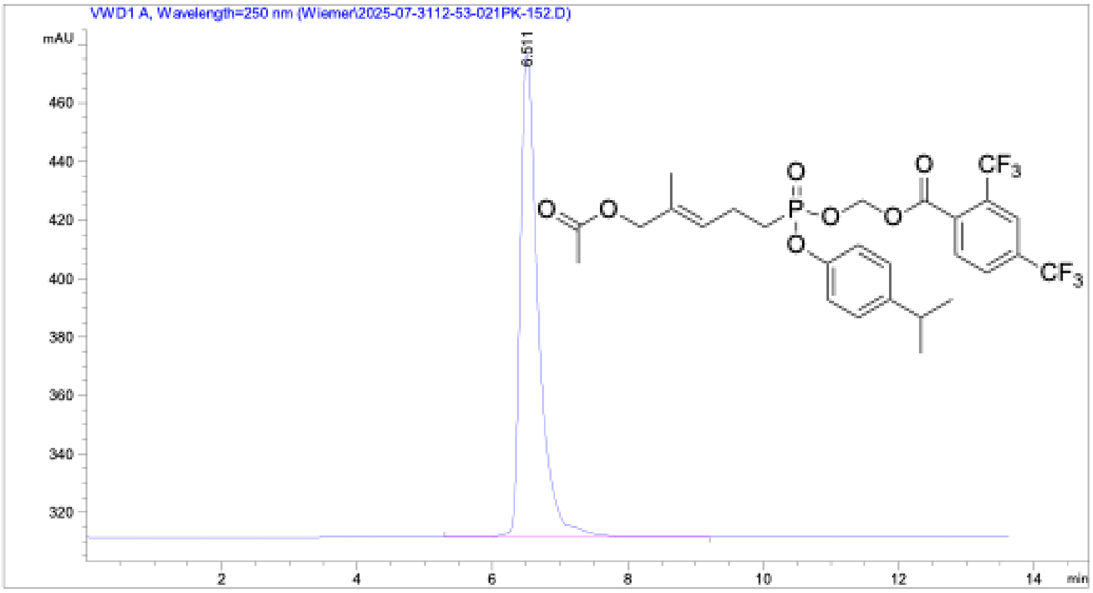

===
Area Percent Report
===

Sorted By : Signal
Multiplier : 1.0000
Dilution : 1.0000
Use Multiplier & Dilution Factor with ISTDs

Signal 1: VWD1 A, Wavelength=250 nm

Peak #	RetTime [min]	Type	Width [min]	Area [mAU*s]	Height [mAU]	Area %
1	6.511	BB	0.2802	3076.04858	165.21829	100.0000

Totals : 3076.04858 165.21829

===
*** End of Report ***

Shared 1220 7/31/2025 1:08:08 PM SYSTEM Page 1 of 1

HPLC Chromatogram of Compound **9h**

Data File C:\Users\P...c\Documents\ChemStation\1\Data\Wiemer\2025-06-2513-16-311US-4-477.D
Sample Name: US-4-477

===
Acq. Operator : SYSTEM
Sample Operator : SYSTEM
Acq. Instrument : Shared 1220 Location : -
Injection Date : 6/25/2025 1:16:32 PM Inj : 1
Inj Volume : No inj
Method : C:\Users\Public\Documents\ChemStation\1\Methods\DEF_LC.M
Last changed : 6/25/2025 1:00:26 PM by SYSTEM
(modified after loading)
Sample Info : US-4-477

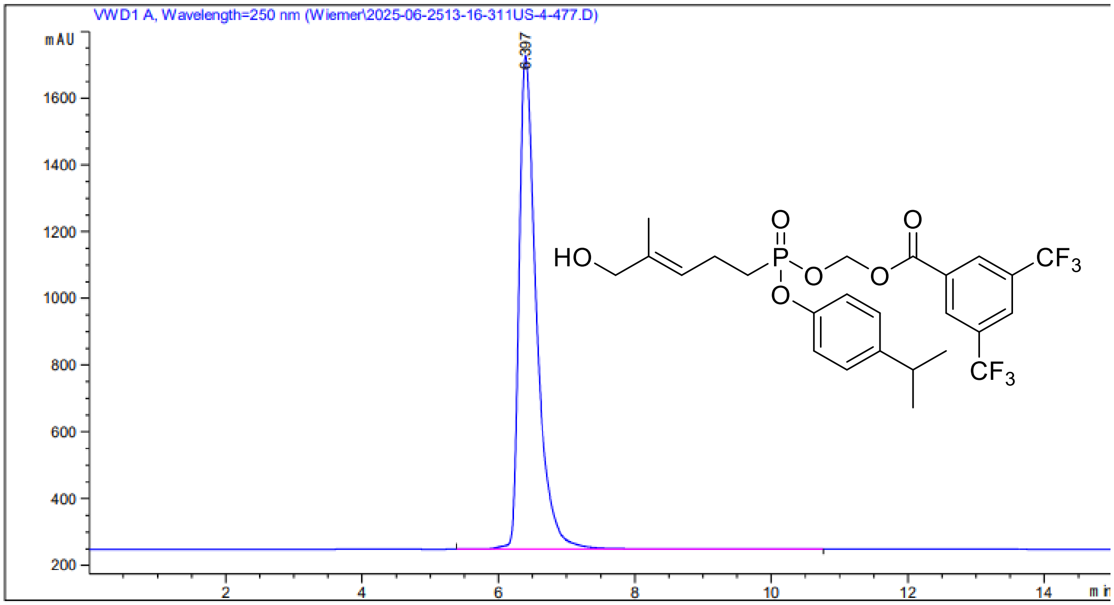

===
Area Percent Report
===

Sorted By : Signal
Multiplier : 1.0000
Dilution : 1.0000
Use Multiplier & Dilution Factor with ISTDs

Signal 1: VWD1 A, Wavelength=250 nm

Peak #	RetTime [min]	Type	Width [min]	Area [mAU*s]	Height [mAU]	Area %
1	6.397	BB	0.2694	2.63930e4	1477.51965	100.0000

Totals : 2.63930e4 1477.51965

===
*** End of Report ***

HPLC Chromatogram of Compound **8i**

Data File C:\Users\P...Documents\ChemStation\1\Data\Wiemer\2025-06-2610-10-171US-4-481-3.D
Sample Name: US-4-481-3

===

Acq. Operator : SYSTEM
Sample Operator : SYSTEM
Acq. Instrument : Shared 1220 Location : -
Injection Date : 6/26/2025 10:10:17 AM Inj : 1
Inj Volume : No inj
Method : C:\Users\Public\Documents\ChemStation\1\Methods\DEF_LC.M
Last changed : 6/26/2025 9:33:46 AM by SYSTEM
(modified after loading)
Sample Info : US-4-481-3

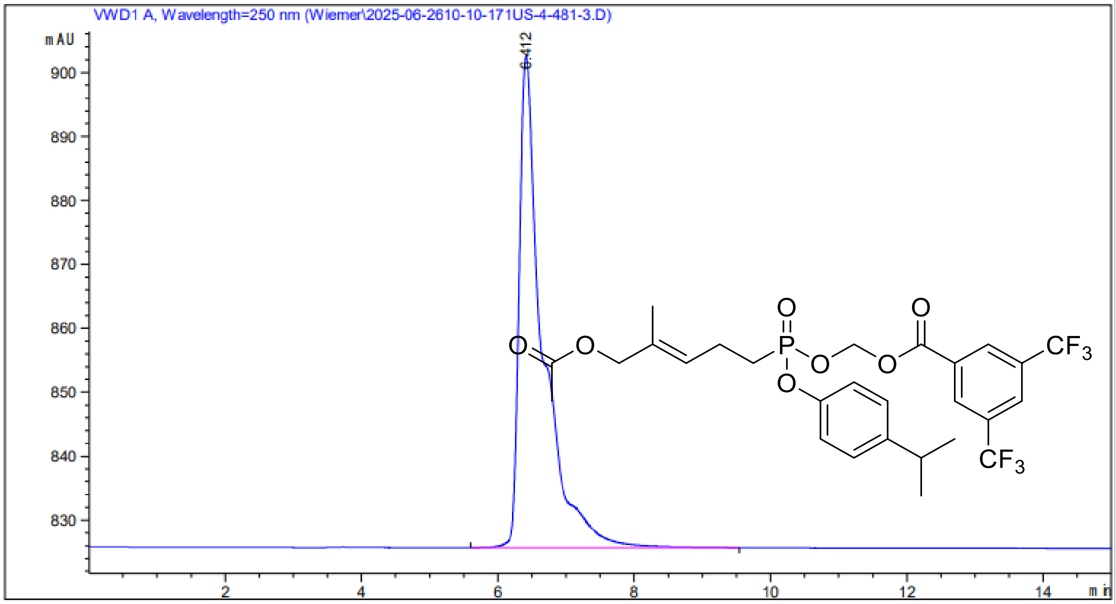

===

Area Percent Report

===

Sorted By : Signal
Multiplier : 1.0000
Dilution : 1.0000
Use Multiplier & Dilution Factor with ISTDs

Signal 1: VWD1 A, Wavelength=250 nm

Peak #	RetTime [min]	Type	Width [min]	Area [mAU*s]	Height [mAU]	Area %
1	6.412	BB	0.3434	1860.96558	76.86928	100.0000

Totals : 1860.96558 76.86928

===

*** End of Report ***

HPLC Chromatogram of Compound **9i**

```
Data File C:\Users\Public\Documents\ChemStation\1\Data\Wiemer\2025-06-2611-15-201PK-130.D
Sample Name: PK-130

    =====================================================================
    Acq. Operator   : SYSTEM
    Sample Operator : SYSTEM
    Acq. Instrument : Shared 1220                    Location :     -
    Injection Date  : 6/26/2025 11:15:21 AM               Inj :    1
                                                   Inj Volume : No inj
    Method          : C:\Users\Public\Documents\ChemStation\1\Methods\DEF_LC.M
    Last changed    : 6/26/2025 9:33:46 AM by SYSTEM
                      (modified after loading)
    Sample Info     : PK-130
```

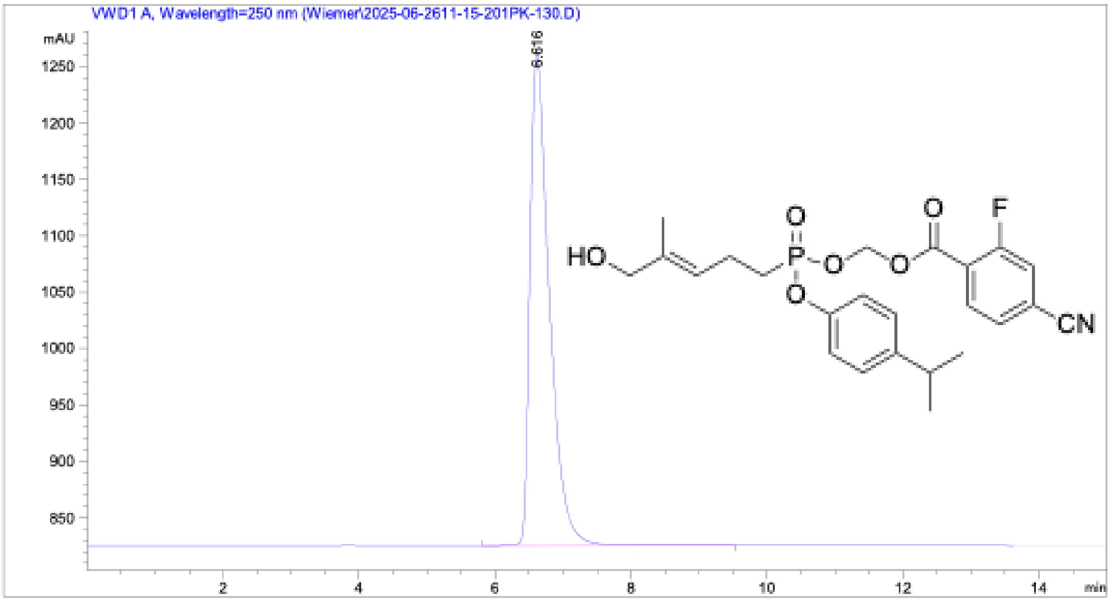


```
    =====================================================================
                             Area Percent Report
    =====================================================================

    Sorted By             :      Signal
    Multiplier            :      1.0000
    Dilution              :      1.0000
    Use Multiplier & Dilution Factor with ISTDs


    Signal 1: VWD1 A, Wavelength=250 nm

    Peak RetTime Type  Width     Area      Height     Area
      #   [min]        [min]   [mAU*s]     [mAU]        %
    ----|-------|----|-------|----------|----------|--------|
       1   6.616 BB    0.2952 8619.97461  434.88681 100.0000

    Totals :                  8619.97461  434.88681


    =====================================================================
                              *** End of Report ***
```

Shared 1220 6/26/2025 11:30:26 AM SYSTEM   Page   1 of 1

HPLC Chromatogram of Compound **8j**

Data File C:\Users\Public\Documents\ChemStation\1\Data\Wiemer\2025-06-2611-56-301PK-134.D
Sample Name: PK-134

===
Acq. Operator : SYSTEM
Sample Operator : SYSTEM
Acq. Instrument : Shared 1220 Location : -
Injection Date : 6/26/2025 11:56:31 AM Inj : 1
Inj Volume : No inj
Method : C:\Users\Public\Documents\ChemStation\1\Methods\DEF_LC.M
Last changed : 6/26/2025 9:33:46 AM by SYSTEM
(modified after loading)
Sample Info : PK-134

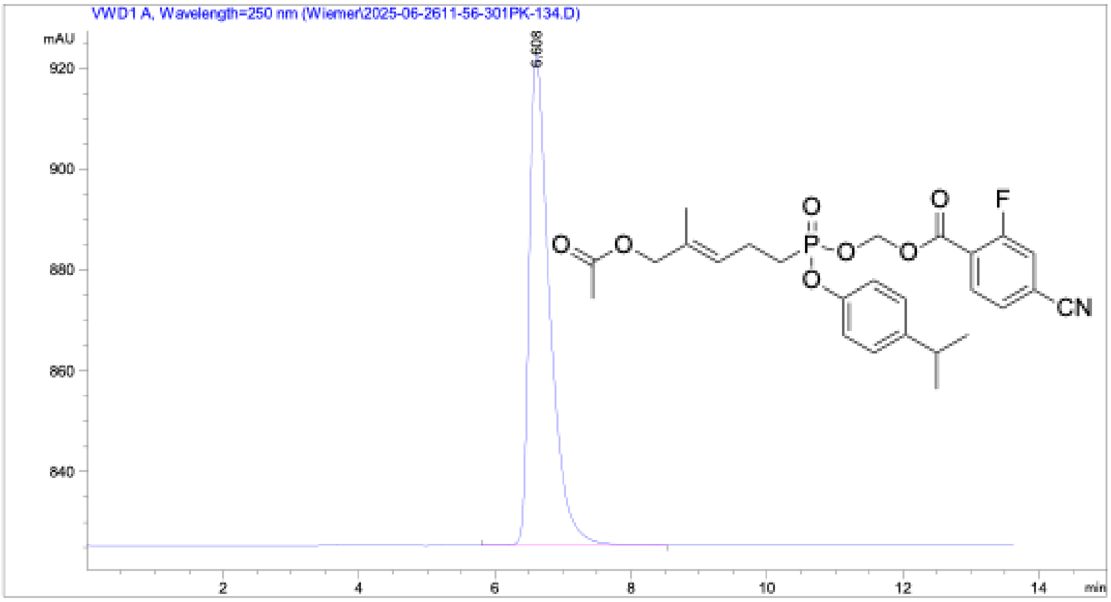

===
Area Percent Report
===

Sorted By : Signal
Multiplier : 1.0000
Dilution : 1.0000
Use Multiplier & Dilution Factor with ISTDs

Signal 1: VWD1 A, Wavelength=250 nm

Peak #	RetTime [min]	Type	Width [min]	Area [mAU*s]	Height [mAU]	Area %
1	6.608	BB	0.3109	2039.86072	97.16035	100.0000
Totals :				2039.86072	97.16035	

===
*** End of Report ***

HPLC Chromatogram of Compound **9j**

Data File C:\Users\P...Documents\ChemStation\1\Data\Wiemer\2025-07-0415-55-401US-4-482-1.D
Sample Name: US-4-482-1

===

Acq. Operator : SYSTEM
Sample Operator : SYSTEM
Acq. Instrument : Shared 1220 Location : -
Injection Date : 7/4/2025 3:55:41 PM Inj : 1
Inj Volume : No inj
Method : C:\Users\Public\Documents\ChemStation\1\Methods\DEF_LC.M
Last changed : 7/4/2025 3:36:56 PM by SYSTEM
(modified after loading)
Sample Info : US-4-482-1

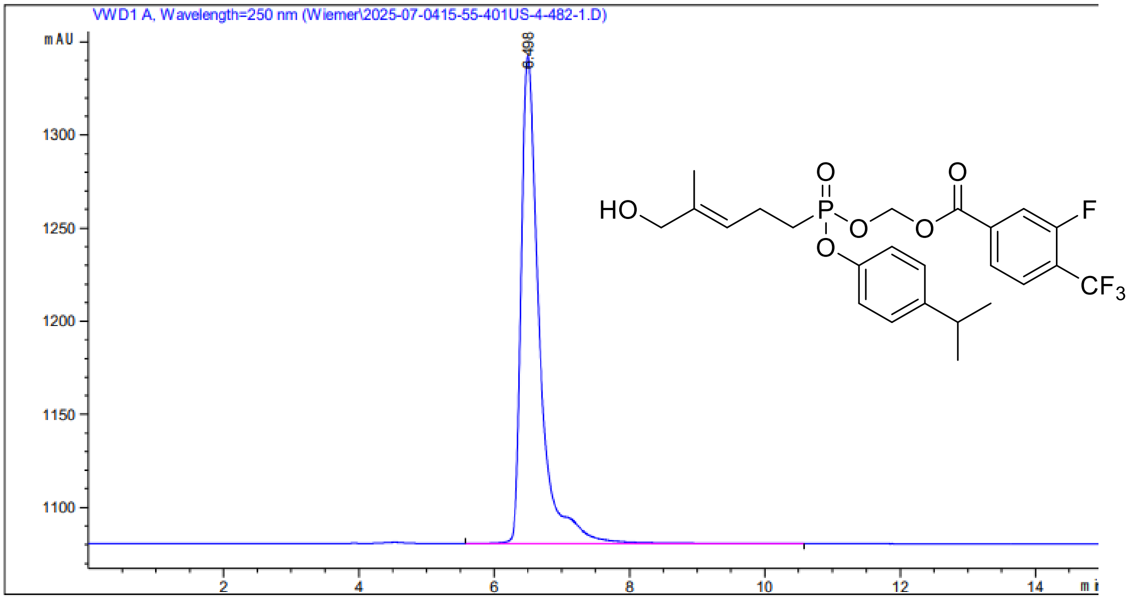

===

Area Percent Report

===

Sorted By : Signal
Multiplier : 1.0000
Dilution : 1.0000
Use Multiplier & Dilution Factor with ISTDs

Signal 1: VWD1 A, Wavelength=250 nm

Peak #	RetTime [min]	Type	Width [min]	Area [mAU*s]	Height [mAU]	Area %
1	6.498	BB	0.2807	4938.47314	262.23917	100.0000

Totals : 4938.47314 262.23917

===

*** End of Report ***

HPLC Chromatogram of Compound **8k**

Data File C:\Users\P...c\Documents\ChemStation\1\Data\Wiemer\2025-07-0713-47-161US-4-483.D
Sample Name: US-4-483

===
Acq. Operator : SYSTEM
Sample Operator : SYSTEM
Acq. Instrument : Shared 1220 Location : -
Injection Date : 7/7/2025 1:47:17 PM Inj : 1
Inj Volume : No inj
Method : C:\Users\Public\Documents\ChemStation\1\Methods\DEF_LC.M
Last changed : 7/7/2025 1:42:55 PM by SYSTEM
(modified after loading)
Sample Info : US-4-483

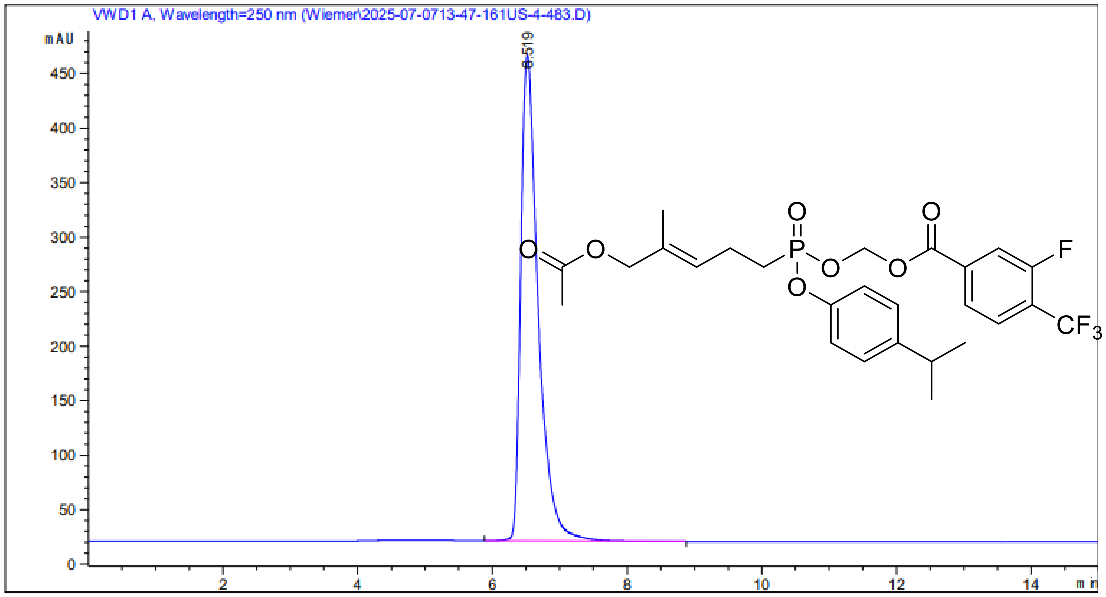

===
Area Percent Report
===

Sorted By : Signal
Multiplier : 1.0000
Dilution : 1.0000
Use Multiplier & Dilution Factor with ISTDs

Signal 1: VWD1 A, Wavelength=250 nm

Peak #	RetTime [min]	Type	Width [min]	Area [mAU*s]	Height [mAU]	Area %
1	6.519	BB	0.2712	8037.48926	446.01971	100.0000

Totals : 8037.48926 446.01971

===
*** End of Report ***

Shared 1220 7/7/2025 2:02:22 PM SYSTEM Page 1 of 1

HPLC Chromatogram of Compound **9k**

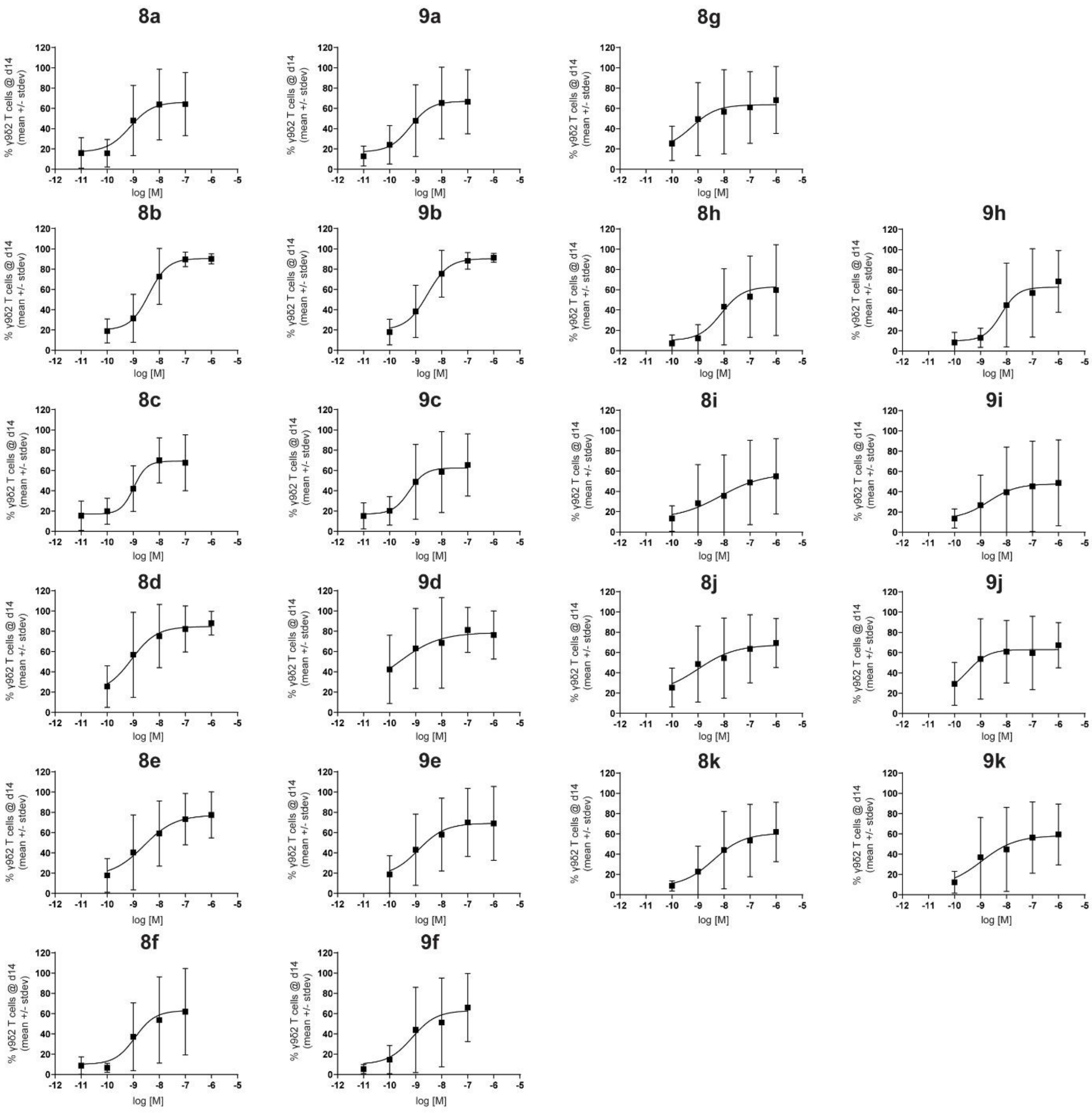

Supplemental Figure S1. Proliferation data used to derive EC_{50} values in the main text. Human PBMCs were treated with the indicated concentrations of compounds for 72 h, allowed to grow for 11 additional days, and proliferation was measured using flow cytometry staining. Each panel shows a dose–response curve for an individual compound. Data are expressed as percentage of γδ T cells at day 14. Error bars represent mean ± standard deviation from three different donors (n=3). Curves were fit using nonlinear regression (log agonist versus response, variable slope four parameters) in GraphPad Prism 10.

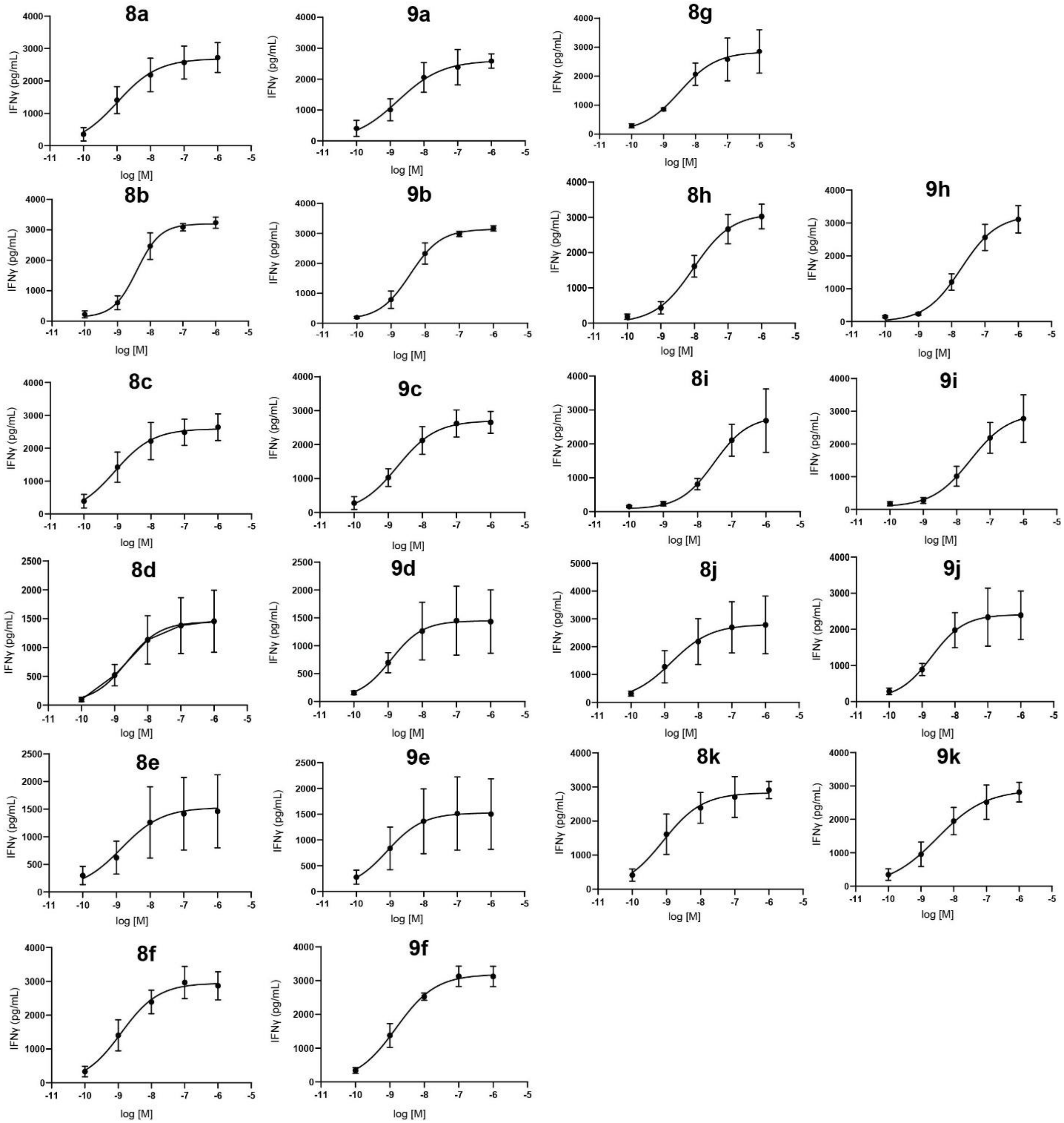

Supplemental Figure S2. ELISA data used to derive EC_{50} values in the main text. IFN-γ levels in culture supernatants were quantified by ELISA following treatment with the indicated compounds. K562 cells were incubated with compounds for 1 h, washed, and mixed with purified effector γδ T cells for 20 hours, after which supernatants were collected and analyzed according to the manufacturer's protocol. Each panel shows IFN-γ secretion for an individual compound. Data are presented as mean ± standard deviation from n=3 independent experiments, each performed in duplicate. Concentrations are indicated on the x-axis.

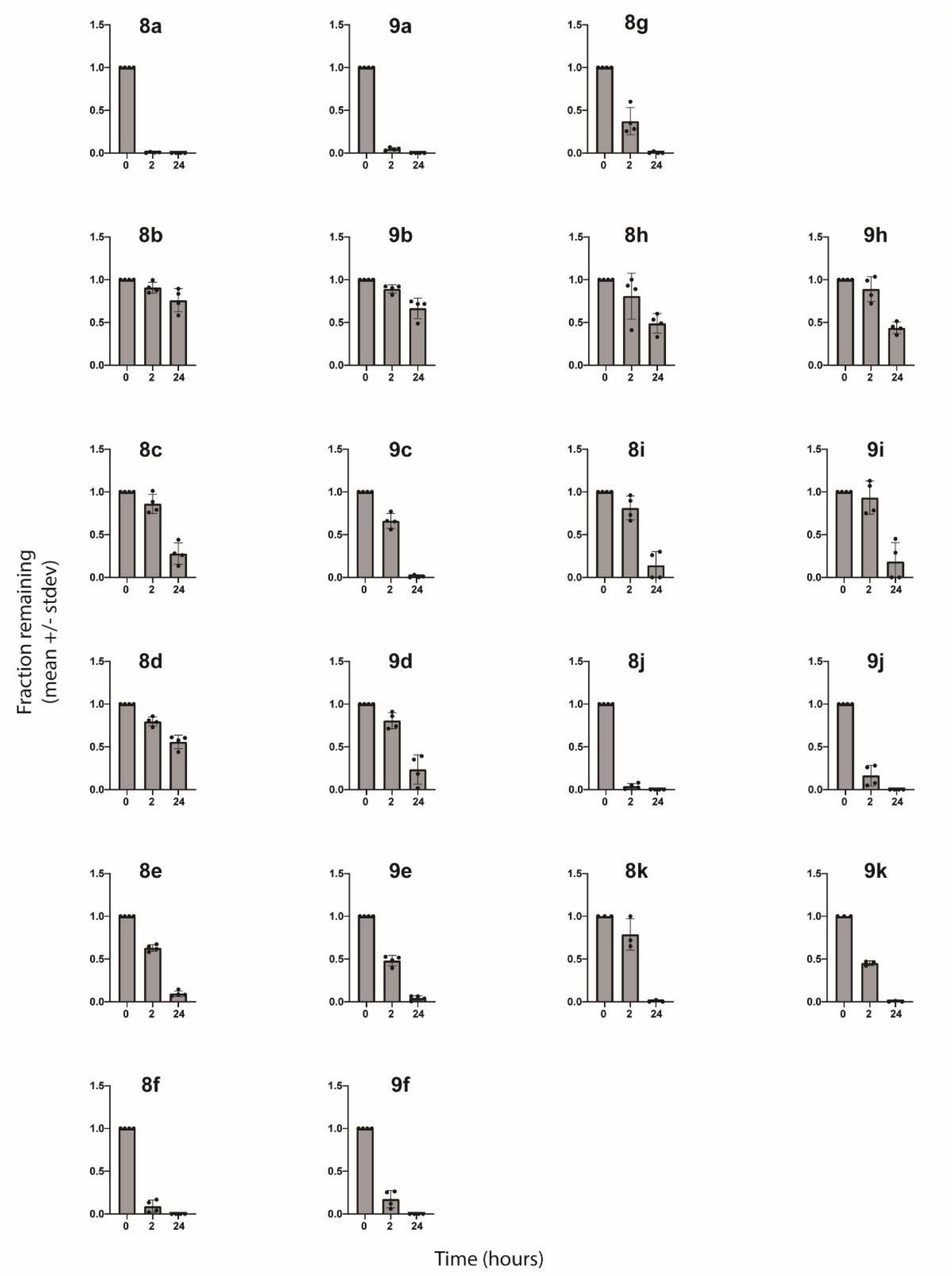

Supplemental Figure S3. Plasma stability data used to derive 2 and 24 hour remaining values in the main text. Compounds were incubated in 50% human plasma in PBS at 37 °C for the indicated times. At each time point, reactions were quenched and remaining parent compound was quantified by LCMS. Each panel shows an individual compound. Data are expressed as fraction of compound remaining relative to time zero and represent mean ± standard deviation from n=4 independent experiments. For compounds **8k** and **9k**, n=3.

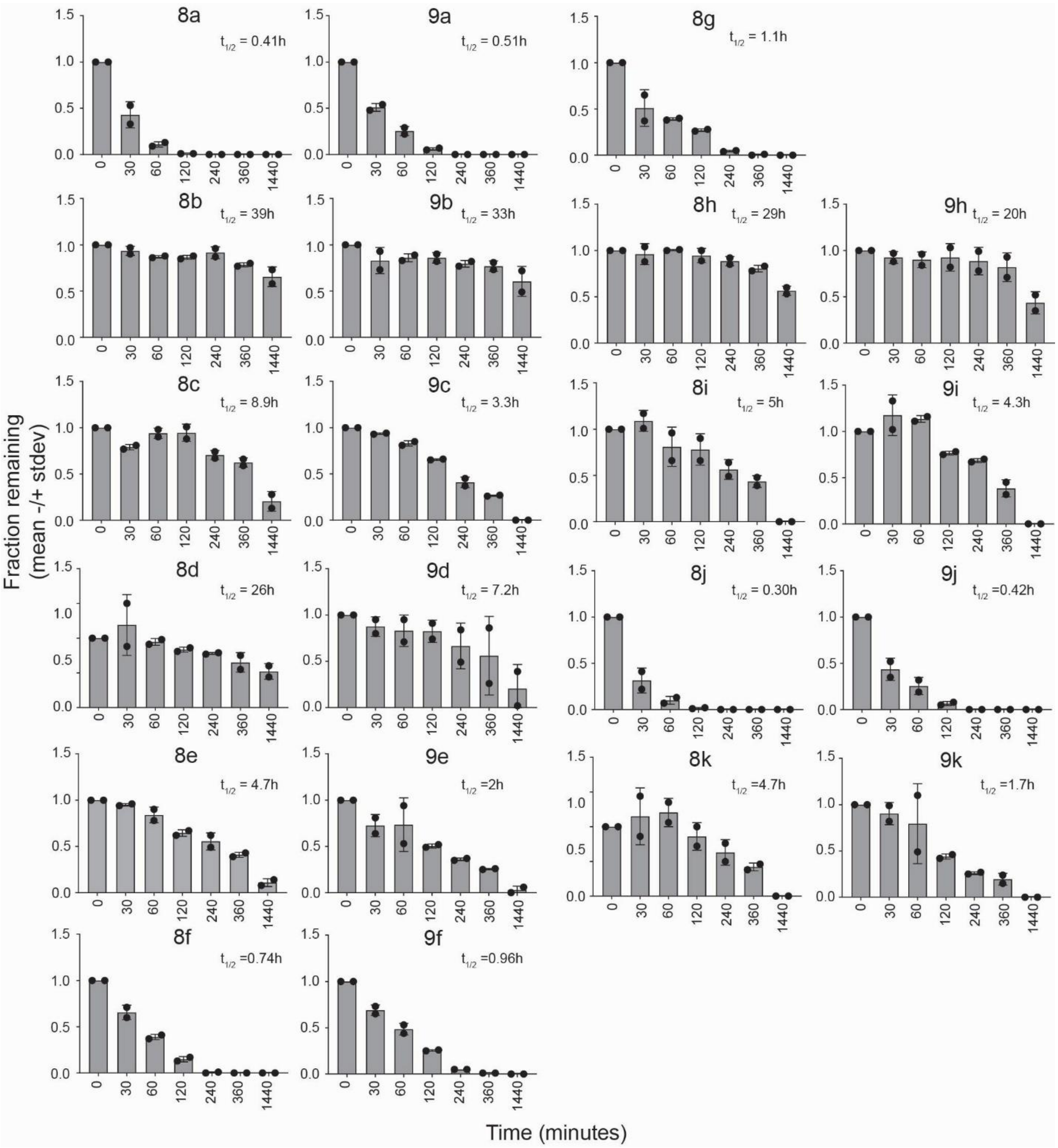

Supplemental Figure S4. Plasma stability data used to derive half life values in the main text. Compounds were incubated in 50% human plasma in PBS at 37 °C for the indicated times. At each time point, reactions were quenched and remaining parent compound was quantified by LCMS. Each panel shows an individual compound. Data are expressed as fraction of compound remaining relative to time zero and represent mean ± standard deviation from n=2 independent experiments.

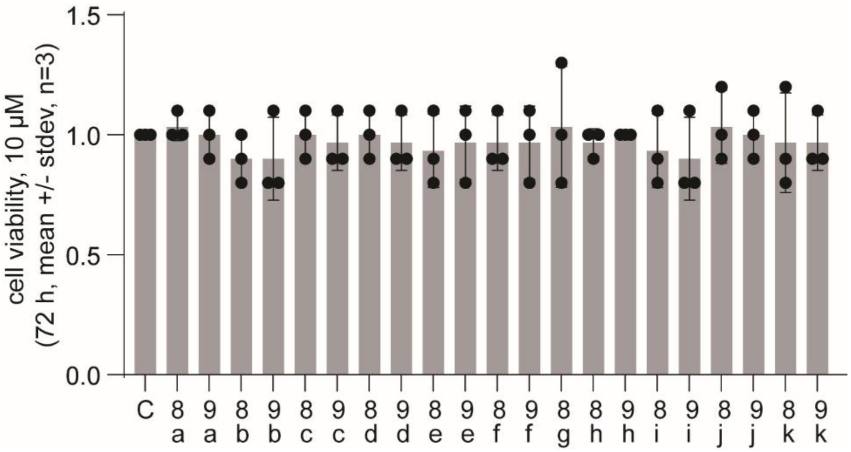

Supplemental Figure S5. 72-hour cell viability. K562 cells were treated with test compounds (10 μM) for 72 hours, then the percentage of viable cells was assessed by Cell QuantiBlue assay (mean +/- stdev, n=3).
